# Supplementary material for: The design of unit cells by combining the self-reproduction systems and metabolic cushioning loads
Source: Commun Biol. 2025 Feb 15;8:241. doi: 10.1038/s42003-025-07655-2 (PMC11830011; doi:10.1038/s42003-025-07655-2)
Supplement: Supplementary file 1 — Supplementary Information [file 42003_2025_7655_MOESM1_ESM.pdf]

SUPPLEMENTARY DISCUSSION 1: LIST OF ABBREVIATIONS 2
SUPPLEMENTARY DISCUSSION 2: LIST OF SYMBOLS 3
SUPPLEMENTARY DISCUSSION 3: LIST OF TERMS..12
SUPPLEMENTARY DISCUSSION 4: LIST OF SHORT DEFINITIONS...13
SUPPLEMENTARY DISCUSSION 5 14
SUPPLEMENTARY DISCUSSION 5.1: OVERVIEW OF RELATIONS BETWEEN PARAMETERS..........14
SUPPLEMENTARY DISCUSSION 5.2: M_U, COMPLEXITY OF SRS AND GROWTH MEDIA..................19
SUPPLEMENTARY DISCUSSION 5.3: MINIMAL CELL SIZE..29
SUPPLEMENTARY DISCUSSION 5.4: CUSHIONING PARAMETER IN UNIT CELLS 30
SUPPLEMENTARY DISCUSSION 5.5: CELL LOAD AND SRS OF THE CELL 32
SUPPLEMENTARY DISCUSSION 5.6: M_{CP}, COMPLEXITY OF SRS AND GROWTH MEDIA 33
SUPPLEMENTARY DISCUSSION 5.7: DESIGN PARAMETERS OF THE UNIT CELL............................39
SUPPLEMENTARY DISCUSSION 5.8: UNIT CELL PARAMETERS, COMPLEXITY OF SRS AND GROWTH MEDIA 48
SUPPLEMENTARY DISCUSSION 5.9: SIMPLIFIED DERIVATION OF T_{CDOPT}....................................99
SUPPLEMENTARY DISCUSSION 5.10: DESCRIPTIONS OF USED MODELS 102
SUPPLEMENTARY DISCUSSION 5.10.1: DESCRIPTION OF SSUCM-SRS-M ... 102
Supplementary Discussion 5.10.1.1: Model components and interactions............102
Supplementary Discussion 5.10.1.1.1: Metabolic network......................................102
Supplementary Discussion 5.10.1.1.2: Protein synthesis 103
Supplementary Discussion 5.10.1.1.3: RNA synthesis..103
Supplementary Discussion 5.10.1.1.4: Lipid synthesis 103
Supplementary Discussion 5.10.1.1.5: Energy balance..104
Supplementary Discussion 5.10.1.1.6: Cell geometry 104
Supplementary Discussion 5.10.1.1.7: Mass balance 104
Supplementary Discussion 5.10.1.1.8: DNA synthesis..104
Supplementary Discussion 5.10.1.2: Model parameters..104
Supplementary Discussion 5.10.1.3: Calculation scheme 106
SUPPLEMENTARY DISCUSSION 5.10.2: DESCRIPTION OF SSUCM-SRS-R ... 110
Supplementary Discussion 5.10.2.1: Model components and interactions............110
Supplementary Discussion 5.10.2.1.1: Metabolic network......................................110
Supplementary Discussion 5.10.2.1.2: Protein synthesis 110
Supplementary Discussion 5.10.2.1.3: RNA synthesis..110
Supplementary Discussion 5.10.2.1.4: Lipid synthesis 110
Supplementary Discussion 5.10.2.1.5: Energy balance..110
Supplementary Discussion 5.10.2.1.6: Cell geometry 110
Supplementary Discussion 5.10.2.1.7: Mass balance 111
Supplementary Discussion 5.10.2.1.8: DNA 111
Supplementary Discussion 5.10.2.2: Model parameters..111
Supplementary Discussion 5.10.2.3: Calculation scheme 111
SUPPLEMENTARY DISCUSSION 5.10.3: DESCRIPTION OF SSUCM-M............ 115
Supplementary Discussion 5.10.3.1: Model components and interactions............115
Supplementary Discussion 5.10.3.1.1: Metabolic network......................................115
Supplementary Discussion 5.10.3.1.2: Protein synthesis 115

Supplementary Discussion 5.10.3.1.3: RNA synthesis *115*
Supplementary Discussion 5.10.3.1.4: Lipid synthesis *116*
Supplementary Discussion 5.10.3.1.5: Energy balance *116*
Supplementary Discussion 5.10.3.1.6: Cell geometry *116*
Supplementary Discussion 5.10.3.1.7: Mass balance *116*
Supplementary Discussion 5.10.3.1.8: DNA synthesis *117*
Supplementary Discussion 5.10.3.2: Model parameters 117
Supplementary Discussion 5.10.3.2.1: Input parameters *117*
Supplementary Discussion 5.10.3.2.2: Output parameters *122*
Supplementary Discussion 5.10.3.3: Calculation scheme 126
SUPPLEMENTARY DISCUSSION 5.10.4: DESCRIPTION OF SSUCM-R 126
Supplementary Discussion 5.10.4.1: Model components and interactions 126
Supplementary Discussion 5.10.4.1.1: Metabolic network *127*
Supplementary Discussion 5.10.4.1.2: Protein synthesis *127*
Supplementary Discussion 5.10.4.1.3: RNA synthesis *127*
Supplementary Discussion 5.10.4.1.4: Lipid synthesis *128*
Supplementary Discussion 5.10.4.1.5: Energy balance *128*
Supplementary Discussion 5.10.4.1.6: Cell geometry *128*
Supplementary Discussion 5.10.4.1.7: Mass balance *128*
Supplementary Discussion 5.10.4.1.8: DNA synthesis *129*
Supplementary Discussion 5.10.4.2: Model parameters 129
Supplementary Discussion 5.10.4.3: Calculation scheme 129
SUPPLEMENTARY REFERENCES **130**

Supplementary Discussion 1: List of abbreviations

CHD – Cooper-Helmstetter-Donachie. Various abbreviations (for example CH[1]) and names (for example Cooper-Helmstetter[1]) have been used in the literature. However, the current abbreviation and name which have been rarely used[2] were selected as they referred, in our opinion, most precisely to the main authors of the corresponding cell cycle theory.
CL – cell load.
CP – cushioning protein.
CPrm – cushioning parameter.
ETC – electron transport chain.
LB – lysogeny broth.
LPE – membrane lipid synthesis enzyme.
RC – replisome complex.
RP – RNA polymerase.
RPC – ribosomal protein complex.
SRS – self-reproduction system of the cell.

SSPCM – simplified single-cell model of proto-cell.
SSPCM-RS+AA – simplified single-cell model of the abstract proto-cell (SRS includes only RPC and amino acid synthesis) from ref. [3].
SSPCM-RS+AA+PROT – simplified single-cell model of the abstract proto-cell (SRS includes only RPC, amino acid synthesis and unspecified protein) from ref. [3].
SSPCM-SRS-M – simplified single-cell model of the abstract proto-cell (SRS includes all main cell components required for self-reproduction) growing on minimal medium[3].
SSPCM-SRS-R – simplified single-cell model of the abstract proto-cell (SRS includes all main cell components required for self-reproduction) growing on rich medium[3].
SSUCM – simplified single-cell model of unit cell.
SSUCM-M – simplified single-cell model of unit cell growing on minimal medium (Fig. 8, Supplementary Discussion 5.10.3).
SSUCM-R – simplified single-cell model of unit cell growing on rich medium (Supplementary Fig. 58, Supplementary Discussion 5.10.4).
SSUCM-SRS-M – simplified single-cell model of self-reproduction system (SRS includes all main cell components required for self-reproduction) of unit cell growing on minimal medium (Supplementary Discussion 5.10.1).
SSUCM-SRS-R – simplified single-cell model of self-reproduction system (SRS includes all main cell components required for self-reproduction) of unit cell growing on rich medium (Supplementary Discussion 5.10.2).
UC – unit cell.

Supplementary Discussion 2: List of symbols

The abbreviations of names of cell components for descriptions of units and indexes of symbols have been written with small letters except for pathway names (PW_1-PW_5). If the description of a symbol does not include terms characterizing values of the corresponding parameter, these parameters either belong to growth dependent parameter class (usually calculated output parameters or independent variables) by default or they are general designations involving various terms or they are not part of the model. The descriptions of units of parameters (separated by square brackets) have some nontrivial features. Besides usual unit symbols (for example g, cm, cm^2, cm^3, s, h), abbreviations (for example aa, dw) and signs (%), other symbols and abbreviations (defined above and below) have been used that are specific for SSUCMs. Superscript $^{-1}$ with (or without) rounded parentheses designate division operation whereas rounded parentheses without superscript express the relationship or belonging between different terms. For example, the unit of *DWC* [g (dw cell) (g (cell))$^{-1}$] must be interpreted as: grams dry weight of cell per gram of cell. The unit of $cell\_comp\%_{mmc}$ [% (g (tot cell comp) (g (dw cell))$^{-1}$)] must be interpreted as: the per cent of macromolecular fraction based on grams of total amounts of all different cell components of the fraction per gram dry weight of the cell. The term "molecules" must be interpreted as "number of molecules".

aa – amino acid (monomer of proteins).
C_{cell_comp} – molar concentration of cell component [mol (cell comp) L^{-1}]. C_{cell_comp} is the general designation for any of the following specific molar concentrations of different cell components: C_{cp}, C_{lpe}, C_{rc}, C_{rce}, C_{rp}, C_{rs}, $C_{rs\_cell\_comp}$, $C_{enz\_PWi\_r}$, C_{etc}, C_{stp}, C_{dna}, C_{rrna}, C_{trna}, $C_{mrna\_cell\_comp}$, C_{lip}, C_{enz}.

C_{cp} – molar concentration of cushioning protein [mol (cp) L^{-1}].
C_{dna} – molar concentration of genome [mol (genome) L^{-1}].
cell_comp – cell component.
cell_comp%$_{fc}$ – total dry weight content of cell component in the corresponding macromolecular fraction [% (g (tot cell comp) (g macromolecular fraction))$^{-1}$)]. *cell_comp%$_{fc}$* is the general designation for any of the following specific fractions of fractional (RNA, protein) compositions: *CP%$_{fc}$*, *RC%$_{fc}$*, *Enz%$_{fc}$*, *ETC%$_{fc}$*, *Lpe%$_{fc}$*, *STP%$_{fc}$*, *RP%$_{fc}$*, *RPC%$_{fc}$*, *rRNA%$_{fc}$*, *tRNA%$_{fc}$*, *mRNA%$_{fc}$*.
cell_comp%$_{mc}$ – total dry weight content of cell component in the cell [% (g (tot cell comp) (g (dw cell))$^{-1}$)]. *cell_comp%$_{mc}$* is the general designation for any of the specific fractions of molecular composition including *CP%$_{mc}$*, *Enz%$_{mc}$*.
cell_comp%$_{mmc}$ – dry weight content of macromolecular fraction in the cell [% (g (tot cell comp) (g (dw cell))$^{-1}$)]. *cell_comp%$_{mmc}$* is the general designation for any of the following specific fractions of macromolecular composition: *DNA%$_{mmc}$*, *LIP%$_{mmc}$*, *PROT%$_{mmc}$*, *RNA%$_{mmc}$*.
C_{enz} – total molar concentration of enzymes [mol (tot enz) L^{-1}].
$C_{enz\_PW1\_r}$ – molar concentration of enzyme catalysing a single reaction r of central metabolic pathway PW_1 [mol (enz PW_1) L^{-1} reaction^{-1}].
$C_{enz\_PW2\_r}$ – molar concentration of enzyme catalysing a single reaction r of amino acid biosynthesis pathway PW_2 [mol (enz PW_2) L^{-1} reaction^{-1}].
$C_{enz\_PW3\_r}$ – molar concentration of enzyme catalysing a single reaction r of deoxyribonucleotide biosynthesis pathway PW_3 [mol (enz PW_3) L^{-1} reaction^{-1}].
$C_{enz\_PW4\_r}$ – molar concentration of enzyme catalysing a single reaction r of ribonucleotide biosynthesis pathway PW_4 [mol (enz PW_4) L^{-1} reaction^{-1}].
$C_{enz\_PW5\_r}$ – molar concentration of enzyme catalysing a single reaction r of lipid biosynthesis pathway PW_5 [mol (enz PW_5) L^{-1} reaction^{-1}].
$C_{enz\_PWi\_r}$ – molar concentration of enzyme catalysing a single reaction r of pathway PW_i [mol (enz PW_i) L^{-1} reaction^{-1}].
C_{etc} – molar concentration of ETC complexes [mol (etc) L^{-1}].
C_{lip} – molar concentration of cell membrane lipid [mol (lip) L^{-1}].
C_{lpe} – molar concentration of lipid synthesis enzyme [mol (lpe) L^{-1}].
$C_{mrna\_cell\_comp}$ – molar concentration of mRNA of protein [mol (mrna prot) L^{-1}]. $C_{mrna\_cell\_comp}$ is the general designation for any of the following specific molar concentrations of mRNAs of different proteins: C_{mrna_cp}, C_{mrna_rc}, C_{mrna_enz}, C_{mrna_etc}, C_{mrna_lpe}, C_{mrna_stp}, C_{mrna_rp}, C_{mrna_rpc}.
C_{mrna_cp} – molar concentration of mRNA of cushioning protein [mol (mrna cp) L^{-1}].
C_{mrna_enz} – molar concentration of mRNA of the enzyme of central and biosynthesis pathways [mol (mrna enz) L^{-1}].
C_{mrna_etc} – molar concentration of mRNA of ETC complex [mol (mrna etc) L^{-1}].
C_{mrna_lpe} – molar concentration of mRNA of lipid synthesis enzyme [mol (mrna lpe) L^{-1}].
C_{mrna_rc} – molar concentration of mRNA of replisome complex [mol (mrna rc) L^{-1}].
C_{mrna_rp} – molar concentration of mRNA of RNA polymerase complex [mol (mrna rp) L^{-1}].
C_{mrna_rpc} – molar concentration of mRNA of a ribosomal protein complex [mol (mrna rpc) L^{-1}].
C_{mrna_stp} – molar concentration of mRNA of transport protein [mol (mrna stp) L^{-1}].
cp – cushioning protein.
CP%$_{fc}$ – total dry weight content of cushioning protein in the protein fraction [% (g (tot cp) (g (tot prot))$^{-1}$)].
CP%$_{mc}$ – total dry weight content of cushioning protein in the cell [% (g (tot cp) (g (dw cell))$^{-1}$)].

C_{rc} – molar concentration of replisome complexes (corresponds to the replisome that is necessary for the periodical replication process during t_C) [mol (rc) L^{-1}].
C_{rce} – molar concentration of effective replisome complexes (corresponds to the replisome that is necessary for the replication process that is averaged over the whole t_{CD} (takes place continuously)) [mol (rce) L^{-1}].
C_{rp} – molar concentration of RNA polymerase complexes [mol (rp) L^{-1}].
C_{rrna} – molar concentration of assembled rRNA complexes [mol (rrna) L^{-1}].
C_{rs} – molar concentration of ribosomes [mol (rs) L^{-1}].
$C_{rs\_cell\_comp}$ – molar concentration of ribosomes for the synthesis of proteins [mol (rs prot) L^{-1}]. $C_{rs\_cell\_comp}$ is the general designation for any of the following specific molar concentrations of ribosomes for the synthesis of different proteins: C_{rs_cp}, C_{rs_rc}, C_{rs_enz}, C_{rs_etc}, C_{rs_lpe}, C_{rs_stp}, C_{rs_rp}, C_{rs_rpc}.
C_{rs_cp} – molar concentration of ribosomes for the synthesis of cushioning proteins [mol (rs cp) L^{-1}].
C_{rs_enz} – molar concentration of ribosomes for the synthesis of enzymes in central and biosynthesis pathways [mol (rs enz) L^{-1}].
C_{rs_etc} – molar concentration of ribosomes for the synthesis of ETC complexes [mol (rs etc) L^{-1}].
C_{rs_lpe} – molar concentration of ribosomes for the synthesis of lipid synthesis enzymes [mol (rs lpe) L^{-1}].
C_{rs_rc} – molar concentration of ribosomes for the synthesis of replisome complexes [mol (rs rc) L^{-1}].
C_{rs_rp} – molar concentration of ribosomes for the synthesis of RNA polymerase complexes [mol (rs rp) L^{-1}].
C_{rs_rpc} – molar concentration of ribosomes for the synthesis of ribosomal protein complexes [mol (rs rpc) L^{-1}].
C_{rs_stp} – molar concentration of ribosomes for the synthesis of transport proteins [mol (rs stp) L^{-1}].
C_{stp} – molar concentration of transport protein complexes [mol (stp) L^{-1}].
C_{trna} – molar concentration of tRNA [mol (trna) L^{-1}].
cyt – cytoplasm.
$DNA\%_{mmc}$ – total dry weight content of DNA fraction in the cell [% (g (tot dna) (g (dw cell))$^{-1}$)].
dnt – deoxyribonucleotide (monomer of DNA).
dp – DNA polymerase.
dw – dry weight.
DWC – dry weight content of the cell (*approximate*, see Supplementary Discussion 3) [g (dw cell) (g (cell))$^{-1}$].
enz – enzyme of central and biosynthesis pathways.
$Enz\%_{fc}$ – total dry weight content of enzymes of central and biosynthesis pathways in the protein fraction [% (g (tot enz) (g (tot prot))$^{-1}$)].
$Enz\%_{mc}$ – total dry weight content of enzymes of central and biosynthesis pathways in the cell [% (g (tot enz) (g (dw cell))$^{-1}$)].
etc – electron transport chain.
$ETC\%_{fc}$ – total dry weight content of electron transport chain complex in the protein fraction [% (g (tot etc) (g (tot prot))$^{-1}$)].
fc – fractional composition.
F_{cell_comp} – metabolic flux of reaction/pathway/process [molecules (metabolite) s^{-1} cell^{-1}]. F_{cell_comp} is the general designation for any of the following specific metabolic fluxes of different reactions/pathways/processes: $F_{enz\_PWi\_r}$, F_{rs}, F_{rce}, F_{etc}, F_{lpe}, F_{stp}, F_{rp}.

$F_{enz\_PW1\_r}$ – flux of reaction r of central metabolic pathway PW_1 [molecules (metabolite) s^{-1} $cell^{-1}$ $reaction^{-1}$].
$F_{enz\_PW2\_r}$ – flux of reaction r of amino acid synthesis pathway PW_2 [molecules (metabolite) s^{-1} $cell^{-1}$ $reaction^{-1}$].
$F_{enz\_PW3\_r}$ – flux of reaction r of deoxyribonucleotide synthesis pathway PW_3 [molecules (metabolite) s^{-1} $cell^{-1}$ $reaction^{-1}$].
$F_{enz\_PW4\_r}$ – flux of reaction r of ribonucleotide synthesis pathway PW_4 [molecules (metabolite) s^{-1} $cell^{-1}$ $reaction^{-1}$].
$F_{enz\_PW5\_r}$ – flux of reaction r of lipid synthesis pathway PW_5 [molecules (metabolite) s^{-1} $cell^{-1}$ $reaction^{-1}$].
$F_{enz\_PWi\_r}$ – flux of reaction r of pathway PW_i [molecules (metabolite) s^{-1} $cell^{-1}$ $reaction^{-1}$].
F_{etc} – ATP synthesis flux of electron transport chain complex [molecules (atp) s^{-1} $cell^{-1}$].
F_{lpe} – lipid synthesis flux [molecules (lip) s^{-1} $cell^{-1}$].
F_{rce} – DNA replication flux [molecules (dnt) s^{-1} $cell^{-1}$].
F_{rp} – transcription flux [molecules (nt) s^{-1} $cell^{-1}$].
F_{rs} – translation flux [molecules (aa) s^{-1} $cell^{-1}$].
F_{stp} – substrate transport flux [molecules (substrate) s^{-1} $cell^{-1}$].
H_{cyl} – length of the cylindrical part of the cell [cm (cell)].
k_{cell_comp} – the apparent working rate of catalysing cell component [molecules (metabolite) s^{-1} (cell comp)$^{-1}$]. k_{cell_comp} is the general designation for any of the specific apparent working rates of different catalysing cell components including k_{enz}, k_{dp}, k_{rp}, k_{rs}, k_{lpe}.
k_{dp} – the apparent working rate of DNA polymerase (*approximate* except for faster growth regions of SSUCM-SRS-R and SSUCM-R if $t_{CD} < t_C = 2320$ s = 0.6 h (Supplementary Discussion 5.10.4.2)) [molecules (dnt) s^{-1} dp^{-1}].
k_{enz} – the apparent working rate of the enzyme catalysing intracellular reactions of central and biosynthesis pathways PW_1 – PW_5 (*generic*, see Supplementary Discussion 3) [molecules (metabolite) s^{-1} enz^{-1}].
k_{lpe} – the apparent working rate of membrane lipid synthesis enzyme (*generic*) [molecules (lip) s^{-1} lpe^{-1}].
k_{rp} – the apparent working rate of RNA polymerase complex (*approximate*) [molecules (nt) s^{-1} rp^{-1}].
k_{rs} – the apparent working rate of ribosome (translation) (*approximate*) [molecules (aa) s^{-1} rs^{-1}].
lip – cell membrane lipid.
$LIP\%_{mmc}$ – total dry weight content of lipid macromolecular fraction in the cell [% (g (tot lip) (g (dw cell))$^{-1}$)].
lpe – membrane lipid synthesis enzyme.
$Lpe\%_{fc}$ – total dry weight content of lipid synthesis enzyme in the protein fraction [% (g (tot lpe) (g (tot prot))$^{-1}$)].
l_{PW2} – number of reactions in amino acid synthesis pathway (i.e. length of the pathway) PW_2 (*generic*) [reactions PW_2^{-1}].
l_{PWi} – number of reactions in linear metabolic pathway (i.e. length of the pathway) PW_i [reactions PW_i^{-1}].
mc – molecular composition.
m_{cp} – mass of cushioning protein molecule (*generic*) [g cp^{-1}].
M_{cp} – total mass of cushioning protein molecules in the cell [g (tot cp) $cell^{-1}$].
M_{cyt} – mass of cytoplasm (cell components and water in cytoplasm) of the cell [g cyt^{-1}].
m_{dna} – mass of genome (*approximate*) [g $genome^{-1}$].
mem – cell membrane.

m_{enz} – mass of a molecule of the enzyme of central and biosynthesis pathways (*generic*) [g enz^{-1}].
m_{etc} – mass of ETC complex (*approximate*) [g etc^{-1}].
m_{lip} – mass of membrane lipid molecule (*generic*) [g lip^{-1}].
m_{lpe} – mass of lipid synthesis enzyme molecule (*generic*) [g lpe^{-1}].
mmc – macromolecular composition.
M_{mem} – mass of cell membrane (cell components and water in cell membrane) [g mem^{-1}].
$m_{mrna\_cell\_comp}$ – mass of a molecule of mRNA of protein [g (mrna cell comp)$^{-1}$]. $m_{mrna\_cell\_comp}$ is the general designation for any of the following specific masses of mRNA molecules of different proteins: m_{mrna_rc}, m_{mrna_cp}, m_{mrna_enz}, m_{mrna_etc}, m_{mrna_lpe}, m_{mrna_stp}, m_{mrna_rp}, m_{mrna_rpc}.
m_{mrna_cp} – mass of a molecule of mRNA of cushioning protein (*generic*) [g (mrna cp)$^{-1}$].
m_{mrna_enz} – mass of a molecule of mRNA of the enzyme of central and biosynthesis pathways (*generic*) [g (mrna enz)$^{-1}$].
m_{mrna_etc} – mass of a molecule of mRNA of ETC complex (*approximate*) [g (mrna etc)$^{-1}$].
m_{mrna_lpe} – mass of a molecule of mRNA of lipid synthesis enzyme (*generic*) [g (mrna lpe)$^{-1}$].
m_{mrna_rc} – mass of a molecule of mRNA of replisome complex (*approximate*) [g (mrna rc)$^{-1}$].
m_{mrna_rp} – mass of a molecule of mRNA of RNA polymerase complex (*approximate*) [g (mrna rp)$^{-1}$].
m_{mrna_rpc} – mass of a molecule of mRNA of ribosomal protein complex (*approximate*) [g (mrna rpc)$^{-1}$].
m_{mrna_stp} – mass of a molecule of mRNA of transport protein (*approximate*) [g (mrna stp)$^{-1}$].
mprot – membrane protein (ETC, substrate transport protein).
M_{prot} – total mass of protein molecules in the cell [g (tot prot) $cell^{-1}$].
m_{rc} – mass of replisome complex (*approximate*) [g rc^{-1}].
M_{rna} – total mass of RNA molecules in the cell [g (tot rna) $cell^{-1}$].
$mRNA\%_{fc}$ – total dry weight content of mRNAs in the RNA fraction [% (g (tot mrna) (g (tot rna))$^{-1}$)].
m_{rp} – mass of RNA polymerase complex (*approximate*) [g rp^{-1}].
m_{rpc} – mass of ribosomal protein complex (*approximate*) [g rpc^{-1}].
m_{rrna} – mass of assembled rRNA complex (*approximate*) [g $rrna^{-1}$].
M_{srs} – mass of the self-reproduction system of the cell [g srs^{-1}].
m_{stp} – mass of transport protein complex (*approximate*) [g stp^{-1}].
M_{tot} – cell mass (cell components and water in the cell) [g $cell^{-1}$].
M_{tot_ave} – average cell mass (mass of cell components and water in the cell, mass of cell with average cell age in the cell culture) [g $cell^{-1}$].
m_{trna} – mass of tRNA molecule (*approximate*) [g $trna^{-1}$].
M_u – unit cell mass (mass of a cell with cell cycle duration t_{CD}) (cell components and water in the cell) [g (unit cell)$^{-1}$].
N_A – Avogadro constant (*specific, precise*, see (Supplementary Discussion 3)) [molecules mol^{-1}].
N_{aa_prot} – number of molecules of polymerized amino acid of unspecified protein in the cell [molecules (aa prot) $cell^{-1}$].
N_{cell_comp} – number of cell component molecules/complexes in the cell [molecules (cell comp) $cell^{-1}$]. N_{cell_comp} is the general designation for any of the specific numbers of different cell component molecules/complexes including N_{cp}, N_{lpe}, N_{rc}, N_{rce}, N_{rp}, N_{rs}, $N_{rs\_cell\_comp}$, $N_{enz\_PWi\_r}$, N_{etc}, N_{stp}, N_{dna}, N_{rrna}, N_{trna}, $N_{mrna\_cell\_comp}$, N_{lip}.
n_{cp} – number of amino acid molecules in (i.e. length of the amino acid sequence of) the cushioning protein molecule (*generic*) [molecules (aa) cp^{-1}].
N_{cp} – number of cushioning protein molecules in the cell [molecules (cp) $cell^{-1}$].

n_{dna} – number of deoxyribonucleotide molecules in (i.e. length of deoxyribonucleotide sequence of) the genome (*specific, precise*) [molecules (dnt) genome^{-1}] or [molecules (bp) genome^{-1}].
N_{dna} – number of genomes (genome copy number) in the cell (*specific, precise*) [molecules (genome) cell^{-1}]. The designation of ref. [3] was used for the parameter instead of the former designation[1].
n_{enz} – number of amino acid molecules in (i.e. length of the amino acid sequence of) the molecule of the enzyme catalysing reactions of central pathway and biosynthesis pathways PW_1 – PW_5 (*generic*) [molecules (aa) enz^{-1}].
N_{enz} – total number of enzyme molecules in the cell [molecules (tot enz) cell^{-1}].
$N_{enz\_PW1\_r}$ – number of molecules of the enzyme catalysing a single reaction r of central metabolic pathway PW_1 [molecules (enz PW_1) cell^{-1} reaction^{-1}].
$N_{enz\_PW2\_r}$ – number of molecules of the enzyme catalysing a single reaction r of amino acid biosynthesis pathway PW_2 [molecules (enz PW_2) cell^{-1} reaction^{-1}].
$N_{enz\_PW2\_r\_e}$ – number of effective molecules of the enzyme catalysing a single reaction r of amino acid biosynthesis pathway PW_2 (corresponds to the enzymes that are necessary for amino acid biosynthesis) [molecules (enz PW_2 e) cell^{-1} reaction^{-1}].
$N_{enz\_PW2\_r\_min}$ – minimal number of molecules of the enzyme catalysing a single reaction r of amino acid biosynthesis pathway PW_2 [molecules (enz PW_2) cell^{-1} reaction^{-1}].
$N_{enz\_PW3\_r}$ – number of molecules of the enzyme catalysing a single reaction r of deoxyribonucleotide biosynthesis pathway PW_3 [molecules (enz PW_3) cell^{-1} reaction^{-1}].
$N_{enz\_PW4\_r}$ – number of molecules of the enzyme catalysing a single reaction r of ribonucleotide biosynthesis pathway PW_4 [molecules (enz PW_4) cell^{-1} reaction^{-1}].
$N_{enz\_PW5\_r}$ – number of molecules of the enzyme catalysing a single reaction r of lipid biosynthesis pathway PW_5 [molecules (enz PW_5) cell^{-1} reaction^{-1}].
$N_{enz\_PWi\_r}$ – number of molecules of the enzyme catalysing a single reaction r of pathway PW_i [molecules (enz PW_i) cell^{-1} reaction^{-1}].
n_{etc} – number of amino acid molecules in (i.e. length of the amino acid sequence of) the ETC complex (*approximate*) [molecules (aa) etc^{-1}].
N_{etc} – number of ETC complexes in the cell [molecules (etc) cell^{-1}].
N_{lip} – number of cell membrane lipid molecules in the cell [molecules (lip) cell^{-1}].
n_{lpe} – number of amino acid molecules in (i.e. length of the amino acid sequence of) the lipid synthesis enzyme molecule (*generic*) [molecules (aa) lpe^{-1}].
N_{lpe} – number of lipid synthesis enzyme molecules in the cell [molecules (lpe) cell^{-1}].
$N_{mrna\_cell\_comp}$ – number of molecules of mRNA of protein in the cell [molecules (mrna prot) cell^{-1}]. $N_{mrna\_cell\_comp}$ is the general designation for any of the following specific numbers of mRNA molecules of different proteins: N_{mrna_cp}, N_{mrna_rc}, N_{mrna_enz}, N_{mrna_etc}, N_{mrna_lpe}, N_{mrna_stp}, N_{mrna_rp}, N_{mrna_rpc}.
$n_{mrna\_cell\_comp}$ – number of ribonucleotide molecules in (i.e. length of ribonucleotide sequence of) the molecule of mRNA of protein [molecules (nt) (mrna prot)$^{-1}$]. $n_{mrna\_cell\_comp}$ is the general designation for any of the following specific numbers of ribonucleotide molecules in mRNA molecule of different proteins: n_{mrna_cp}, n_{mrna_rc}, n_{mrna_enz}, n_{mrna_etc}, n_{mrna_lpe}, n_{mrna_stp}, n_{mrna_rp}, n_{mrna_rpc}.
N_{mrna_cp} – number of molecules of mRNA of cushioning protein in the cell [molecules (mrna cp) cell^{-1}].
n_{mrna_cp} – number of ribonucleotide molecules in (i.e. length of ribonucleotide sequence of) the molecule of mRNA of cushioning protein (*generic*) [molecules (nt) (mrna cp)$^{-1}$].
N_{mrna_enz} – number of molecules of mRNA of the enzyme of central and biosynthesis pathways in the cell [molecules (mrna enz) cell^{-1}].

n_{mrna_enz} – number of ribonucleotide molecules in (i.e. length of ribonucleotide sequence of) the molecule of mRNA of the enzyme of central and biosynthesis pathways (*generic*) [molecules (nt) (mrna enz)$^{-1}$].
N_{mrna_etc} – number of molecules of mRNA of ETC complex in the cell [molecules (mrna etc) cell^{-1}].
n_{mrna_etc} – number of ribonucleotide molecules in (i.e. length of ribonucleotide sequence of) the molecule of mRNA of ETC complex (*approximate*) [molecules (nt) (mrna etc)$^{-1}$].
N_{mrna_lpe} – number of molecules of mRNA of lipid synthesis enzyme in the cell [molecules (mrna lpe) cell^{-1}].
n_{mrna_lpe} – number of ribonucleotide molecules in (i.e. length of ribonucleotide sequence of) the molecule of mRNA of lipid synthesis enzyme (*generic*) [molecules (nt) (mrna lpe)$^{-1}$].
N_{mrna_rc} – number of molecules of mRNA of replisome complex in the cell [molecules (mrna rc) cell^{-1}].
n_{mrna_rc} – number of ribonucleotide molecules in (i.e. length of ribonucleotide sequence of) the molecule of mRNA of replisome complex (*approximate*) [molecules (nt) (mrna rc)$^{-1}$].
N_{mrna_rp} – number of molecules of mRNA of RNA polymerase complex in the cell [molecules (mrna rp) cell^{-1}].
n_{mrna_rp} – number of ribonucleotide molecules in (i.e. length of ribonucleotide sequence of) the molecule of mRNA of RNA polymerase complex (*approximate*) [molecules (nt) (mrna rp)$^{-1}$].
N_{mrna_rpc} – number of molecules of mRNA of a ribosomal protein complex in the cell [molecules (mrna rpc) cell^{-1}].
n_{mrna_rpc} – number of ribonucleotide molecules in (i.e. length of ribonucleotide sequence of) the molecule of mRNA of ribosomal protein complex (*approximate*) [molecules (nt) (mrna rpc)$^{-1}$].
N_{mrna_stp} – number of molecules of mRNA of transport protein in the cell [molecules (mrna stp) cell^{-1}].
n_{mrna_stp} – number of ribonucleotide molecules in (i.e. length of ribonucleotide sequence of) the molecule of mRNA of transport protein (*approximate*) [molecules (nt) (mrna stp)$^{-1}$].
n_{rc} – number of amino acid molecules in (i.e. length of the amino acid sequence of) the replisome complex (*approximate*) [molecules (aa) rc^{-1}].
N_{rc} – number of replisome complexes (corresponds to the replisomes that are necessary for the periodical replication process during t_C) in the cell (*specific, precise*) [molecules (rc) cell^{-1}].
N_{rce} – number of effective replisome complexes (corresponds to the replisomes that are necessary for the replication process that is averaged over the whole t_{CD} (takes place continuously)) in the cell (*specific, precise* for faster growth regions of SSUCM-SRS-R and SSUCM-R if $t_{CD} < t_C$ = 2320 s = 0.6 h (Supplementary Discussion 5.10.4.2)) [molecules (rce) cell^{-1}].
n_{rp} – number of amino acid molecules in (i.e. length of the amino acid sequence of) the RNA polymerase complex (*approximate*) [molecules (aa) rp^{-1}].
N_{rp} – number of RNA polymerase complexes in the cell [molecules (rp) cell^{-1}].
n_{rpc} – number of amino acid molecules in (i.e. length of the amino acid sequence of) the ribosomal protein complex (*approximate*) [molecules (aa) rpc^{-1}].
N_{rrna} – number of assembled rRNA complexes in the cell [molecules (rrna) cell^{-1}].
n_{rrna} – number of ribonucleotide molecules in (i.e. length of ribonucleotide sequence of) the assembled rRNA complex (*specific, precise*) [molecules (nt) rrna^{-1}].
N_{rs} – number of ribosomes (equal for ribosomal protein complexes) in the cell [molecules (rs) cell^{-1}].

N_{rs_ave} – average number of ribosomes in the cell (number of ribosomes of the cell with average cell age in exponentially growing cell culture) [molecules (rs) cell^{-1}].
$N_{rs\_cell\_comp}$ – number of ribosomes for the synthesis of protein in the cell [molecules (rs prot) cell^{-1}]. $N_{rs\_cell\_comp}$ is the general designation for any of the following specific numbers of ribosomes for the synthesis of different proteins: N_{rs_cp}, N_{rs_rc}, N_{rs_enz}, N_{rs_etc}, N_{rs_lpe}, N_{rs_stp}, N_{rs_rp}, N_{rs_rpc}.
N_{rs_cp} – number of ribosomes for the synthesis of cushioning protein [molecules (rs cp) cell^{-1}].
N_{rs_enz} – number of ribosomes for the synthesis of enzymes in central and biosynthesis pathways [molecules (rs enz) cell^{-1}].
N_{rs_etc} – number of ribosomes for the synthesis of ETC complexes [molecules (rs etc) cell^{-1}].
N_{rs_lpe} – number of ribosomes for the synthesis of lipid synthesis enzymes [molecules (rs lpe) cell^{-1}].
N_{rs_min} – minimal number of ribosomes (equal for ribosomal protein complexes) in the cell [molecules (rs) cell^{-1}].
N_{rs_rc} – number of ribosomes for the synthesis of replisome complexes [molecules (rs rc) cell^{-1}].
N_{rs_rp} – number of ribosomes for the synthesis of RNA polymerase complexes [molecules (rs rp) cell^{-1}].
N_{rs_rpc} – number of ribosomes for the synthesis of ribosomal proteins [molecules (rs rpc) cell^{-1}].
N_{rs_stp} – number of ribosomes for the synthesis of transport proteins [molecules (rs stp) cell^{-1}].
N_{rse} – number of effective ribosomes (equal for effective ribosomal protein complexes, corresponds to the ribosomes that are necessary for the translation process) in the cell [molecules (rse) cell^{-1}].
n_{stp} – number of amino acid molecules in (i.e. length of the amino acid sequence of) the transport protein complex (*approximate*) [molecules (aa) stp^{-1}].
N_{stp} – number of transport protein complexes in the cell [molecules (stp) cell^{-1}].
nt – ribonucleotide (monomer of RNA).
n_{trna} – number of ribonucleotide molecules in (i.e. length of ribonucleotide sequence of) the tRNA molecule (*average*, see Supplementary Discussion 3) [molecules (nt) trna^{-1}].
N_{trna} – number of tRNA molecules in the cell [molecules (trna) cell^{-1}].
P_{cell_comp} – polysome density of mRNA of protein [molecules (nt (covered by rs)) (molecules (nt (covered by tot rs)))$^{-1}$]. P_{cell_comp} is the general designation for any of the specific polysome densities of mRNAs of different proteins including P_{cp}.
P_{cp} – polysome density of mRNA of cushioning protein (*generic*) [molecules (nt (covered by rs)) (molecules (nt (covered by tot rs)))$^{-1}$].
P_{cp_min} – minimum polysome density of mRNA of cushioning protein [molecules (nt (covered by rs)) (molecules (nt (mrna cp)$^{-1}$))$^{-1}$].
prot – protein (CP, replisome complex, enzyme of metabolic pathways, electron transport chain, lipid synthesis enzyme, transport protein, RNA polymerase complex, ribosome protein complex).
$PROT\%_{mmc}$ – total dry weight content of protein fraction in the cell [% (g (tot prot) (g (dw cell))$^{-1}$)].
PW_1 – central metabolic pathway consisting of a linear chain of reactions (synthesis of building blocks from substrate).
PW_2 – amino acid synthesis pathway consisting of a linear chain of reactions (synthesis of amino acids from building blocks).
PW_3 – deoxyribonucleotide synthesis pathway consisting of a linear chain of reactions (synthesis of deoxyribonucleotides from building blocks).

PW_4 – ribonucleotide synthesis pathway consisting of a linear chain of reactions (synthesis of ribonucleotides from building blocks).
PW_5 – lipid synthesis pathway consisting of a linear chain of reactions (synthesis of lipids from building blocks).
PW_i – metabolic pathway i consisting of a linear chain of reactions.
Q_{cp} – specific productivity of cushioning protein synthesis [molecules (cp) (g (cell))$^{-1}$ h^{-1}].
Q_{cp_max} – maximum specific productivity of cushioning protein synthesis [molecules (cp) (g cell)$^{-1}$ h^{-1}].
rc – replisome complex. Corresponds to the replisomes that are necessary for the periodical replication process.
$RC\%_{fc}$ – total dry weight content of replisome complex in the protein fraction [% (g (tot rc) (g (tot prot))$^{-1}$)].
rce – effective replisome complex. Corresponds to the replisomes that are necessary for the replication process that is averaged over t_{CD} (takes place continuously).
$RNA\%_{mmc}$ – total dry weight content of RNA fraction in the cell [% (g (tot rna) (g (dw cell))$^{-1}$)].
rp – RNA polymerase complex.
$RP\%_{fc}$ – total dry weight content of RNA polymerase complex in the protein fraction [% (g (tot rp) (g (tot prot))$^{-1}$)].
rpc – ribosomal protein complex.
$RPC\%_{fc}$ – total dry weight content of ribosomal protein complex in the protein fraction [% (g (tot rpc) (g (tot prot))$^{-1}$)].
$rRNA\%_{fc}$ – total dry weight content of assembled rRNA complex in the RNA fraction [% (g (tot rrna) (g (tot rna))$^{-1}$)].
rs – ribosome.
rse – effective ribosome. Corresponds to the ribosomes that are necessary for the translation process.
R_{tot} – cell radius [cm cell^{-1}].
S_{etc} – the total cell membrane surface area covered by ETC complexes [cm^2 (tot etc)$^{-1}$].
S_{lip} – the total cell membrane surface area covered by membrane lipids [cm^2 (tot lip)$^{-1}$].
S_{mem_i} – the surface area of the internal layer of the cell membrane [cm^2 cell^{-1}].
S_{prot} – the total cell membrane surface area covered by membrane proteins (ETC, transporters) [cm^2 (tot mprot)$^{-1}$].
srs – self-replication system.
S_{stp} – the total cell membrane surface area covered by transport proteins [cm^2 (tot stp)$^{-1}$].
S_{tot} – the surface area of the cell (membrane) [cm^2 cell^{-1}].
stp – substrate transport protein.
$STP\%_{fc}$ – total dry weight content of transporter protein in the protein fraction [% (g (tot stp) (g (tot prot))$^{-1}$)].
t_0 – cell age at the beginning of cell cycle [s].
t_a – cell age [s]. The parameter was originally designated as *a* or α (for example[4]) in previous works but the designation was changed in the current work for clarification purposes (*t* is an established designation for time in the literature).
t_{a_ave} – average cell age in the cell culture [s].
t_C – genome replication time (the period between DNA replication initiation and termination of a single genome) (*approximate* except for faster growth regions of SSUCM-SRS-R and SSUCM-R if t_{CD} < 2320 s = 0.6 h) [s and h] or [s genome^{-1}]. The designation of ref. [3] was used for the parameter instead of the former designation[1].
t_{CD} – cell cycle duration or length (doubling time) of unit cell, cell age at the end of the cell cycle [s and h].

t_{CDmin} – minimal cell cycle duration or length of unit cell, cell cycle length on the growth boundary of unit cell [s and h].
t_{CDopt} – cell cycle duration or length of the unit cell at Q_{cp_max} [s and h].
T_{cp} – maximal relative productivity of cushioning protein synthesis calculated according to Supplementary Eq. (10) [s s^{-2}].
t_d – cell cycle duration or length (doubling time), cell age at the end of the cell cycle [s and h]. The designation of ref. [3] was used for the parameter instead of the former designation[1].
t_D – division time (period from DNA replication termination until the end of cell division) (*approximate* for the calculations if t_{CD} = 3520 s = 1.0 h) [s and h]. The parameter was originally designated as *D* in previous works (for example[5]) but the designation was changed in the current work for clarification purposes (*t* is an established designation for time in the literature).
t_{d_rs} – doubling time of SSPCM-RS or doubling time of a single RPC [s and h] or [s rpc^{-1}].
t_{d_rs+PW2} – doubling time of SSPCM-RS+AA [s].
t_{d_srs} – doubling time of the self-reproduction system of the abstract proto-cell [s and h]. t_{d_srs} is the general designation for any of the specific doubling times of different self-reproduction systems including t_{d_srs-m}, t_{d_srs-r}, t_{d_rs}, t_{d_rs+PW2}.
t_{d_srs-m} – doubling time of SSPCM-SRS-M [s and h].
t_{d_srs-r} – doubling time of SSPCM-SRS-R [s and h].
tot – total. In the case of symbols, tot designates whole-cell parameters (for example M_{tot} as cell mass). In the case of unit descriptions, tot designates the total amount of all different (if available) necessary cell components that belong to the corresponding fraction (for example [g (tot cp) $cell^{-1}$] as the mass of all cushioning protein molecules in the cell).
$tRNA\%_{fc}$ – total dry weight content of tRNA in the RNA fraction [% (g (tot trna) (g (tot rna))$^{-1}$)].
t_{se} – abstract period spent for the rearrangements of SRS in the robust estimation of T_{cp} [s and h].
V_{tot} – cell volume (cell components and water in the cell) [cm^3 $cell^{-1}$].
V_{tot_ave} – average cell volume (volume of cell components and water in the cell, volume of cell with average cell age in exponentially growing cell culture) [cm^3 $cell^{-1}$].
V_{u_ave} – unit cell volume of average cell in the cell culture [μm^3 $cell^{-1}$].
μ – specific growth rate of the cell culture [h^{-1}].
ρ_{tot} – cell density (*generic*) [g (cell) (cm^3 (cell))$^{-1}$].

Supplementary Discussion 3: List of terms

The following list of terms[3] is used to characterize cellular entities (cell components, cellular processes, cellular interactions) and their properties (values of parameters) in developed SSUCMs.

Approximate – corresponds to entities and properties that belong to the usual range of varying values of *E. coli* cells. The sources of variations in values include different *E. coli* K12 strains (growth independent physiological parameters, part of the input parameter values of used models), effects of cell growth and changes in the growth environment (growth dependent physiological parameters, part of the input and output parameter values of used models). The range of values is only possible due to the lack of information about *E. coli* K12 MG1655, not well described (exact) effects of cell growth and changes in the growth environment, simplifications made in the models. The values with this term comprised part of the input parameter values of used models.

Average – corresponds to the arithmetic mean of entities and properties of *E. coli* K12 MG1655. Again, the term is involved for the same parameter types and used models as mentioned before. Average enables to reduce the complexity (number of components, relations and parameters) of the models but maintains principal relations between cell components.
Generic – corresponds to entities and properties that are not related to any certain strain of *E. coli* K12 but are still generally acceptable and correspond to the known biological range.
Specific, precise – corresponds to entities and properties of *E. coli* K12 MG1655. The term is usually utilized for growth independent physiological parameters and part of the input parameter values of used models.

Supplementary Discussion 4: List of short definitions

Cell load (CL) — here as a collection of cell components that are not directly necessary for cell self-reproduction and of unused (i.e. currently inactive) cell components from the SRS of the cell. In the current work, CL is manifested as generic cushioning protein (CP) in the used SSUCMs. Possible examples of CL in real prokaryotic cells might involve different proteins with various secondary functions in terms of self-reproduction, reserve materials, just-in-case pathways and transporters, idle ribosomes, recombinant proteins, etc.
Complexity of SRS — the total number of species of all relevant cell components and respective cell processes belonging to the SRS. The complexity of SRS is independent of cell growth in the used models. Indirectly, this term characterizes the size of the metabolic network (number of reactions) and growth media type. Rich growth medium (includes monomers of macromolecules) corresponds to simpler SRS while minimal growth medium requires more complex SRS.
Cushioning parameter (CPrm) — a central concept and parameter(s) (cell growth dependent physiological parameter(s)) in SSUCM that secures reasonable cell sizes, cellular concentrations and slower growth rates. Indirectly it represents the united functioning of CL components. It should be stressed that CPrm does not correspond to a homonymic mining industry term with the same name but a completely different meaning[6].
Cushioning protein (CP) — the generic protein in the model cell that is not directly necessary for the self-reproduction of the cell and does not have any specific function in the cell. CP is an abstract possible example of the manifestation of the CPrm and CL. Possible examples of CP in real prokaryotic cells might involve different proteins with various secondary functions. It should be stressed that CP does not correspond to homonymic medical (joint cushioning)[7] and reproduction biology (egg coats)[8] terms.
Proto-cell — a cell (model) that consists of only SRS (essential cell components necessary for the self-reproduction of the cell) and lacks all other cell components and processes which can be found in living cells. Note that the term proto-cell has been used relatively loosely in the literature to refer to primitive cells or the first cells[9].
Self-reproduction system (SRS) — a set of essential cell components with functions (including polymerisation of macromolecules and synthesis of respective monomers/metabolites, energy synthesis, substrate transport, cell membrane formation) directly necessary for cell growth and self-reproduction at given growth conditions. Cell components and processes that are not directly necessary for self-reproduction (such as regulation) do not belong to SRS including those cell components that are necessary for self-reproduction but at different growth conditions (such as metabolic pathways for different

media). It should be stressed that SRS does not correspond to homonymic computer science[10], vesicle formation[11], viral protein assemblies[12] and complexity theory[13] terms.
Size of SRS — the total number of molecules/complexes or the total mass of all relevant cell components belonging to the synthesis equipment or SRS. The size of SRS is dependent on cell growth in the used models. Size generally increases during faster growth because there is less time to finish all the required cellular processes and, therefore, the numbers of catalysts increase to compensate for the lack of time. Note that not all sizes of subparts of SRS increase monotonously during faster growth (lipid synthesis depends on the change of surface/volume change and available free space on the cell membrane, DNA replication is required only for a single genome due to the unit cell condition studied in this paper).
Unit cell (UC) — refers to a cell growing at t_{CD}. A single genome is synthesized by a pair of replisome complexes during the cell cycle of UC. DNA replication starts at cell age t_0 and lasts a time period t_C, then a time period t_D follows during which cell division is prepared and carried out, and at the end of t_D the cell division processes get completed (i.e. the daughter cells become fully separated) at cell age $t_{CD} = t_C + t_D$. Note that M_u (cell size parameter of UC) is identical to the same parameter in CHD cell cycle theory.

Supplementary Discussion 5

Supplementary Discussion 5.1: Overview of relations between parameters

The dependencies between main parameters at cell age t_0 (visualized in Supplementary Fig. 1) were shortly as follows assuming that the apparent working rates were constant:

1. The values of M_u and N_{rs} decreased non-linearly with the increase of t_{CD} (and t_D) at constant N_{cp} because less synthesis equipment was needed for the doubling of the cell during slower growth (the size of SRS decreased).
2. The values of M_u and N_{cp} increased linearly with the increase of t_{CD} (and t_D) at constant N_{rs} because more synthesis equipment was available for the doubling of the cell during slower growth (the size of SRS was relatively constant).
3. The values of M_u and N_{rs} increased linearly with the increase of N_{cp} at constant t_{CD} (and t_D) because more synthesis equipment was needed for the doubling of the cell. Similarly, the values of M_u and N_{cp} increased linearly with the increase of N_{rs} in the cell at constant t_{CD} (and t_D).
4. Faster growth (decrease of t_{CD} values) or increasing cell load (increase of N_{cp} values) was accompanied with the increase of values of most of the fluxes, numbers of molecules in the cell and cell dimensions except those of DNA (did not change due to single genome in the cell) and membrane lipids (complicated dependences due change of the ratio of surface to volume and due to requirement of membrane proteins).

Obviously, visualized interactions involving CP do not correspond to SSUCM-SRS-M and SSUCM-SRS-R because these models include only components of SRS. The dependencies between t_{CD} and other cell parameters are qualitatively similar between models but not quantitatively. Quantitative differences are the effects of different complexities of SRS and cell load which are considered important cell design elements and their exploration is one of the main tasks in this work. Note that the dependencies involving molar concentrations are much more complex compared to numbers of molecules in the cell.

(a)

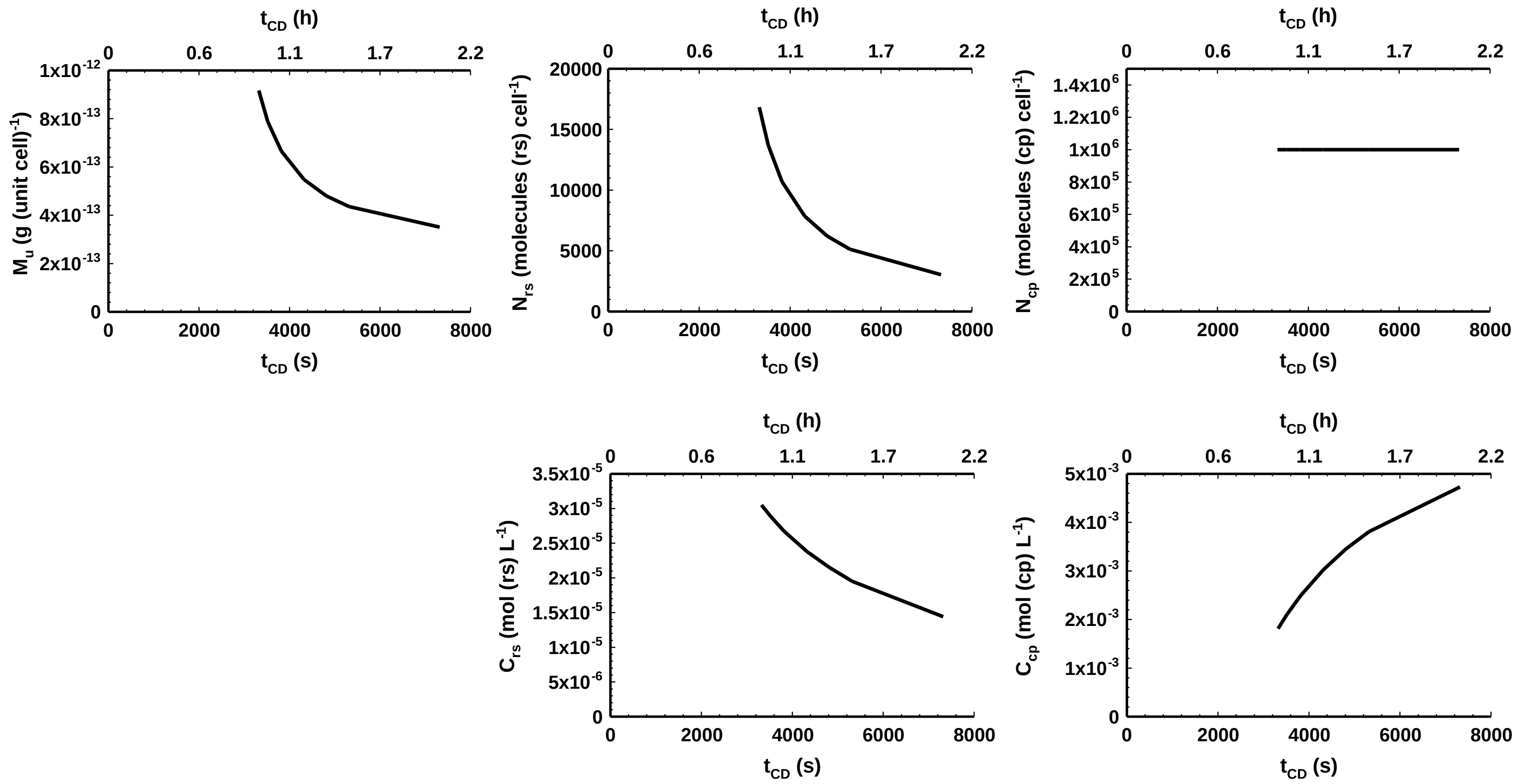

t_CD (h)
0
0.6
1.1
1.7
2.2
M_u (g (unit cell)^-1)
1x10^-12
8x10^-13
6x10^-13
4x10^-13
2x10^-13
0
2000
4000
6000
8000
t_CD (s)
N_rs (molecules (rs) cell^-1)
20000
15000
10000
5000
N_cp (molecules (cp) cell^-1)
1.4x10^6
1.2x10^6
1x10^6
8x10^5
6x10^5
4x10^5
2x10^5
C_rs (mol (rs) L^-1)
3.5x10^-5
3x10^-5
2.5x10^-5
2x10^-5
1.5x10^-5
1x10^-5
5x10^-6
C_cp (mol (cp) L^-1)
5x10^-3
4x10^-3
3x10^-3
2x10^-3
1x10^-3

(b)

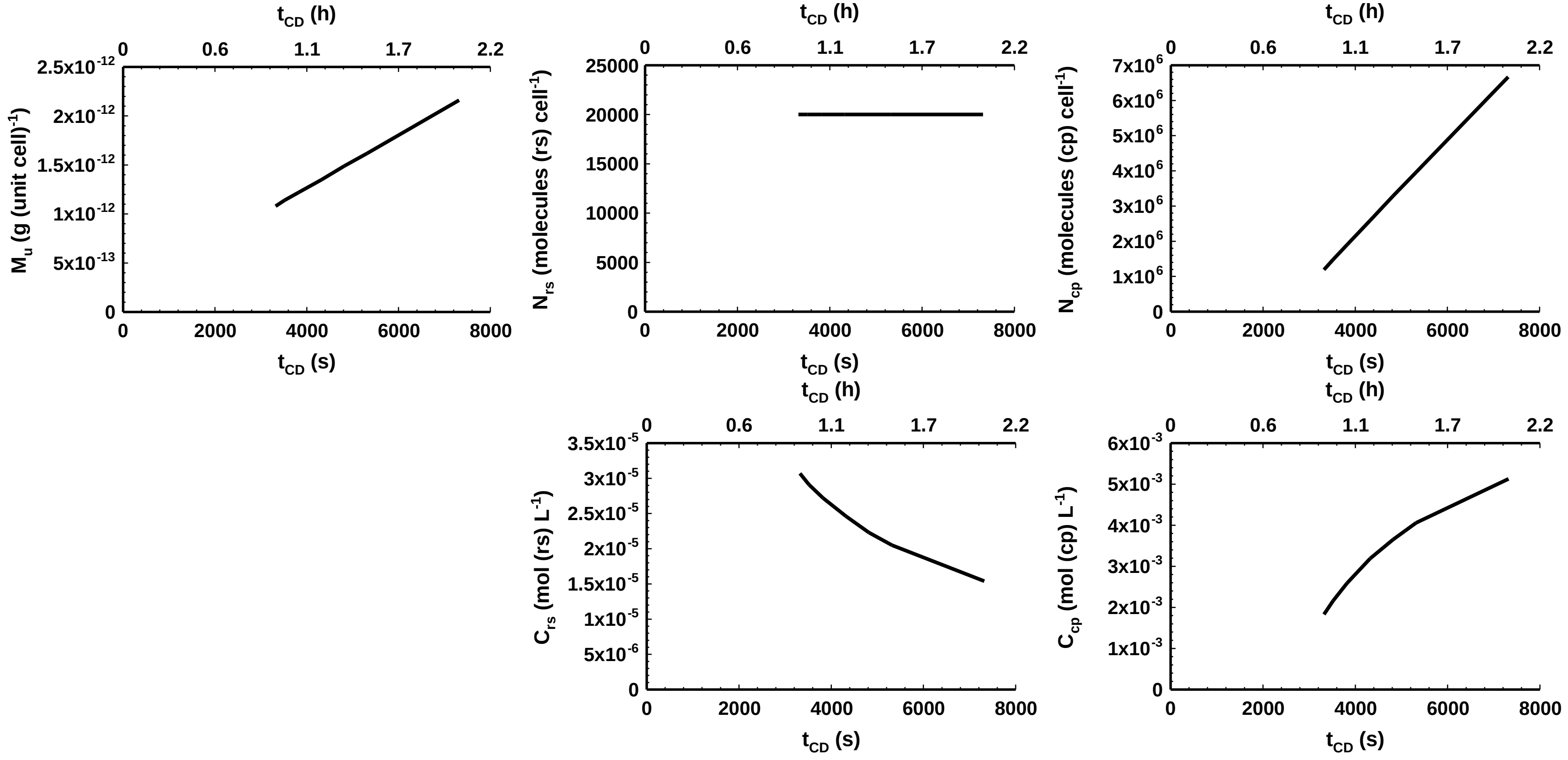

$$t_{CD} = \frac{\ldots + N_{cp} \cdot n_{cp} + N_{rs} \cdot n_{rpc} + \ldots}{\left(\ldots + N_{rs\_cp} + N_{rs\_rpc} + \ldots\right) \cdot k_{rs}}$$

$$M_u - M_{srs} \approx M_{cp}$$

$$M_u = \ldots + N_{cp} \cdot m_{cp} + N_{rs} \cdot \left(m_{rpc} + m_{rrna}\right) + \ldots$$

(c)

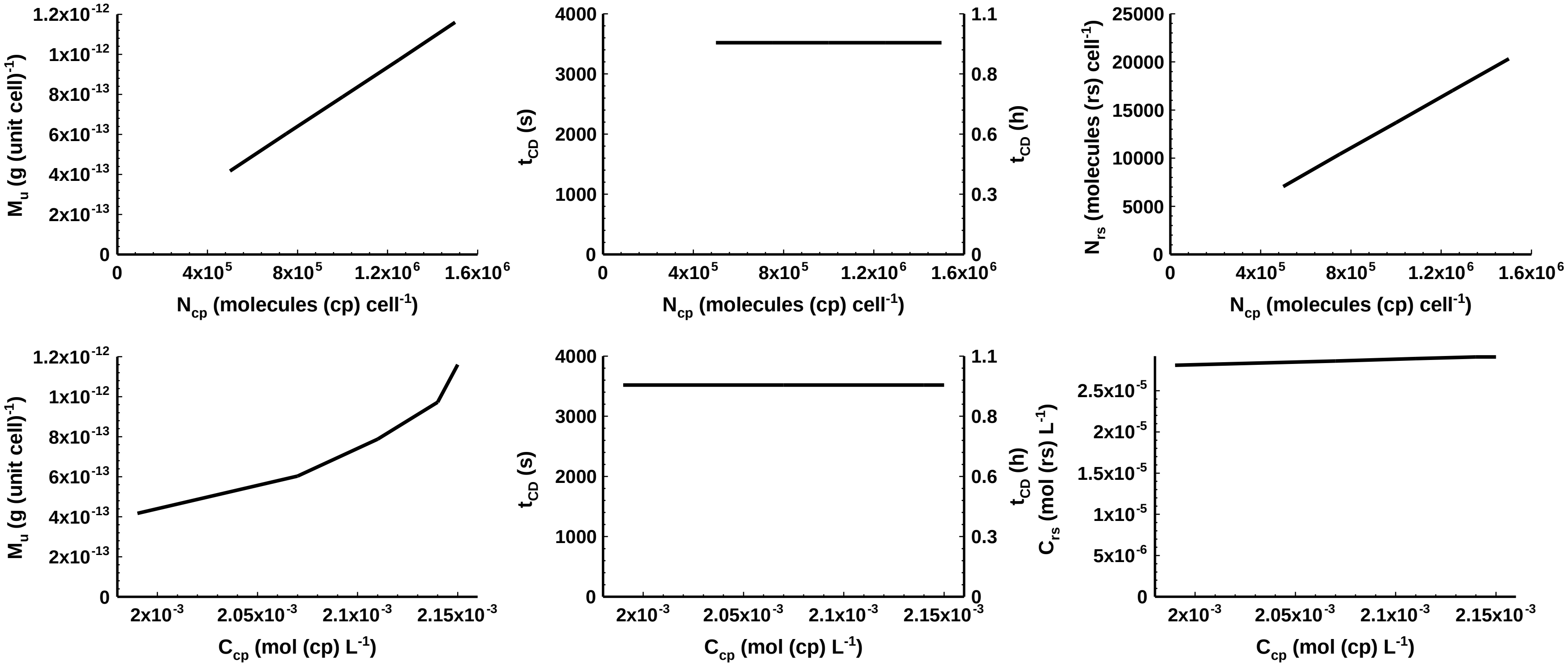

1.2x10-12
1x10-12
8x10-13
6x10-13
4x10-13
2x10-13
0
Mu (g (unit cell)-1)
0
4x105
8x105
1.2x106
1.6x106
Ncp (molecules (cp) cell-1)
4000
3000
2000
1000
tCD (s)
1.1
0.8
0.6
0.3
tCD (h)
25000
20000
15000
10000
5000
Nrs (molecules (rs) cell-1)
2x10-3
2.05x10-3
2.1x10-3
2.15x10-3
Ccp (mol (cp) L-1)
2.5x10-5
2x10-5
1.5x10-5
1x10-5
5x10-6
Crs (mol (rs) L-1)

(d)

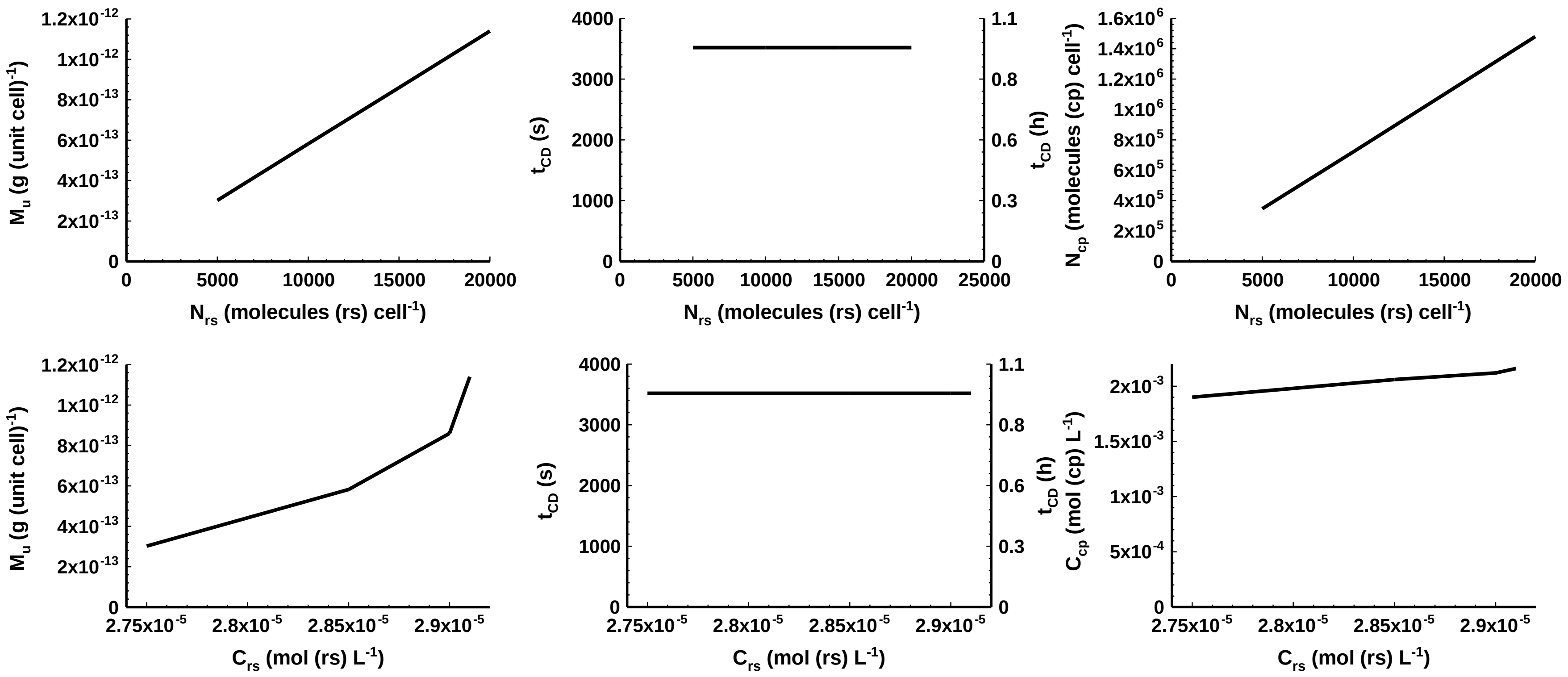

Supplementary Fig. 1. Simple schematic modes explaining connection between key parameters of models. Key parameters: cell cycle length of unit cell (t_{CD}, s and h), unit cell mass (M_u, g (unit cell)$^{-1}$), number of ribosomes in the cell (N_{rs}, molecules (rs) cell^{-1}), number of cushioning protein molecules in the cell (N_{cp}, molecules (cp) cell^{-1}), molar concentration of ribosomes (C_{rs}, mol (rs) L^{-1}), molar concentration of cushioning protein (C_{cp}, mol (cp) L^{-1}). t_{CD} is determined, for example, by the ratio of numbers of synthesized proteins (including N_{cp}) to N_{rs}. M_u is the sum of masses of cell components including ribosomes and cushioning protein. N_{cp} is determined by the difference of M_u and the size of the SRS (M_{srs}). N_{rs} is determined by the number of required proteins. (a) Relations between parameters if N_{cp} = const and t_{CD} is variable. (b) Relations between parameters if N_{rs} = const and t_{CD} is variable. (c) Relations between parameters if t_{CD} = const and N_{cp} is variable. (d) Relations between parameters if t_{CD} = const and N_{rs} is variable.

Supplementary Discussion 5.2: M_u, complexity of SRS and growth media

The calculated value of M_u of SSUCM-SRS-M was approximately 2 times larger than that of SSUCM-SRS-R because larger SRS was needed for the growth at minimal medium (Table 1, Supplementary Tables 13-14, section "Calculated unit cells based only on self-reproduction systems are problematic"). Also, the calculated value of $DNA\%_{mmc}$ was lower and enzymes of central and biosynthesis pathways comprised ($Enz\%_{mc}$) 21.5 % (g (tot enz) (g (dw cell))$^{-1}$).
At first view, these results seem obvious as cells growing on minimal medium needed biosynthesis pathways and more enzymes-polymerases to synthesize all biomass components from the generic substrate. Although systematic experimental studies of UCs have not been carried out by cultivating the cells at the same t_{CD} on different media, it is possible to calculate M_u values from available data[14] based on CHD theory (assuming M_u = const condition for the whole growth range and standard cell cycle parameter values of *E. coli*). The reanalysed data (Supplementary Table 1) indicated that the M_u values of cells growing on complex media were really approximately 20 to 60 % smaller than those of cells growing on mineral media which was in accordance with the calculated difference of 50 % the in case of M_u values of SSUCM-SRS-M and SSUCM-SRS-R (Table 1). Based on the results obtained, it seems that the M_u of cells is dependent on growth conditions (currently on the availability of different substrates).

Supplementary Table 1. Unit cell mass I. Unit cell mass (M_u, g (unit cell)$^{-1}$) values were calculated from experimentally determined cell parameter (specific growth rate of the cell culture (μ, h^{-1}), volume of the average cell in the cell culture (V_{tot_ave}, fL cell^{-1})) values of cells of *E. coli* grown on different mineral and rich media[14]. In addition, t_d is the cell cycle length (s and h), M_{tot_ave} is the mass of the average cell in the cell culture (g cell^{-1}) and M_{tot} is the cell mass (g cell^{-1}).

Growth condition	μ [1.1]	V_{tot_ave} [1.1]	t_d (s) [1.2]	t_d (h) [1.3]	M_{tot_ave} [1.4]	M_{tot} [1.5]	M_u [1.6]
**Complex medium**							
Lysogeny broth (LB)	1.61	4.4	1550	0.43	$4.4 \cdot 10^{-12}$	$3.12 \cdot 10^{-12}$	$1.35 \cdot 10^{-12}$
LB MG1655	1.62	3.9	1540	0.43	$3.9 \cdot 10^{-12}$	$2.77 \cdot 10^{-12}$	$1.19 \cdot 10^{-12}$
Glucose + amino acids	1.49	4.0	1675	0.47	$4 \cdot 10^{-12}$	$2.84 \cdot 10^{-12}$	$1.35 \cdot 10^{-12}$
Mannose + amino acids	1.28	4.1	1949	0.54	$4.1 \cdot 10^{-12}$	$2.91 \cdot 10^{-12}$	$1.74 \cdot 10^{-12}$
Glycerol + amino acids	1.26	3.9	1980	0.55	$3.9 \cdot 10^{-12}$	$2.77 \cdot 10^{-12}$	$1.69 \cdot 10^{-12}$
**Carbon sources**							
Acetate	0.29	2.4	8605	2.39	$2.4 \cdot 10^{-12}$	$1.70 \cdot 10^{-12}$	$2.70 \cdot 10^{-12}$
Fumarate	0.47	2.4	5309	1.47	$2.4 \cdot 10^{-12}$	$1.70 \cdot 10^{-12}$	$2.27 \cdot 10^{-12}$
Galactose	0.17	1.9	14678	4.08	$1.9 \cdot 10^{-12}$	$1.35 \cdot 10^{-12}$	$2.38 \cdot 10^{-12}$
Glucose	0.60	3.2	4159	1.16	$3.2 \cdot 10^{-12}$	$2.27 \cdot 10^{-12}$	$2.62 \cdot 10^{-12}$
Glucose MG1655	0.67	3.0	3724	1.03	$3 \cdot 10^{-12}$	$2.13 \cdot 10^{-12}$	$2.25 \cdot 10^{-12}$
Glucosamine	0.39	2.9	6398	1.78	$2.9 \cdot 10^{-12}$	$2.06 \cdot 10^{-12}$	$2.99 \cdot 10^{-12}$
Glycerol	0.47	2.3	5309	1.47	$2.3 \cdot 10^{-12}$	$1.63 \cdot 10^{-12}$	$2.18 \cdot 10^{-12}$
Pyruvate	0.40	2.1	6238	1.73	$2.1 \cdot 10^{-12}$	$1.49 \cdot 10^{-12}$	$2.14 \cdot 10^{-12}$
Succinate	0.49	2.4	5093	1.41	$2.4 \cdot 10^{-12}$	$1.70 \cdot 10^{-12}$	$2.22 \cdot 10^{-12}$
**Fixed μ on glucose**							
Chemostat μ = 0.5 h^{-1}	0.5	2.6	4991	1.39	$2.6 \cdot 10^{-12}$	$1.84 \cdot 10^{-12}$	$2.38 \cdot 10^{-12}$
Chemostat μ = 0.35 h^{-1}	0.35	2.4	7130	1.98	$2.4 \cdot 10^{-12}$	$1.70 \cdot 10^{-12}$	$2.56 \cdot 10^{-12}$
Chemostat μ = 0.2 h^{-1}	0.2	2.2	12477	3.47	$2.2 \cdot 10^{-12}$	$1.56 \cdot 10^{-12}$	$2.68 \cdot 10^{-12}$
Chemostat μ = 0.12 h^{-1}	0.12	2.1	20794	5.78	$2.1 \cdot 10^{-12}$	$1.49 \cdot 10^{-12}$	$2.73 \cdot 10^{-12}$

[1.1] Data of ref. [14] without standard deviation values.
[1.2] Calculated according to the Supplementary Eq. (115) of ref. [3] and using values of μ.
[1.3] The values of t_d (s) divided by the conversion factor 3600 (conversion between s and h).

[1.4] Calculated according to the following equation using values of *specific*, *precise* V_{tot_ave}, conversion factor (between fL and cm^3) and *generic* ρ_{tot} = 1 g (cell) (cm^3 (cell))$^{-1}$ from Supplementary Table 24 of ref. [3]:

$$M_{tot\_ave} = V_{tot\_ave} \cdot 10^{-12} \cdot \rho_{tot} \quad (1)$$

[1.5] Calculated from the following equation using values of previously calculated M_{tot_ave} and t_{a_ave} assuming that the relative average cell age t_{a_ave}/t_d = 0.41 based on the ideal age distribution for the balanced cell culture[15]:

$$M_{tot} = \frac{M_{tot\_ave}}{1 + \frac{t_{a\_ave}}{t_d}} \quad (2)$$

[1.6] Calculated according to the Eqs. (13)-(14), (16) of ref. [1] (the notations of C, D, τ, $M_{0(lin)}$ of ref. [1] correspond to t_C, t_D, t_d, M_{tot}/M_u in the current work respectively) and Supplementary Eq. (104) of ref. [3] using values of previously calculated (t_d, M_{tot}) and constant (*approximate* t_D, *specific, precise* n_{dna}, *approximate* k_{dp}; Supplementary Tables 4, 10 and 24 of ref. [3]) cell parameters.

Note that the calculations of Supplementary Table 1 were carried out assuming that the values of *approximate* k_{dp} (t_C) and *approximate* t_D were constant which might not be the case for real cells. However, there have been reports that the values of t_C and t_D for *E. coli* are very similar at least between certain medium types (based on LB and glucose) at optimal temperature (37 °C) whereas clear differences also exist depending on the medium and temperature[16]. More generalized view links the values of t_C and t_D to the value of t_d according to monotonous functions[17] but they do not explain, for example, the experimentally determined data mentioned in the previous sentence because very similar values of t_C and t_D were measured at very different values of t_d (1260 s = 0.4 h for LB and 3060 s = 0.9 h for glucose medium). On the other hand, some reports exclude the dependence between M_u and type of the media used[18], however, demonstrating differences between strains/species[19].
Another data-set of cells of different *E. coli* strains grown on various media[20] was similarly reanalysed (Supplementary Fig. 2). This time the data-set included also experimentally determined t_C and t_D values. The values of M_u of genetically modified *E. coli* strain grown on LB medium supplemented with antibiotics varied only 10 % and the values were similar to M_u values of wild-type strain that was also grown on complex media. The values of M_u of wild-type strain grown on minimal media were approximately 0 to 40 % higher than the values on complex media. It appears that in most cases the differences were below 10 % excluding certain extreme cases (very low values of M_u in case of LB growth and growth on acetate). Calculated values were generally smaller than those in Supplementary Table 1 because experimentally determined t_{CD} values were usually longer than the *approximate* t_{CD}. Note that the values of M_u are approximately 30 to 35 % higher than visualized in Fig. 3C of ref. [21] due to differences in calculation assumptions. Unit cell sizes were calculated as average cell size (corresponds to the cell with average cell age) divided by average OriC/cell in ref. [21] whereas M_u values were calculated as the ratio of cell mass and number of origins at the beginning of cell cycle.

Supplementary Table 2. Unit cell mass II. Unit cell mass (M_u, g (unit cell)$^{-1}$) values were calculated from experimentally determined cell parameter (cell cycle length (t_d, s and h), genome replication time (t_C, s and h), division time (t_D, s and h), volume of the average cell in the cell culture (V_{tot_ave}, μm^3 cell^{-1})) values of cells of different *E. coli* strains grown on mineral and rich media[20].

**Growth condition**	V_{tot_ave} [2.1]	t_d (s) [2.1]	t_C (s) [2.1]	t_D (s) [2.1]	t_d (h) [2.2]	t_C (h) [2.2]	t_D (h) [2.2]	M_u [2.3]
LB 5 ng/mL cTc	15.5	1686	4860	3120	0.47	1.35	0.87	$8.70\cdot10^{-13}$
LB 10 ng/mL cTc	9.4	1446	3780	2160	0.40	1.05	0.60	$7.88\cdot10^{-13}$
LB 20 ng/mL cTc	6.6	1332	2400	2220	0.37	0.67	0.62	$8.96\cdot10^{-13}$
LB 30 ng/mL cTc	5.3	1326	2160	2220	0.37	0.60	0.62	$7.97\cdot10^{-13}$
LB 50 ng/mL cTc	5.2	1242	1560	2400	0.35	0.43	0.67	$8.35\cdot10^{-13}$
LB broth	6.5	1278	2280	2160	0.36	0.63	0.60	$8.79\cdot10^{-13}$
RDM+glucose	6.6	1380	2460	2382	0.38	0.68	0.66	$8.73\cdot10^{-13}$
Glucose cAA	3.3	1950	2520	2112	0.54	0.70	0.59	$9.51\cdot10^{-13}$
Glucose	2.3	2520	2640	2220	0.70	0.73	0.62	$8.74\cdot10^{-13}$
Glycerol	1.7	3600	2880	2280	1.00	0.80	0.63	$9.44\cdot10^{-13}$
Acetate	1.7	5400	3480	3000	1.50	0.97	0.83	$1.09\cdot10^{-12}$
Mannose	1.4	5880	4080	2880	1.63	1.13	0.80	$9.02\cdot10^{-13}$

[2.1] Data of ref. [20] without standard deviation values. The values of t_d, t_C and t_D are multiplied by 60 (conversion of minutes to s).
[2.2] The values of parameters (s) divided by the conversion factor 3600 (conversion between s and h).
[2.3] Calculated according to Supplementary Eqs. (1)-(2) and Eqs. (13)-(14), (16) of ref. [1] (the notations of C, D, τ, $M_{0(lin)}$ of ref. [1] correspond to t_C, t_D, t_d, M_{tot}/M_u in the current work respectively) using experimentally determined values (V_{tot_ave}, t_d, t_C, t_D), *generic* ρ_{tot} = 1 g (cell) (cm^3 (cell))$^{-1}$ from Supplementary Table 24 of ref. [3] and assuming that the relative average cell age t_{a_ave}/t_d = 0.41 based on the ideal age distribution for the balanced cell culture[15].

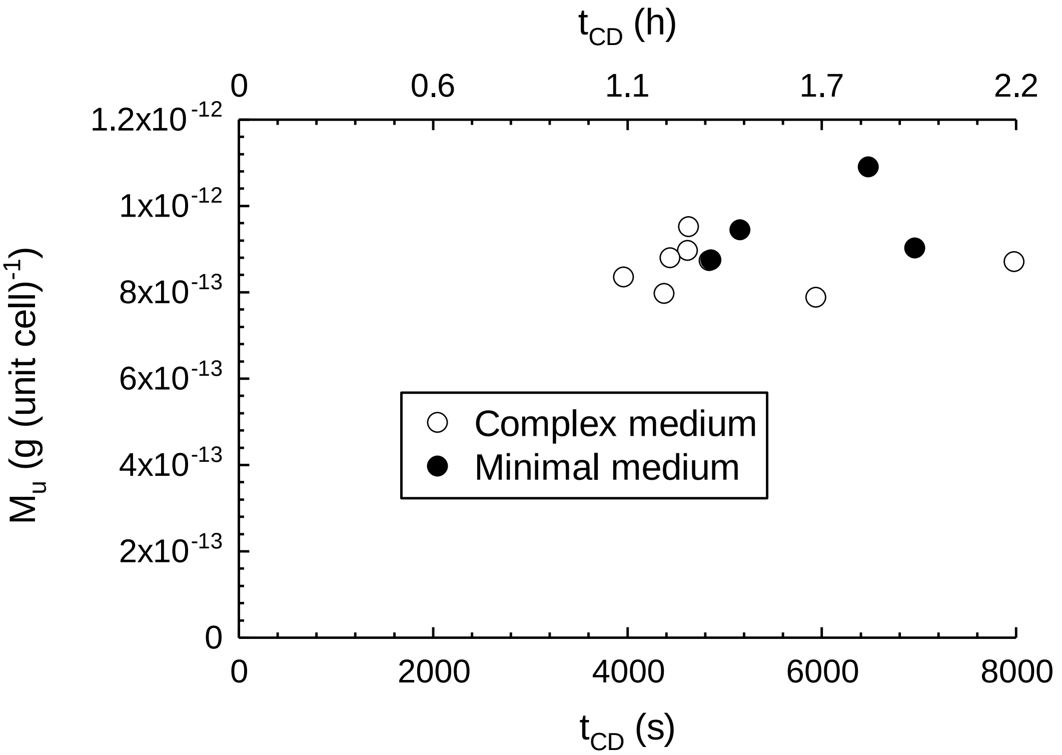

Supplementary Fig. 2. Sizes of unit cells. The dependence between values of calculated unit cell mass (M_u, g (unit cell)$^{-1}$) and experimentally determined cell cycle length of unit cell (t_{CD}, s and h) from data of ref. [20] (Supplementary Table 2).

The data-set of ref. [18] included experimentally determined values of average unit cell size. Calculated values of M_u were again higher than those of V_{u_ave} due to different cell age assumptions (Supplementary Fig. 3). It was not possible to draw any clear correlation between media type and unit cell size M_u values but in some cases the differences exceeded 50 % (Supplementary Fig. 4).

Supplementary Table 3. Unit cell mass III. Unit cell mass (M_u, g (unit cell)$^{-1}$) values were calculated from experimentally determined cell parameter (cell cycle length (t_d, s and h), genome replication time (t_C, s and h), division time (t_D, s and h), volume of the average cell in the cell culture (V_{tot_ave}, µm^3 cell^{-1})) values of cells of different *E. coli* strains grown on mineral and rich media[18]. In addition, V_{u_ave} is the unit cell volume of average cell in the cell culture (µm^3 cell^{-1}).

Growth condition	V_{u_ave} 3.1	V_{tot_ave} 3.1	t_C (s) 3.1	t_D (s) 3.1	t_d (s) 3.1	t_C (h) 3.2	t_D (h) 3.2	t_d (h) 3.2	M_u 3.3
**NCM3722 strain complex media**									
MOPS glucose synthetic rich	0.25	2.9	2495	2014	1278	0.69	0.56	0.36	$3.83\cdot10^{-13}$
	0.27	2.8	2554	2214	1375	0.71	0.62	0.38	$3.86\cdot10^{-13}$
	0.26	3.2	2385	2442	1329	0.66	0.68	0.37	$3.89\cdot10^{-13}$
	0.25	2.7	2572	2188	1380	0.71	0.61	0.38	$3.77\cdot10^{-13}$
TSB	0.34	3.8	2260	2084	1246	0.63	0.58	0.35	$5.11\cdot10^{-13}$
	0.26	2.9	2557	2210	1375	0.71	0.61	0.38	$3.88\cdot10^{-13}$
	0.36	3.7	1739	2245	1181	0.48	0.62	0.33	$5.33\cdot10^{-13}$
**NCM3722 strain semi-complex media**									
MOPS glucose + 12 a. a.	0.37	0.9	2648	1651	3320	0.74	0.46	0.92	$5.42\cdot10^{-13}$
	0.27	1.6	2537	1870	1711	0.70	0.52	0.48	$4.03\cdot10^{-13}$
	0.28	1.6	2017	1980	1593	0.56	0.55	0.44	$4.27\cdot10^{-13}$
MOPS glucose + 6 a. a.	0.17	0.9	2315	2307	1967	0.64	0.64	0.55	$2.50\cdot10^{-13}$
	0.25	1.3	2361	2323	1990	0.66	0.65	0.55	$3.68\cdot10^{-13}$
	0.35	1.3	2729	1240	2167	0.76	0.34	0.60	$5.18\cdot10^{-13}$
MOPS glucose + casamino acids	0.31	1.9	2519	1965	1743	0.70	0.55	0.48	$4.68\cdot10^{-13}$
**NCM3722 strain minimal media**									
MOPS glucose	0.31	1.0	1962	1976	2412	0.55	0.55	0.67	$4.68\cdot10^{-13}$
	0.36	0.9	2104	1830	2833	0.58	0.51	0.79	$5.36\cdot10^{-13}$
	0.32	1.0	2089	1802	2347	0.58	0.50	0.65	$4.88\cdot10^{-13}$
	0.33	1.0	2073	1547	2263	0.58	0.43	0.63	$4.91\cdot10^{-13}$
MOPS glycerol	0.21	0.5	2648	1636	3305	0.74	0.45	0.92	$3.13\cdot10^{-13}$
	0.26	0.6	3341	1517	4177	0.93	0.42	1.16	$3.84\cdot10^{-13}$
	0.23	0.5	2224	1632	3162	0.62	0.45	0.88	$3.38\cdot10^{-13}$
MOPS sorbitol	0.27	0.6	3526	1550	4383	0.98	0.43	1.22	$3.92\cdot10^{-13}$

	0.27	0.6	2648	2100	3803	0.74	0.58	1.06	$3.97\cdot10^{-13}$
	0.24	0.6	2648	1640	3309	0.74	0.46	0.92	$3.60\cdot10^{-13}$
	0.27	0.6	3141	1495	3974	0.87	0.42	1.10	$3.96\cdot10^{-13}$
**MG1655 strain complex media**									
MOPS glucose synthetic rich	0.38	2.9	2664	1523	1439	0.74	0.42	0.40	$5.54\cdot10^{-13}$
	0.48	2.8	2326	1269	1398	0.65	0.35	0.39	$7.19\cdot10^{-13}$
	0.56	3.3	2070	1528	1413	0.57	0.42	0.39	$8.44\cdot10^{-13}$
	0.52	3.7	2483	1787	1509	0.69	0.50	0.42	$7.60\cdot10^{-13}$
	0.52	3.5	2264	1632	1428	0.63	0.45	0.40	$7.81\cdot10^{-13}$
MOPS glycerol synthetic rich	0.45	2.7	2368	1914	1664	0.66	0.53	0.46	$6.75\cdot10^{-13}$
	0.39	2.7	2399	2234	1666	0.67	0.62	0.46	$5.87\cdot10^{-13}$
MOPS mannose synthetic rich	0.30	2.7	2864	2979	1866	0.80	0.83	0.52	$4.40\cdot10^{-13}$
**MG1655 strain semi-complex media**									
M9 glucose + 3 a. a.	0.33	0.9	2595	2539	3390	0.72	0.71	0.94	$4.99\cdot10^{-13}$
	0.53	1.3	3093	1337	3516	0.86	0.37	0.98	$7.82\cdot10^{-13}$
M9 glucose + 3 a. a. + 0.2mM uracil	0.39	1.1	2796	1785	3011	0.78	0.50	0.84	$5.80\cdot10^{-13}$
	0.51	1.1	2696	755	3149	0.75	0.21	0.87	$7.34\cdot10^{-13}$
MOPS glucose + 0.2mM uracil	0.49	1.2	3186	940	3312	0.89	0.26	0.92	$7.25\cdot10^{-13}$
	0.45	1.2	3108	1440	3298	0.86	0.40	0.92	$6.75\cdot10^{-13}$
MOPS glucose + 12 a. a.	0.45	1.3	2932	2003	3118	0.81	0.56	0.87	$6.73\cdot10^{-13}$
MOPS glucose + 12 a. a. + 0.2mM uracil	0.32	1.0	3602	2066	3464	1.00	0.57	0.96	$4.75\cdot10^{-13}$
MOPS glucose + 6 a. a.	0.42	1.4	3476	1716	2985	0.97	0.48	0.83	$6.32\cdot10^{-13}$
	0.35	1.3	2719	2926	2996	0.76	0.81	0.83	$5.17\cdot10^{-13}$
	0.45	1.6	2817	2645	3012	0.78	0.73	0.84	$6.63\cdot10^{-13}$
MOPS glucose + 6 a. a. + 0.2mM uracil	0.42	1.4	2146	1891	2342	0.60	0.53	0.65	$6.28\cdot10^{-13}$
	0.44	1.4	2489	1429	2404	0.69	0.40	0.67	$6.59\cdot10^{-13}$
MOPS glucose + casamino acids	0.38	1.5	3424	1035	2214	0.95	0.29	0.61	$5.42\cdot10^{-13}$
	0.34	1.2	2824	1327	2307	0.78	0.37	0.64	$5.10\cdot10^{-13}$
	0.48	1.6	3281	727	2346	0.91	0.20	0.65	$7.22\cdot10^{-13}$
MOPS glycerol + 0.2mM uracil	0.39	0.7	3988	1278	6040	1.11	0.35	1.68	$5.75\cdot10^{-13}$
	0.44	0.8	3933	2147	7337	1.09	0.60	2.04	$6.56\cdot10^{-13}$
**MG1655 strain minimal media**									

MOPS glucose	0.40	1.0	3106	1617	3517	0.86	0.45	0.98	$5.91 \cdot 10^{-13}$
	0.38	1.1	2892	2391	3604	0.80	0.66	1.00	$5.78 \cdot 10^{-13}$
	0.48	1.4	3427	2294	3732	0.95	0.64	1.04	$7.22 \cdot 10^{-13}$
MOPS glycerol	0.30	0.5	4565	891	6376	1.27	0.25	1.77	$4.35 \cdot 10^{-13}$
	0.32	0.7	3543	5230	7979	0.98	1.45	2.22	$4.60 \cdot 10^{-13}$
	0.33	0.8	6348	1249	5546	1.76	0.35	1.54	$4.88 \cdot 10^{-13}$

[3.1] Data points from different nutrient condition experiments of *E. coli* without inducers or drugs[18]. The values of t_d, t_C and t_D are multiplied by 60 (conversion of minutes to s).
[3.2] The values of parameters (s) divided by the conversion factor 3600 (conversion between s and h).
[3.3] Calculated according to Supplementary Eqs. (1)-(2) and Eqs. (13)-(14), (16) of ref. [1] (the notations of C, D, τ, $M_{0(lin)}$ of ref. [1] correspond to t_C, t_D, t_d, M_{tot}/M_u in the current work respectively) using values of V_{tot_ave}, t_d, t_{CD}, *generic* ρ_{tot} = 1 g (cell) (cm^3 (cell))$^{-1}$ from Supplementary Table 24 of ref. [3] and assuming that the relative average cell age t_{a_ave}/t_d = 0.41 based on the ideal age distribution for the balanced cell culture[15].

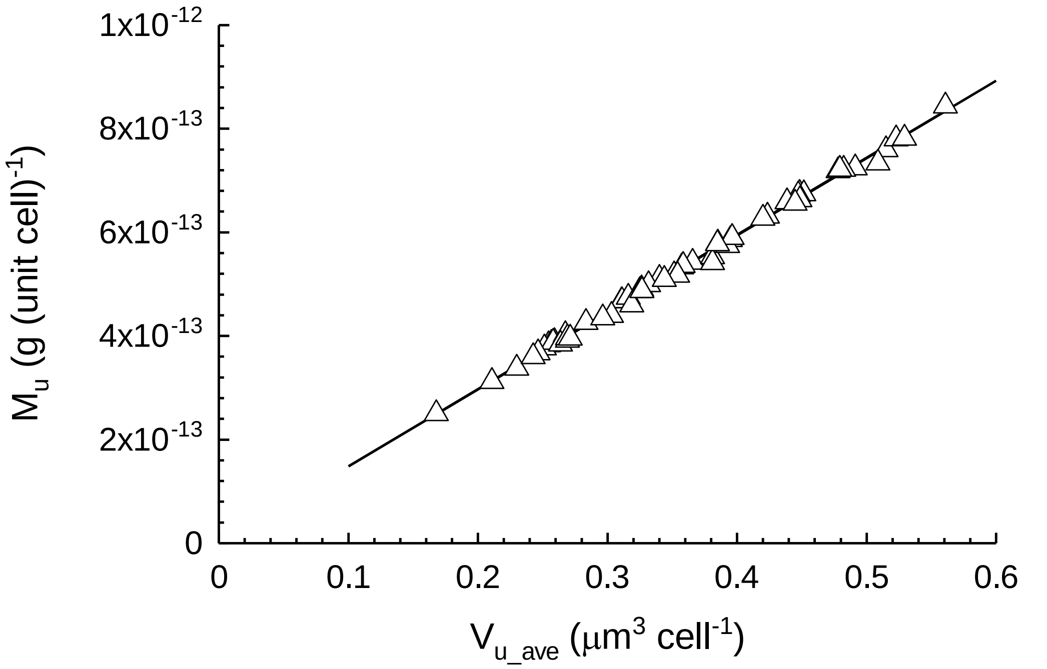

Supplementary Fig. 3. Sizes of unit cells. The dependence between values of calculated unit cell mass (M_u, g (unit cell)$^{-1}$) and experimentally determined unit cell volume of average cell in the cell culture (V_{u_ave}, μm^3 cell^{-1}) from data of ref. [18] (Supplementary Table 3). Linear regression line is included, R^2 = 0.997.

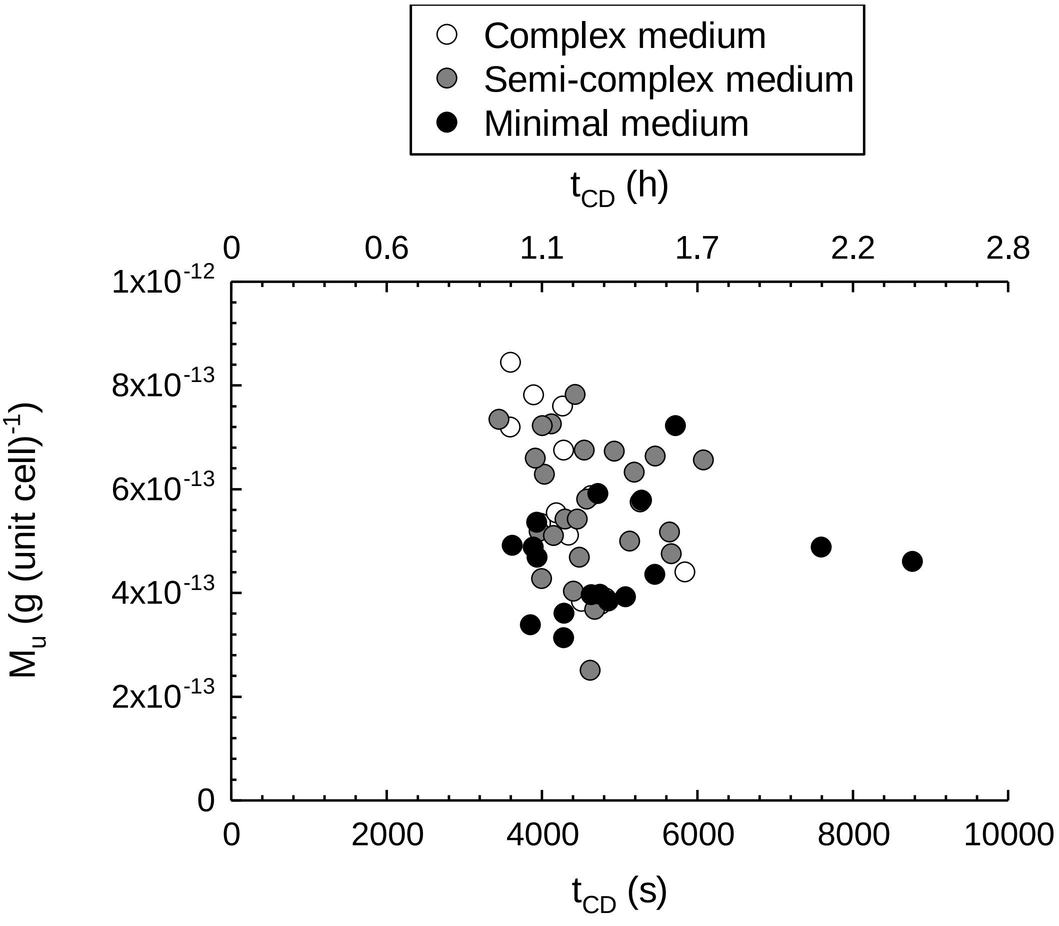

Supplementary Fig. 4. Sizes of unit cells. The dependence between values of calculated unit cell mass (M_u, g (unit cell)$^{-1}$) and experimentally determined cell cycle length of unit cell (t_{CD}, s and h) from data of ref. [18] (Supplementary Table 3).

Clear dependence between media types and M_u values in Supplementary Table 1 is most probably artificial effect of constant t_{CD} value taking into account missing dependencies in case of Supplementary Tables 2-3. There are opposite results confirming[22] or excluding[23-24] the constant M_u value for the whole growth range at the same medium. Neither it is possible to say whether M_u depends on genotype, physiological state or growth conditions. Even if M_u values of cells are sometimes smaller during growth on rich medium, it is not clear how the SRSs of cells are reorganized and whether the complexity of SRS is decreased as expected in the current work. However, the metabolic scaling analyses of prokaryotes have shown that cell size increases linearly with the size of the metabolic network and the number of different genes[25].

Supplementary Discussion 5.3: Minimal cell size

Experimental results show that typical M_u values for the *E. coli* are mainly in the range 10^{-13} g (unit cell)$^{-1}$ according to the literature[18,20]. However, it was not possible to state that considerably smaller calculated values of Table 1 were below minimal theoretical cell sizes. Theoretically, the smallest cell size is mostly determined by the catalytic efficiency of enzymes and protein synthetic machinery[26] as also shown in ref. [3]. According to the literature[27], the diameter of one of the smallest free-living bacterium *Mycoplasma genitalium* is about 300 nm which corresponds to approximate M_{tot} = 10^{-14} g cell^{-1}. M_u values calculated from models were slightly higher than that value but largely because of the roughly 9 times longer genome compared to the genome of *M. genitalium*. Although, the value of $DNA\%_{mmc}$ of *M. genitalium* is considerable[28], previously calculated $DNA\%_{mmc}$ values (Table 1) are several times higher.
Analysis of filtrated groundwater has revealed even smaller cells (average size 0.009 µm^3 cell^{-1}) from diverse less known bacterial domains[29]. Besides considerably short genomes (< 1 Mb), missing outer membrane, apparently slow growth mode (concluded from the average N_{rs} < 50 molecules (rs) cell^{-1} or $C_{rs} < 9 \cdot 10^{-6}$ mol (rs) L^{-1}) and being symbionts (they require ready-made building blocks), they probably use certain space optimization strategies as cytoplasmic compartment composes only half of the cell size and $DNA\%_{mmc}$ > 20 % (g (tot dna) (g (dw cell))$^{-1}$) of the cytoplasm. Similar N_{rs} values (92 molecules (rs) cell^{-1}) and cell sizes (0.009 to 0.04 µm^3 cell^{-1}) have been reported also for ultra-small archaeons[30]. More examples can be found in ref. [31].
In addition, metabolic scaling analysis based on various experimentally determined data (values of volumes of cellular compartments and macromolecules) showed that the minimal theoretical V_{tot} for Gram-positive bacteria would be approximately $1.02 \cdot 10^{-14}$ cm^3 cell^{-1}. Interestingly, the latter analysis indicated that the theoretical minimal cell is mostly filled by DNA[32] as in our calculations. Theoretical biophysical analysis[33] indicated that the critical range of cell diameter values for free-floating spherical microbial cells is 0.6 – 1.8 µm cell^{-1}. Below that range the ability of the cell to respond to environmental signals (temperature, chemical gradient, light) and to disperse becomes challenging. Based on that analysis, all previously reviewed ultra-small cells must be non-motile. On the contrary to current calculations, most of the experimentally observed or theoretically obtained values of M_{tot} and N_{rs} comparable to current calculations correspond to very low μ values. It was shown[25] that there is a positive correlation between cell size and μ. It has been suggested that small cells

indicate certain shutdown-states characterized by reduced levels of protein machinery and densely compacted genomes[26].

Supplementary Discussion 5.4: Cushioning parameter in unit cells

The calculations of the UCs composed of only SRS showed that their t_{CD} values in the case of the reasonable molecular configurations were between 900 s = 0.3 h (SSUCM-SRS-R) and 2500 s = 0.7 h (SSUCM-SRS-M) (Supplementary Tables 13-14). If the usual physiologically feasible t_d (3600 s = 1.0 h) of *E. coli* was targeted, the corresponding UCs were far too small and the composition unrealistic compared to cells of *E. coli* (section "Calculated unit cells based only on self-reproduction systems are problematic", Table 1).

The simplest solution for this difference mentioned in the section "Calculated unit cells based only on self-reproduction systems are problematic" (assuming that the model is not overwhelmingly wrong and the input parameter values are not completely underestimated (especially l_{PWi})) would be to include additional molecules (mass) of cell components which also increase the M_{srs} due to additional synthesis requirements. That would also increase M_u, N_{rs} and t_{CD} and decrease $DNA\%_{mmc}$. Let us define those additional cell components collectively as the CPrm of the cell. The identification of such cell components is potentially useful because it might be possible to replace them with those that are biotechnologically valuable without changing SRS. We introduced N_{aa_prot} of unspecified protein (similar to CP in the models of the current work) in the case of SSPCM-RS+AA+PROT in our earlier calculations. N_{aa_prot} could be also considered as a CPrm meeting the requirements presented here (Supplementary Discussion 5.3 of ref. [3]).

The term CPrm has a wider meaning involving cell components of CL for specific functions and unused SRS cell components. Some of the possible CL candidates for CPrm can be substrate transporters that do not correspond to the growth medium[34], certain regulatory proteins, stress mechanisms, non-optimal metabolic pathways, unneeded proteins[35], reserve materials (glycogen, polyhydroxy butyrate, lipid components of cell wall, polyphosphates), accumulating intracellular metabolites. Possible SRS candidates for CPrm might be unused enzymes from essential synthesis pathways, enzymes maintaining futile cycles on metabolic pathways, idle ribosomes[36] or other cell components prepared in excess, for example, to overcome possible sudden changes in growth conditions. It was shown previously (section "Ribosomal proteins—minimal core components of the SRS" of ref. [3] and Supplementary Discussion 5.1 of ref. [3]) that the value of t_{d_rs} increases if the k_{rs} value decreases (due to changes in environmental conditions or part of the ribosomes are not working due to limited resources ($F_{enz\_PW2\_r}$ is smaller than possible F_{rs}) for example). The dependence between t_{d_rs} and N_{rs} might be, however, different for different cases. If k_{rs} decreases (not influenced by N_{rs}) then t_{d_rs} increases but is still independent from N_{rs}. If $F_{enz\_PW2\_r}$ is less than possible F_{rs} (which is an exception of base assumptions of SSUCM framework) then effective ribosomes (analogical to effective RC (Supplementary Discussion 5.11.1 of ref. [3])) must be introduced and t_{d_rs} would increase and depend on N_{cell_comp}. The latter case is described by following Supplementary Eq. (3) which states that the number of effective ribosomes $N_{rse} < N_{rs}$.

$$t_{d\_rs} = \frac{N_{rs} \cdot n_{rpc}}{N_{rse} \cdot k_{rs}} \tag{3}$$

The corresponding t_{d_rs} increases linearly with the increase of N_{rs} to the infinity starting from the certain point where exists condition $N_{rse} = N_{rs}$ (t_{d_rs} has theoretical minimum). Below that N_{rs} value there is a familiar independency between t_{d_rs} and N_{rs} (described by Eq. (1) of ref. [3]). For example, if N_{rs} = 100 molecules (rs) cell^{-1} ($C_{rs} = 3.5 \cdot 10^{-4}$ mol (rs) L^{-1}) and N_{rse} = 20

molecules (rse) cell^{-1} (C_{rs} = 7.0·10^{-5} mol (rs) L^{-1}) then corresponding t_{d_rs} = 1810 s = 0.5 h. The theoretical minimum value of t_{d_rs} (362 s = 0.1 h) is achieved if N_{rs} = 20 molecules (rs) cell^{-1} (C_{rs} = 3.5·10^{-4} mol (rs) L^{-1}). The more serious the lack of amino acids, the smaller the value of N_{rs} where t_{d_rs} is minimal. Therefore, it would be reasonable for the proto-cell to diminish its size to grow faster. Mathematically it is also possible that the latter condition is achieved in the case of extremely small proto-cells (starving conditions). Certainly, low concentrations make the growth of such proto-cells very difficult due to possible fluctuations. Another issue is that there should be also functional ribosomes in the proto-cell. Assuming that the value of N_{rs} cannot be infinitely small, there exists some kind of minimal threshold value designated as N_{rs_min}. If there exists condition $N_{rs} > N_{rs\_min} > N_{rse}$ then the theoretical minimum of t_{d_rs} is not achieved at all. If N_{rs} = 1 molecules (rs) cell^{-1} (C_{rs} = 3.5·10^{-4} mol (rs) L^{-1}) and N_{rs_min} = 0.5 molecules (rs) cell^{-1} (C_{rs} = 1.7·10^{-4} mol (rs) L^{-1}) then corresponding t_{d_rs} = 724 s = 0.2 h and $N_{rse} = N_{rs\_min}$. It should be reminded that the growth range of proto-cell where $N_{rse} < N_{rs}$ is an exception of base assumptions of the SSUCM framework.
Let us now consider the situation with the varying availability of amino acids from the environment for the same proto-cell. It would mean that the value of N_{rse} changes if the $F_{enz\_PW2\_r}$ changes whereas the value of N_{rs} remains constant if $N_{rs} > N_{rse}$. It appears that the value of t_{d_rs} decreases smoothly and exponentially with the increase of N_{rse} (Supplementary Eq. (3)) until N_{rse} becomes equal to N_{rs}. Therefore, the maximum value of t_{d_rs} appears at an infinitely small N_{rse} value whereas the theoretical minimum value of t_{d_rs} is achieved if $N_{rs} = N_{rse}$. The subsequent increase of N_{rse} leads also to the increase of N_{rs} and therefore the proto-cell grows with a theoretical minimum value of t_{d_rs}. It should be stressed that the dependence of N_{rse} and t_{d_rs} is similar for all N_{rs} values with slight differences concerning N_{rs_min}. If there exists N_{rs_min} (for example 1 molecules (rs) cell^{-1}) then minimal theoretical t_{d_rs} is achieved only at $N_{rse} = N_{rs\_min}$ and/or further ($N_{rse} > N_{rs\_min}$). Again, the described example of varying availability of amino acids from the environment (varying N_{rse}) is actually an exception of base assumptions of the current modelling framework.
Let us assume now that besides RPC there are also enzymes that form the amino acid synthesis pathway PW_2 in the proto-cell as in SSPCM-RS+AA of ref. [3]. Again, the independence of t_{d_rs+PW2} on N_{rs} is valid only if all the base assumptions of the modelling framework are taken into account (Supplementary Eqs. (4)-(5)). If the size of the proto-cell does not correspond to the availability of growth substrate ($N_{rs} > N_{rse}$ and $N_{enz\_PW2\_r} > N_{enz\_PW2\_r\_e}$), then the t_{d_rs+PW2} is longer than the theoretical minimum and the value of t_{d_rs+PW2} increases linearly with the increase of N_{rs} and $N_{enz\_PW2\_r}$ (N_{rse} and $N_{enz\_PW2\_r\_e}$ are constant).

$$t_{d\_rs+PW2} = \frac{N_{rs} \cdot n_{rpc} + N_{enz\_PW2\_r} \cdot n_{enz} \cdot l_{PW2}}{N_{rse} \cdot k_{rs}} \tag{4}$$

$$N_{rse} \cdot k_{rs} = N_{enz\_PW2\_r\_e} \cdot k_{enz} \tag{5}$$

Also, t_{d_rs+PW2} is longer than the theoretical minimum and increases exponentially with the decrease of N_{rse} and $N_{enz\_PW2\_r\_e}$ (N_{rs} and $N_{enz\_PW2\_r}$ are constant). Note that the theoretical minimum of t_{d_rs+PW2} cannot be achieved below some kind of minimum size of the proto-cell. Assuming that this minimum is a single functional molecule, it is obvious that the amino acid synthesis enzyme ($N_{enz\_PW2\_r\_min}$) defines the minimum in the present proto-cell example (proto-cell has 5 ribosomes beside single enzyme taking into account the differences of *generic* k_{enz} and *approximate* k_{rs}) and a theoretical minimum of t_{d_rs+PW2} can be achieved only at $N_{enz\_PW2\_r\_min}$ and/or further ($N_{enz\_PW2\_r} > N_{enz\_PW2\_r\_min}$).
In conclusion, effective macromolecules are also necessary in case molecule numbers have minimal levels or they are integers (for example one molecule). Note that values of N_{cell_comp} calculated from the models in the current work are mostly non-integers and also below 1 in

the case of small cells which means that part of the total value of CPrm can be actually explained by effective molecules. It should be stressed that the dependence between t_{CD} and effective molecule numbers is similar to proto-cells involving cell components (lipids, DNA) which do not have fixed stoichiometry with ribosomes as shown in ref. [3].

The introduction of CPrm is the reason why the base assumption of (strictly) optimized metabolism of the current modelling framework (Supplementary Discussion 5.11.1 of ref. [3]) must be considered with possibilities of exceptions (for example CP in SSUCM-M and SSUCM-R). Generally speaking, the idea of a strictly optimized metabolism cannot be considered viable. We need to accept sub-optimality in a number of cases (in terms of the self-replication rate of cells). If we assume that cells are at a sub-optimal state then synthesis of additional cell components of CL, which at first view seems to be an ineffective wasting process, actually allows cells to increase their mass and concentrations of cell components consequently eliminating the risk of stochastic fluctuations due to low copy numbers[37], maintaining optimal macromolecule collision rates[38] and forming the catastrophe kit for the cells to be prepared for sudden changes in growth conditions where feast occurs only very seldom and passes fast[26]. However, as the issue of sub-optimality and its physiological consequences is a really complicated and large issue, we skip it here. Note that the mentioned base-assumption in the modelling framework has a different meaning than in widely used constraint-based metabolic network models. Optimized metabolism in latter models is associated with finding specific optimal flux patterns among others in heavily under-determined metabolic networks whereas in SSUCMs it means correspondence between fluxes and synthesis equipment. Examples of sub-optimal intracellular flux patterns (maximum yield problem) can be found in the literature[39-41] but they cannot be taken as direct evidence of CPrm.

However, the existence of CPrm in UC is indirectly demonstrated by calculations of theoretical t_{CDmin} (see section "Calculated unit cells based only on self-reproduction systems are problematic"). If the t_{CDmin} of the SSUCM-SRS-M was approximately 2500 s = 0.7 h, every ribosome should be busy for an additional 1100 s = 0.3 h to reach the t_{CD} = 3600 s = 1.0 h assuming that the value of N_{rs} is independent of t_{CD} (same N_{rs} value for t_{CDmin} and t_{CD}). Assuming that *approximate* k_{rs} = 20 molecules (aa) s^{-1} rs^{-1} (Supplementary Table 4 of ref. [3]) each ribosome should translate additionally $(t_{CD} - t_{CDmin}) \cdot F_{rs} = 2.2 \cdot 10^4$ peptide bonds (molecules (aa) $cell^{-1}$) or about 73 proteins of *generic* n_{enz} = 300 molecules (aa) enz^{-1} each. The cushioning mass in the UC would depend on N_{rs}.

Supplementary Discussion 5.5: Cell load and SRS of the cell

Self-reproduction is of utmost importance to the microorganisms, single-cell organisms. The growth rate in their competitive environments is a decisive factor for their survival. The subpart of the cells responsible for carrying out processes necessary for self-reproduction is designated in our SSUCMs as SRS. Ribosomes are the central components of SRS due to their self-reproducing ability. Their functioning is supported by the other components – enzymes, polymerases, RNAs etc.

The second important aspect is that cells are capable of carrying out special functions and differentiating – this means that part of the metabolic machinery of cells which is not directly engaged in self-reproduction, is engaged in these complex coordinated activities – these parts of the metabolic machinery of cells are designated as CL in the SSUCMs. CL is consisting also of proteins, RNAs etc. but it does not contain cell components needed "directly" for carrying out self-reproduction. In addition to secondary functions, cell components of CL are associated with CPrms of the cell and perform as cushioning mass in the cell.

The simplest way is to divide cell components qualitatively into SRS and CL by their species based on their role in the cell (for example ribosomes belong to SRS as ribosomes carry out translation). However, a precise quantitative approach is more complicated because the same cell component forms different sub-populations (in terms of their N_{cell_comp} or synthesis potential) considering different cell processes (for example ribosomes that polymerize themselves, polymerases or CP) which is the basis for more precise division according to which the same cell component might belong to both groups, SRS and CL. Therefore, the self-replication of SRS and the self-replication of cells must be considered separately.

The simplest cases are cell components that do not have roles in self-replication (CP and corresponding mRNA in SSUCMs)[35-36,42]. This category is analogical to non-essential genes/proteins of the cell in a given growth environment[43]. The extraction of sub-fractions of synthesis equipment (sub-fractions of ribosomes, nucleotide and amino acid synthesis enzymes, ETCs etc) is more difficult. Ribosomes that are synthesizing only CP are not strictly required for self-reproduction and according to the definition, they should be CL. RP complexes that are synthesizing mRNAs of CP are also not required for self-replication. This category can be extended also to the sub-population of tRNAs involved in CP synthesis. Similarly, the sub-population of amino acid synthesis enzymes that provide amino acids to the previously mentioned ribosome sub-population is not strictly needed for self-reproduction. The same can be said for the sub-population of ribonucleotide synthesis enzymes that provide ribonucleotides for mRNAs of CP. Sub-populations of ETC and substrate transporters providing energy and material for all aforementioned cell components are also not directly necessary for self-reproduction. In conclusion, almost all cell components are somehow related to CP after the decomposition of cell components into sub-populations. On the other hand, the division of lipids (lipids covering only SRS and lipids covering the whole cell) is already quite ambiguous and difficult to imagine.

Regardless, only part of the simplest part of CL (CP without their mRNA) is presented in graphs, calculations and analysis of the current work as it is the most intuitive example for the reader. Also, this is why we are analysing simplified models of the cells with the daisy-like structure of the metabolic network consisting of independent linear reaction chains responsible each for the synthesis of an individual biopolymer, and a central metabolic pathway that provides key metabolites, building blocks for the synthesis pathways[3]. Dissecting the metabolism into SRS and CL is more intuitive and simpler in these models but, as already explained above, not entirely.

Supplementary Discussion 5.6: M_{cp}, complexity of SRS and growth media

Figure 2 describes the dependencies between most important cell design parameters: N_{rs}, N_{cp} and M_u. It must be stressed that if molar concentration units are used instead of molecule numbers, then simple linear relations are transformed into more complex non-linear relations (Supplementary Fig. 5). Whereas the dependence between C_{rs} and C_{cp} remains linear, other dependencies become exponential.

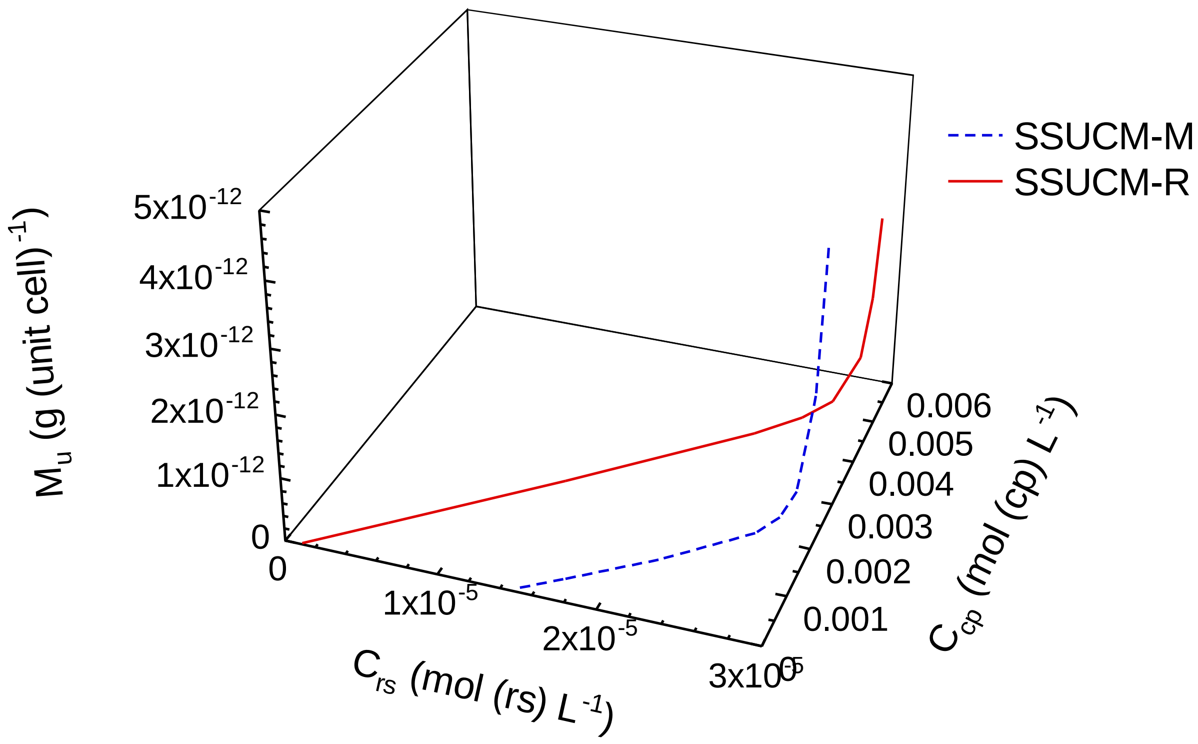

Supplementary Fig. 5. Cushioning protein synthesis in unit cells (UCs). The relationship between the molar concentration of cushioning protein (C_{cp}, mol (cp) L^{-1}), the molar concentration of ribosomes (C_{rs}, mol (rs) L^{-1}) and unit cell mass (M_u, g (unit cell)$^{-1}$) of UCs growing on rich (solid line) and minimal medium (dashed line) if the cell cycle length of the unit cell t_{CD} is equal to *approximate* 3520 s = 1.0 h.

Indirect evidence for cushioning mass in UC can be also found from the reanalysis of data of *E. coli* grown on different mineral and rich media[14]. Previously estimated M_u values (Supplementary Table 1) were used as input data for SSUCM-R and SSUCM-M. The values of M_{cp} were calculated from the models and the results indicated that, although, UCs on rich media were smaller, they, nevertheless, contained in most cases more CPs (up to 90 % more) (Supplementary Fig. 6). The values of M_{cp} decreased characteristically linearly (as in Fig. 2) with the decrease of M_u because *approximate* t_{CD} was used for all data-points. It also appeared that the value of M_{cp} in UC increased approximately 15 % in chemostat cultures towards slower growth. Remarkably, similar change happened also in case of M_u values which means that the value of $CP\%_{mc}$ remained to very narrow range of 26.3 – 26.5 % (g (tot cp) (g (dw cell))$^{-1}$). It means that the data of ref. [14] does not support the assumption of constant M_u from original CHD theory but, instead, it provides evidence of constant $CP\%_{mc}$.
There were no clear linear dependencies between M_{cp} and M_u values calculated from the data of ref. [20] due to the variability of t_{CD}. It even seems that M_{cp} is independent of M_u. Still, the division between complex and minimal media was visible (Supplementary Fig. 7). Generally, the values of M_{cp} were higher for cells growing on complex media although respective UCs had the same size or were even smaller.
The data-set of ref. [18] shows clear differences between used *E. coli* strains and also between media types (Supplementary Fig. 8). UCs of K12 MG1655 are bigger and cells growing on complex media contain more CP. Compared to the data of ref. [20], there were no clear dependencies between M_{cp} and M_u values during growth on minimal media.

In conclusion, there should be a positive linear correlation between M_{cp} and M_u based on experimentally determined cell size data in case of t_{CD} = const. However, dependencies are much more complicated in case of varying t_{CD}. There are clear differences between used strains and it seems that generally cells have more cushioning mass during growth on rich media whereas M_u does not depend on growth media. These observations can be explained by similar range of M_u values for various growth media types. If smaller SRS for cells growing on complex media is expected, then CP should occupy the remaining mass.
However, it must be stressed that the correspondence between models and actual SRSs of real cells in those experiments might be insufficient and difficult to estimate. Considerable variation of complexities of SRSs between different strains and media (especially semi-complex media) can not be excluded.

Supplementary Table 4. Cushioning mass. Unit cell mass (M_u, g (unit cell)$^{-1}$) and total mass of cushioning protein molecules in the cell (M_{cp}, g (tot cp) cell^{-1}) values were calculated from experimentally determined cell parameter values of cells of different *E. coli* strains grown on various mineral and rich media[14,18,20].

**Growth condition**	M_u [4.1]	M_{cp}
**Complex medium [4.2]**		
Lysogeny broth (LB)	$1.35\cdot10^{-12}$	$2.46\cdot10^{-13}$
LB MG1655	$1.19\cdot10^{-12}$	$2.16\cdot10^{-13}$
Glucose + amino acids	$1.35\cdot10^{-12}$	$2.46\cdot10^{-13}$
Mannose + amino acids	$1.74\cdot10^{-12}$	$3.19\cdot10^{-13}$
Glycerol + amino acids	$1.69\cdot10^{-12}$	$3.10\cdot10^{-13}$
**Carbon sources [4.3]**		
Acetate	$2.70\cdot10^{-12}$	$2.14\cdot10^{-13}$
Fumarate	$2.27\cdot10^{-12}$	$1.79\cdot10^{-13}$
Galactose	$2.38\cdot10^{-12}$	$1.88\cdot10^{-13}$
Glucose	$2.62\cdot10^{-12}$	$2.08\cdot10^{-13}$
Glucose MG1655	$2.25\cdot10^{-12}$	$1.78\cdot10^{-13}$
Glucosamine	$2.99\cdot10^{-12}$	$2.38\cdot10^{-13}$
Glycerol	$2.18\cdot10^{-12}$	$1.72\cdot10^{-13}$
Pyruvate	$2.14\cdot10^{-12}$	$1.69\cdot10^{-13}$
Succinate	$2.22\cdot10^{-12}$	$1.75\cdot10^{-13}$
**Fixed μ on glucose [4.3]**		
Chemostat μ = 0.5 h^{-1}	$2.38\cdot10^{-12}$	$1.88\cdot10^{-13}$
Chemostat μ = 0.35 h^{-1}	$2.56\cdot10^{-12}$	$2.03\cdot10^{-13}$
Chemostat μ = 0.2 h^{-1}	$2.68\cdot10^{-12}$	$2.13\cdot10^{-13}$
Chemostat μ = 0.12 h^{-1}	$2.73\cdot10^{-12}$	$2.17\cdot10^{-13}$
**Complex medium [4.4]**		
LB 5 ng/mL cTc	$8.70\cdot10^{-13}$	$2.03\cdot10^{-13}$
LB 10 ng/mL cTc	$7.88\cdot10^{-13}$	$1.71\cdot10^{-13}$
LB 20 ng/mL cTc	$8.96\cdot10^{-13}$	$1.81\cdot10^{-13}$
LB 30 ng/mL cTc	$7.97\cdot10^{-13}$	$1.57\cdot10^{-13}$
LB 50 ng/mL cTc	$8.35\cdot10^{-13}$	$1.59\cdot10^{-13}$
LB broth	$8.79\cdot10^{-13}$	$1.75\cdot10^{-13}$
RDM+glucose	$8.73\cdot10^{-13}$	$1.79\cdot10^{-13}$
Glucose cAA	$9.51\cdot10^{-13}$	$1.93\cdot10^{-13}$
**Carbon sources [4.5]**		
Glucose	$8.74\cdot10^{-13}$	$1.13\cdot10^{-13}$

Glycerol	$9.44 \cdot 10^{-13}$	$1.30 \cdot 10^{-13}$
Acetate	$1.09 \cdot 10^{-12}$	$1.82 \cdot 10^{-13}$
Mannose	$9.02 \cdot 10^{-13}$	$1.56 \cdot 10^{-13}$
**NCM3722 strain complex media** [4.6]		
MOPS glucose synthetic rich	$3.83 \cdot 10^{-13}$	$7.37 \cdot 10^{-14}$
	$3.86 \cdot 10^{-13}$	$7.57 \cdot 10^{-14}$
	$3.89 \cdot 10^{-13}$	$7.68 \cdot 10^{-14}$
	$3.77 \cdot 10^{-13}$	$7.39 \cdot 10^{-14}$
TSB	$5.11 \cdot 10^{-13}$	$9.86 \cdot 10^{-14}$
	$3.88 \cdot 10^{-13}$	$7.61 \cdot 10^{-14}$
	$5.34 \cdot 10^{-13}$	$9.97 \cdot 10^{-14}$
**NCM3722 strain semi-complex media** [4.6]		
MOPS glucose + 12 a. a.	$5.42 \cdot 10^{-13}$	$1.05 \cdot 10^{-13}$
	$4.03 \cdot 10^{-13}$	$7.70 \cdot 10^{-14}$
	$4.27 \cdot 10^{-13}$	$7.88 \cdot 10^{-14}$
MOPS glucose + 6 a. a.	$2.50 \cdot 10^{-13}$	$4.69 \cdot 10^{-14}$
	$3.68 \cdot 10^{-13}$	$7.15 \cdot 10^{-14}$
	$5.18 \cdot 10^{-13}$	$9.65 \cdot 10^{-14}$
MOPS glucose + casamino acids	$4.68 \cdot 10^{-13}$	$9.09 \cdot 10^{-14}$
**NCM3722 strain minimal media** [4.7]		
MOPS glucose	$4.68 \cdot 10^{-13}$	$4.29 \cdot 10^{-14}$
	$5.36 \cdot 10^{-13}$	$4.97 \cdot 10^{-14}$
	$4.88 \cdot 10^{-13}$	$4.40 \cdot 10^{-14}$
	$4.91 \cdot 10^{-13}$	$3.80 \cdot 10^{-14}$
MOPS glycerol	$3.13 \cdot 10^{-13}$	$3.17 \cdot 10^{-14}$
	$3.84 \cdot 10^{-13}$	$4.70 \cdot 10^{-14}$
	$3.38 \cdot 10^{-13}$	$2.87 \cdot 10^{-14}$
MOPS sorbitol	$3.92 \cdot 10^{-13}$	$5.03 \cdot 10^{-14}$
	$3.97 \cdot 10^{-13}$	$4.73 \cdot 10^{-14}$
	$3.60 \cdot 10^{-13}$	$3.71 \cdot 10^{-14}$
	$3.96 \cdot 10^{-13}$	$4.58 \cdot 10^{-14}$
**MG1655 strain complex media** [4.6]		
MOPS glucose synthetic rich	$5.54 \cdot 10^{-13}$	$1.06 \cdot 10^{-13}$
	$7.19 \cdot 10^{-13}$	$1.30 \cdot 10^{-13}$
	$8.44 \cdot 10^{-13}$	$1.53 \cdot 10^{-13}$
	$7.60 \cdot 10^{-13}$	$1.48 \cdot 10^{-13}$
	$7.81 \cdot 10^{-13}$	$1.47 \cdot 10^{-13}$
MOPS glycerol synthetic rich	$6.75 \cdot 10^{-13}$	$1.31 \cdot 10^{-13}$
	$5.87 \cdot 10^{-13}$	$1.17 \cdot 10^{-13}$
MOPS mannose synthetic rich	$4.40 \cdot 10^{-13}$	$9.27 \cdot 10^{-14}$
**MG1655 strain semi-complex media** [4.6]		
M9 glucose + 3 a. a.	$4.99 \cdot 10^{-13}$	$1.02 \cdot 10^{-13}$
	$7.82 \cdot 10^{-13}$	$1.55 \cdot 10^{-13}$
M9 glucose + 3 a. a. + 0.2mM uracil	$5.80 \cdot 10^{-13}$	$1.15 \cdot 10^{-13}$
	$7.34 \cdot 10^{-13}$	$1.30 \cdot 10^{-13}$
MOPS glucose + 0.2mM uracil	$7.25 \cdot 10^{-13}$	$1.39 \cdot 10^{-13}$
	$6.75 \cdot 10^{-13}$	$1.34 \cdot 10^{-13}$
MOPS glucose + 12 a. a.	$6.73 \cdot 10^{-13}$	$1.38 \cdot 10^{-13}$
MOPS glucose + 12 a. a. + 0.2mM uracil	$4.75 \cdot 10^{-13}$	$9.96 \cdot 10^{-14}$
MOPS glucose + 6 a. a.	$6.32 \cdot 10^{-13}$	$1.31 \cdot 10^{-13}$

	$5.17 \cdot 10^{-13}$	$1.09 \cdot 10^{-13}$
	$6.63 \cdot 10^{-13}$	$1.40 \cdot 10^{-13}$
MOPS glucose + 6 a. a. + 0.2mM uracil	$6.28 \cdot 10^{-13}$	$1.19 \cdot 10^{-13}$
	$6.59 \cdot 10^{-13}$	$1.23 \cdot 10^{-13}$
MOPS glucose + casamino acids	$5.42 \cdot 10^{-13}$	$1.06 \cdot 10^{-13}$
	$5.10 \cdot 10^{-13}$	$9.66 \cdot 10^{-14}$
	$7.22 \cdot 10^{-13}$	$1.37 \cdot 10^{-13}$
MOPS glycerol + 0.2mM uracil	$5.75 \cdot 10^{-13}$	$1.19 \cdot 10^{-13}$
	$6.56 \cdot 10^{-13}$	$1.43 \cdot 10^{-13}$
**MG1655 strain minimal media [4.7]**		
MOPS glucose	$5.91 \cdot 10^{-13}$	$7.21 \cdot 10^{-14}$
	$5.78 \cdot 10^{-13}$	$7.95 \cdot 10^{-14}$
	$7.22 \cdot 10^{-13}$	$1.08 \cdot 10^{-13}$
MOPS glycerol	$4.35 \cdot 10^{-13}$	$6.04 \cdot 10^{-14}$
	$4.60 \cdot 10^{-13}$	$8.68 \cdot 10^{-14}$
	$4.88 \cdot 10^{-13}$	$8.61 \cdot 10^{-14}$

[4.1] Taken from Supplementary Tables 1-3, respectively.

[4.2] Values of N_{cp} were calculated from SSUCM-R using values of previously calculated M_u in addition to standard values of cell parameters[3]. The values of M_{cp} were calculated from Supplementary Eq. (39).

[4.3] Values of N_{cp} were calculated from SSUCM-M using values of previously calculated M_u in addition to standard values of cell parameters[3]. The values of M_{cp} were calculated from Supplementary Eq. (39).

[4.4] Values of N_{cp} were calculated from SSUCM-R using values of previously calculated M_u, experimentally determined t_C and t_D (Supplementary Table 2) in addition to standard values of cell parameters[3]. The values of M_{cp} were calculated from Supplementary Eq. (39).

[4.5] Values of N_{cp} were calculated from SSUCM-M using values of previously calculated M_u, experimentally determined t_C and t_D (Supplementary Table 2) in addition to standard values of cell parameters[3]. The values of M_{cp} were calculated from Supplementary Eq. (39).

[4.6] Values of N_{cp} were calculated from SSUCM-R using values of previously calculated M_u, experimentally determined t_C and t_D (Supplementary Table 3) in addition to standard values of cell parameters[3]. The values of M_{cp} were calculated from Supplementary Eq. (39).

[4.7] Values of N_{cp} were calculated from SSUCM-M using values of previously calculated M_u, experimentally determined t_C and t_D (Supplementary Table 3) in addition to standard values of cell parameters[3]. The values of M_{cp} were calculated from Supplementary Eq. (39).

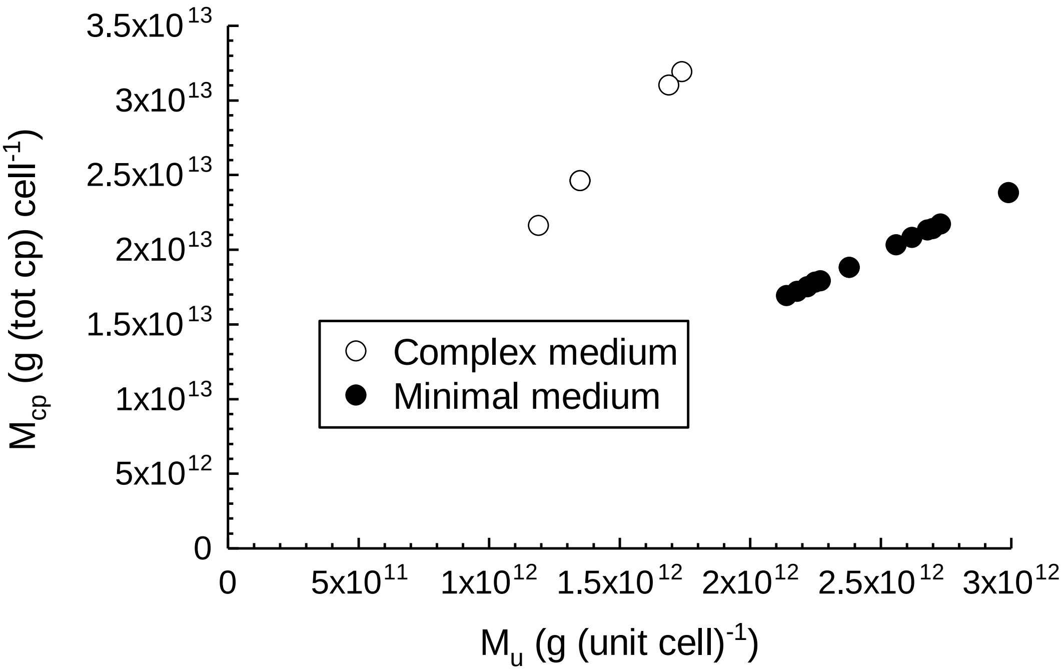

Supplementary Fig. 6. Cushioning mass of unit cells. The dependence between values of calculated unit cell mass (M_u, g (unit cell)$^{-1}$) and calculated total mass of cushioning protein molecules in the cell (M_{cp}, g (tot cp) cell^{-1}) from data of ref. [14] using SSUCM-M and SSUCM-R models (Supplementary Table 4).

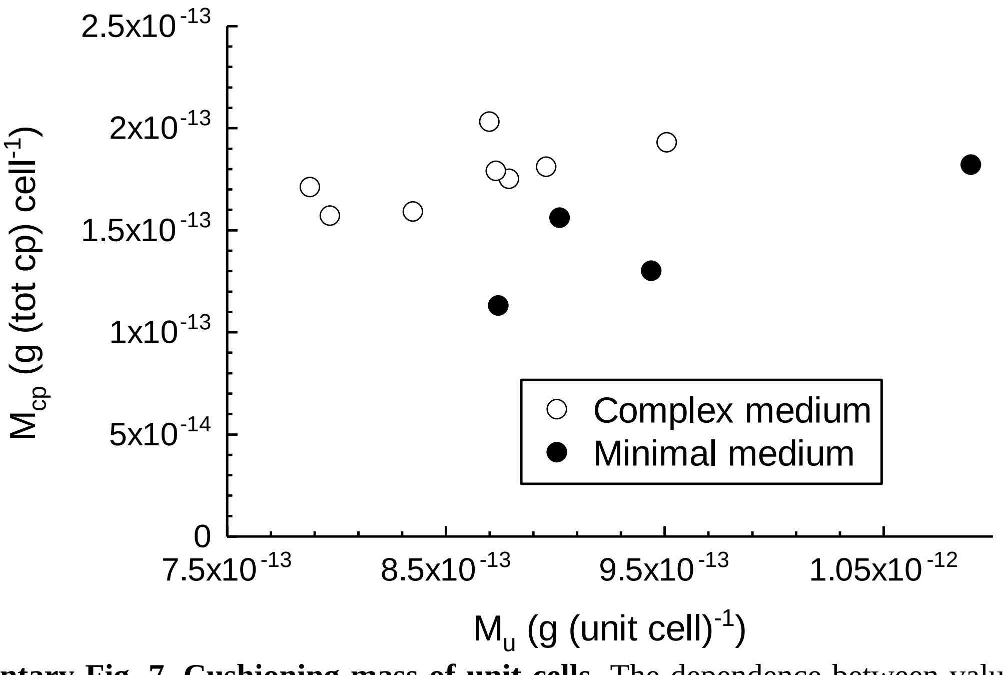

Supplementary Fig. 7. Cushioning mass of unit cells. The dependence between values of calculated unit cell mass (M_u, g (unit cell)$^{-1}$) and calculated total mass of cushioning protein molecules in the cell (M_{cp}, g (tot cp) cell^{-1}) from data of ref. [20] using SSUCM-M and SSUCM-R models (Supplementary Table 4).

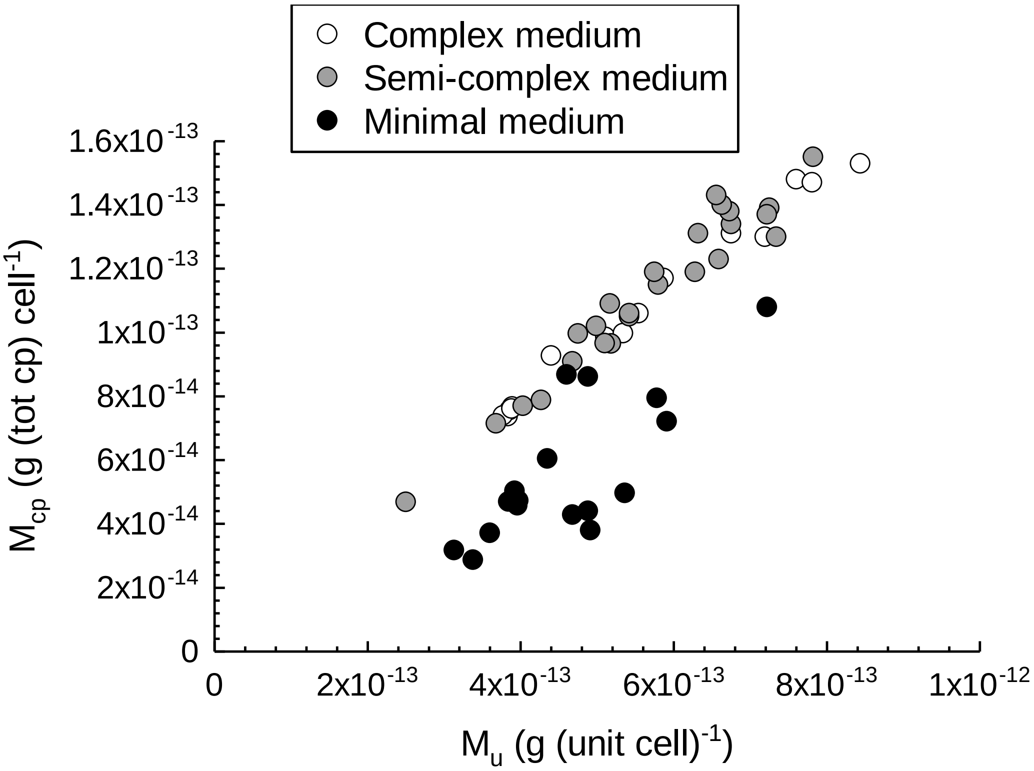

Supplementary Fig. 8. Cushioning mass of unit cells. The dependence between values of calculated unit cell mass (M_u, g (unit cell)$^{-1}$) and calculated total mass of cushioning protein molecules in the cell (M_{cp}, g (tot cp) cell^{-1}) from data of ref. [18] using SSUCM-M and SSUCM-R models (Supplementary Table 4).

Supplementary Discussion 5.7: Design parameters of the unit cell

Currently, it is shown that SSUCMs enable to specifically calculate the values of corresponding UC parameters including M_u (Supplementary Tables 5-8, section "Cushioning protein ensures physiologically reasonable cells") characterised briefly below (a longer overview will be provided in the future). If the lines in Fig. 2 are cut by a plane that is parallel to the x and y axis, then the cutting points characterise cases that have equal M_u values for both models. Equal M_u values mean that also other global cell dimensions (H_{cyl}, R_{tot}, S_{tot}, V_{tot}) are equal for both models. UC condition means that almost all DNA related parameter (N_{dna}, N_{rce}, $N_{enz\_PW3\_r}$, N_{mrna_rc}, N_{rs_rc}, F_{rce}, $DNA\%_{mmc}$) values are also equal for both models at the same t_{CD} value. The list of similarities between models is even longer considering substrate consumption (comparable N_{stp} values) and polymerization (N_{rp}, N_{rs}, N_{trna}). The differences between the compositions of UCs of both models are modest: the most important constituents are proteins that form the majority in the UC, $RNA\%_{mmc}$ is mainly determined by the value of N_{rs} because the majority of the RNA fraction comes from rRNA, the values of $DNA\%_{mmc}$ and $LIP\%_{mmc}$ are relatively low.

The latter similarities between model parameter values do not contradict the fact that SSUCM-M and SSUCM-R have very different SRSs. The size and complexity of the SRS are smaller in the case of SSUCM-R (no synthesis pathways and enzymes) which means that it can synthesize more CP. As CP and enzymes of synthesis pathways all belong to protein fraction, it is understandable why F_{stp}, F_{rs} or $PROT\%_{mmc}$ values are very similar. It is also clear that the compositions of protein fraction must be completely different between models. Enzymes of synthesis pathways formed 37.4 % (g (tot enz) (g (tot prot))$^{-1}$) ($Enz\%_{fc}$) and CP

30.5 % (g (tot cp) (g (tot prot))$^{-1}$) ($CP\%_{fc}$) of the protein fractional composition in SSUCM-M whereas CP formed even 75.0 % (g (tot cp) (g (tot prot))$^{-1}$) in case of SSUCM-R. Therefore, the value of M_{cyt} was also higher in the case of SSUCM-R. The lack of biosynthesis pathways is also the reason why the value of N_{etc} was about 1/3 lower (no biosynthesis energy costs) and $LIP\%_{mmc}$ was higher than that of SSUCM-M.

Note that the calculated biomass compositions can not be directly compared to compositions of real cells due to simplifications in SSUCMs. Real cells have much more complicated cell membranes and cell walls which include additional cell components (peptidoglycan, lipopolysaccharides etc) that were left out from SSUCMs. These differences explain also relatively high $PROT\%_{mmc}$ and low $LIP\%_{mmc}$ in SSUCMs.

N_{cp} value decreases with the SRS size increase because more synthesis equipment is needed for the UC and less space is available for CP in the cytoplasm. Therefore, most of the values of N_{cell_comp}, F_{cell_comp} and geometric cellular dimensions increase. The main exceptions are cell parameters related to DNA and membrane lipids. UCs must transport more substrate and produce more energy for cellular processes (mainly protein synthesis due to increased CP synthesis). Therefore, more membrane proteins are needed but the increase of S_{prot} exceeds the increases of S_{mem_i} and S_{tot} (change of surface to volume ratio of the cell). Eventually, the S_{lip} value starts to decrease to provide enough room for membrane proteins. Certainly, the proposed growth boundary value of $N_{lip} = 0$ is probably too extreme for real living cells and some kind of interim value exists that marks the upper limit for functional membranes. However, the main point is that even such simple UCs can not be very large and there will be also a growth boundary for them (determined by the membrane surface area for the current case). The increase in N_{cp} value also affects the cellular compositions. Considering macromolecular composition, the values of $DNA\%_{mmc}$ and $LIP\%_{mmc}$ must decrease whereas $RNA\%_{mmc}$ (more ribosomes and rRNA) and $PROT\%_{mmc}$ (more CP) must increase. Considering protein fractional composition, the values of $ETC\%_{fc}$ and $RPC\%_{fc}$ increase also noticeably beside $CP\%_{fc}$.

Supplementary Table 5. Numbers of cell components. Calculated values of numbers of cell component molecules/complexes (N_{cell_comp}) and molar concentrations of cell component/complexes (C_{cell_comp}) in the unit cell (UC) growing on minimal medium (SSUCM-M) and rich medium (SSUCM-R) at a constant value of cell cycle length of UC (3520 s = 1.0 h).

**Cell parameter**	**Symbol**	**Units**	**SSUCM-M**	**SSUCM-R**	**SSUCM-M**	**SSUCM-R**
UC mass	M_u	g (unit cell)$^{-1}$	$4.98 \cdot 10^{-13}$	$4.98 \cdot 10^{-13}$	$4.99 \cdot 10^{-12}$	$1.64 \cdot 10^{-11}$
Number of genomes in the cell	N_{dna}	molecules (genome) cell^{-1}	1	1	1	1
Molar concentration of genome	C_{dna}	mol (genome) L^{-1}	$3.3 \cdot 10^{-9}$	$3.3 \cdot 10^{-9}$	$3.3 \cdot 10^{-10}$	$1.0 \cdot 10^{-10}$
Number of replisome complexes (RCs) in the cell	N_{rc}	molecules (rc) cell^{-1}	2	2	2	2
Molar concentration of RCs	C_{rc}	mol (rc) L^{-1}	$6.7 \cdot 10^{-9}$	$6.7 \cdot 10^{-9}$	$6.7 \cdot 10^{-10}$	$2.0 \cdot 10^{-10}$
Number of effective replisome complexes in the cell	N_{rce}	molecules (rce) cell^{-1}	1.3	1.3	1.3	1.3
Molar concentration of effective replisome complexes	C_{rce}	mol (rce) L^{-1}	$4.4 \cdot 10^{-9}$	$4.4 \cdot 10^{-9}$	$4.4 \cdot 10^{-10}$	$1.3 \cdot 10^{-10}$
Number of molecules of the enzyme catalysing a single reaction r of central metabolic pathway PW_1	$N_{enz\_PW1\_r}$	molecules (enz PW_1) cell^{-1} reaction^{-1}	1864		$1.9 \cdot 10^{4}$	
Molar concentration of enzyme catalysing a single reaction r of central metabolic pathway PW_1	$C_{enz\_PW1\_r}$	mol (enz PW_1) L^{-1} reaction^{-1}	$6.2 \cdot 10^{-6}$		$6.4 \cdot 10^{-6}$	
Number of molecules of the enzyme catalysing a single reaction r of amino acid biosynthesis pathway PW_2	$N_{enz\_PW2\_r}$	molecules (enz PW_2) cell^{-1} reaction^{-1}	1700		$1.8 \cdot 10^{4}$	
Molar concentration of enzyme catalysing a single reaction r of amino acid biosynthesis pathway PW_2	$C_{enz\_PW2\_r}$	mol (enz PW_2) L^{-1} reaction^{-1}	$5.7 \cdot 10^{-6}$		$6.0 \cdot 10^{-6}$	
Number of molecules of the enzyme catalysing a single reaction r of deoxyribonucleotide biosynthesis pathway PW_3	$N_{enz\_PW3\_r}$	molecules (enz PW_3) cell^{-1} reaction^{-1}	26		26	
Molar concentration of enzyme catalysing a single reaction r of deoxyribonucleotide biosynthesis pathway PW_3	$C_{enz\_PW3\_r}$	mol (enz PW_3) L^{-1} reaction^{-1}	$8.8 \cdot 10^{-8}$		$8.8 \cdot 10^{-9}$	

Number of molecules of the enzyme catalysing a single reaction r of ribonucleotide biosynthesis pathway PW_4	$N_{enz\_PW4\_r}$	molecules (enz PW_4) $cell^{-1}$ $reaction^{-1}$	122		1291	
Molar concentration of enzyme catalysing a single reaction r of ribonucleotide biosynthesis pathway PW_4	$C_{enz\_PW4\_r}$	mol (enz PW_4) L^{-1} $reaction^{-1}$	$4.1 \cdot 10^{-7}$		$4.3 \cdot 10^{-7}$	
Number of molecules of the enzyme catalysing a single reaction r of lipid biosynthesis pathway PW_5	$N_{enz\_PW5\_r}$	molecules (enz PW_5) $cell^{-1}$ $reaction^{-1}$	16		0	
Molar concentration of enzyme catalysing a single reaction r of lipid biosynthesis pathway PW_5	$C_{enz\_PW5\_r}$	mol (enz PW_5) L^{-1} $reaction^{-1}$	$5.3 \cdot 10^{-8}$		0	
Number of electron transport chain (ETC) complexes in the cell	N_{etc}	molecules (etc) $cell^{-1}$	$1.3 \cdot 10^{4}$	8276	$1.3 \cdot 10^{5}$	$2.9 \cdot 10^{5}$
Molar concentration of ETC complexes	C_{etc}	mol (etc) L^{-1}	$4.2 \cdot 10^{-5}$	$2.8 \cdot 10^{-5}$	$4.2 \cdot 10^{-5}$	$2.9 \cdot 10^{-5}$
Number of cell membrane lipid molecules in the cell	N_{lip}	molecules (lip) $cell^{-1}$	$5.6 \cdot 10^{6}$	$7.6 \cdot 10^{6}$	0	0
Molar concentration of cell membrane lipid	C_{lip}	mol (lip) L^{-1}	0.019	0.025	0	0
Number of lipid synthesis enzyme (LPE) molecules in the cell	N_{lpe}	molecules (lpe) $cell^{-1}$	16	22	0	0
Molar concentration of LPE	C_{lpe}	mol (lpe) L^{-1}	$5.3 \cdot 10^{-8}$	$7.2 \cdot 10^{-8}$	0	0
Number of molecules of mRNA of the enzyme in the cell	N_{mrna_enz}	molecules (mrna enz) $cell^{-1}$	32		329	
Molar concentration of mRNA of enzyme	C_{mrna_enz}	mol (mrna enz) L^{-1}	$1.1 \cdot 10^{-7}$	0	$1.1 \cdot 10^{-7}$	0
Number of molecules of mRNA of cushioning protein (CP) in the cell	N_{mrna_cp}	molecules (mrna cp) $cell^{-1}$	129.69	315	1445	$1.1 \cdot 10^{4}$
Molar concentration of mRNA of CP	C_{mrna_cp}	mol (mrna cp) L^{-1}	$4.3 \cdot 10^{-7}$	$1.1 \cdot 10^{-6}$	$4.8 \cdot 10^{-7}$	$1.1 \cdot 10^{-6}$
Number of molecules of mRNA of RC in the cell	N_{mrna_rc}	molecules (mrna rc) $cell^{-1}$	0.01	0.01	0.01	0.014
Molar concentration of mRNA of RC	C_{mrna_rc}	mol (mrna rc) L^{-1}	$3.3 \cdot 10^{-11}$	$3.3 \cdot 10^{-11}$	$3.3 \cdot 10^{-12}$	$1.4 \cdot 10^{-12}$
Number of molecules of mRNA of ETC complex in the cell	N_{mrna_etc}	molecules (mrna etc) $cell^{-1}$	18	12	181	410
Molar concentration of mRNA of ETC complex	C_{mrna_etc}	mol (mrna etc) L^{-1}	$5.9 \cdot 10^{-8}$	$3.9 \cdot 10^{-8}$	$6.0 \cdot 10^{-8}$	$4.2 \cdot 10^{-8}$

Number of molecules of mRNA of LPE in the cell	N_{mrna_lpe}	molecules (mrna lpe) cell^{-1}	0.0034	0.0046	0	0
Molar concentration of mRNA of LPE	C_{mrna_lpe}	mol (mrna lpe) L^{-1}	$1.1\cdot10^{-11}$	$1.5\cdot10^{-11}$	0	0
Number of molecules of mRNA of transport protein in the cell	N_{mrna_stp}	molecules (mrna stp) cell^{-1}	0.5	0.5	5	18
Molar concentration of mRNA of transport protein	C_{mrna_stp}	mol (mrna stp) L^{-1}	$1.8\cdot10^{-9}$	$1.8\cdot10^{-9}$	$1.8\cdot10^{-9}$	$1.8\cdot10^{-9}$
Number of molecules of mRNA of RNA polymerase (RP) complex in the cell	N_{mrna_rp}	molecules (mrna rp) cell^{-1}	0.2	0.2	3	8
Molar concentration of mRNA of RP complex	C_{mrna_rp}	mol (mrna rp) L^{-1}	$8.0\cdot10^{-10}$	$8.0\cdot10^{-10}$	$8.5\cdot10^{-10}$	$8.5\cdot10^{-10}$
Number of molecules of mRNA of ribosomal protein complex (RPC) in the cell	N_{mrna_rpc}	molecules (mrna rpc) cell^{-1}	9	9	92	305
Molar concentration of mRNA of RPC	C_{mrna_rpc}	mol (mrna rpc) L^{-1}	$2.9\cdot10^{-8}$	$2.9\cdot10^{-8}$	$3.1\cdot10^{-8}$	$3.1\cdot10^{-8}$
Number of molecules of mRNA of transport protein in the cell	N_{stp}	molecules (stp) cell^{-1}	1864	1854	$1.9\cdot10^{4}$	$6.4\cdot10^{4}$
Molar concentration of mRNA of transport protein	C_{stp}	mol (stp) L^{-1}	$6.2\cdot10^{-6}$	$6.2\cdot10^{-6}$	$6.4\cdot10^{-6}$	$6.4\cdot10^{-6}$
Number of CP molecules in the cell	N_{cp}	molecules (cp) cell^{-1}	$6.1\cdot10^{5}$	$1.5\cdot10^{6}$	$6.8\cdot10^{6}$	$5.2\cdot10^{7}$
Molar concentration of CP	C_{cp}	mol (cp) L^{-1}	0.002	0.0049	0.0023	0.0053
Number of RP complexes in the cell	N_{rp}	molecules (rp) cell^{-1}	305	302	3226	$1.1\cdot10^{4}$
Molar concentration of RP complexes	C_{rp}	mol (rp) L^{-1}	$1.0\cdot10^{-6}$	$1.0\cdot10^{-6}$	$1.1\cdot10^{-6}$	$1.1\cdot10^{-6}$
Number of assembled rRNA complexes in the cell	N_{rrna}	molecules (rrna) cell^{-1}	8500	8423	$9.0\cdot10^{4}$	$3.0\cdot10^{5}$
Molar concentration of assembled rRNA complexes	C_{rrna}	mol (rrna) L^{-1}	$2.8\cdot10^{-5}$	$2.8\cdot10^{-5}$	$3.0\cdot10^{-5}$	$3.0\cdot10^{-5}$
Number of ribosomes in the cell	N_{rs}	molecules (rs) cell^{-1}	8500	8423	$9.0\cdot10^{4}$	$3.0\cdot10^{5}$
Molar concentration of ribosomes	C_{rs}	mol (rs) L^{-1}	$2.8\cdot10^{-5}$	$2.8\cdot10^{-5}$	$3.0\cdot10^{-5}$	$3.0\cdot10^{-5}$
Number of ribosomes for the synthesis of enzymes	N_{rs_enz}	molecules (rs enz) cell^{-1}	3178		$3.3\cdot10^{4}$	
Molar concentration of ribosomes for the synthesis of enzymes	C_{rs_enz}	mol (rs enz) L^{-1}	$1.1\cdot10^{-5}$	0	$1.1\cdot10^{-5}$	0
Number of ribosomes for the synthesis of CP	N_{rs_cp}	molecules (rs cp)	2594	6303	$2.8\cdot10^{4}$	$2.2\cdot10^{5}$

		cell^{-1}				
Molar concentration of ribosomes for the synthesis of CPs	C_{rs_cp}	mol (rs cp) L^{-1}	$8.7 \cdot 10^{-6}$	$2.1 \cdot 10^{-5}$	$9.6 \cdot 10^{-6}$	$2.3 \cdot 10^{-5}$
Number of ribosomes for the synthesis of RC	N_{rs_rc}	molecules (rs rc) cell^{-1}	1.4	1.4	1.4	1.4
Molar concentration of ribosomes for the synthesis of RCs	C_{rs_rc}	mol (rs rc) L^{-1}	$4.6 \cdot 10^{-9}$	$4.6 \cdot 10^{-9}$	$4.6 \cdot 10^{-10}$	$1.4 \cdot 10^{-10}$
Number of ribosomes for the synthesis of ETC complexes	N_{rs_etc}	molecules (rs etc) cell^{-1}	1775	1176	$1.8 \cdot 10^{4}$	$4.1 \cdot 10^{4}$
Molar concentration of ribosomes for the synthesis of ETC complexes	C_{rs_etc}	mol (rs etc) L^{-1}	$5.9 \cdot 10^{-6}$	$3.9 \cdot 10^{-6}$	$6.0 \cdot 10^{-6}$	$4.2 \cdot 10^{-6}$
Number of ribosomes for the synthesis of LPE	N_{rs_lpe}	molecules (rs lpe) cell^{-1}	0.068	0.092	0	0
Molar concentration of ribosomes for the synthesis of LPEs	C_{rs_lpe}	mol (rs lpe) L^{-1}	$2.3 \cdot 10^{-10}$	$3.1 \cdot 10^{-10}$	0	0
Number of ribosomes for the synthesis of transport proteins	N_{rs_stp}	molecules (rs stp) cell^{-1}	53	53	548	1804
Molar concentration of ribosomes for the synthesis of transport proteins	C_{rs_stp}	mol (rs stp) L^{-1}	$1.8 \cdot 10^{-7}$	$1.8 \cdot 10^{-7}$	$1.8 \cdot 10^{-7}$	$1.8 \cdot 10^{-7}$
Number of ribosomes for the synthesis of RP complexes	N_{rs_rp}	molecules (rs rp) cell^{-1}	24	24	255	841
Molar concentration of ribosomes for the synthesis of RP complexes	C_{rs_rp}	mol (rs rp) L^{-1}	$8.1 \cdot 10^{-8}$	$8.0 \cdot 10^{-8}$	$8.5 \cdot 10^{-8}$	$8.5 \cdot 10^{-8}$
Number of ribosomes for the synthesis of RPC	N_{rs_rpc}	molecules (rs rpc) cell^{-1}	874	867	9248	$3.1 \cdot 10^{4}$
Molar concentration of ribosomes for the synthesis of RPCs	C_{rs_rpc}	mol (rs rpc) L^{-1}	$2.9 \cdot 10^{-6}$	$2.9 \cdot 10^{-6}$	$3.1 \cdot 10^{-6}$	$3.1 \cdot 10^{-6}$
Number of tRNA molecules in the cell	N_{trna}	molecules (trna) cell^{-1}	$4.3 \cdot 10^{4}$	$4.2 \cdot 10^{4}$	$4.5 \cdot 10^{5}$	$1.5 \cdot 10^{6}$
Molar concentration of tRNA	C_{trna}	mol (trna) L^{-1}	0.0014	$1.4 \cdot 10^{-4}$	$1.5 \cdot 10^{-4}$	$1.5 \cdot 10^{-4}$

Supplementary Table 6. Metabolic fluxes. Calculated values of metabolic fluxes of reactions/pathways/processes (F_{cell_comp}) and specific productivities of cushioning protein (CP) synthesis (Q_{cp}) in the unit cell (UC) growing on minimal medium (SSUCM-M) and rich medium (SSUCM-R) at a constant value of cell cycle length of UC (3520 s = 1.0 h).

**Cell parameter**	**Symbol**	**Units**	**SSUCM-M**	**SSUCM-R**	**SSUCM-M**	**SSUCM-R**
UC mass	M_u	g (unit cell)$^{-1}$	$4.98\cdot10^{-13}$	$4.98\cdot10^{-13}$	$4.99\cdot10^{-12}$	$1.64\cdot10^{-11}$
DNA replication flux	F_{rce}	molecules (dnt) s^{-1} cell^{-1}	2636	2636	2636	2636
Flux of reaction r of central metabolic pathway PW_1	$F_{enz\_PW1\_r}$	molecules (metabolite) s^{-1} cell^{-1} reaction^{-1}	$1.9\cdot10^{5}$		$1.9\cdot10^{6}$	
Flux of reaction r of amino acid synthesis pathway PW_2	$F_{enz\_PW2\_r}$	molecules (metabolite) s^{-1} cell^{-1} reaction^{-1}	$1.7\cdot10^{5}$		$1.8\cdot10^{6}$	
Flux of reaction r of deoxyribonucleotide synthesis pathway PW_3	$F_{enz\_PW3\_r}$	molecules (metabolite) s^{-1} cell^{-1} reaction^{-1}	2636		2636	
Flux of reaction r of ribonucleotide synthesis pathway PW_4	$F_{enz\_PW4\_r}$	molecules (metabolite) s^{-1} cell^{-1} reaction^{-1}	$1.2\cdot10^{4}$		$1.3\cdot10^{5}$	
Flux of reaction r of lipid synthesis pathway PW_5	$F_{enz\_PW5\_r}$	molecules (metabolite) s^{-1} cell^{-1} reaction^{-1}	1596		0	
ATP synthesis flux of electron transport chain complex	F_{etc}	molecules (atp) s^{-1} cell^{-1}	$1.3\cdot10^{6}$	$8.3\cdot10^{5}$	$1.3\cdot10^{7}$	$2.9\cdot10^{7}$
Lipid synthesis flux	F_{lpe}	molecules (lip) s^{-1} cell^{-1}	1596	2164	0	0
Substrate transport flux	F_{stp}	molecules (substrate) s^{-1} cell^{-1}	$1.9\cdot10^{5}$	$1.9\cdot10^{5}$	$1.9\cdot10^{6}$	$6.4\cdot10^{6}$
Transcription flux	F_{rp}	molecules (nt) s^{-1} cell^{-1}	$1.2\cdot10^{4}$	$1.2\cdot10^{4}$	$1.3\cdot10^{5}$	$4.3\cdot10^{5}$
Translation flux	F_{rs}	molecules (aa) s^{-1} cell^{-1}	$1.7\cdot10^{5}$	$1.7\cdot10^{5}$	$1.8\cdot10^{6}$	$5.9\cdot10^{6}$
Specific productivity of CP synthesis	Q_{cp}	molecules (cp) (g (cell))$^{-1}$ h^{-1}	$8.7\cdot10^{17}$	$2.1\cdot10^{18}$	$9.6\cdot10^{17}$	$2.3\cdot10^{18}$

Supplementary Table 7. Cellular composition. Calculated values of compositions (dry weight content of macromolecular fraction in the cell ($cell\_comp\%_{mmc}$), dry weight content of cell component in the cell ($cell\_comp\%_{mc}$) and dry weight content of cell component in the corresponding macromolecular fraction ($cell\_comp\%_{fc}$)) of unit cells (UCs) growing on minimal medium (SSUCM-M) and rich medium (SSUCM-R) at constant value of cell cycle length of UC (3520 s = 1.0 h).

Cell parameter	Symbol	Units	SSUCM-M	SSUCM-R	SSUCM-M	SSUCM-R
UC mass	M_u	g (unit cell)$^{-1}$	$4.98 \cdot 10^{-13}$	$4.98 \cdot 10^{-13}$	$4.99 \cdot 10^{-12}$	$1.64 \cdot 10^{-11}$
Total dry weight content of cushioning protein in the cell	$CP\%_{mc}$	% (g (tot cp) (g (dw cell))$^{-1}$)	24.1	58.6	26.8	62.7
Macromolecular composition						
Total dry weight content of DNA fraction in the cell	$DNA\%_{mmc}$	% (g (tot dna) (g (dw cell))$^{-1}$)	3.2	3.2	0.32	0.097
Total dry weight content of lipid macromolecular fraction in the cell	$LIP\%_{mmc}$	% (g (tot lip) (g (dw cell))$^{-1}$)	2.4	3.3	0	0
Total dry weight content of protein fraction in the cell	$PROT\%_{mmc}$	% (g (tot prot) (g (dw cell))$^{-1}$)	79.1	78.3	83.5	83.7
Total dry weight content of RNA fraction in the cell	$RNA\%_{mmc}$	% (g (tot rna) (g (dw cell))$^{-1}$)	15.4	15.2	16.2	16.2
**Fractional composition (proteins)**						
Total dry weight content of cushioning protein in the protein fraction	$CP\%_{fc}$	% (g (tot cp) (g (tot prot))$^{-1}$)	30.5	74.8	50.6	75.0
Total dry weight content of enzymes of central and biosynthesis pathways in the protein fraction	$Enz\%_{fc}$	% (g (tot enz) (g (tot prot))$^{-1}$)	37.4		0.29	
Total dry weight content of electron transport chain complex in the protein fraction	$ETC\%_{fc}$	% (g (tot etc) (g (tot prot))$^{-1}$)	20.8	13.9	31.6	13.8
Total dry weight content of lipid synthesis enzyme in the protein fraction	$Lpe\%_{fc}$	% (g (tot lpe) (g (tot prot))$^{-1}$)	$8 \cdot 10^{-4}$	0.0011	0	0
Total dry weight content of replisome complex in the protein fraction	$RC\%_{fc}$	% (g (tot rc) (g (tot prot))$^{-1}$)	0.016	0.017	0.0024	$4.7 \cdot 10^{-4}$
Total dry weight content of RNA polymerase complex in the protein fraction	$RP\%_{fc}$	% (g (tot rp) (g (tot prot))$^{-1}$)	0.28	0.28	0.45	0.28
Total dry weight content of ribosomal protein complex in the protein fraction	$RPC\%_{fc}$	% (g (tot rpc) (g (tot prot))$^{-1}$)	10.4	10.3	16.2	10.3
Total dry weight content of transporter protein in the protein fraction	$STP\%_{fc}$	% (g (tot stp) (g (tot prot))$^{-1}$)	0.6	0.6	1.0	0.6
**Fractional composition (RNA)**						
Total dry weight content of mRNAs in the RNA	$mRNA\%_{fc}$	% (g (tot mrna)	2.0	2.0	2.0	2.0

fraction		(g (tot rna))$^{-1}$)				
Total dry weight content of assembled rRNA complex in the RNA fraction	$rRNA\%_{fc}$	% (g (tot rrna) (g (tot rna))$^{-1}$)	90.3	90.4	90.4	90.4
Total dry weight content of tRNA in the RNA fraction	$tRNA\%_{fc}$	% (g (tot trna) (g (tot rna))$^{-1}$)	7.6	7.6	7.6	7.6

Supplementary Table 8. Cell dimensions. Calculated values of geometrical parameters of the unit cell (UC) growing on minimal medium (SSUCM-M) and rich medium (SSUCM-R) at a constant value of cell cycle length of UC (3520 s = 1.0 h).

**Cell parameter**	**Symbol**	**Units**	**SSUCM-M**	**SSUCM-R**	**SSUCM-M**	**SSUCM-R**
UC mass	M_u	g (unit cell)$^{-1}$	$4.98 \cdot 10^{-13}$	$4.98 \cdot 10^{-13}$	$4.99 \cdot 10^{-12}$	$1.64 \cdot 10^{-11}$
Length of the cell	H_{cyl}	cm cell^{-1}	$7.8 \cdot 10^{-5}$	$7.8 \cdot 10^{-5}$	$1.7 \cdot 10^{-4}$	$2.5 \cdot 10^{-4}$
Mass of cytoplasm of the cell	M_{cyt}	g cyt^{-1}	$4.01 \cdot 10^{-13}$	$4.25 \cdot 10^{-13}$	$4.13 \cdot 10^{-12}$	$1.44 \cdot 10^{-11}$
Mass of cell membrane	M_{mem}	g mem^{-1}	$9.67 \cdot 10^{-14}$	$7.33 \cdot 10^{-14}$	$8.60 \cdot 10^{-13}$	$2.00 \cdot 10^{-12}$
Cell radius	R_{tot}	cm cell^{-1}	$3.6 \cdot 10^{-5}$	$3.6 \cdot 10^{-5}$	$7.7 \cdot 10^{-5}$	$1.1 \cdot 10^{-4}$
Relative cell membrane surface area covered by membrane lipids	S_{lip}/S_{mem_i}	cm^2 (tot lip)$^{-1}$ (cm^2 cell^{-1})$^{-1}$	0.54	0.70	0	0
The surface area of the internal layer of the cell membrane	S_{mem_i}	cm^2 (mem^{-1})	$2.9 \cdot 10^{-8}$	$3.0 \cdot 10^{-8}$	$1.4 \cdot 10^{-7}$	$3.1 \cdot 10^{-7}$
Relative cell membrane surface area covered by membrane proteins	S_{prot}/S_{mem_i}	cm^2 (tot prot)$^{-1}$ (cm^2 cell^{-1})$^{-1}$	0.46	0.30	1.0	1.0
The surface area of the cell	S_{tot}	cm^2 (cell)$^{-1}$	$3.3 \cdot 10^{-8}$	$3.3 \cdot 10^{-8}$	$1.6 \cdot 10^{-7}$	$3.4 \cdot 10^{-7}$

Supplementary Discussion 5.8: Unit cell parameters, complexity of SRS and growth media

Various UC parameter values were calculated from SSUCM-M and SSUCM-R based on experimentally determined data (cell size, growth rate, cell cycle parameters) of different *E. coli* strains grown on several complex and minimal growth media. UC parameter values of ref. [14] were calculated at *approximate* t_{CD} which is why the ratio of RNA to protein was constant in all cases (Supplementary Table 10). Data of ref. [18,20] included also experimentally determined t_{CD} values that varied between strains and growth media used. Therefore, calculated ratio of M_{rna} to M_{prot} was not constant but decreased gradually with increasing t_{CD} in the range of 0.03 – 0.07 (Supplementary Table 10). Ratio of M_{rna} to M_{prot} was also experimentally determined by ref. [18]. However, the cultivation experiments were carried out using turbidostat method and therefore growth points were located at maximal μ values. On the other hand, developed SSUCMs describe only the growth of UCs and their growth rate is usually considerably lower. Therefore, it was not possible to do direct fitting of whole measured and calculated data. However, it was possible to estimate the dependencies between data points and growth rate. Calculated ratios of UCs and experimentally determined ratios were plotted on the same graph and linear regression was applied (Supplementary Fig. 9). It is evident that the ratio of M_{rna} to M_{prot} calculated from SSUCMs based on standard parameter values and experimentally determined data of ref. [18] (cell size and t_{CD}) is considerably lower although the general trend is reproduced. There are probably several reasons: SSUCMs are simplified models and do not describe experimentally studied cells precisely (metabolic networks, missing cell wall components), cushioning mass is expected to be solely CP whereas in reality cushioning mass might include a long list of substances including inactive ribosomes, some cell parameters have growth dependent values but SSUCMs have currently constant parameter values etc.

The comparison of t_d and t_{CD} values showed that there are few more or less overlapping growth points: MG1655 strain growing on semi-complex media and both *E. coli* strains growing on minimal media. It was demonstrated that calculated and measured ratio of M_{rna} to M_{prot} could be approximately fitted lowering the value of k_{rs} from 20 to the range of 3 – 7 molecules (aa) s^{-1} rs^{-1}, for example (Supplementary Fig. 10, Supplementary Table 9). It must be stressed that the standard value of k_{rs} in SSUCMs is probably possible upper limit which is achieved during very fast growth. Slower growth, however, is characterized by lower k_{rs} values according to the literature[17]. Most probably, the real k_{rs} values corresponding to the data of ref. [18] are somewhat higher than found during the fitting because other above mentioned factors influence the calculation results also. In order to clarify the effect of different factors, strain-specific models are needed. It must be stressed that the ratio of M_{rna} to M_{prot} is not exactly equal to the ratio of M_{rrna} to M_{prot} which is used to describe the macromolecular fraction of ribosomes because of the synthesis of tRNA and mRNAs (Supplementary Eqs. (7)-(8)). The fraction of ribosomes can be described more precisely by the ratio of N_{rs_rpc} to N_{rs} derived from Supplementary Eq. (6):

$$\frac{N_{rs\_rpc}}{N_{rs}} = \frac{n_{rpc}}{t_{CD} \cdot k_{rs}} \tag{6}$$

The calculations showed that the ratio of N_{rs_rpc} to N_{rs} is somewhat higher than the ratio of M_{rna} to M_{prot}.

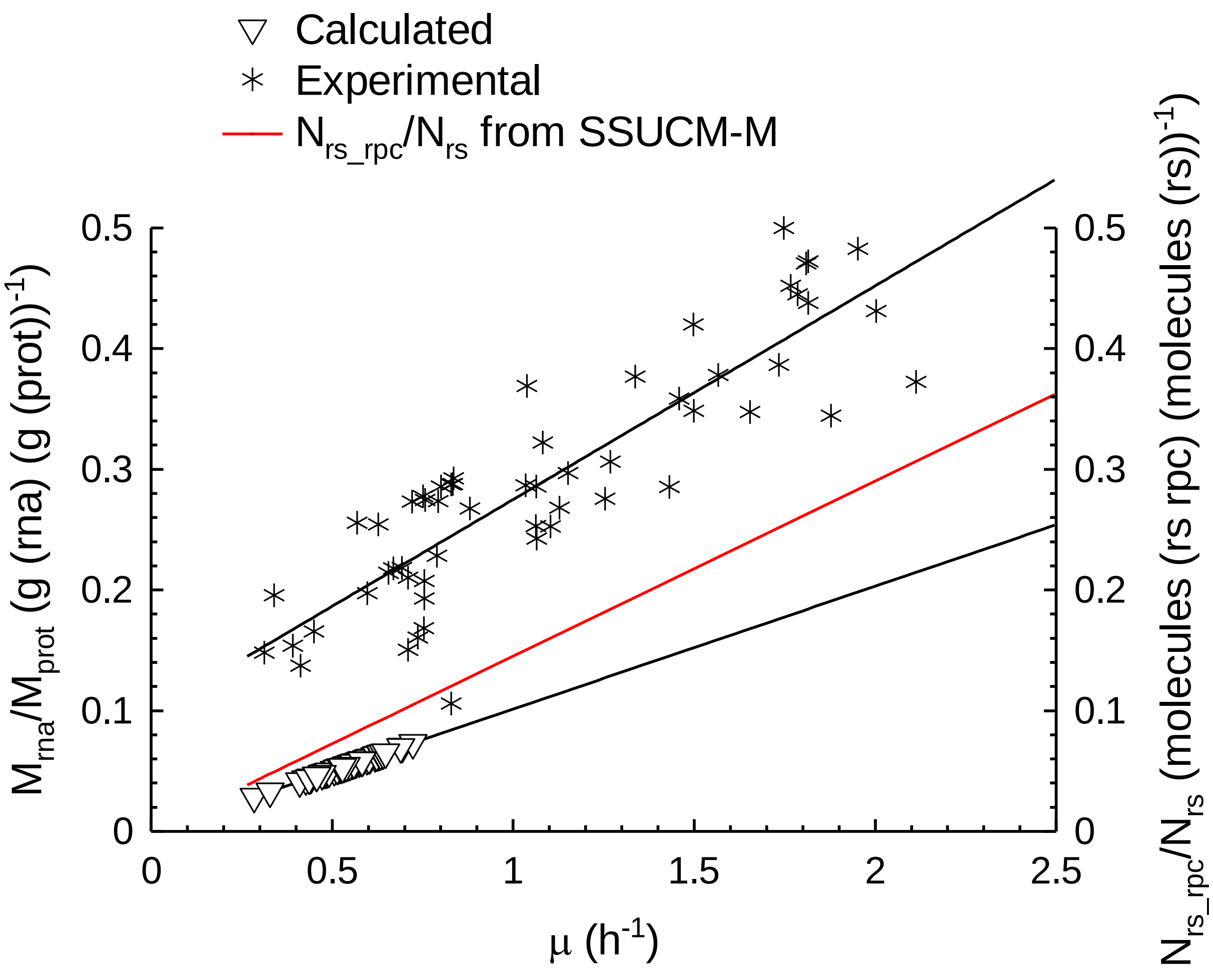

Supplementary Fig. 9. The calculated and experimentally determined ratios of RNA to proteins. The dependence between values of specific growth rate of the cell culture (μ, h^{-1}) and ratio of RNA to protein (M_{rna}/M_{prot}, g (rna) (g (prot))$^{-1}$). Experimentally determined values of M_{rna}/M_{prot} were taken from data of ref. [18] and calculated values of M_{rna}/M_{prot} were generated using SSUCM-M and SSUCM-R models based on standard parameter values (Supplementary Table 10). Both data-sets include linear regression lines. N_{rs_rpc} is the number of ribosomes for the synthesis of ribosomal proteins (molecules (rs rpc) cell^{-1}) and N_{rs} is the number of ribosomes in the cell (molecules (rs) cell^{-1}). The ratio of N_{rs_rpc} to N_{rs} characterizes the fraction of ribosomal proteins and is calculated according to Supplementary Eq. (6) based on standard parameter values.

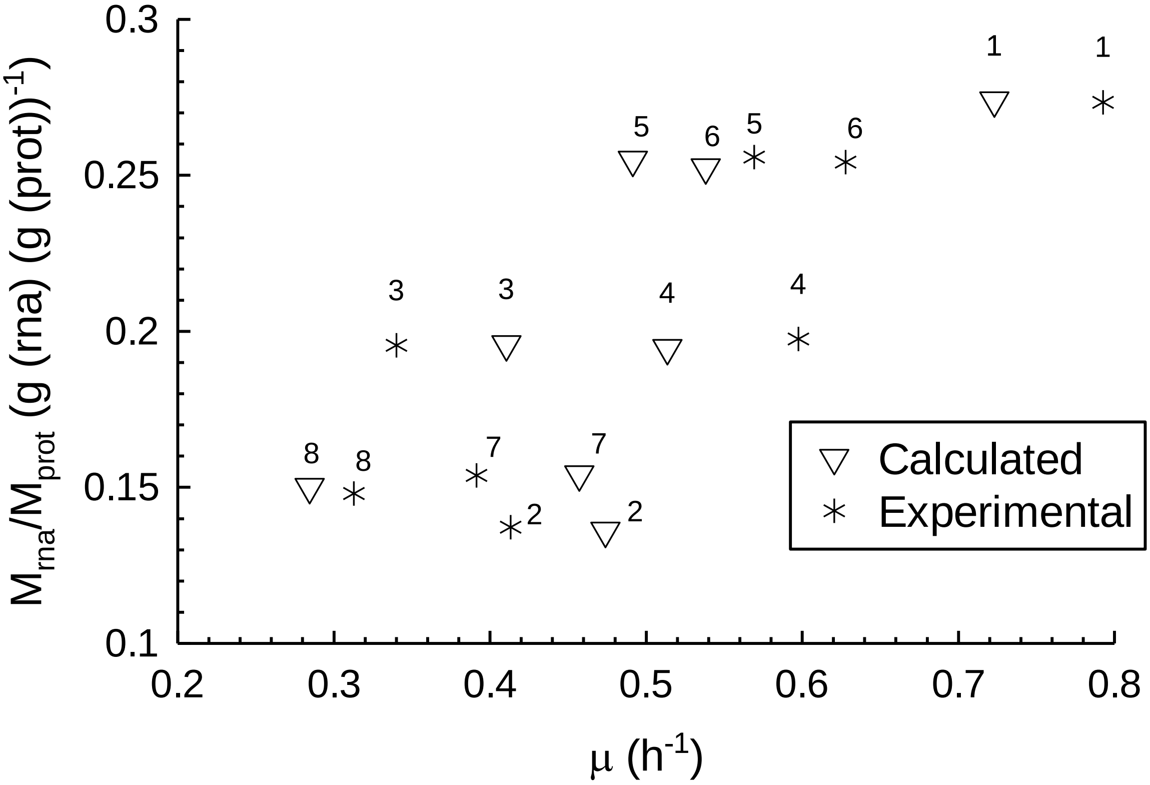

Supplementary Fig. 10. The correlation of the ratio of RNA to proteins between calculations and experiment. The dependence between values of specific growth rate of the cell culture (μ, h^{-1}) and ratio of RNA to protein (M_{rna}/M_{prot}, g (rna) (g (prot))$^{-1}$). Experimentally determined values of M_{rna}/M_{prot} were taken from data of ref. [18] and calculated values of M_{rna}/M_{prot} were generated using SSUCM-M (data points 1-3) and SSUCM-R (data points 4-8) models based on standard parameter values except the apparent working rate of the ribosome (k_{rs}). The value of k_{rs} was decreased during data fitting (Supplementary Table 9). Data points: 1 – MG1655 strain on M9 glucose + 3 a. a. + 0.2mM uracil, 2 and 3 – MG1655 strain on MOPS glycerol + 0.2mM uracil, 4 – NCM strain on MOPS glycerol, 5 and 6 – NCM strain on MOPS sorbitol, 7 and 8 – MG1655 strain on MOPS glycerol.

Supplementary Table 9. Input parameter values corresponding to fitted data. The values of the apparent working rate of the ribosome (k_{rs}, molecules (aa) s^{-1} rs^{-1}) corresponding to the fitted values of the ratio of RNA to protein (Supplementary Fig. 10) based on experimentally determined data of ref. [18] and calculated data using SSUCMs.

Data point	Strain	Growth condition	k_{rs}
1	MG1655	M9 glucose + 3 a. a. + 0.2mM uracil	5.1
2	MG1655	MOPS glycerol + 0.2mM uracil	6.7
3	MG1655	MOPS glycerol + 0.2mM uracil	4.0
4	NCM	MOPS glycerol	5.0
5	NCM	MOPS sorbitol	3.7
6	NCM	MOPS sorbitol	4.1
7	MG1655	MOPS glycerol	5.7
8	MG1655	MOPS glycerol	3.6

It was possible to estimate further dependencies of experimentally determined (various growth regions) and calculated (unit cell condition) data points of cell size and number of ribosomes (Supplementary Figs. 11-14). The comparison showed that calculated data points were sufficiently reasonably aligned with the trends of experimentally determined data points at different growth rates. It must be stressed that the calculated values of N_{rs_ave} from SSUCM-M fitted more or less to the same curve without changes in k_{rs} value (standard input parameter is used). Therefore, there seem to be considerable differences between different datasets in the literature which excludes single combination of input parameters for model.

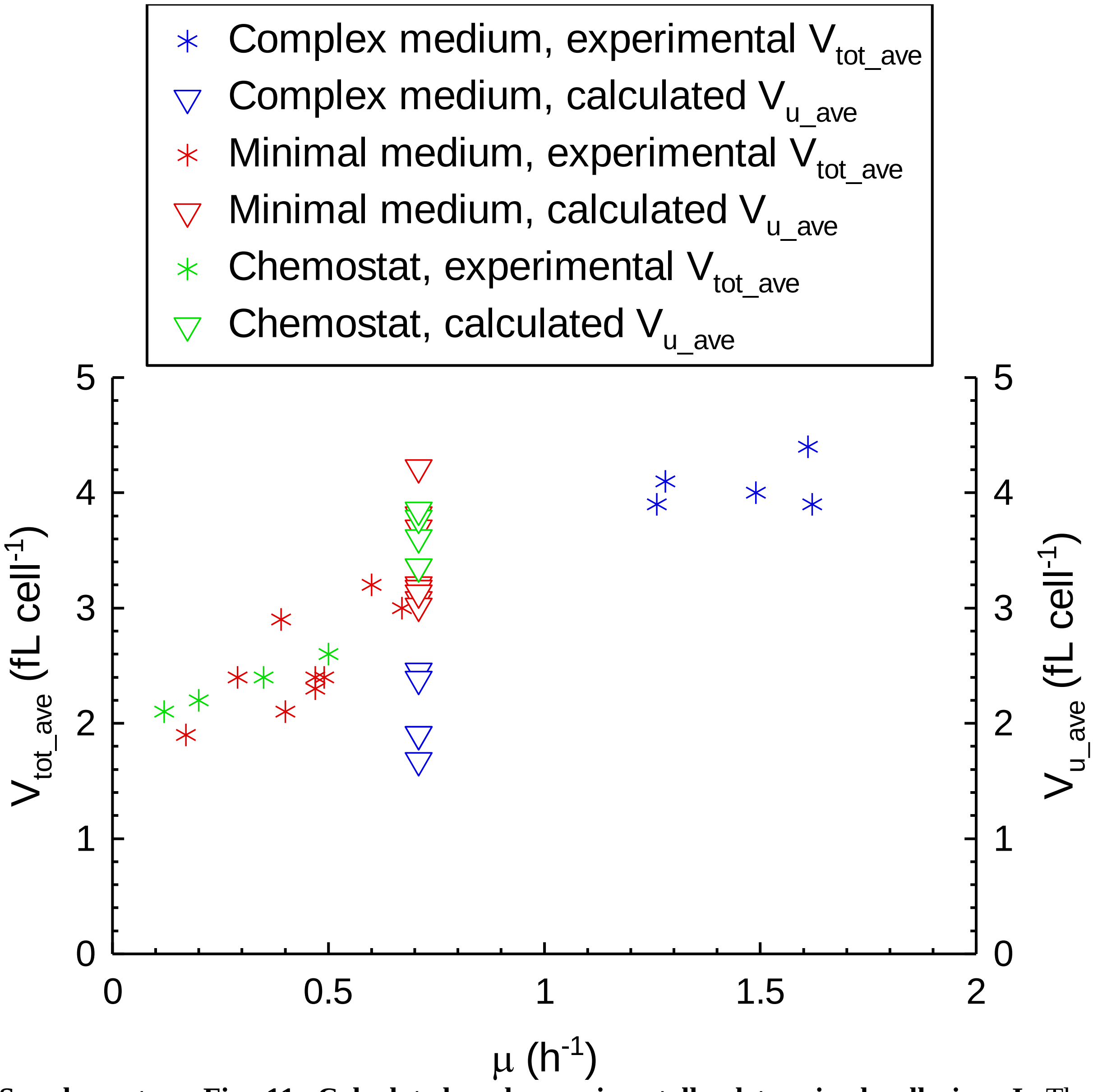

Supplementary Fig. 11. Calculated and experimentally determined cell sizes I. The dependence between values of specific growth rate of the cell culture (μ, h^{-1}) and cell size of *E. coli* cultures (batch, chemostat) growing on various complex and minimal media. Experimentally determined values of μ and average cell volume (V_{tot_ave}, fL $cell^{-1}$) were taken from the data of ref. [14] (Supplementary Table 1). The values of average unit cell volume (V_{u_ave}, fL $cell^{-1}$) were calculated from unit cell mass (M_u, g (unit $cell)^{-1}$) values (Supplementary Table 1) as: $V_{u\_ave} = M_u \cdot (1 + t_{a\_ave}/t_{CD})/(\rho_{tot} \cdot 10^{-12})$ assuming that $t_{a\_ave}/t_{CD} = 0.41$. Because constant standard value of cell cycle length of unit cell (t_{CD}, 3520 s = 1.0 h) was used for all M_u calculations, corresponding μ values were also constant (0.7 h^{-1}).

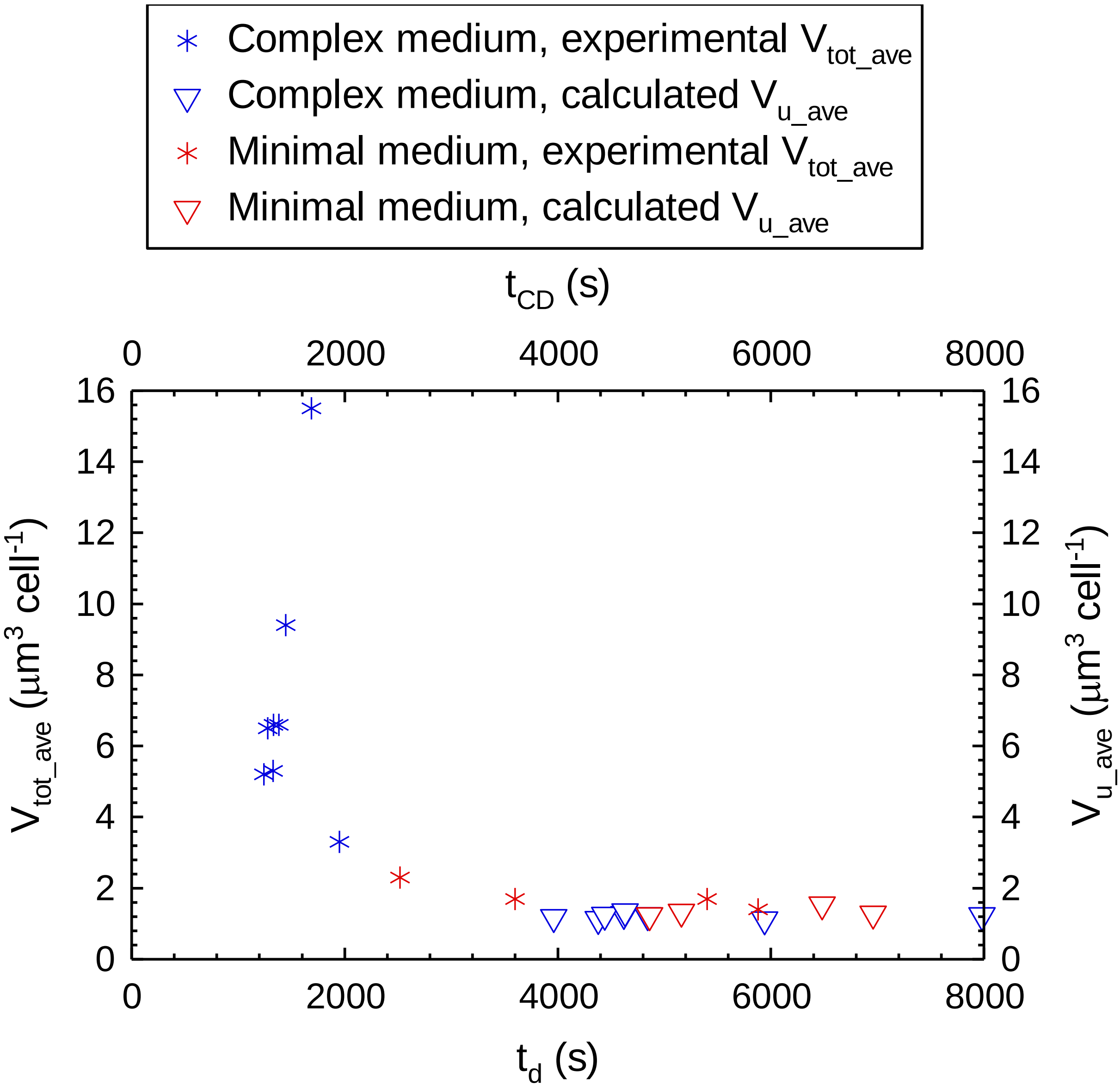

Supplementary Fig. 12. Calculated and experimentally determined cell sizes II. The dependence between experimentally determined values of cell cycle length (t_d, s) and average cell volume (V_{tot_ave}, µm^3 cell^{-1}) of *E. coli* cultures growing on various complex and minimal media based on data of ref. [20] (Supplementary Table 2). The dependence between calculated cell cycle length of unit cell (t_{CD}, s) and average unit cell volume (V_{u_ave}, µm^3 cell^{-1}). The values of V_{u_ave} were calculated from unit cell mass (M_u, g (unit cell)$^{-1}$) values (Supplementary Table 2) as: $V_{u\_ave} = M_u \cdot (1 + t_{a\_ave}/t_{CD})/\rho_{tot}$ assuming that $t_{a\_ave}/t_{CD} = 0.41$ The values of t_{CD} were calculated from Eq. (1) based on experimentally determined genome replication time (t_C, s) and division time (t_D, s) values (Supplementary Table 2). The values of t_d and t_{CD} in seconds (major tick mark values of x-axis) correspond to following values in hours: 0.6, 1.1, 1.7, 2.2.

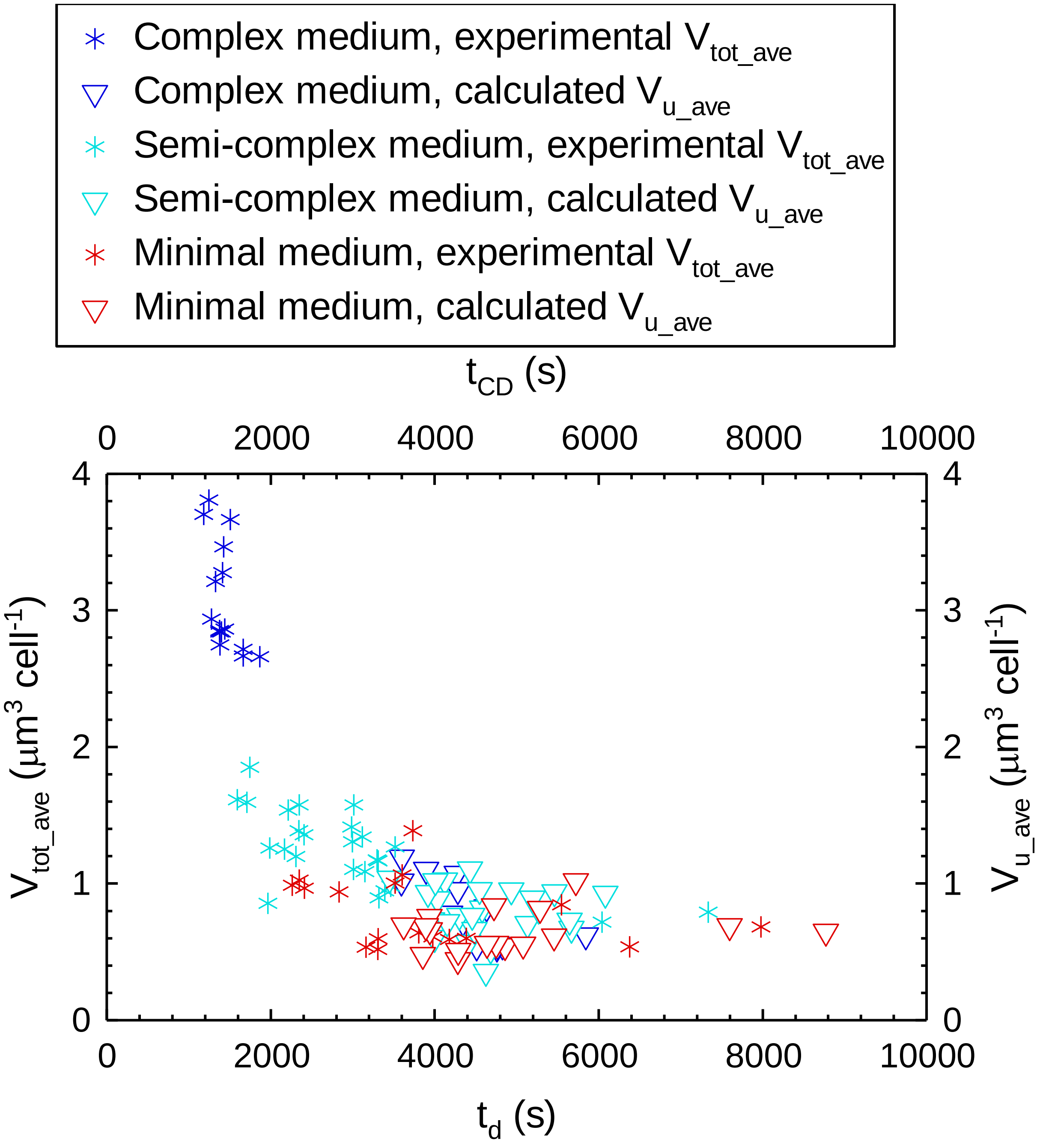

Supplementary Fig. 13. Calculated and experimentally determined cell sizes III. The dependence between experimentally determined values of cell cycle length (t_d, s) and average cell volume (V_{tot_ave}, µm^3 cell^{-1}) of *E. coli* cultures growing on various complex and minimal media based on data of ref. [18] (Supplementary Table 3). The dependence between calculated cell cycle length of unit cell (t_{CD}, s) and average unit cell volume (V_{u_ave}, µm^3 cell^{-1}). The values of V_{u_ave} were calculated from unit cell mass (M_u, g (unit cell)$^{-1}$) values (Supplementary Table 3) as: $V_{u\_ave} = M_u \cdot (1 + t_{a\_ave}/t_{CD})/\rho_{tot}$ assuming that $t_{a\_ave}/t_{CD} = 0.41$. The values of t_{CD} were calculated from Eq. (1) based on experimentally determined genome replication time (t_C, s) and division time (t_D, s) values (Supplementary Table 3). The values of t_d and t_{CD} in seconds (major tick mark values of x-axis) correspond to following values in hours: 0.6, 1.1, 1.7, 2.2, 2.8.

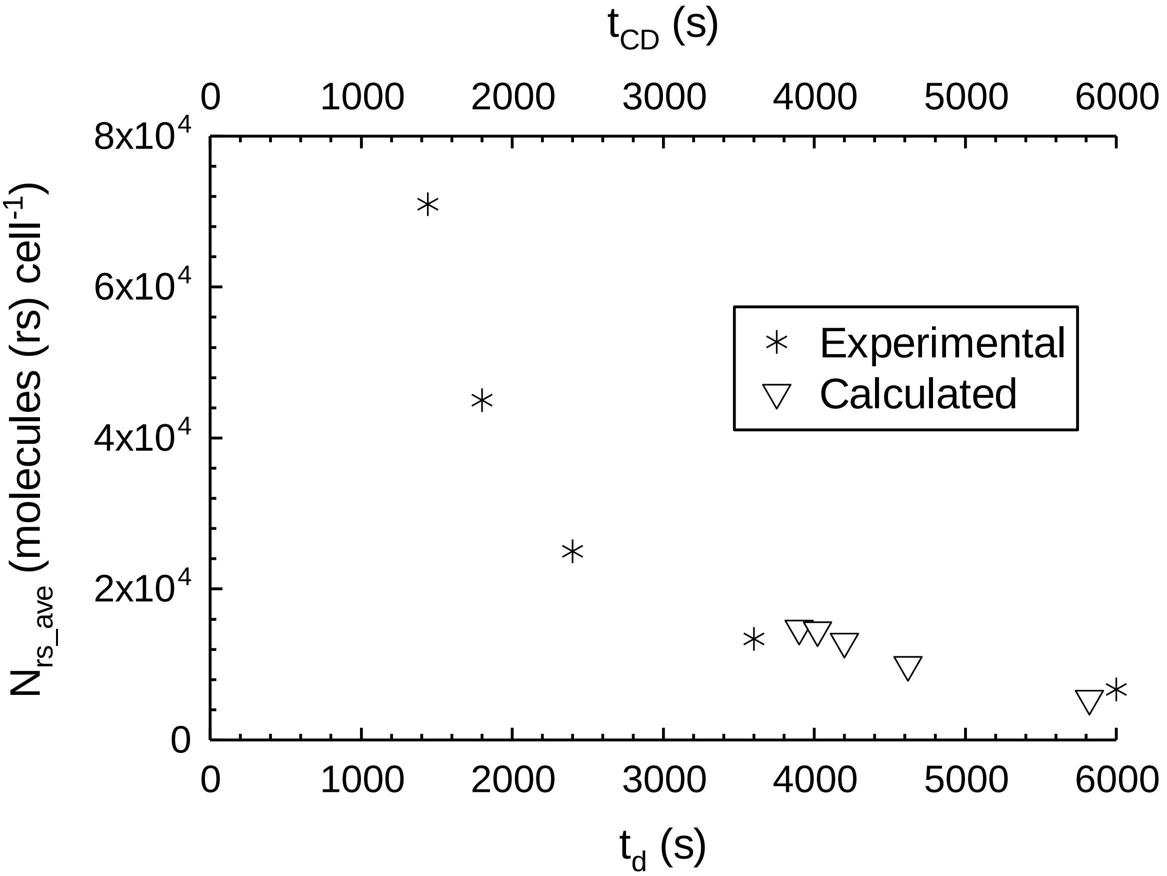

Supplementary Fig. 14. Calculated and experimentally determined numbers of ribosomes. The dependence between experimentally determined values of cell cycle length (t_d, s) and number of ribosomes in the average cell (N_{rs_ave}, molecules (rs) cell^{-1}) of *E. coli* based on data of ref. [17]. The dependence between calculated cell cycle length of unit cell (t_{CD}, s) and N_{rs_ave} for unit cells. The values of N_{rs_ave} of unit cells were calculated as: $N_{rs\_ave} = N_{rs} \cdot (1 + t_{a\_ave}/t_{CD})$ assuming that $t_{a\_ave}/t_{CD} = 0.41$. The values of N_{rs} were calculated from SSUCM-M model using standard parameter values, experimentally determined genome replication time (t_C, s) and division time (t_D, s) values[17]. The values of t_{CD} were calculated from Eq. (1) based on experimentally determined t_C and t_D values. The values of t_d and t_{CD} in seconds (major tick mark values of x-axis) correspond to following values in hours: 0.3, 0.6, 0.8, 1.1, 1.4, 1.7.

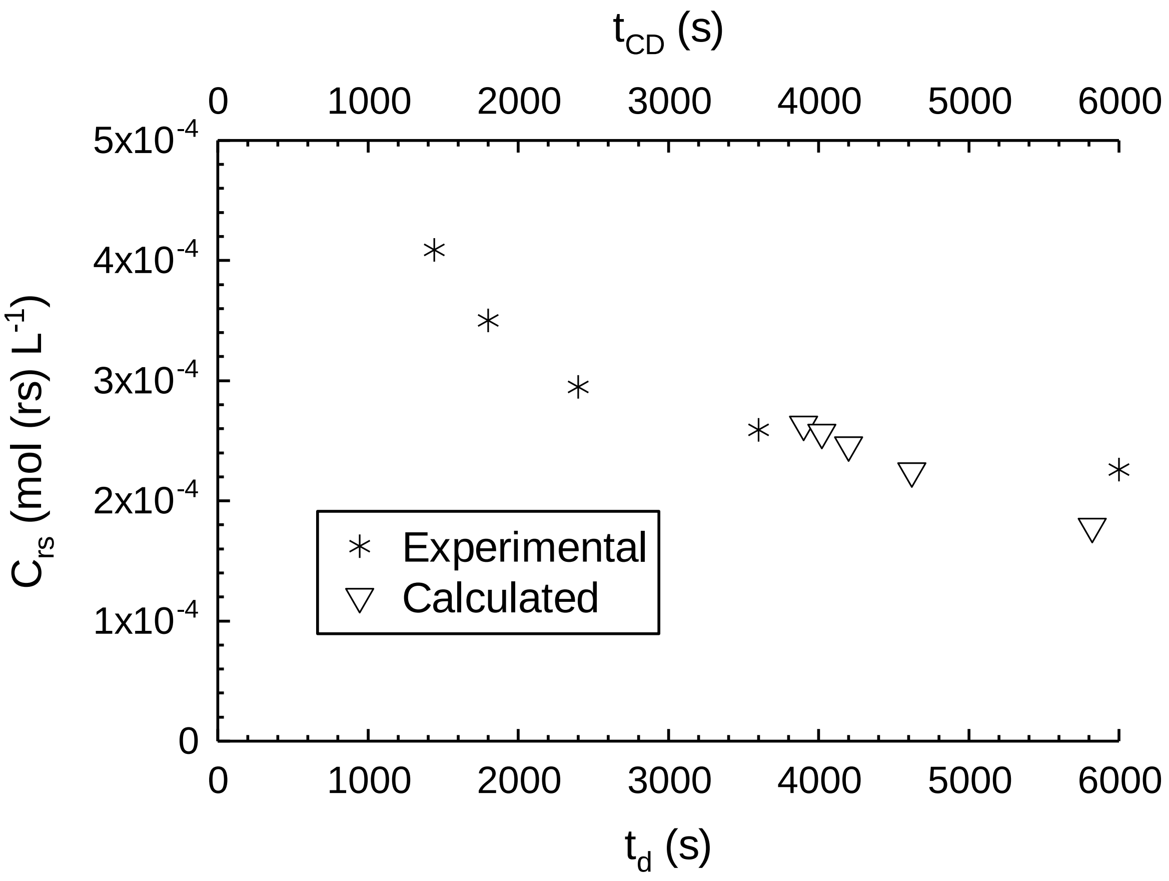

Supplementary Fig. 15. Calculated and experimentally determined concentrations of ribosomes. The dependence between experimentally determined values of cell cycle length (t_d, s) and calculated molar concentration of ribosomes (C_{rs}, mol (rs) L^{-1}) of *E. coli* based on data of ref. [17]. The values of C_{rs} were based on experimentally determined values of number of ribosomes in the average cell (N_{rs_ave}, molecules (rs) $cell^{-1}$) and experimentally determined values of dry weight cell mass of average cell ($M_{tot\_ave\_dw}$, g (dw $cell^{-1}$)): $C_{rs} = N_{rs\_ave} \cdot \rho_{tot} \cdot DWC \cdot 10^3 / (N_A \cdot M_{tot\_ave\_dw})$. The dependence between calculated cell cycle length of unit cell (t_{CD}, s) and C_{rs} for unit cells. The values of C_{rs} of unit cells were calculated from SSUCM-M model using standard parameter values, experimentally determined genome replication time (t_C, s) and division time (t_D, s) values[17]. The values of t_{CD} were calculated from Eq. (1) based on experimentally determined t_C and t_D values. The values of t_d and t_{CD} in seconds (major tick mark values of x-axis) correspond to following values in hours: 0.3, 0.6, 0.8, 1.1, 1.4, 1.7.

Additional calculated UC parameter values based on standard values of model input parameters have been stored in Supplementary Table 10 (Supplementary Figs. 16-56). It appears that the values of N_{rs}, N_{enz} and F_{etc} decrease and the ratio of S_{lip} to S_{tot} increase with t_{CD} increase. The dependences between calculated parameter values and M_u or growth media types do not show clear patterns except energy flux being larger and lipid area on membrane smaller in case of minimal media due to differences of energy requirements for biosynthesis. Also, calculated values of same UC parameters based on previously fitted k_{rs} values have been stored in Supplementary Table 11. Naturally, there are large differences between data of Supplementary Table 10 and data of Supplementary Table 11 due to considerable differences in k_{rs} values (approximate range of 65 – 85 %). For example (MG1655 strain on MOPS glycerol + 0.2mM uracil), the content of ribosomes, rRNA and RP increases approximately 70 % in Supplementary Table 11 because more ribosomes (also rRNA) are needed for translation

and more RPs are needed for the synthesis of rRNA. More translational synthesis equipment means that there is less room for CP in the cell and the content of CP decreases about 50 %. This leads to the overall protein content decrease of 25 %. Because protein synthesis is energetically most expensive, less ETCs are needed on membrane (decrease also approximately 25 %). Again, correlations between calculated parameter values and growth media types were not observed except that unit cells growing on richer media were larger and contained also more molecules.

It must be stressed again that simplified models are not suitable tools for specific data reproduction. Therefore, calculated values of unit cell parameters corresponding to fitted M_{rna}/M_{prot} values are probably still different compared to the real values. The advantage of simplified models is the possibility to explain complicated issues using simple examples.

Supplementary Table 10. Calculated unit cell parameter values based on standard values of model input parameters. Unit cell mass (M_u, g (unit cell)$^{-1}$), ratio of RNA to protein (M_{rna}/M_{prot}, g (rna) (g (prot))$^{-1}$), number of ribosomes in the cell (N_{rs}, molecules (rs) cell^{-1}), molar concentration of ribosomes (C_{rs}, mol (rs) L^{-1}), number of enzymes in the cell (N_{enz}, molecules (enz) cell^{-1}), total molar concentration of enzymes (C_{enz}, mol (enz) L^{-1}), energy flux (F_{etc}, molecules (atp) s^{-1} cell^{-1}), ratio of membrane surface covered by lipids to the cell surface (S_{lip}/S_{tot}, cm^2 (tot lip) ((cm^2 cell^{-1})$^{-1}$) and the apparent working rate of DNA polymerase (k_{dp}, molecules (dnt) s^{-1} dp^{-1}) values were calculated from experimentally determined cell parameter (Supplementary Tables 1-3) values of cells of *E. coli* grown on different mineral and rich media[14,18,20].

**Growth condition**	**M_u 10.1**	**M_{rna}/M_{prot} 10.8**	**N_{rs}**	**C_{rs} 10.13**	**N_{enz} 10.9**	**C_{enz} 10.13**	**F_{etc} 10.10**	**S_{lip}/S_{tot} 10.11**	**k_{dp} 10.12**
**Complex medium 10.2**									
Lysogeny broth (LB)	$1.35\cdot10^{-12}$	0.07	23651	$2.9\cdot10^{-5}$			$2.3\cdot10^{6}$	0.57	1000
LB MG1655	$1.19\cdot10^{-12}$	0.07	20782	$2.9\cdot10^{-5}$			$2.0\cdot10^{6}$	0.59	1000
Glucose + amino acids	$1.35\cdot10^{-12}$	0.07	23651	$2.9\cdot10^{-5}$			$2.3\cdot10^{6}$	0.57	1000
Mannose + amino acids	$1.74\cdot10^{-12}$	0.07	30653	$2.9\cdot10^{-5}$			$3.0\cdot10^{6}$	0.53	1000
Glycerol + amino acids	$1.69\cdot10^{-12}$	0.07	29754	$2.9\cdot10^{-5}$			$2.9\cdot10^{6}$	0.54	1000
**Carbon sources 10.3**									
Acetate	$2.70\cdot10^{-12}$	0.07	48295	$3.0\cdot10^{-5}$	$4.2\cdot10^{6}$	0.0026	$6.9\cdot10^{6}$	0.18	1000
Fumarate	$2.27\cdot10^{-12}$	0.07	40495	$3.0\cdot10^{-5}$	$3.5\cdot10^{6}$	0.0026	$5.8\cdot10^{6}$	0.23	1000
Galactose	$2.38\cdot10^{-12}$	0.07	42489	$3.0\cdot10^{-5}$	$3.7\cdot10^{6}$	0.0026	$6.1\cdot10^{6}$	0.22	1000
Glucose	$2.62\cdot10^{-12}$	0.07	46843	$3.0\cdot10^{-5}$	$4.0\cdot10^{6}$	0.0026	$6.7\cdot10^{6}$	0.19	1000
Glucose MG1655	$2.25\cdot10^{-12}$	0.07	40132	$3.0\cdot10^{-5}$	$3.5\cdot10^{6}$	0.0026	$5.7\cdot10^{6}$	0.23	1000
Glucosamine	$2.99\cdot10^{-}$	0.07	53560	$3.0\cdot10^{-}$	$4.6\cdot10^{6}$	0.0026	$7.6\cdot10^{6}$	0.16	1000

	12			5					
Glycerol	$2.18\cdot10^{-12}$	0.07	38863	$3.0\cdot10^{-5}$	$3.4\cdot10^{6}$	0.0026	$5.5\cdot10^{6}$	0.24	1000
Pyruvate	$2.14\cdot10^{-12}$	0.07	38138	$3.0\cdot10^{-5}$	$3.3\cdot10^{6}$	0.0026	$5.4\cdot10^{6}$	0.25	1000
Succinate	$2.22\cdot10^{-12}$	0.07	39588	$3.0\cdot10^{-5}$	$3.4\cdot10^{6}$	0.0026	$5.6\cdot10^{6}$	0.24	1000
**Fixed μ on glucose** [10.3]									
Chemostat μ = 0.5 h^{-1}	$2.38\cdot10^{-12}$	0.07	42489	$3.0\cdot10^{-5}$	$3.7\cdot10^{6}$	0.0026	$6.1\cdot10^{6}$	0.22	1000
Chemostat μ = 0.35 h^{-1}	$2.56\cdot10^{-12}$	0.07	45755	$3.0\cdot10^{-5}$	$3.9\cdot10^{6}$	0.0026	$6.5\cdot10^{6}$	0.20	1000
Chemostat μ = 0.2 h^{-1}	$2.68\cdot10^{-12}$	0.07	47932	$3.0\cdot10^{-5}$	$4.1\cdot10^{6}$	0.0026	$6.8\cdot10^{6}$	0.19	1000
Chemostat μ = 0.12 h^{-1}	$2.73\cdot10^{-12}$	0.07	48840	$3.0\cdot10^{-5}$	$4.2\cdot10^{6}$	0.0026	$6.9\cdot10^{6}$	0.18	1000
**Complex medium** [10.4]									
LB 5 ng/mL cTc	$8.70\cdot10^{-13}$	0.03	7237.61	$1.4\cdot10^{-5}$			703674	0.83	477
LB 10 ng/mL cTc	$7.88\cdot10^{-13}$	0.04	8568.79	$1.8\cdot10^{-5}$			835100	0.78	614
LB 20 ng/mL cTc	$8.96\cdot10^{-13}$	0.06	12256	$2.3\cdot10^{-5}$			$1.2\cdot10^{6}$	0.71	967
LB 30 ng/mL cTc	$7.97\cdot10^{-13}$	0.06	11381	$2.4\cdot10^{-5}$			$1.1\cdot10^{6}$	0.71	1074
LB 50 ng/mL cTc	$8.35\cdot10^{-13}$	0.06	13041	$2.6\cdot10^{-5}$			$1.3\cdot10^{6}$	0.68	1487
LB broth	$8.79\cdot10^{-13}$	0.06	12444	$2.4\cdot10^{-5}$			$1.2\cdot10^{6}$	0.70	1017
RDM+glucose	$8.73\cdot10^{-13}$	0.05	11446	$2.2\cdot10^{-5}$			$1.1\cdot10^{6}$	0.73	943
Glucose cAA	$9.51\cdot10^{-}$	0.05	13003	$2.3\cdot10^{-}$			$1.3\cdot10^{6}$	0.71	921

	13			5					
**Carbon sources 10.5**									
Glucose	$8.74\cdot10^{-13}$	0.05	11488	$2.2\cdot10^{-5}$	982176	0.0019	$1.6\cdot10^{6}$	0.60	879
Glycerol	$9.44\cdot10^{-13}$	0.05	11787	$2.1\cdot10^{-5}$	$1.0\cdot10^{6}$	0.0018	$1.7\cdot10^{6}$	0.62	806
Acetate	$1.09\cdot10^{-12}$	0.04	11109	$1.7\cdot10^{-5}$	936838	0.0014	$1.5\cdot10^{6}$	0.68	667
Mannose	$9.02\cdot10^{-13}$	0.04	8559	$1.6\cdot10^{-5}$	721651	0.0013	$1.2\cdot10^{6}$	0.72	569
**NCM3722 strain complex media 10.6**									
MOPS glucose synthetic rich	$3.83\cdot10^{-13}$	0.06	5150	$2.2\cdot10^{-5}$			505663	0.78	930
	$3.86\cdot10^{-13}$	0.05	4935	$2.1\cdot10^{-5}$			484261	0.79	908
	$3.89\cdot10^{-13}$	0.05	4934	$2.1\cdot10^{-5}$			484002	0.80	973
	$3.77\cdot10^{-13}$	0.05	4830	$2.1\cdot10^{-5}$			474086	0.80	902
TSB	$5.11\cdot10^{-13}$	0.06	7218	$2.3\cdot10^{-5}$			707272	0.75	1026
	$3.88\cdot10^{-13}$	0.05	4965	$2.1\cdot10^{-5}$			487182	0.79	907
	$5.34\cdot10^{-13}$	0.06	8146	$2.5\cdot10^{-5}$			798742	0.73	1334
**NCM3722 strain semi-complex media 10.6**									
MOPS glucose + 12 a. a.	$5.42\cdot10^{-13}$	0.06	7749	$2.4\cdot10^{-5}$			759079	0.74	876
	$4.03\cdot10^{-13}$	0.06	5539	$2.3\cdot10^{-5}$			543761	0.77	914
	$4.27\cdot10^{-}$	0.06	6414	$2.5\cdot10^{-}$			629962	0.75	1150

	13			5					
MOPS glucose + 6 a. a.	$2.50\cdot10^{-13}$	0.05	3183	$2.1\cdot10^{-5}$			314077	0.82	1002
	$3.68\cdot10^{-13}$	0.05	4765	$2.2\cdot10^{-5}$			467826	0.79	982
	$5.18\cdot10^{-13}$	0.06	7921	$2.5\cdot10^{-5}$			776845	0.73	850
MOPS glucose + casamino acids	$4.68\cdot10^{-13}$	0.06	6395	$2.3\cdot10^{-5}$			626885	0.77	921
**NCM3722 strain minimal media** [10.7]									
MOPS glucose	$4.68\cdot10^{-13}$	0.06	7220	$2.6\cdot10^{-5}$	630248	0.0022	$1.1\cdot10^{6}$	0.60	1182
	$5.36\cdot10^{-13}$	0.06	8338	$2.6\cdot10^{-5}$	725826	0.0023	$1.2\cdot10^{6}$	0.58	1102
	$4.88\cdot10^{-13}$	0.06	7626	$2.6\cdot10^{-5}$	665471	0.0023	$1.1\cdot10^{6}$	0.59	1110
	$4.91\cdot10^{-13}$	0.07	8175	$2.8\cdot10^{-5}$	716274	0.0024	$1.2\cdot10^{6}$	0.55	1119
MOPS glycerol	$3.13\cdot10^{-13}$	0.06	4367	$2.3\cdot10^{-5}$	383557	0.0020	644255	0.68	876
	$3.84\cdot10^{-13}$	0.05	4870	$2.1\cdot10^{-5}$	422655	0.0018	703440	0.70	694
	$3.38\cdot10^{-13}$	0.07	5208	$2.6\cdot10^{-5}$	458924	0.0023	772326	0.63	1043
MOPS sorbitol	$3.92\cdot10^{-13}$	0.05	4780	$2.0\cdot10^{-5}$	413831	0.0018	687524	0.71	658
	$3.97\cdot10^{-13}$	0.05	5142	$2.2\cdot10^{-5}$	446384	0.0019	742978	0.69	876
	$3.60\cdot10^{-13}$	0.06	5077	$2.3\cdot10^{-5}$	443995	0.0021	743149	0.66	876
	$3.96\cdot10^{-13}$	0.05	5239	$2.2\cdot10^{-5}$	455292	0.0019	758429	0.68	738

**MG1655 strain complex media $^{10.6}$**							
MOPS glucose synthetic rich	$5.54\cdot10^{-13}$	0.06	8102	$2.4\cdot10^{-5}$	793743	0.73	871
	$7.19\cdot10^{-13}$	0.07	12147	$2.8\cdot10^{-5}$	$1.2\cdot10^{6}$	0.66	998
	$8.44\cdot10^{-13}$	0.07	14323	$2.8\cdot10^{-5}$	$1.4\cdot10^{6}$	0.64	1121
	$7.60\cdot10^{-13}$	0.06	11072	$2.4\cdot10^{-5}$	$1.1\cdot10^{6}$	0.71	934
	$7.81\cdot10^{-13}$	0.06	12340	$2.6\cdot10^{-5}$	$1.2\cdot10^{6}$	0.68	1025
MOPS glycerol synthetic rich	$6.75\cdot10^{-13}$	0.06	9772	$2.4\cdot10^{-5}$	955925	0.72	980
	$5.87\cdot10^{-13}$	0.05	7875	$2.2\cdot10^{-5}$	770438	0.75	967
MOPS mannose synthetic rich	$4.40\cdot10^{-13}$	0.04	4731	$1.8\cdot10^{-5}$	462820	0.82	810
**MG1655 strain semi-complex media $^{10.6}$**							
M9 glucose + 3 a. a.	$4.99\cdot10^{-13}$	0.05	6070	$2.0\cdot10^{-5}$	593873	0.79	894
	$7.82\cdot10^{-13}$	0.06	11052	$2.4\cdot10^{-5}$	$1.1\cdot10^{6}$	0.71	750
M9 glucose + 3 a. a. + 0.2mM uracil	$5.80\cdot10^{-13}$	0.05	7855	$2.3\cdot10^{-5}$	768601	0.75	830
	$7.34\cdot10^{-13}$	0.07	12841	$2.9\cdot10^{-5}$	$1.3\cdot10^{6}$	0.65	861
MOPS glucose + 0.2mM uracil	$7.25\cdot10^{-13}$	0.06	10863	$2.5\cdot10^{-5}$	$1.1\cdot10^{6}$	0.70	728
	$6.75\cdot10^{-13}$	0.06	9266	$2.3\cdot10^{-5}$	905864	0.74	746
MOPS glucose + 12 a. a.	$6.73\cdot10^{-13}$	0.05	8594	$2.1\cdot10^{-5}$	839583	0.76	791

MOPS glucose + 12 a. a. + 0.2mM uracil	$4.75\cdot10^{-13}$	0.04	5271	$1.8\cdot10^{-5}$			515452	0.81	644
MOPS glucose + 6 a. a.	$6.32\cdot10^{-13}$	0.05	7689	$2.0\cdot10^{-5}$			751102	0.77	667
	$5.17\cdot10^{-13}$	0.04	5787	$1.9\cdot10^{-5}$			565598	0.80	853
	$6.63\cdot10^{-13}$	0.05	7732	$1.9\cdot10^{-5}$			754721	0.78	824
MOPS glucose + 6 a. a. + 0.2mM uracil	$6.28\cdot10^{-13}$	0.06	9538	$2.5\cdot10^{-5}$			934065	0.71	1081
	$6.59\cdot10^{-13}$	0.06	10303	$2.6\cdot10^{-5}$			$1.0\cdot10^{6}$	0.70	932
MOPS glucose + casamino acids	$5.42\cdot10^{-13}$	0.06	7499	$2.3\cdot10^{-5}$			734284	0.75	678
	$5.10\cdot10^{-13}$	0.06	7485	$2.4\cdot10^{-5}$			733773	0.74	821
	$7.22\cdot10^{-13}$	0.06	11100	$2.6\cdot10^{-5}$			$1.1\cdot10^{6}$	0.70	707
MOPS glycerol + 0.2mM uracil	$5.75\cdot10^{-13}$	0.05	6881	$2.0\cdot10^{-5}$			672489	0.78	582
	$6.56\cdot10^{-13}$	0.04	6940	$1.8\cdot10^{-5}$			676852	0.80	590
**MG1655 strain minimal media** $^{10.7}$									
MOPS glucose	$5.91\cdot10^{-13}$	0.05	7846	$2.2\cdot10^{-5}$	675615	0.0019	$1.1\cdot10^{6}$	0.64	747
	$5.78\cdot10^{-13}$	0.05	6940	$2.0\cdot10^{-5}$	594885	0.0017	980201	0.68	802
	$7.22\cdot10^{-13}$	0.04	8133	$1.9\cdot10^{-5}$	692483	0.0016	$1.1\cdot10^{6}$	0.69	677
MOPS glycerol	$4.35\cdot10^{-13}$	0.05	5001	$1.9\cdot10^{-5}$	430565	0.0016	712273	0.72	508
	$4.60\cdot10^{-}$	0.03	3422	$1.2\cdot10^{-}$	289822	0.0011	473643	0.83	655

	13			5					
	$4.88\cdot10^{-13}$	0.03	4163	$1.4\cdot10^{-5}$	353523	0.0012	578788	0.79	365

[10.1] Taken from Supplementary Tables 1-3, respectively.
[10.2] Values of UC parameters were calculated from SSUCM-R using values of previously calculated M_u in addition to standard values of cell parameters[3].
[10.3] Values of UC parameters were calculated from SSUCM-M using values of previously calculated M_u in addition to standard values of cell parameters[3].
[10.4] Values of UC parameters were calculated from SSUCM-R using values of previously calculated M_u, experimentally determined t_C and t_D (Supplementary Table 2) in addition to standard values of cell parameters[3].
[10.5] Values of UC parameters were calculated from SSUCM-M using values of previously calculated M_u, experimentally determined t_C and t_D (Supplementary Table 2) in addition to standard values of cell parameters[3].
[10.6] Values of UC parameters were calculated from SSUCM-R using values of previously calculated M_u, experimentally determined t_C and t_D (Supplementary Table 3) in addition to standard values of cell parameters[3].
[10.7] Values of UC parameters were calculated from SSUCM-M using values of previously calculated M_u, experimentally determined t_C and t_D (Supplementary Table 3) in addition to standard values of cell parameters[3].
[10.8] The ratio of M_{rna} to M_{prot} for SSUCM-M was calculated according to the following equation:

$$\frac{M_{rna}}{M_{prot}} = \frac{N_{rrna}\cdot m_{rrna} + N_{trna}\cdot m_{trna} + \sum_{1}^{8} N_{mrna\_cell\_comp}\cdot m_{mrna\_cell\_comp}}{N_{rc}\cdot m_{rc} + N_{rp}\cdot m_{rp} + N_{rs}\cdot m_{rpc} + N_{lpe}\cdot m_{lpe} + m_{enz}\cdot\sum_{i=1}^{5} N_{enz\_PW_i\_r}\cdot l_{PW_i} + N_{cp}\cdot m_{cp} + N_{stp}\cdot m_{stp} + N_{etc}\cdot m_{etc}} = \frac{t_{CD}\cdot N_{rp}\cdot k_{rp}}{t_{CD}\cdot N_{rs}\cdot k_{rs}} = \frac{F_{rp}}{F_{rs}} \tag{7}$$

The ratio of M_{rna} to M_{prot} for SSUCM-R was calculated according to the following equation:

$$\frac{M_{rna}}{M_{prot}} = \frac{N_{rrna}\cdot m_{rrna} + N_{trna}\cdot m_{trna} + \sum_{1}^{7} N_{mrna\_cell\_comp}\cdot m_{mrna\_cell\_comp}}{N_{rc}\cdot m_{rc} + N_{rp}\cdot m_{rp} + N_{rs}\cdot m_{rpc} + N_{lpe}\cdot m_{lpe} + N_{cp}\cdot m_{cp} + N_{stp}\cdot m_{stp} + N_{etc}\cdot m_{etc}} = \frac{t_{CD}\cdot N_{rp}\cdot k_{rp}}{t_{CD}\cdot N_{rs}\cdot k_{rs}} = \frac{F_{rp}}{F_{rs}} \tag{8}$$

[10.9] N_{enz} was calculated according to the following equation:

$$N_{enz} = \sum_{i=1}^{5} N_{enz\_PW_i\_r}\cdot l_{PW_i} \tag{9}$$

[10.10] F_{etc} was calculated according to the Supplementary Eq. (117) of ref. [3].

[10.11] The ratio of S_{lip} to S_{tot} was calculated according to the Supplementary Eqs. (122), (125) of ref. [3].
[10.12] k_{dp} was calculated according to the Supplementary Eq. (114) of ref. [3].
[10.13] C_{cell_comp} was calculated according to the Supplementary Eq. (24).

Supplementary Table 11. Calculated unit cell parameter values based on fitted values of model input parameters. Unit cell mass (M_u, g (unit cell)$^{-1}$), ratio of RNA to protein (M_{rna}/M_{prot}, g (rna) (g (prot))$^{-1}$), number of ribosomes in the cell (N_{rs}, molecules (rs) cell^{-1}), molar concentration of ribosomes (C_{rs}, mol (rs) L^{-1}), number of enzymes in the cell (N_{enz}, molecules (enz) cell^{-1}), total molar concentration of enzymes (C_{enz}, mol (enz) L^{-1}), energy flux (F_{etc}, molecules (atp) s^{-1} cell^{-1}), ratio of membrane surface covered by lipids to the cell surface (S_{lip}/S_{tot}, cm^2 (tot lip) ((cm^2 cell^{-1})$^{-1}$) and the apparent working rate of DNA polymerase (k_{dp}, molecules (dnt) s^{-1} dp^{-1}) values were calculated from experimentally determined cell parameter (Supplementary Table 3) values of cells of *E. coli* grown on different mineral and rich media[18]. The calculations were carried out using SSUCM-M and SSUCM-R, standard input parameter values of models and fitted values (minimal differences between M_{rna}/M_{prot} values, Supplementary Fig. 10) of the apparent working rate of ribosome (k_{rs}, molecules (aa) s^{-1} rs^{-1}).

**Growth condition**	M_u [11.1]	M_{rna}/M_{prot} [11.4]	N_{rs}	C_{rs} [11.9]	N_{enz} [11.5]	C_{enz} [11.9]	F_{etc} [11.6]	S_{lip}/S_{tot} [11.7]	k_{dp} [11.8]	k_{rs}
**NCM3722 strain minimal media [11.3]**										
MOPS glycerol	$3.84\cdot10^{-13}$	0.20	14457	$6.2\cdot10^{-5}$	359304	0.0016	651365	0.72	694	5.1
MOPS sorbitol	$3.92\cdot10^{-13}$	0.25	17312	$7.3\cdot10^{-5}$	334267	0.0014	622128	0.74	658	6.7
	$3.96\cdot10^{-13}$	0.25	17384	$7.3\cdot10^{-5}$	370788	0.0016	688960	0.71	738	4.0
**MG1655 strain semi-complex media [11.2]**										
M9 glucose + 3 a. a. + 0.2mM uracil	$7.34\cdot10^{-13}$	0.27	34475	$7.8\cdot10^{-5}$			896517	0.75	861	5.0
MOPS glycerol + 0.2mM uracil	$5.75\cdot10^{-13}$	0.14	16922	$4.9\cdot10^{-5}$			563987	0.82	582	3.7
	$6.56\cdot10^{-13}$	0.20	25151	$6.4\cdot10^{-5}$			506011	0.85	590	4.1
**MG1655 strain minimal media [11.3]**										
MOPS glycerol	$4.35\cdot10^{-}$	0.15	13914	$5.3\cdot10^{-}$	378352	0.0014	669365	0.74	508	5.7

	13			5						
	$4.60\cdot10^{-13}$	0.15	14557	$5.3\cdot10^{-5}$	249391	$9.0\cdot10^{-4}$	440439	0.84	655	3.6

11.1 Taken from Supplementary Tables 3.
11.2 Values of UC parameters were calculated from SSUCM-R using values of previously calculated M_u, experimentally determined t_C and t_D (Supplementary Table 3) in addition to standard values of cell parameters[3] and fitted value of k_{rs}.
11.3 Values of UC parameters were calculated from SSUCM-M using values of previously calculated M_u, experimentally determined t_C and t_D (Supplementary Table 3) in addition to standard values of cell parameters[3] and fitted value of k_{rs}.
11.4 The ratio of M_{rna} to M_{prot} for SSUCM-M and SSUCM-R were calculated according to the Supplementary Eqs. (7)-(8), respectively.
11.5 N_{enz} was calculated according to the Supplementary Eq. (9).
11.6 F_{etc} was calculated according to the Supplementary Eq. (117) of ref. [3].
11.7 The ratio of S_{lip} to S_{tot} was calculated according to the Supplementary Eqs. (122), (125) of ref. [3].
11.8 k_{dp} was calculated according to the Supplementary Eq. (114) of ref. [3].
11.9 C_{cell_comp} was calculated according to the Supplementary Eq. (24).

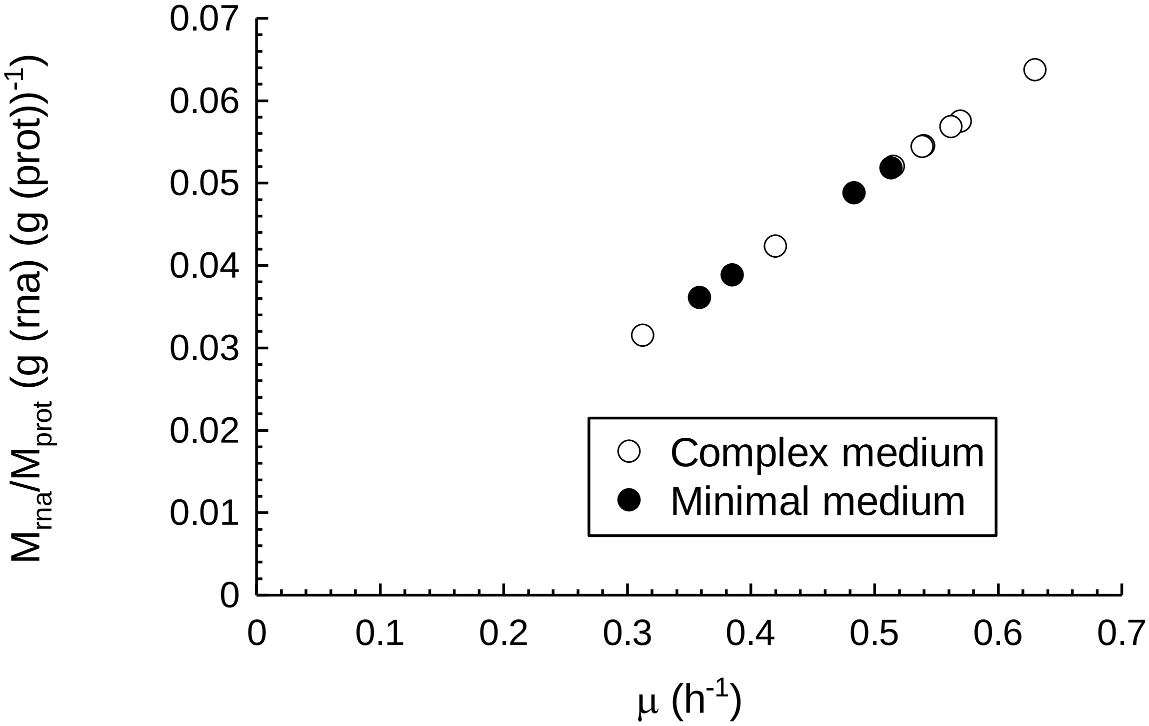

Supplementary Fig. 16. Ratio of RNA to proteins of unit cells. The dependence between values of calculated specific growth rate of the cell culture (μ, h^{-1}) and calculated ratio of RNA to protein (M_{rna}/M_{prot}, g (rna) (g (prot))$^{-1}$) from data of ref. [20] using SSUCM-M and SSUCM-R models (Supplementary Table 10).

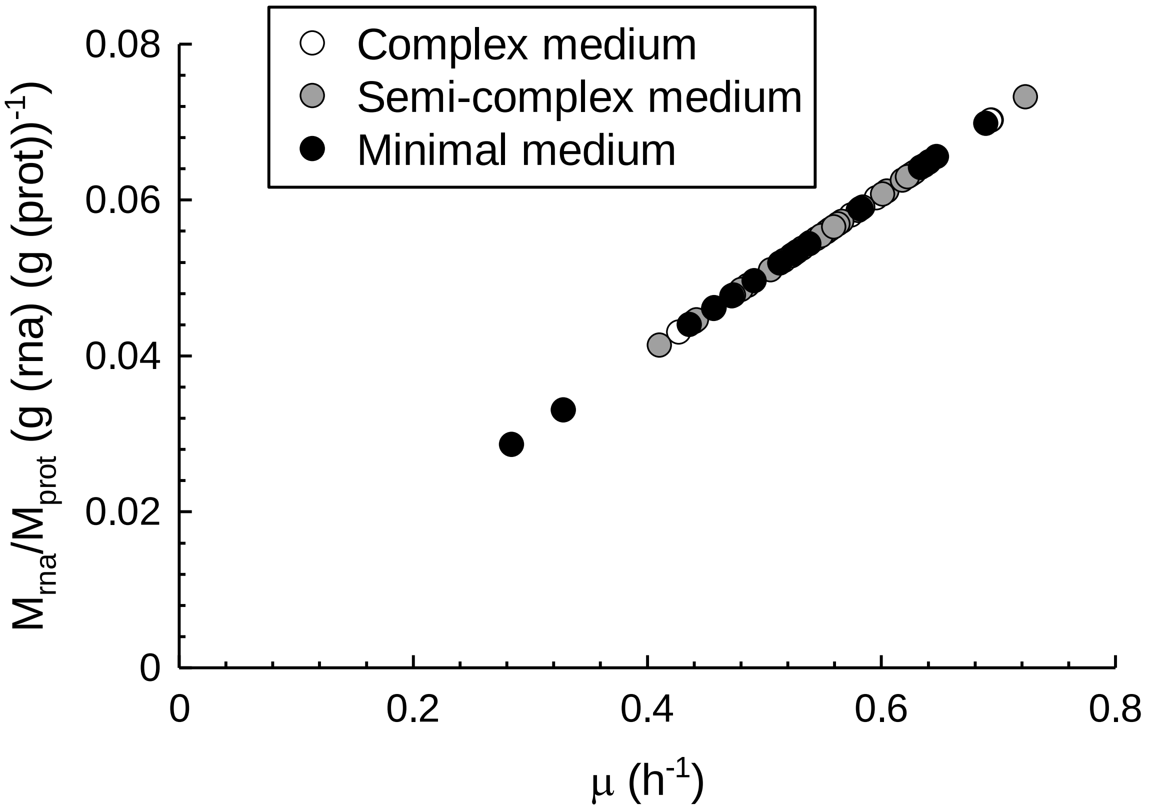

Supplementary Fig. 17. Ratio of RNA to proteins of unit cells. The dependence between values of calculated specific growth rate of the cell culture (μ, h^{-1}) and calculated ratio of RNA to protein (M_{rna}/M_{prot}, g (rna) (g (prot))$^{-1}$) from data of ref. [18] using SSUCM-M and SSUCM-R models (Supplementary Table 10).

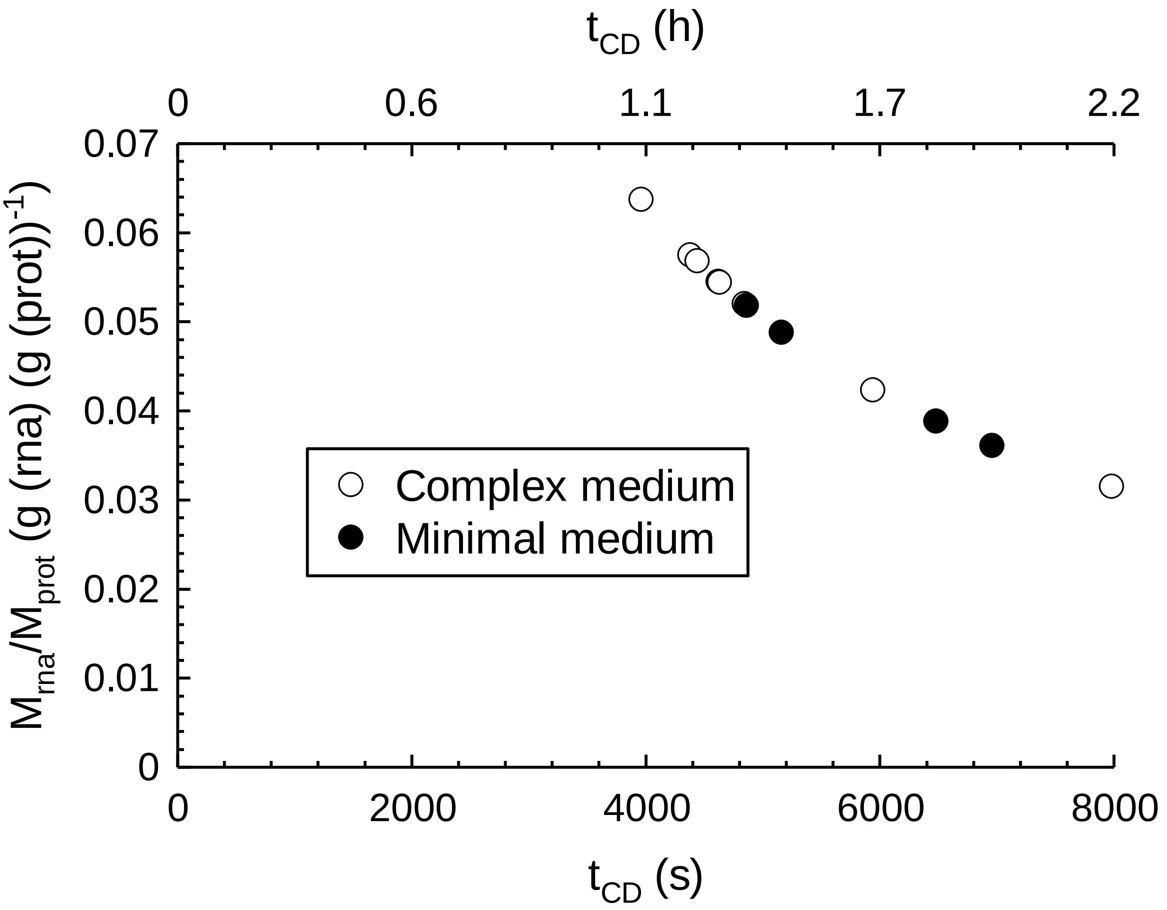

Supplementary Fig. 18. Ratio of RNA to proteins of unit cells. The dependence between values of experimentally determined cell cycle length of unit cell (t_{CD}, s and h) and calculated ratio of RNA to protein (M_{rna}/M_{prot}, g (rna) (g (prot))$^{-1}$) from data of ref. [20] using SSUCM-M and SSUCM-R models (Supplementary Table 10).

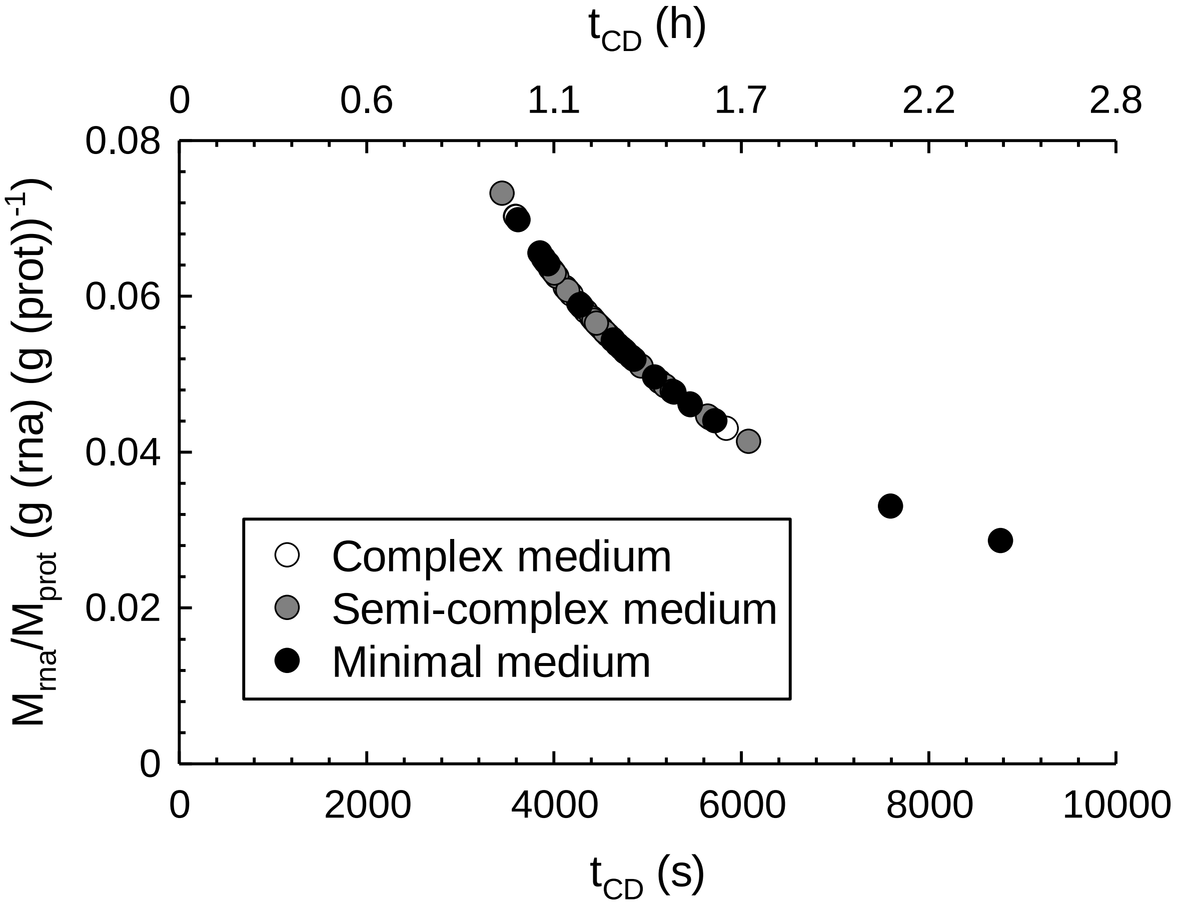

Supplementary Fig. 19. Ratio of RNA to proteins of unit cells. The dependence between values of experimentally determined cell cycle length of unit cell (t_{CD}, s and h) and calculated ratio of RNA to protein (M_{rna}/M_{prot}, g (rna) (g (prot))$^{-1}$) from data of ref. [18] using SSUCM-M and SSUCM-R models (Supplementary Table 10).

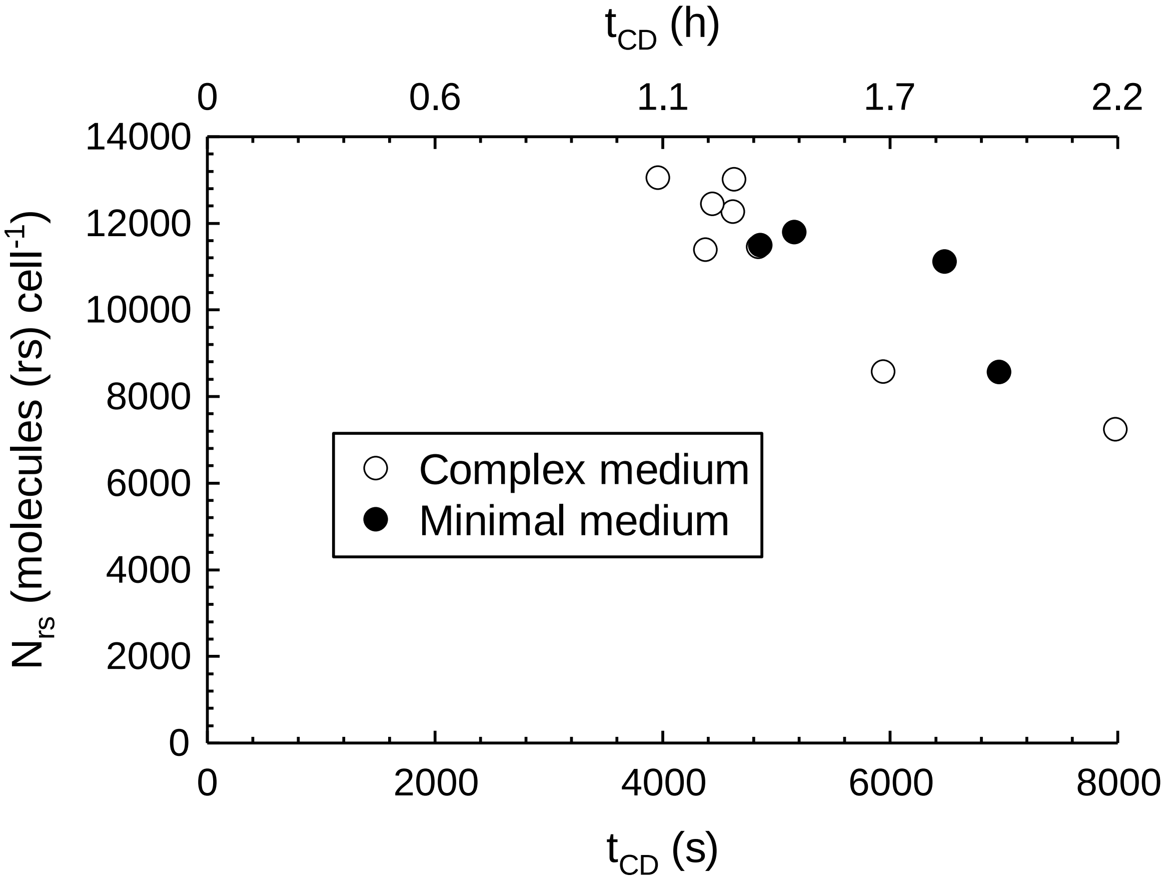

Supplementary Fig. 20. Number of ribosomes in unit cells. The dependence between values of experimentally determined cell cycle length of unit cell (t_{CD}, s and h) and calculated number of ribosomes in the cell (N_{rs}, molecules (rs) cell^{-1}) from data of ref. [20] using SSUCM-M and SSUCM-R models (Supplementary Table 10).

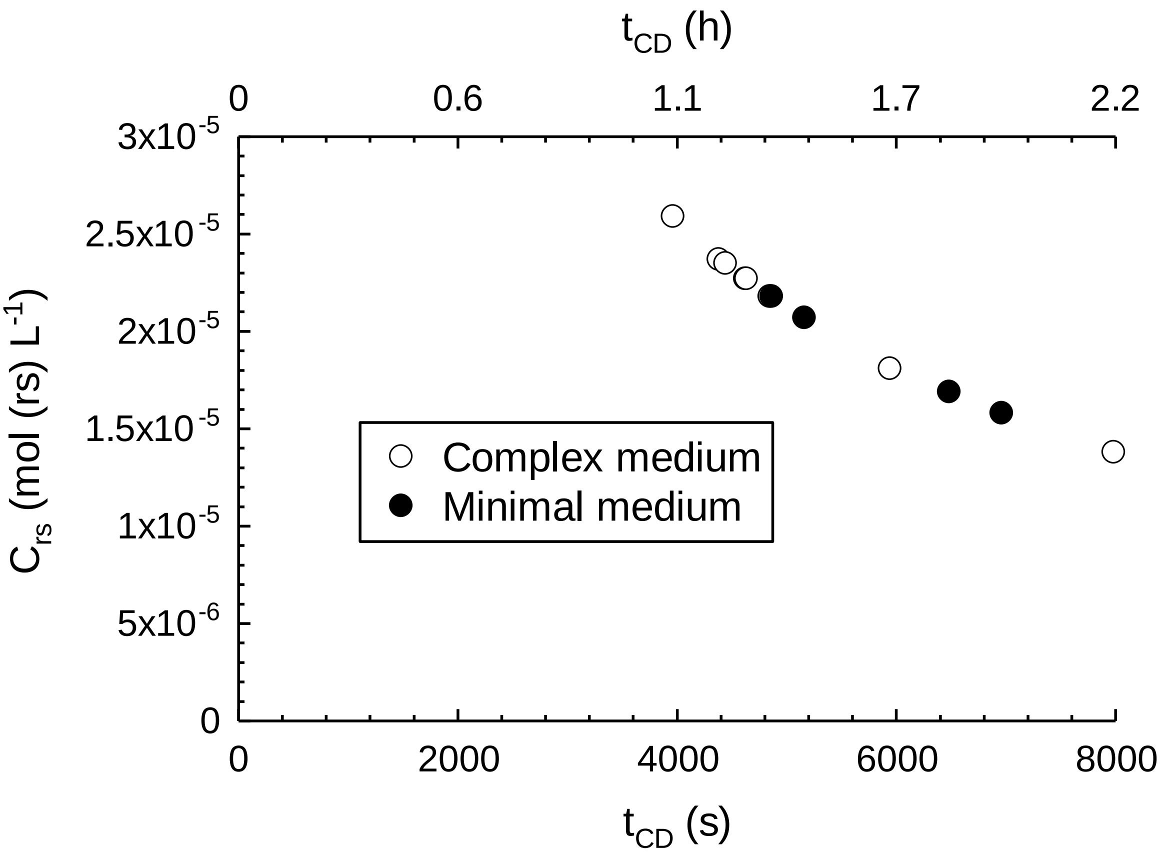

Supplementary Fig. 21. Concentration of ribosome in unit cells. The dependence between values of experimentally determined cell cycle length of unit cell (t_{CD}, s and h) and calculated molar concentration of ribosomes (C_{rs}, mol (rs) L^{-1}) from data of ref. [20] using SSUCM-M and SSUCM-R models (Supplementary Table 10).

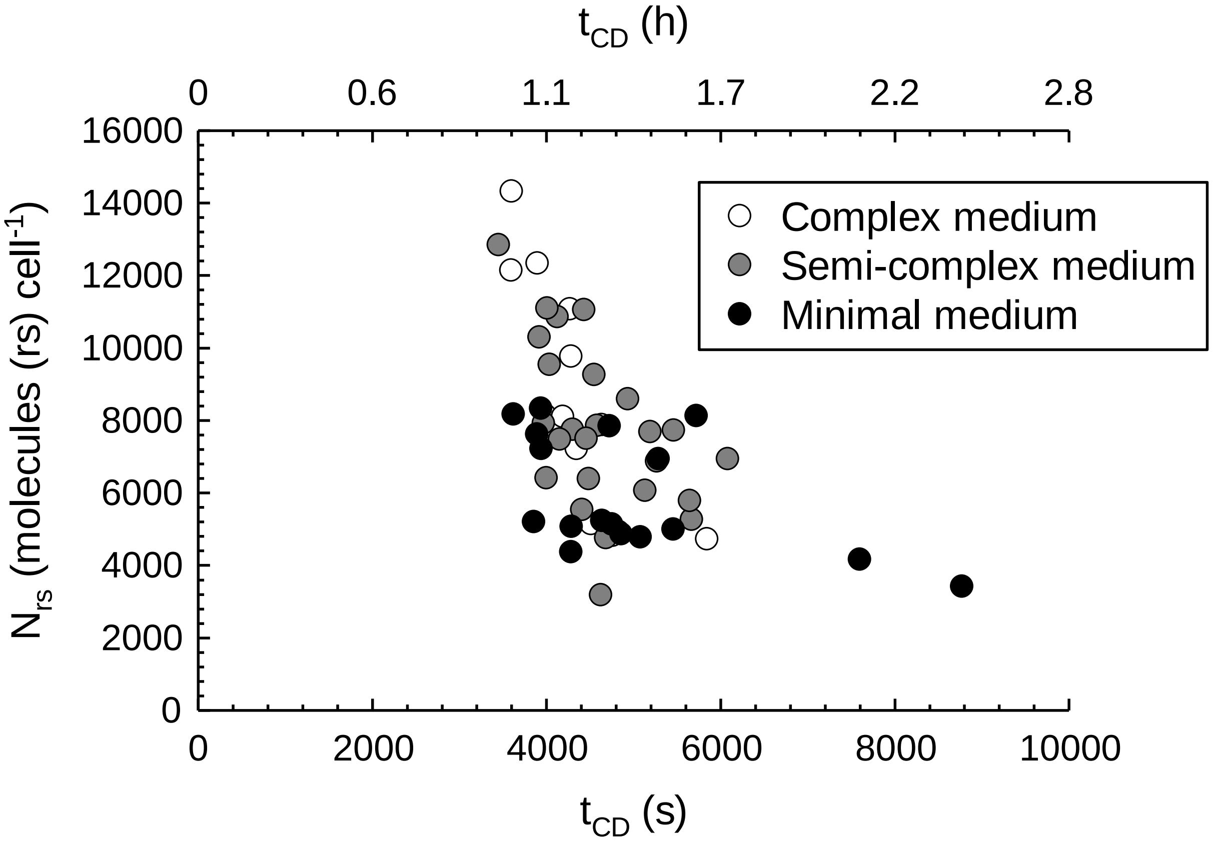

Supplementary Fig. 22. Number of ribosomes in unit cells. The dependence between values of experimentally determined cell cycle length of unit cell (t_{CD}, s and h) and calculated number of ribosomes in the cell (N_{rs}, molecules (rs) cell^{-1}) from data of ref. [18] using SSUCM-M and SSUCM-R models (Supplementary Table 10).

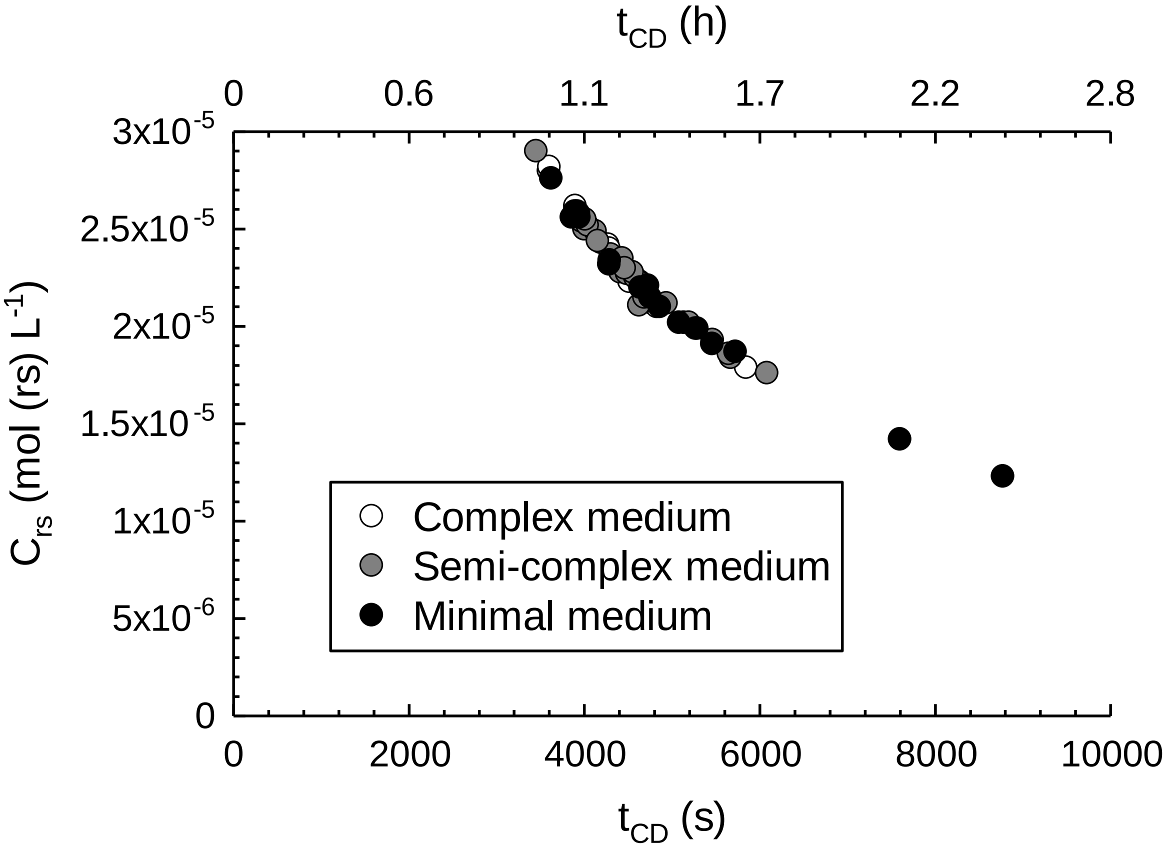

Supplementary Fig. 23. Concentration of ribosome in unit cells. The dependence between values of experimentally determined cell cycle length of unit cell (t_{CD}, s and h) and calculated molar concentration of ribosomes (C_{rs}, mol (rs) L^{-1}) from data of ref. [18] using SSUCM-M and SSUCM-R models (Supplementary Table 10).

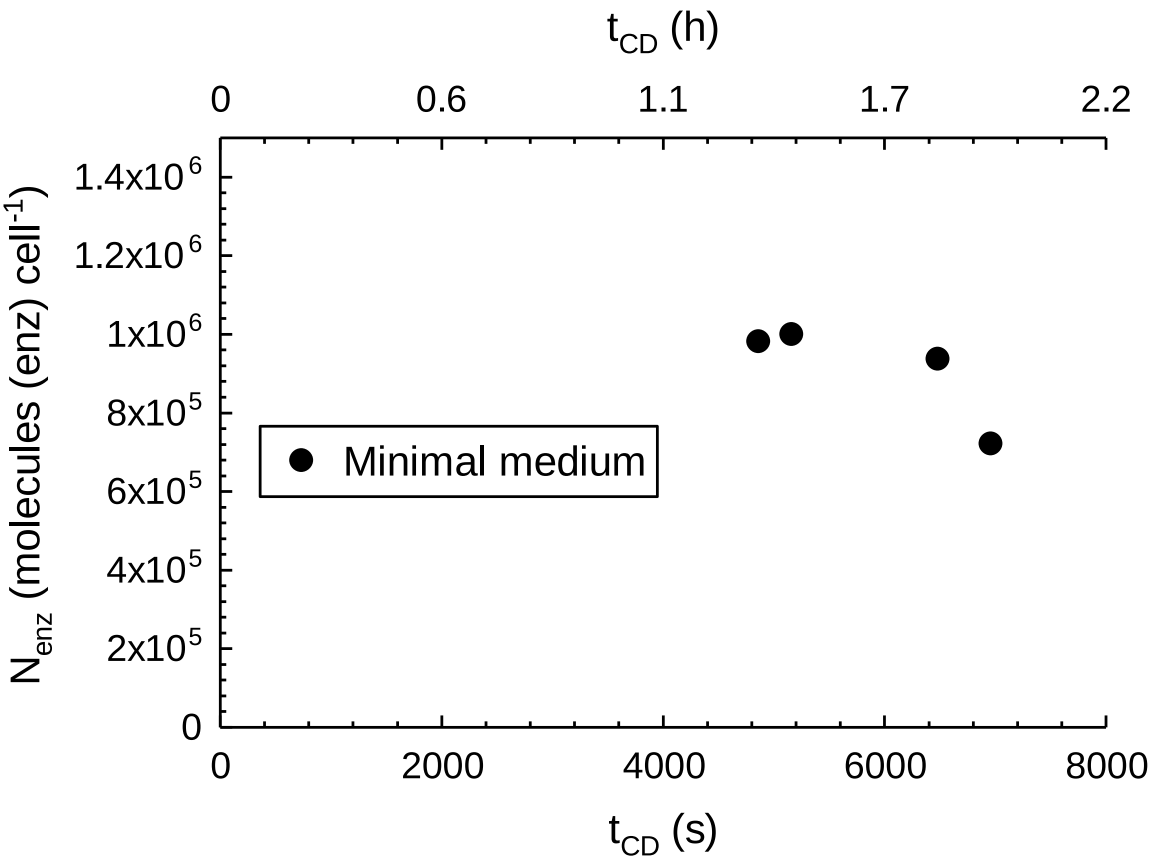

Supplementary Fig. 24. Number of enzymes in unit cells. The dependence between values of experimentally determined cell cycle length of unit cell (t_{CD}, s and h) and calculated number of enzymes in the cell (N_{enz}, molecules (enz) cell^{-1}) from data of ref. [20] using SSUCM-M and SSUCM-R models (Supplementary Table 10).

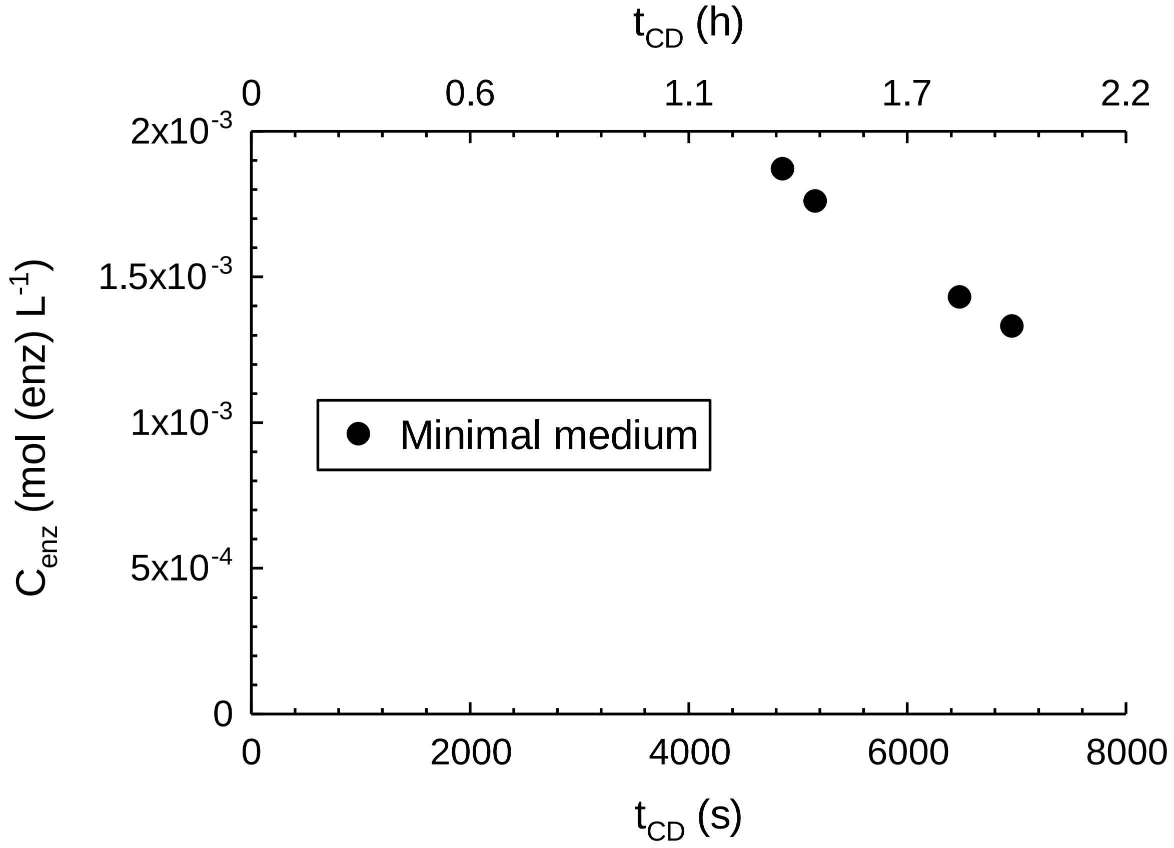

Supplementary Fig. 25. Concentration of enzymes in unit cells. The dependence between values of experimentally determined cell cycle length of unit cell (t_{CD}, s and h) and calculated molar concentration of enzymes (C_{enz}, mol (enz) L^{-1}) from data of ref. [20] using SSUCM-M and SSUCM-R models (Supplementary Table 10).

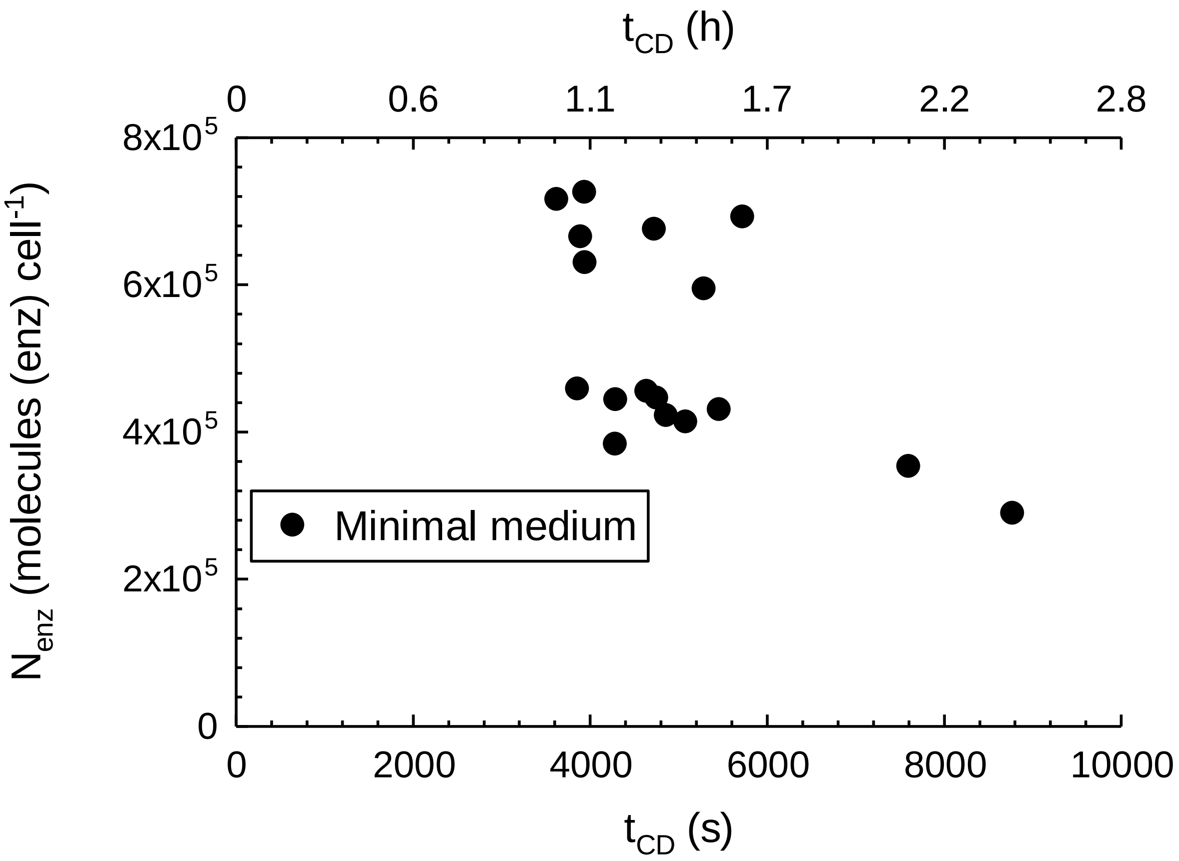

Supplementary Fig. 26. Number of enzymes in unit cells. The dependence between values of experimentally determined cell cycle length of unit cell (t_{CD}, s and h) and calculated number of enzymes in the cell (N_{enz}, molecules (enz) cell^{-1}) from data of ref. [18] using SSUCM-M and SSUCM-R models (Supplementary Table 10).

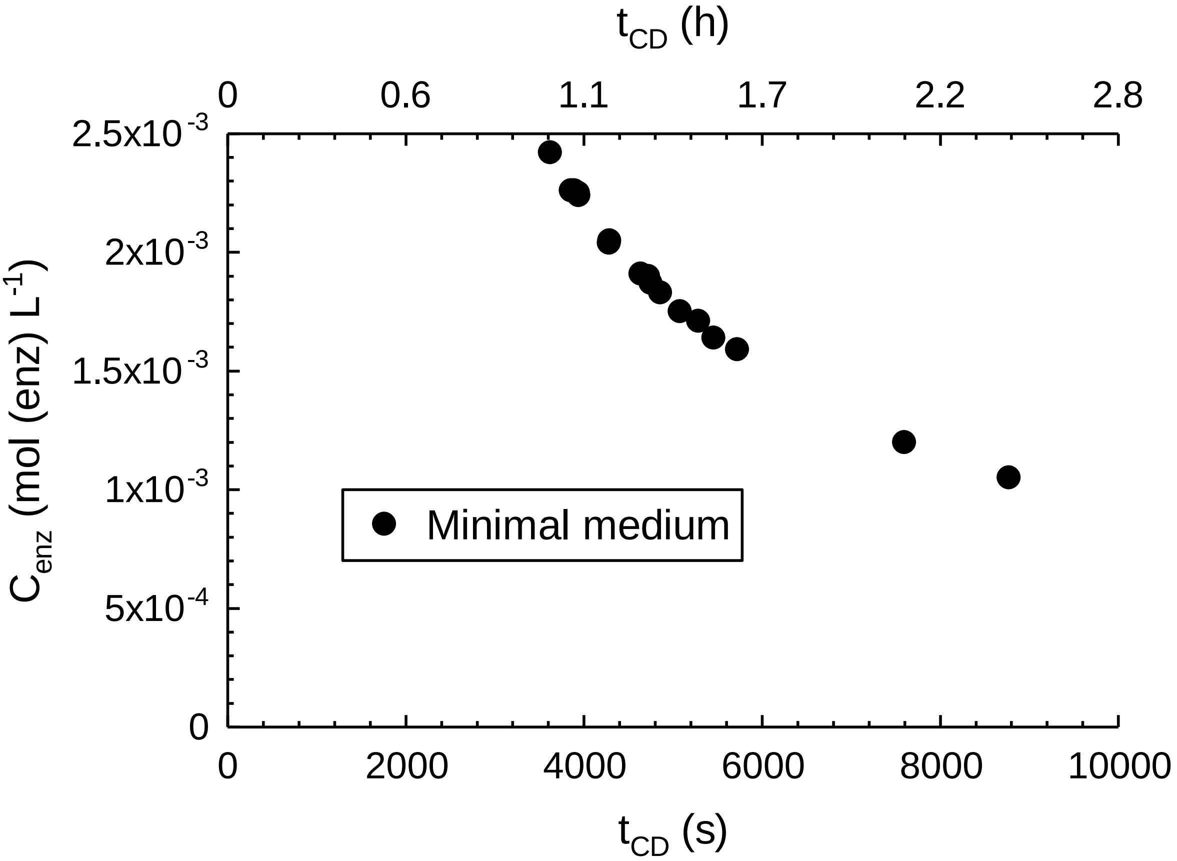

Supplementary Fig. 27. Concentration of enzymes in unit cells. The dependence between values of experimentally determined cell cycle length of unit cell (t_{CD}, s and h) and calculated total molar concentration of enzymes (C_{enz}, mole (enz) L^{-1}) from data of ref. [18] using SSUCM-M and SSUCM-R models (Supplementary Table 10).

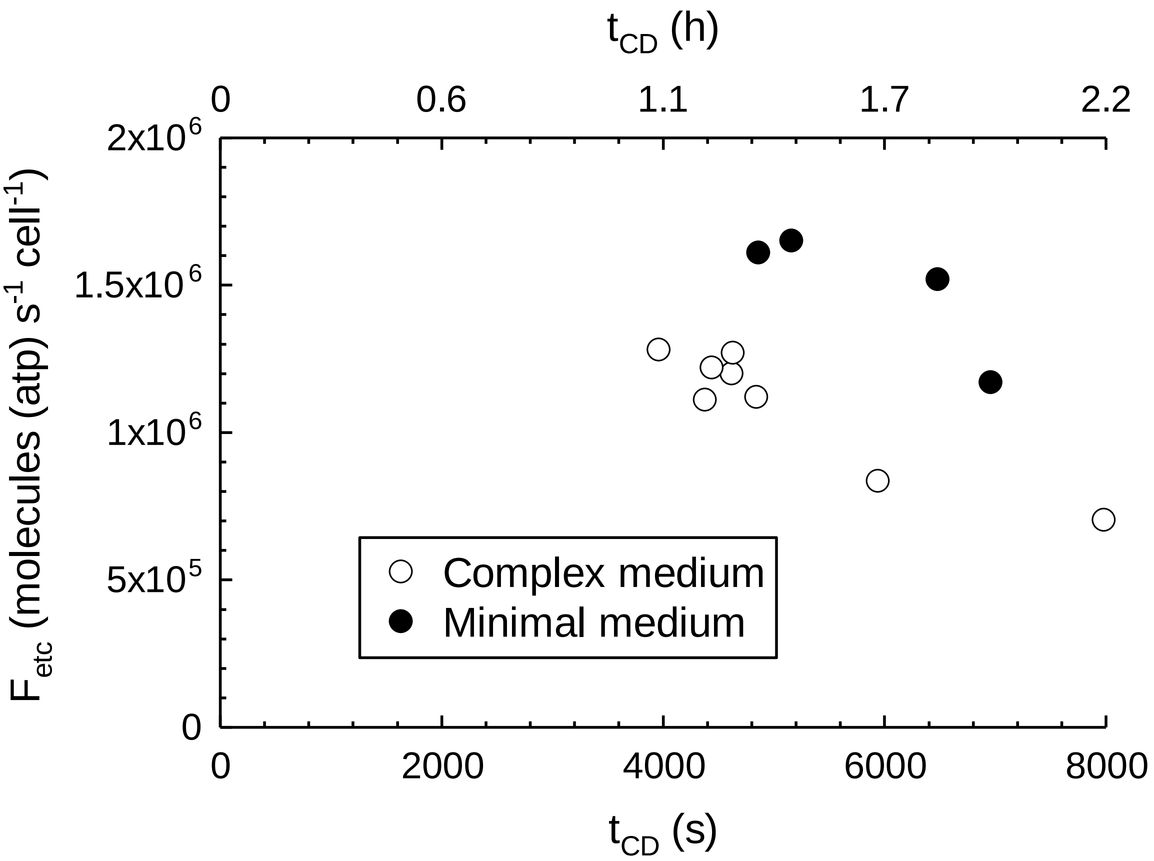

Supplementary Fig. 28. Energy flux in unit cells. The dependence between values of experimentally determined cell cycle length of unit cell (t_{CD}, s and h) and calculated ATP synthesis flux of electron transport chain complex (F_{etc}, molecules (atp) s^{-1} $cell^{-1}$) from data of ref. [20] using SSUCM-M and SSUCM-R models (Supplementary Table 10).

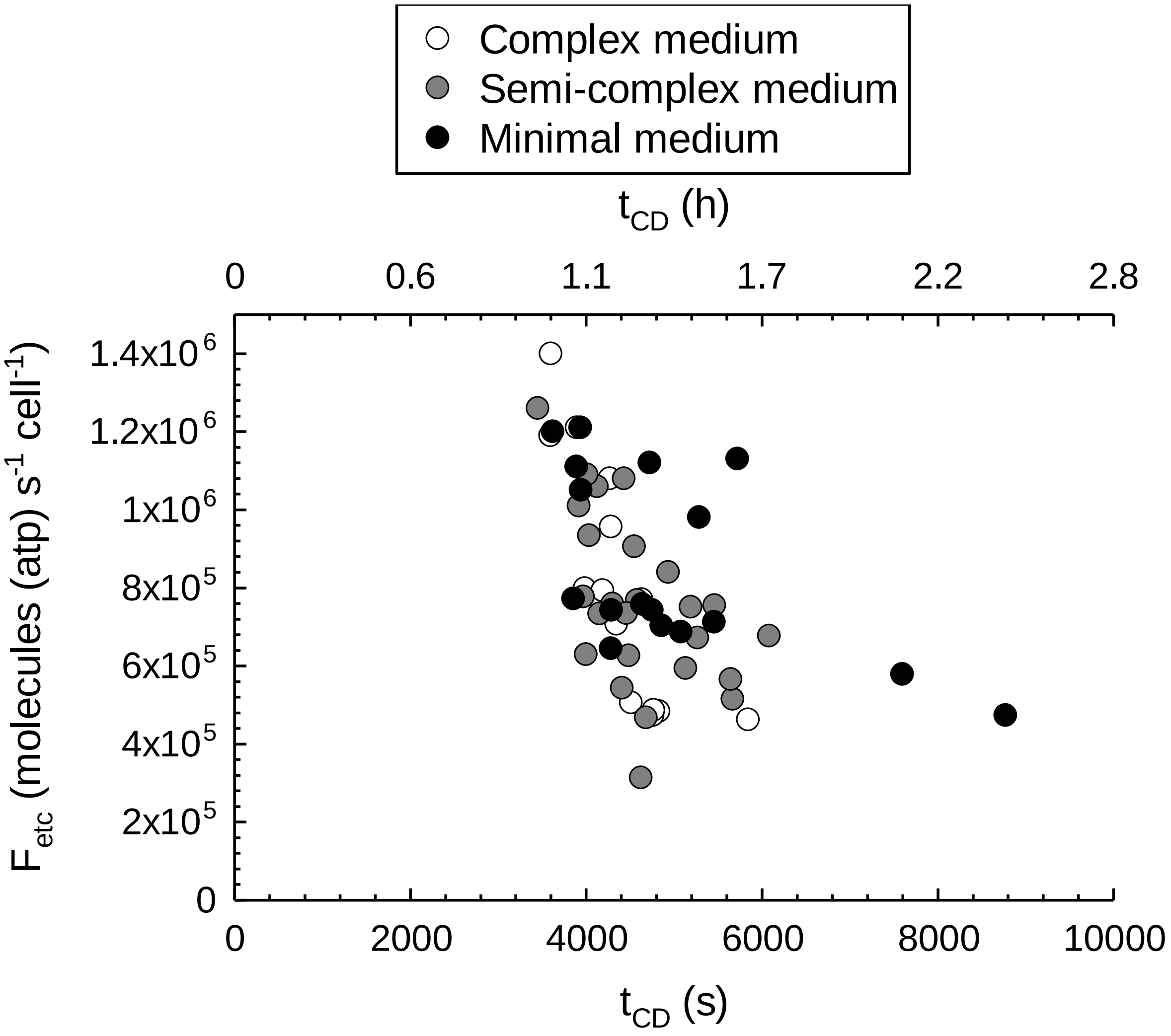

Supplementary Fig. 29. Energy flux in unit cells. The dependence between values of experimentally determined cell cycle length of unit cell (t_{CD}, s and h) and calculated ATP synthesis flux of electron transport chain complex (F_{etc}, molecules (atp) s^{-1} $cell^{-1}$) from data of ref. [18] using SSUCM-M and SSUCM-R models (Supplementary Table 10).

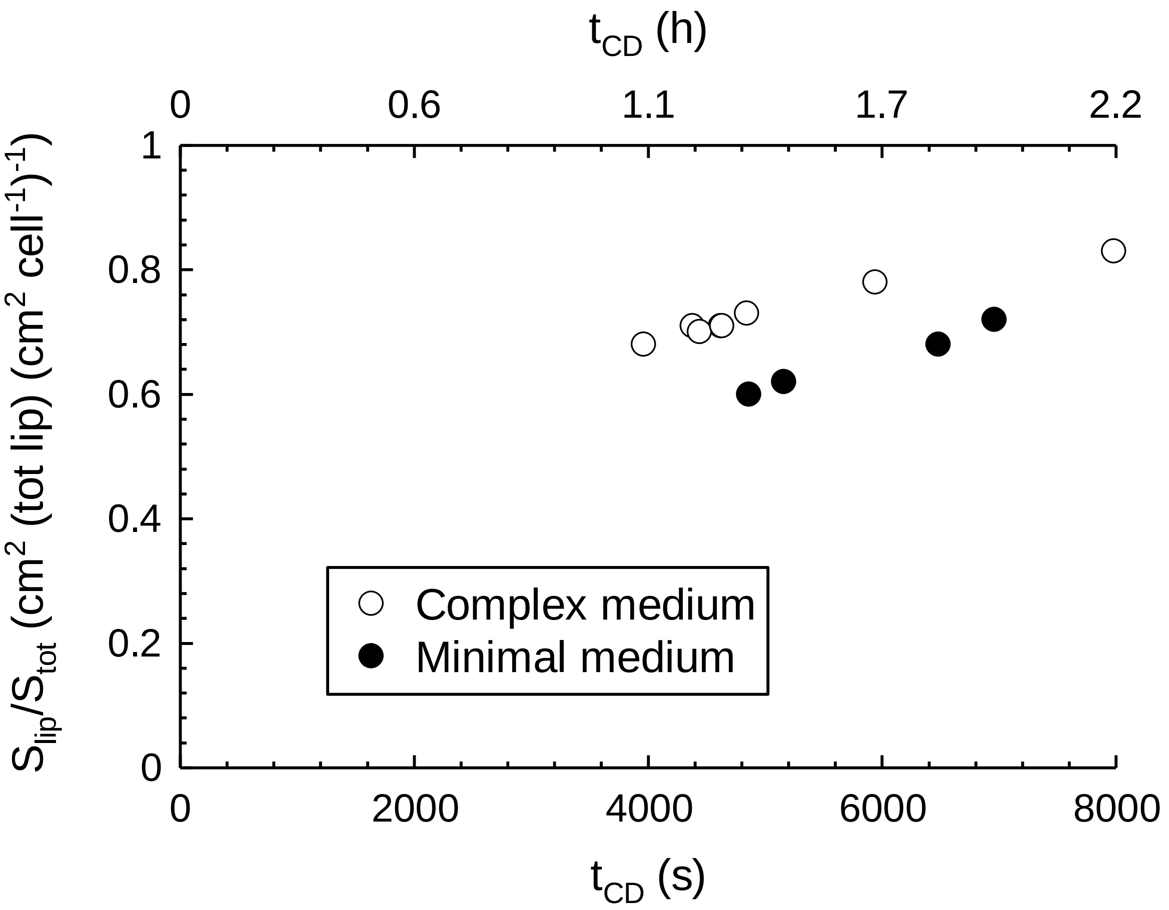

Supplementary Fig. 30. Membrane coverage in unit cells. The dependence between values of experimentally determined cell cycle length of unit cell (t_{CD}, s and h) and calculated ratio of membrane surface covered by lipids to the cell surface (S_{lip}/S_{tot}, cm^2 (tot lip) ((cm^2 cell^{-1})$^{-1}$) from data of ref. [20] using SSUCM-M and SSUCM-R models (Supplementary Table 10).

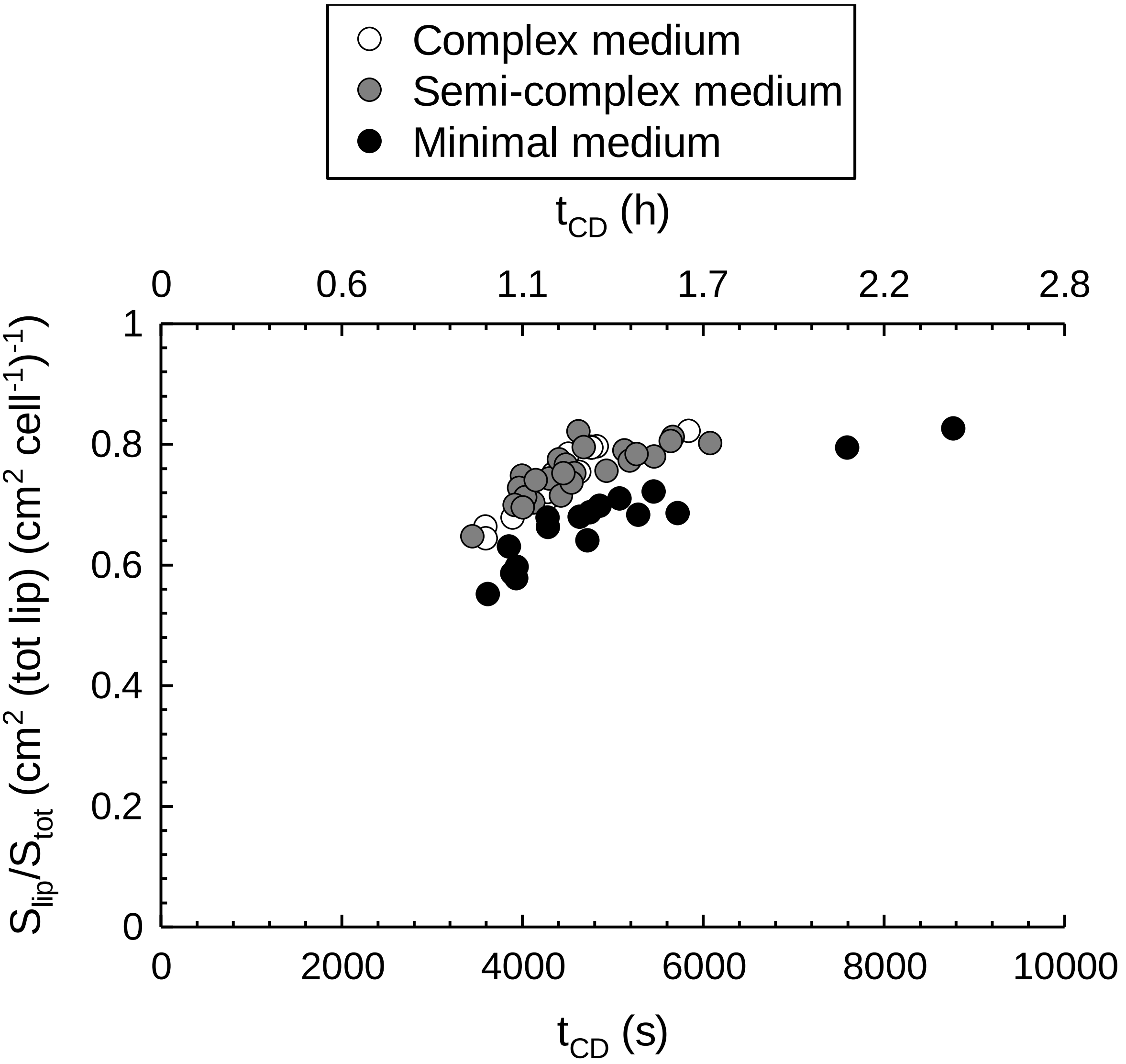

Supplementary Fig. 31. Membrane coverage in unit cells. The dependence between values of experimentally determined cell cycle length of unit cell (t_{CD}, s and h) and calculated ratio of membrane surface covered by lipids to the cell surface (S_{lip}/S_{tot}, cm^2 (tot lip) ((cm^2 $cell^{-1}$)$^{-1}$) from data of ref. [18] using SSUCM-M and SSUCM-R models (Supplementary Table 10).

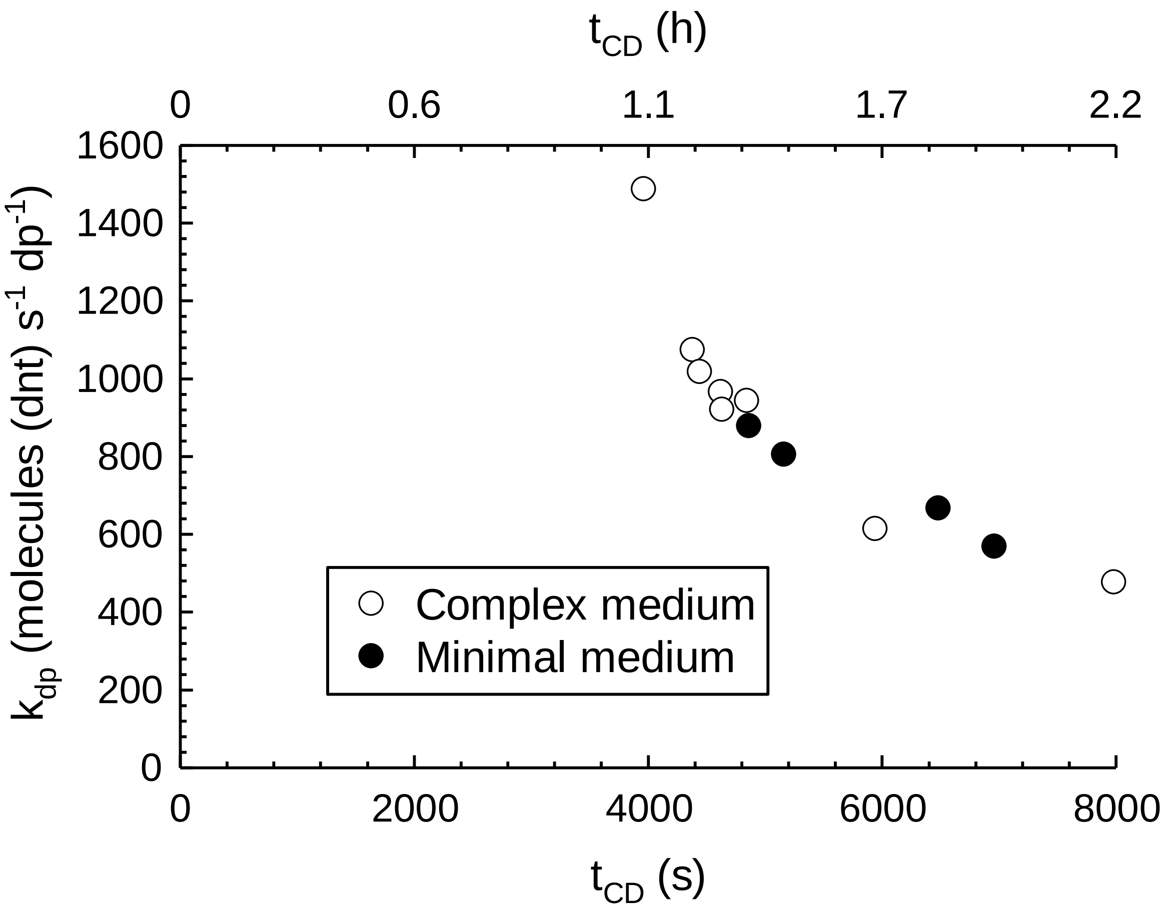

Supplementary Fig. 32. DNA polymerase velocity in unit cells. The dependence between values of experimentally determined cell cycle length of unit cell (t_{CD}, s and h) and calculated apparent working rate of DNA polymerase (k_{dp}, molecules (dnt) s^{-1} dp^{-1}) from data of ref. [20] using SSUCM-M and SSUCM-R models (Supplementary Table 10).

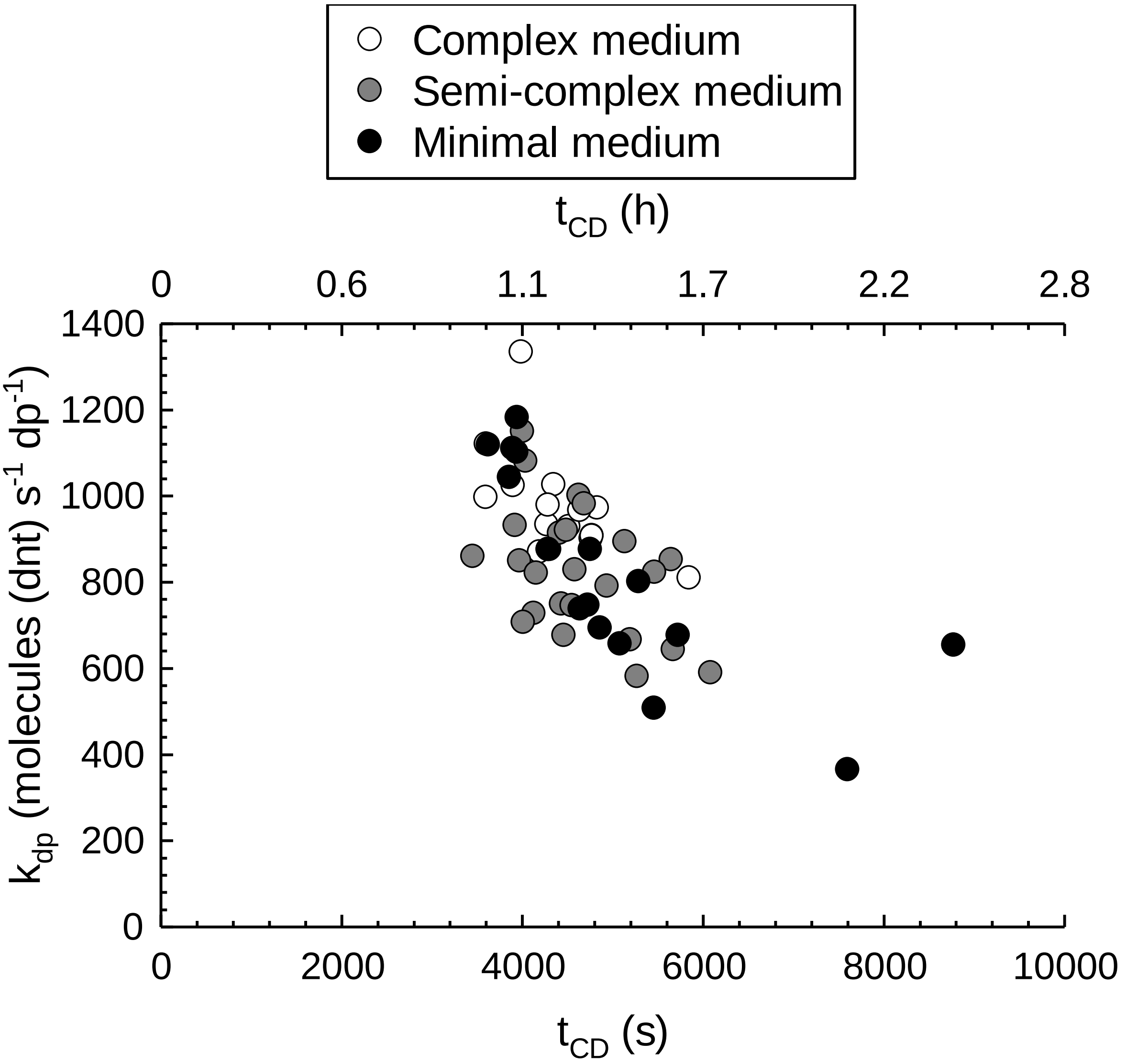

Supplementary Fig. 33. DNA polymerase velocity in unit cells. The dependence between values of experimentally determined cell cycle length of unit cell (t_{CD}, s and h) and calculated apparent working rate of DNA polymerase (k_{dp}, molecules (dnt) s^{-1} dp^{-1}) from data of ref. [18] using SSUCM-M and SSUCM-R models (Supplementary Table 10).

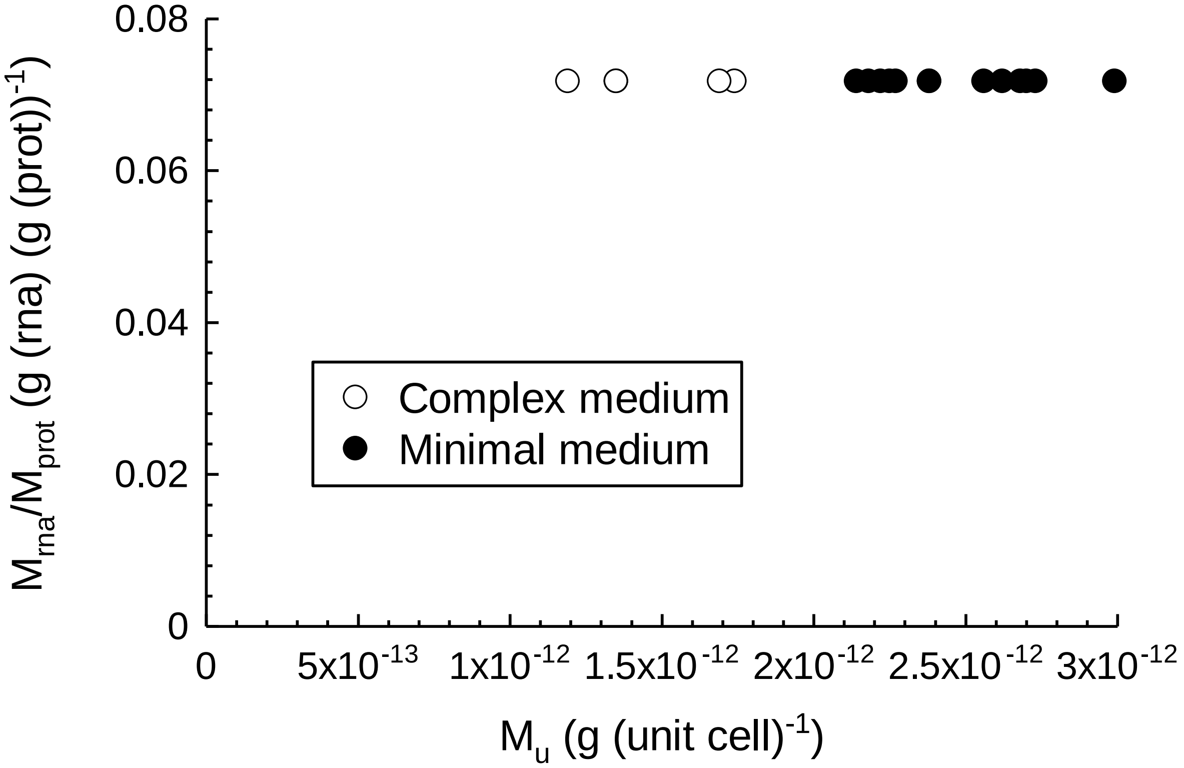

Supplementary Fig. 34. Ratio of RNA to proteins of unit cells. The dependence between calculated values of unit cell mass (M_u, g (unit cell)$^{-1}$) and ratio of RNA to protein (M_{rna}/M_{prot}, g (rna) (g (prot))$^{-1}$) from data of ref. [14] using SSUCM-M and SSUCM-R models (Supplementary Table 10).

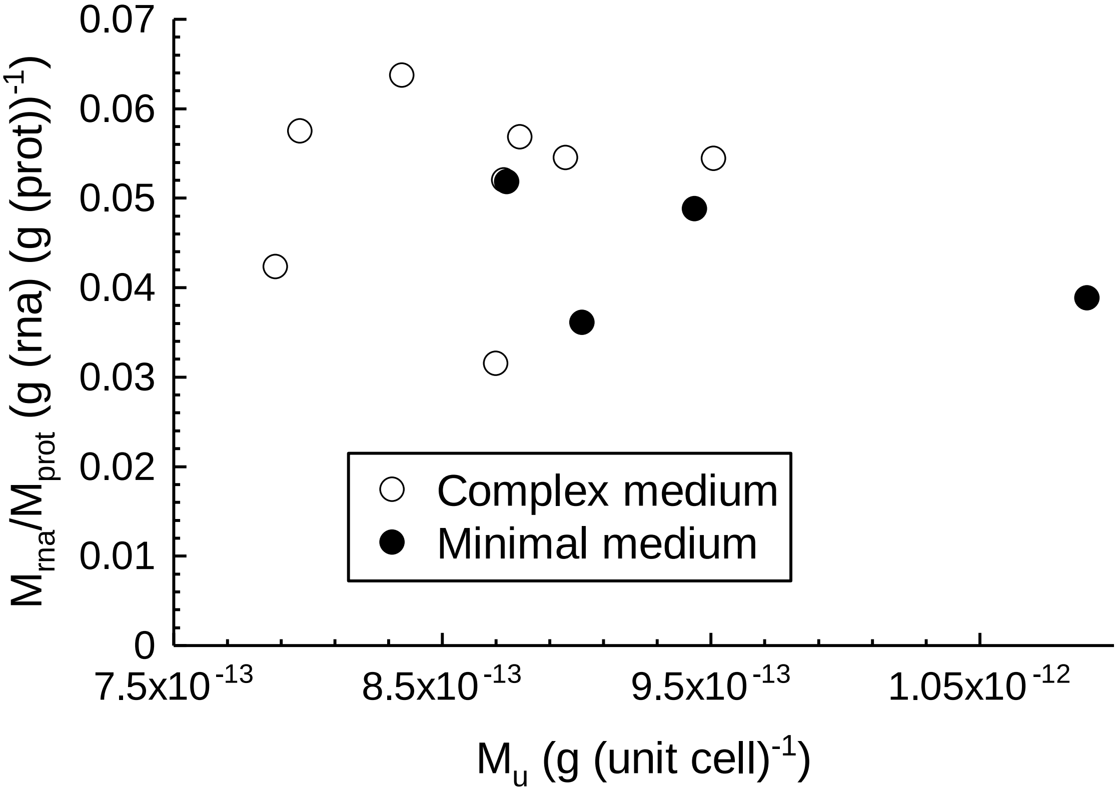

Supplementary Fig. 35. Ratio of RNA to proteins of unit cells. The dependence between calculated values of unit cell mass (M_u, g (unit cell)$^{-1}$) and ratio of RNA to protein (M_{rna}/M_{prot}, g (rna) (g (prot))$^{-1}$) from data of ref. [20] using SSUCM-M and SSUCM-R models (Supplementary Table 10).

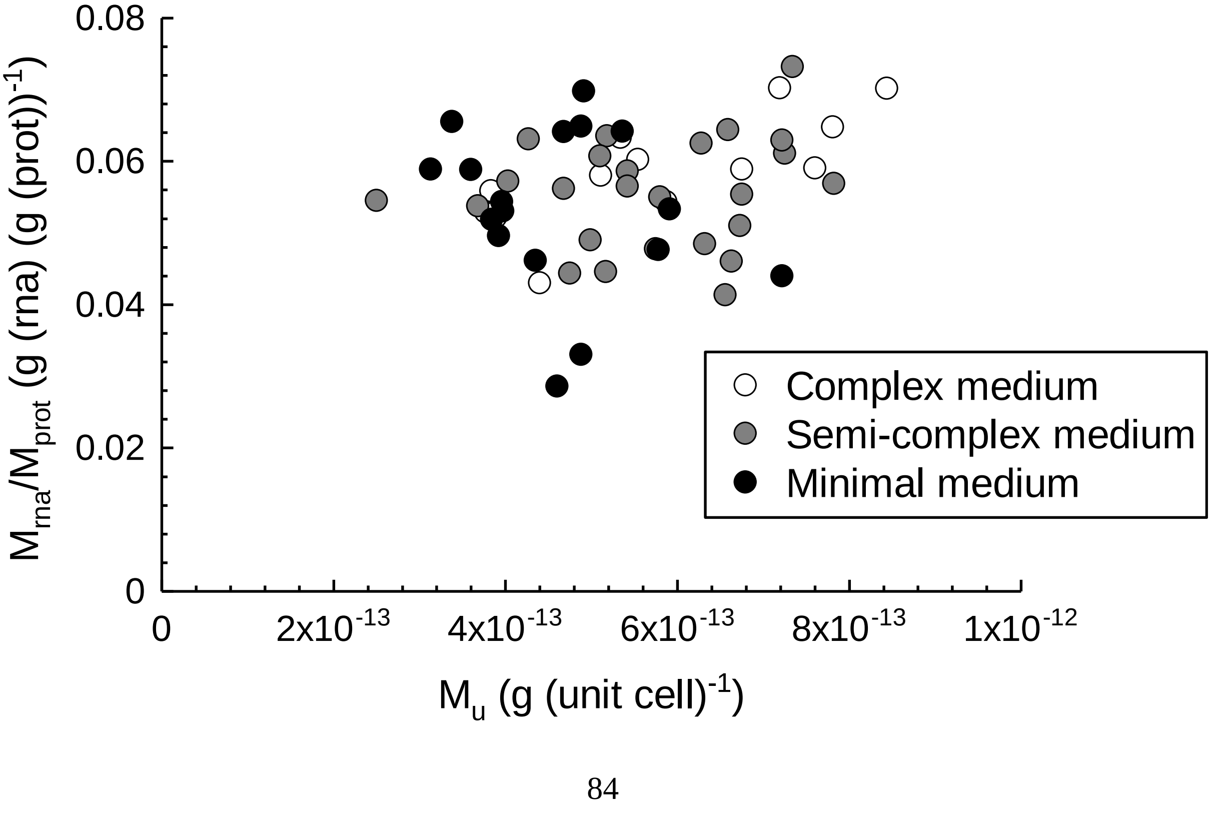

Supplementary Fig. 36. Ratio of RNA to proteins of unit cells. The dependence between calculated values of unit cell mass (M_u, g (unit cell)$^{-1}$) and ratio of RNA to protein (M_{rna}/M_{prot}, g (rna) (g (prot))$^{-1}$) from data of ref. [18] using SSUCM-M and SSUCM-R models (Supplementary Table 10).

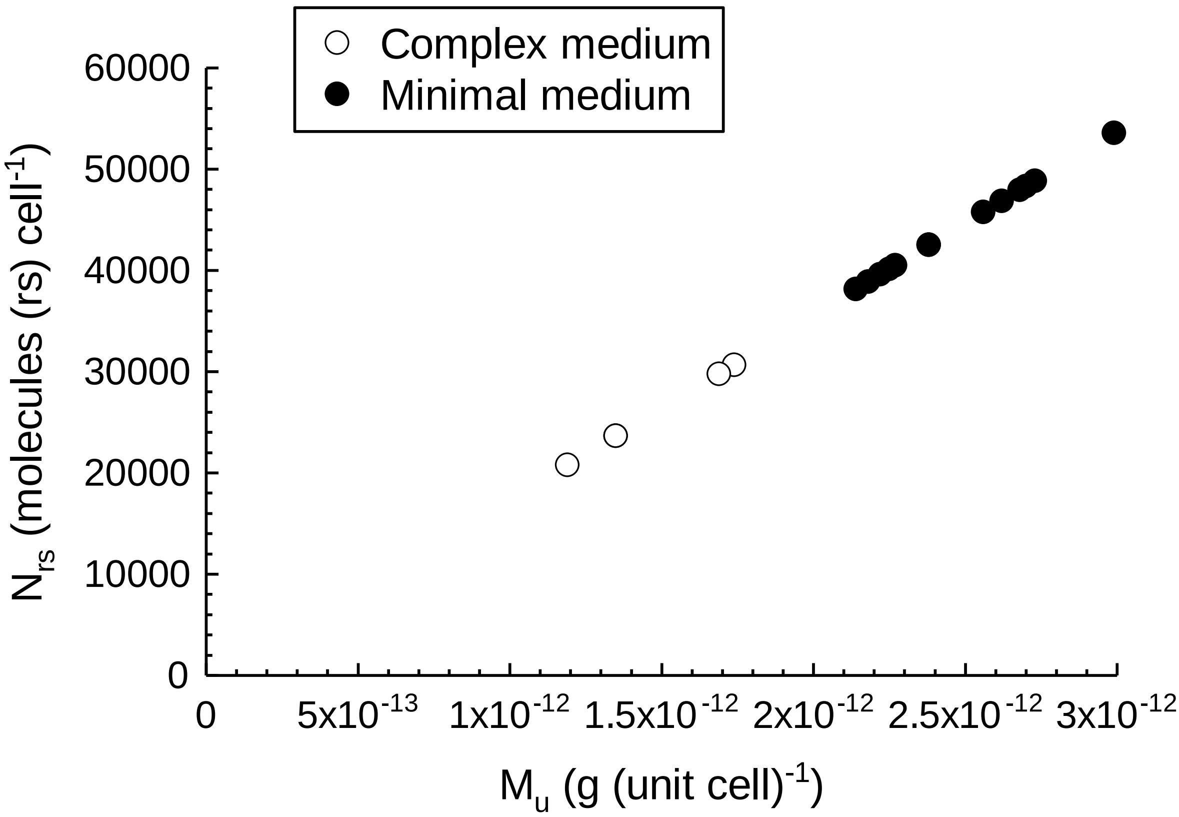

Supplementary Fig. 37. Number of ribosomes in unit cells. The dependence between calculated values of unit cell mass (M_u, g (unit cell)$^{-1}$) and number of ribosomes in the cell (N_{rs}, molecules (rs) cell^{-1}) from data of ref. [14] using SSUCM-M and SSUCM-R models (Supplementary Table 10).

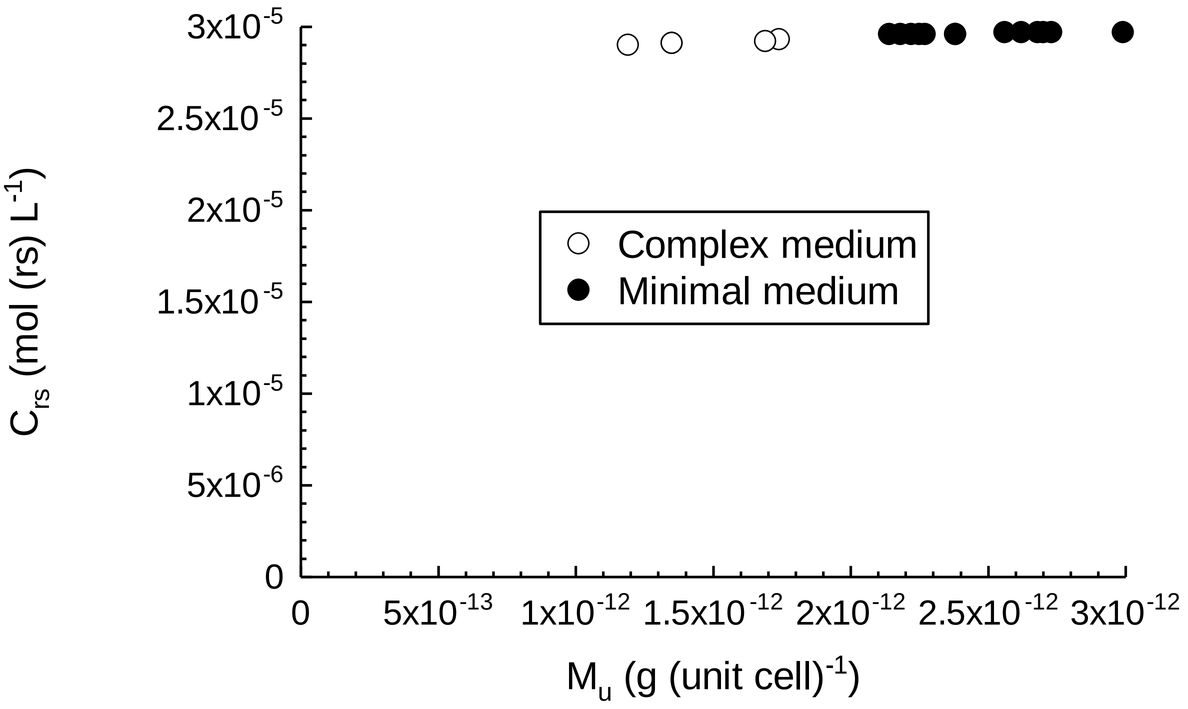

Supplementary Fig. 38. Concentration of ribosome in unit cells. The dependence between calculated values of unit cell mass (M_u, g (unit cell)$^{-1}$) and molar concentration of ribosomes (C_{rs}, mol (rs) L^{-1}) from data of ref. [14] using SSUCM-M and SSUCM-R models (Supplementary Table 10).

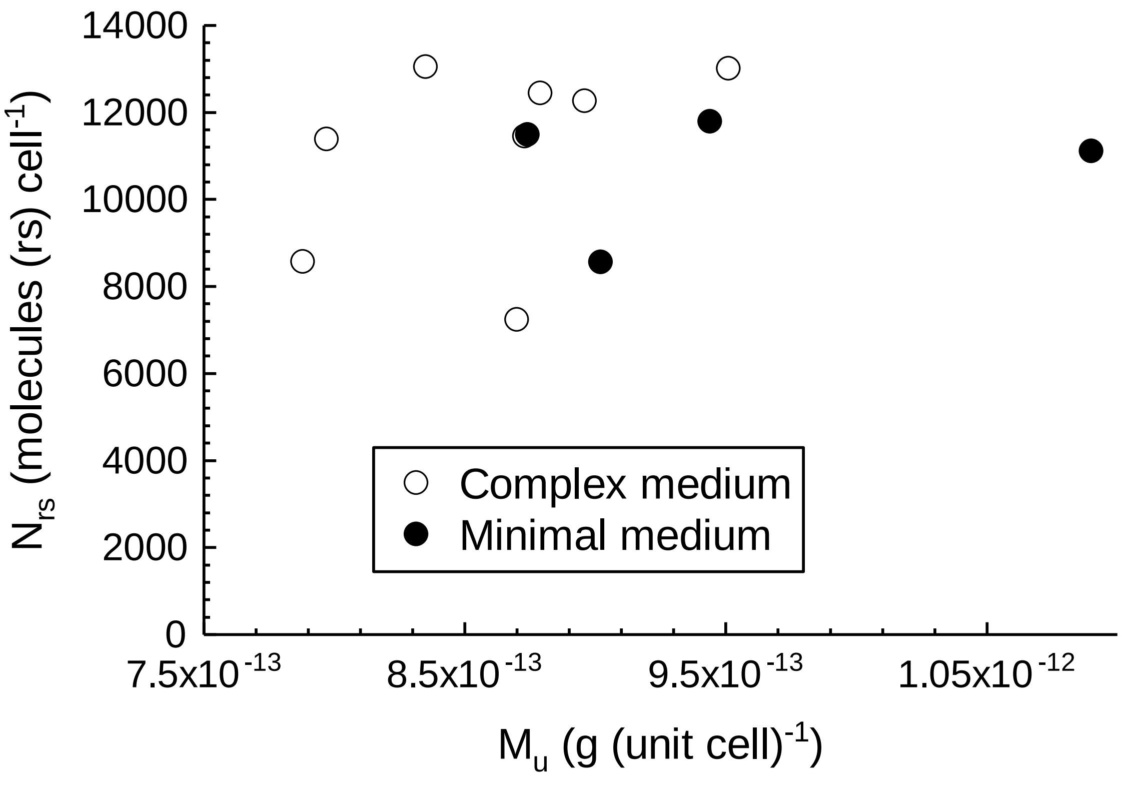

Supplementary Fig. 39. Number of ribosomes in unit cells. The dependence between calculated values of unit cell mass (M_u, g (unit cell)$^{-1}$) and number of ribosomes in the cell (N_{rs}, molecules (rs) cell^{-1}) from data of ref. [20] using SSUCM-M and SSUCM-R models (Supplementary Table 10).

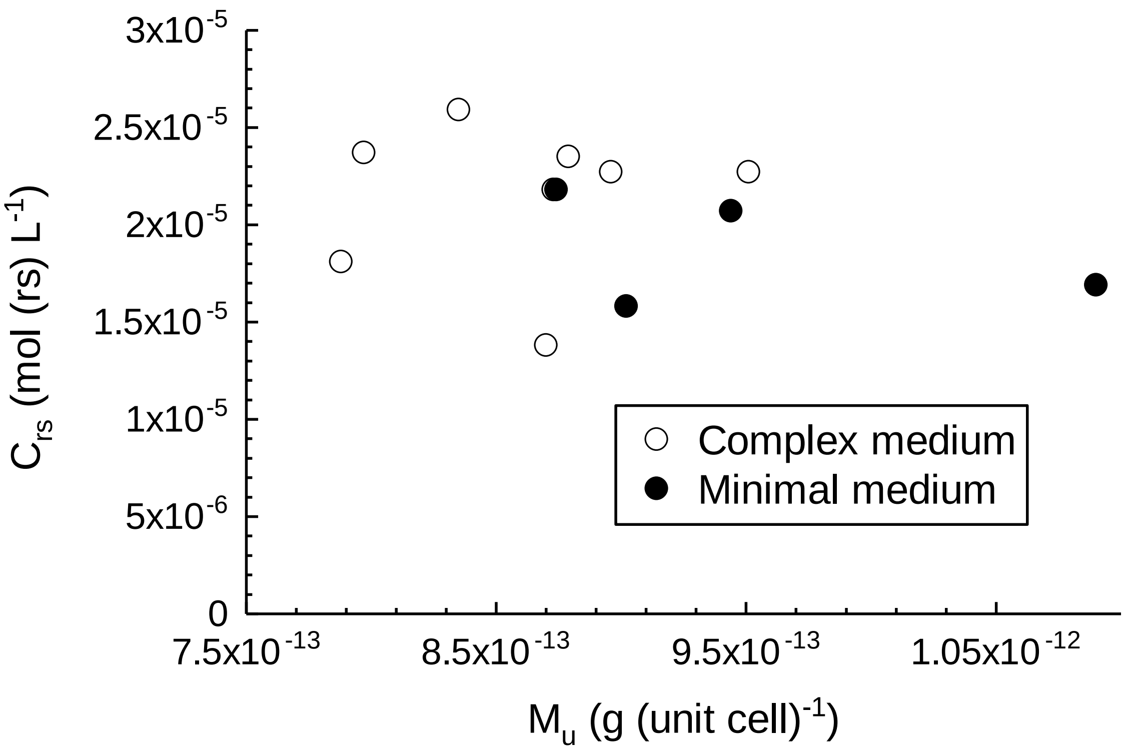

Supplementary Fig. 40. Concentration of ribosome in unit cells. The dependence between calculated values of unit cell mass (M_u, g (unit cell)$^{-1}$) and molar concentration of ribosomes (C_{rs}, mol (rs) L^{-1}) from data of ref. [20] using SSUCM-M and SSUCM-R models (Supplementary Table 10).

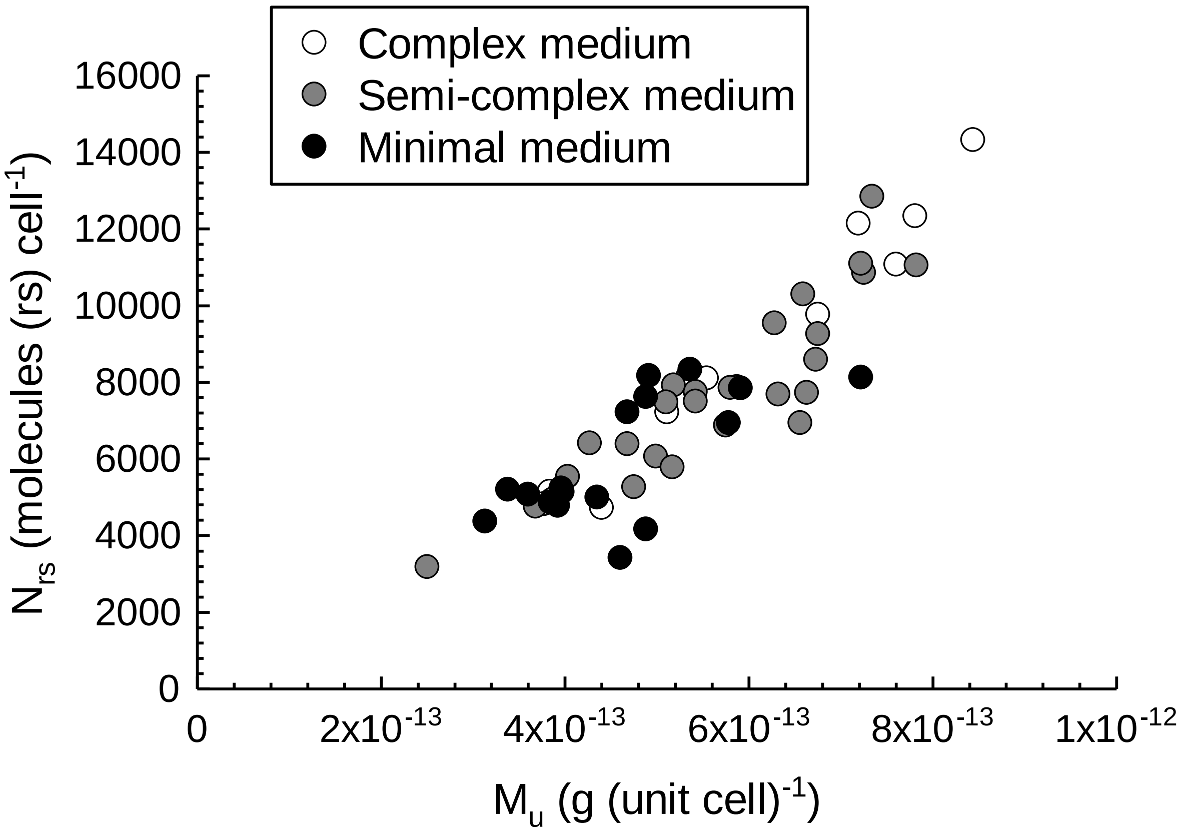

Supplementary Fig. 41. Number of ribosomes in unit cells. The dependence between calculated values of unit cell mass (M_u, g (unit cell)$^{-1}$) and number of ribosomes in the cell (N_{rs}, molecules (rs) cell^{-1}) from data of ref. [18] using SSUCM-M and SSUCM-R models (Supplementary Table 10).

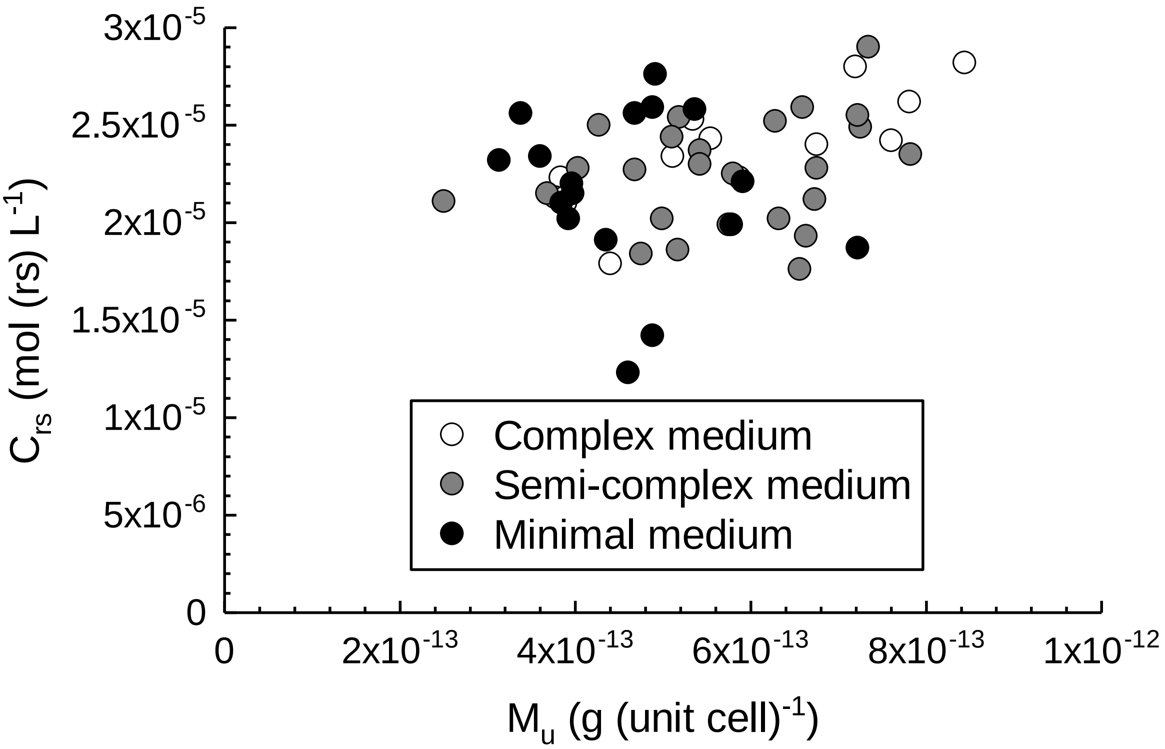

Supplementary Fig. 42. Concentration of ribosome in unit cells. The dependence between calculated values of unit cell mass (M_u, g (unit cell)$^{-1}$) and molar concentration of ribosomes (C_{rs}, mol (rs) L^{-1}) from data of ref. [18] using SSUCM-M and SSUCM-R models (Supplementary Table 10).

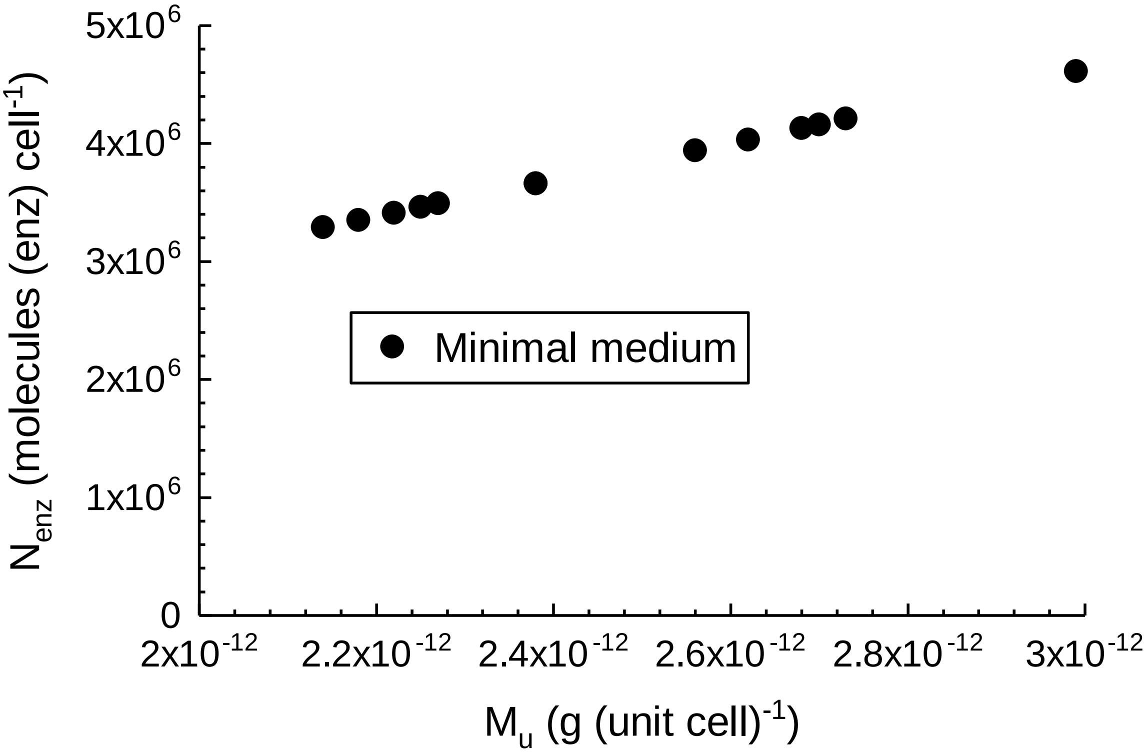

Supplementary Fig. 43. Number of enzymes in unit cells. The dependence between calculated values of unit cell mass (M_u, g (unit cell)$^{-1}$) and number of enzymes in the cell (N_{enz}, molecules (enz) cell^{-1}) from data of ref. [14] using SSUCM-M and SSUCM-R models (Supplementary Table 10).

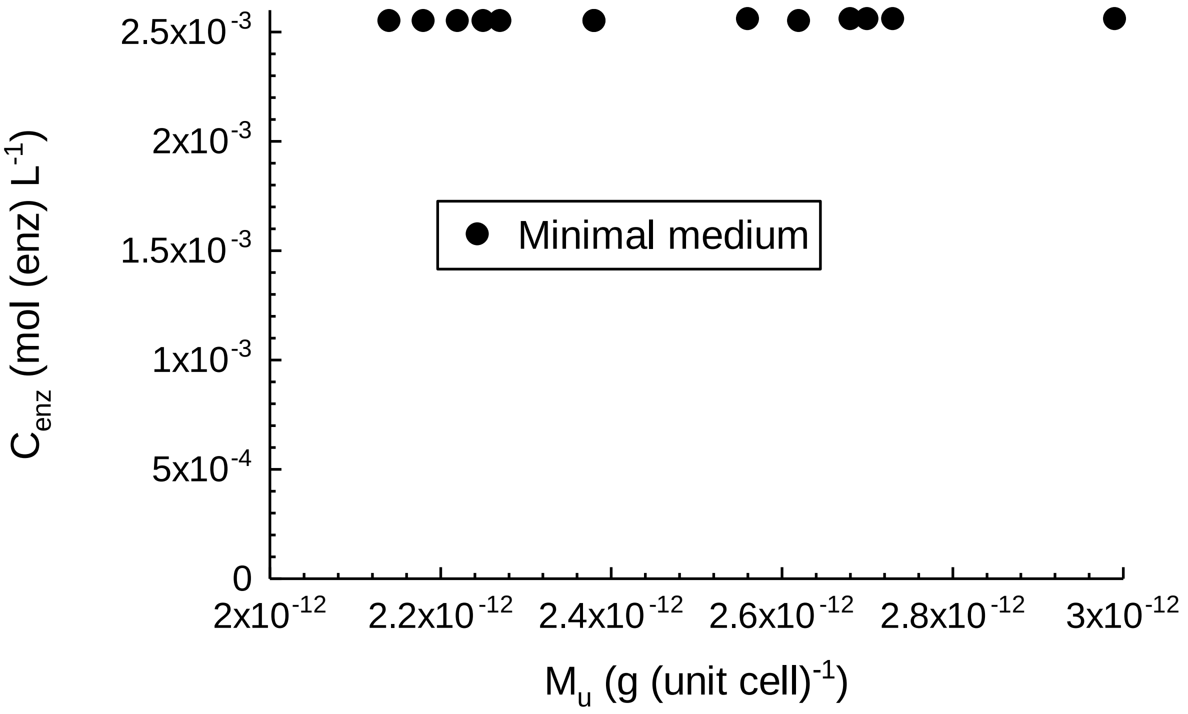

Supplementary Fig. 44. Concentration of enzymes in unit cells. The dependence between calculated values of unit cell mass (M_u, g (unit cell)$^{-1}$) and total molar concentration of enzymes (C_{enz}, mol (enz) L^{-1}) from data of ref. [14] using SSUCM-M and SSUCM-R models (Supplementary Table 10).

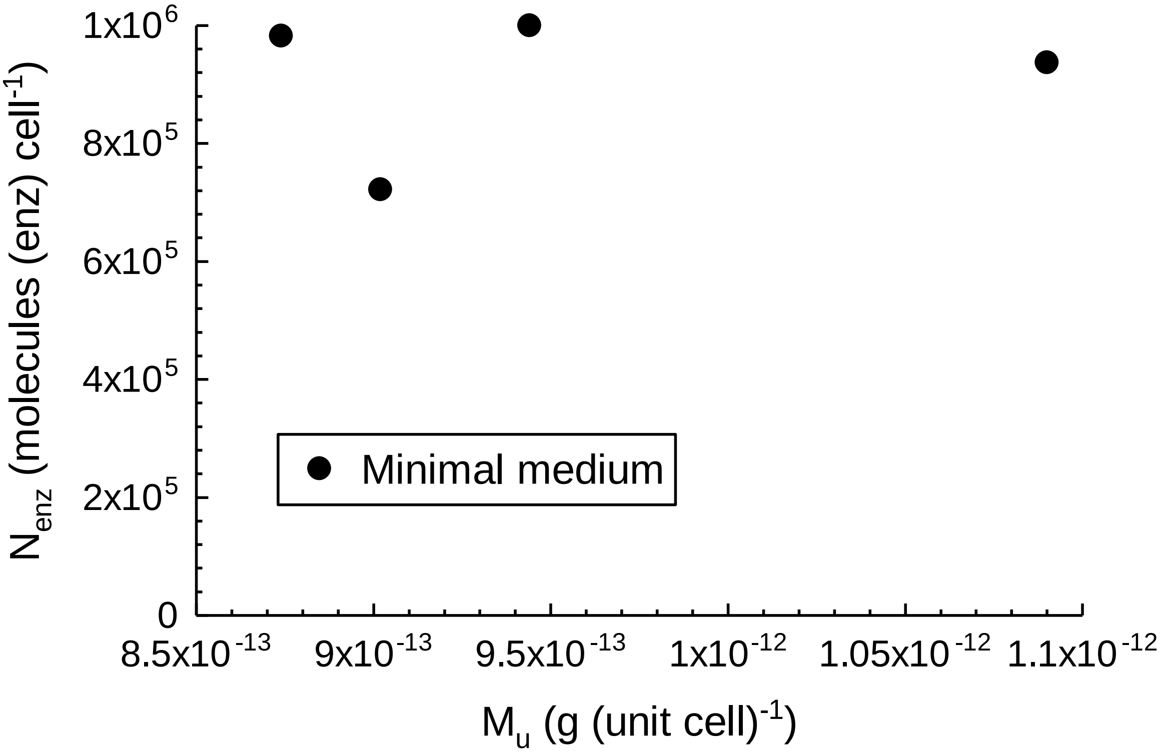

Supplementary Fig. 45. Number of enzymes in unit cells. The dependence between calculated values of unit cell mass (M_u, g (unit cell)$^{-1}$) and number of enzymes in the cell (N_{enz}, molecules (enz) cell^{-1}) from data of ref. [20] using SSUCM-M and SSUCM-R models (Supplementary Table 10).

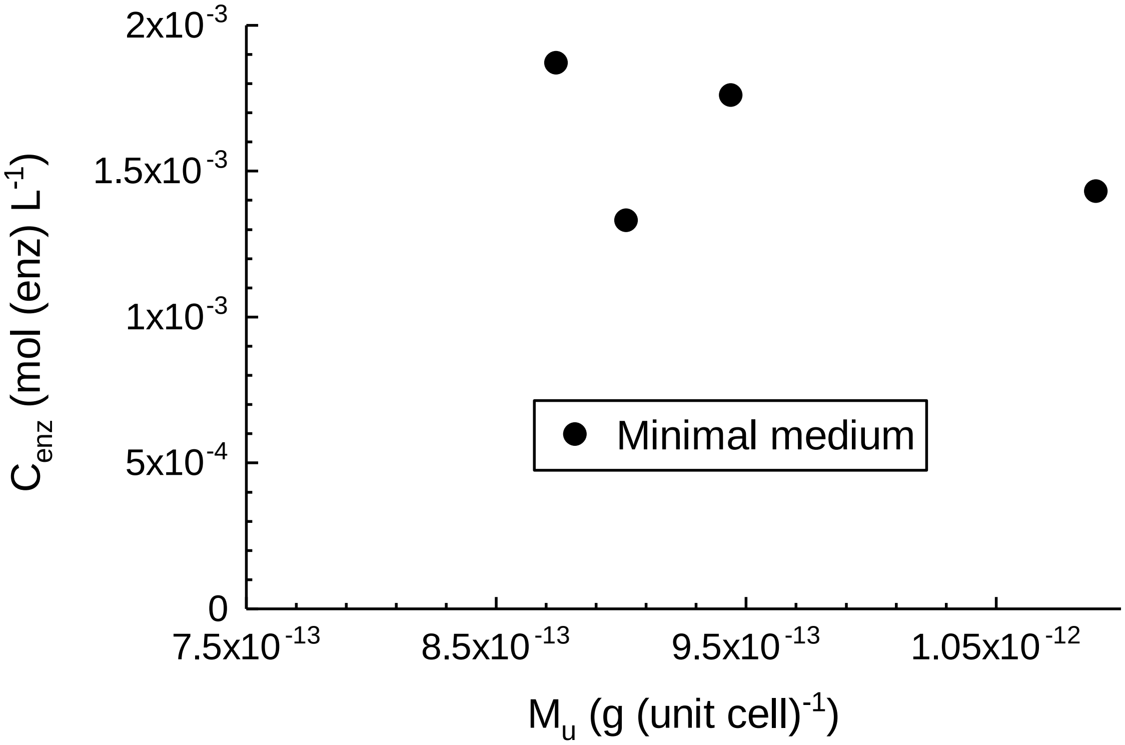

Supplementary Fig. 46. Concentration of enzymes in unit cells. The dependence between calculated values of unit cell mass (M_u, g (unit cell)$^{-1}$) and total molar concentration of enzymes (C_{enz}, mol (enz) L^{-1}) from data of ref. [20] using SSUCM-M and SSUCM-R models (Supplementary Table 10).

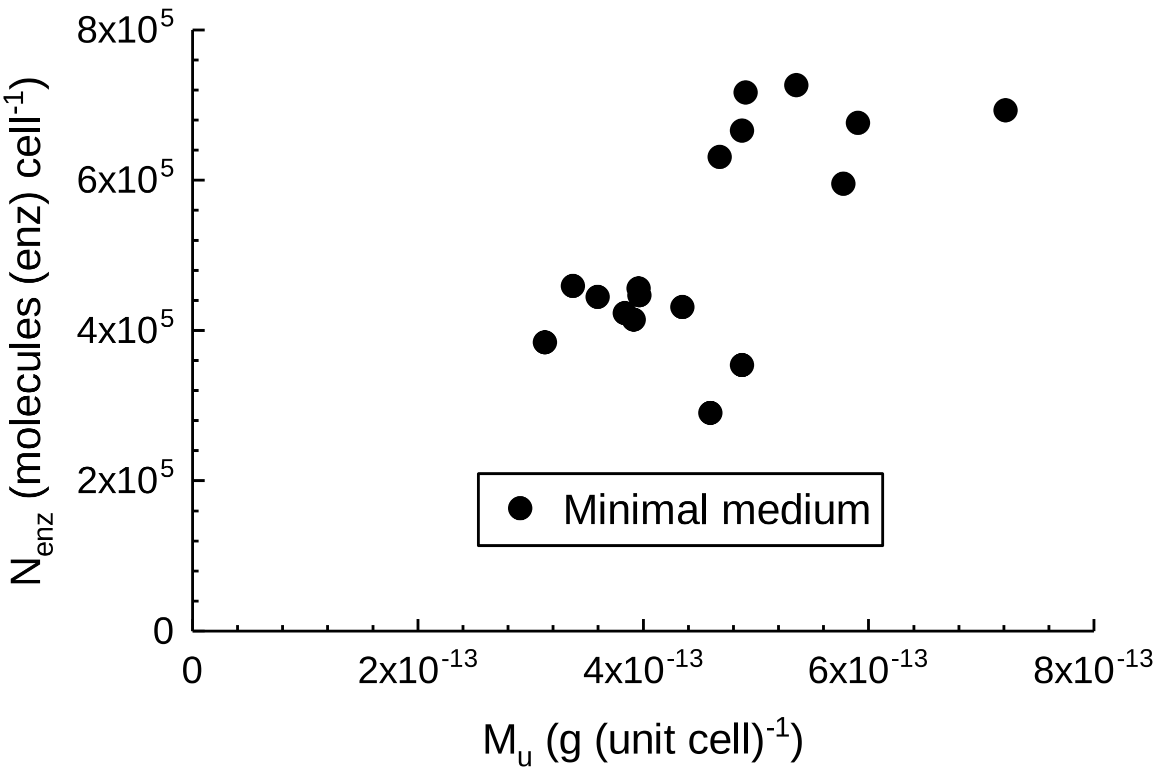

Supplementary Fig. 47. Number of enzymes in unit cells. The dependence between calculated values of unit cell mass (M_u, g (unit cell)$^{-1}$) and number of enzymes in the cell (N_{enz}, molecules (enz) cell^{-1}) from data of ref. [18] using SSUCM-M and SSUCM-R models (Supplementary Table 10).

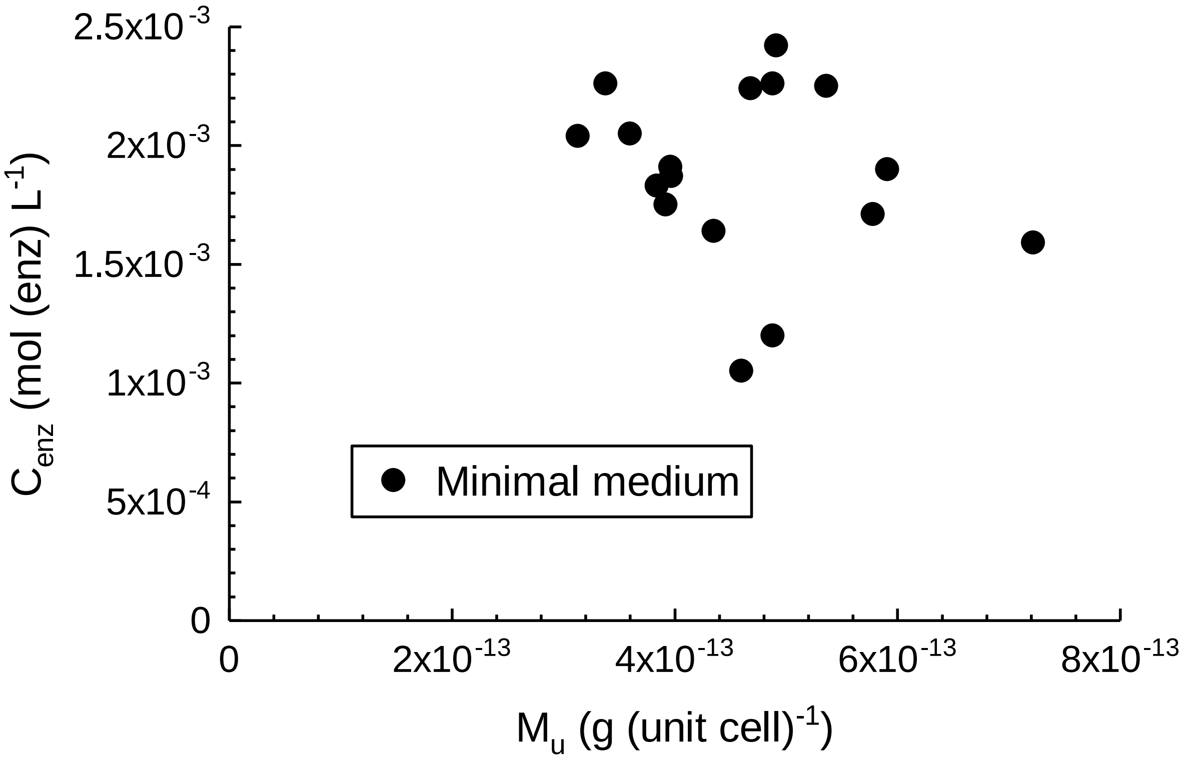

Supplementary Fig. 48. Concentration of enzymes in unit cells. The dependence between calculated values of unit cell mass (M_u, g (unit cell)$^{-1}$) and total molar concentration of enzymes (C_{enz}, mol (enz) L^{-1}) from data of ref. [18] using SSUCM-M and SSUCM-R models (Supplementary Table 10).

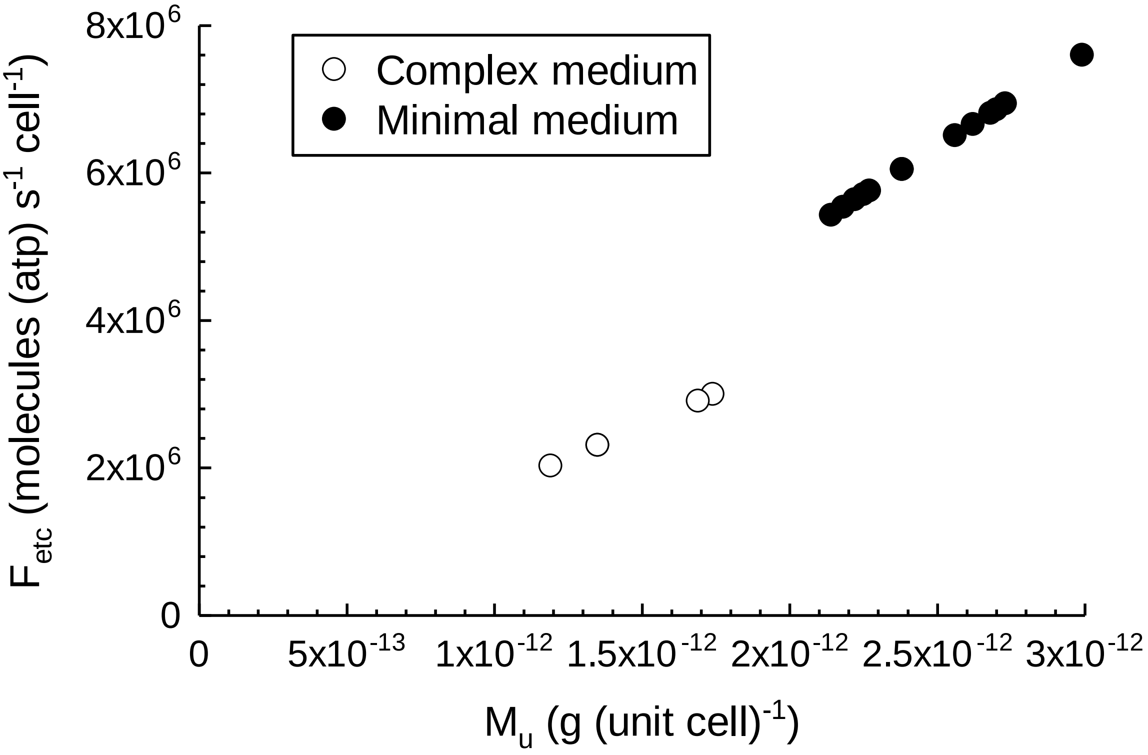

Supplementary Fig. 49. Energy flux in unit cells. The dependence between calculated values of unit cell mass (M_u, g (unit cell)$^{-1}$) and ATP synthesis flux of electron transport chain complex (F_{etc}, molecules (atp) s^{-1} cell^{-1}) from data of ref. [14] using SSUCM-M and SSUCM-R models (Supplementary Table 10).

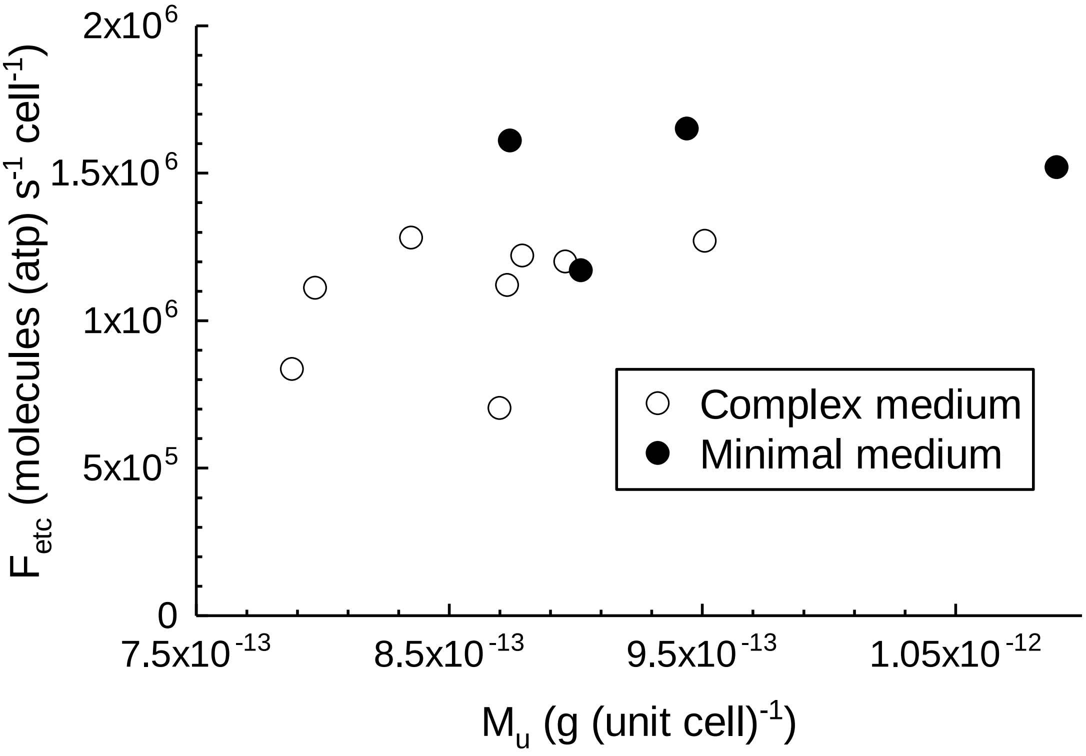

Supplementary Fig. 50. Energy flux in unit cells. The dependence between calculated values of unit cell mass (M_u, g (unit cell)$^{-1}$) and ATP synthesis flux of electron transport chain complex (F_{etc}, molecules (atp) s^{-1} cell^{-1}) from data of ref. [20] using SSUCM-M and SSUCM-R models (Supplementary Table 10).

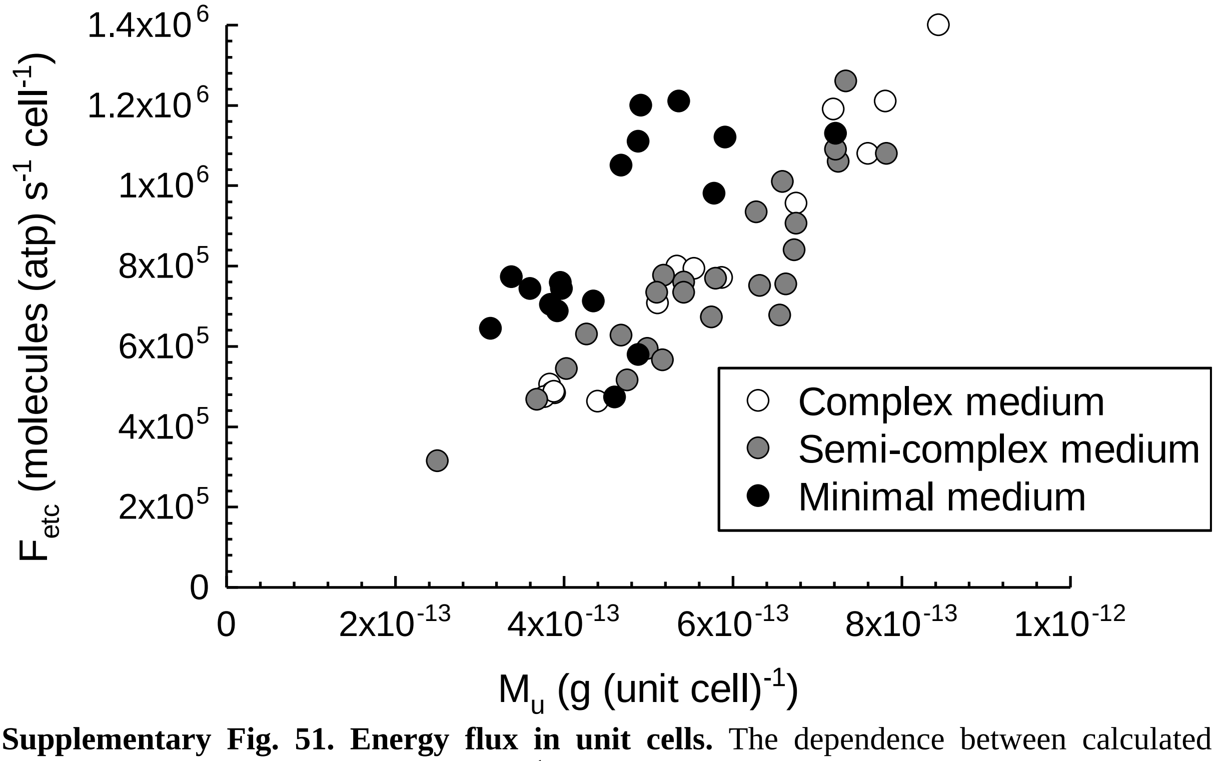

Supplementary Fig. 51. Energy flux in unit cells. The dependence between calculated values of unit cell mass (M_u, g (unit cell)$^{-1}$) and ATP synthesis flux of electron transport chain

complex (F_{etc}, molecules (atp) s^{-1} $cell^{-1}$) from data of ref. [18] using SSUCM-M and SSUCM-R models (Supplementary Table 10).

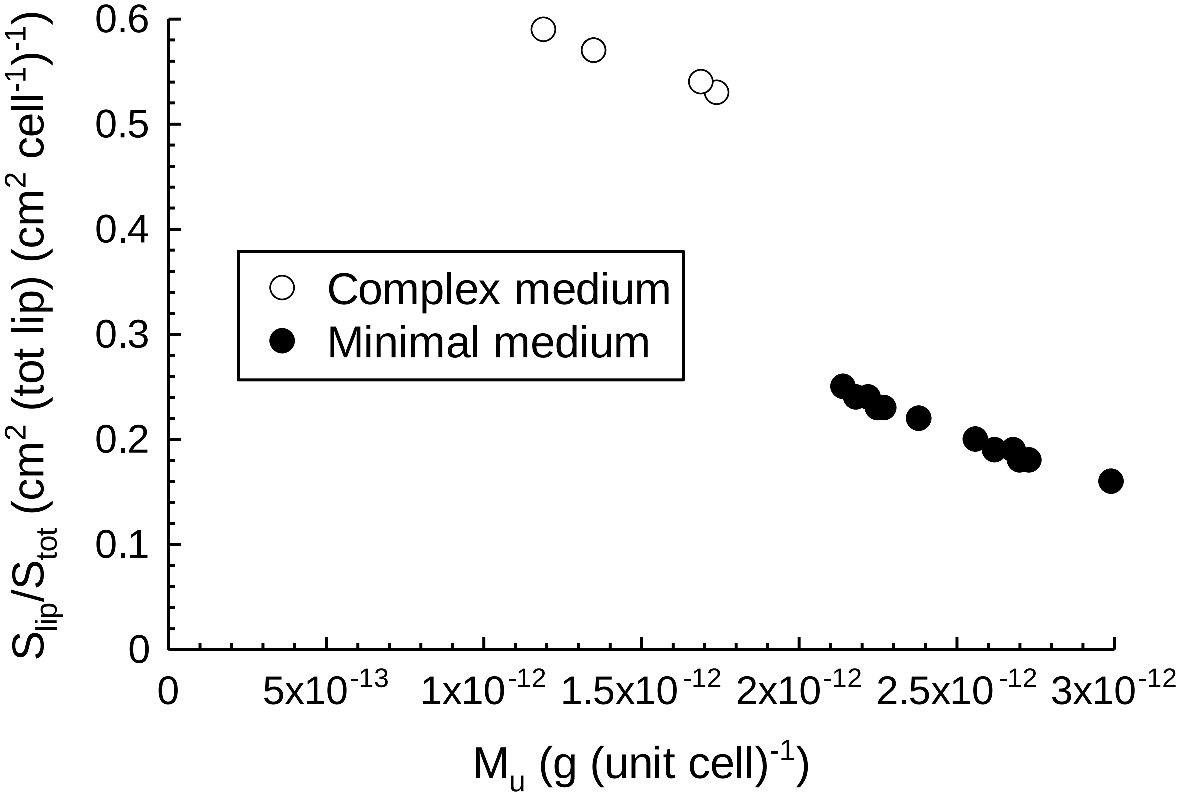

Supplementary Fig. 52. Membrane coverage in unit cells. The dependence between calculated values of unit cell mass (M_u, g (unit cell)$^{-1}$) and ratio of membrane surface covered by lipids to the cell surface (S_{lip}/S_{tot}, cm^2 (tot lip) ((cm^2 $cell^{-1}$)$^{-1}$) from data of ref. [14] using SSUCM-M and SSUCM-R models (Supplementary Table 10).

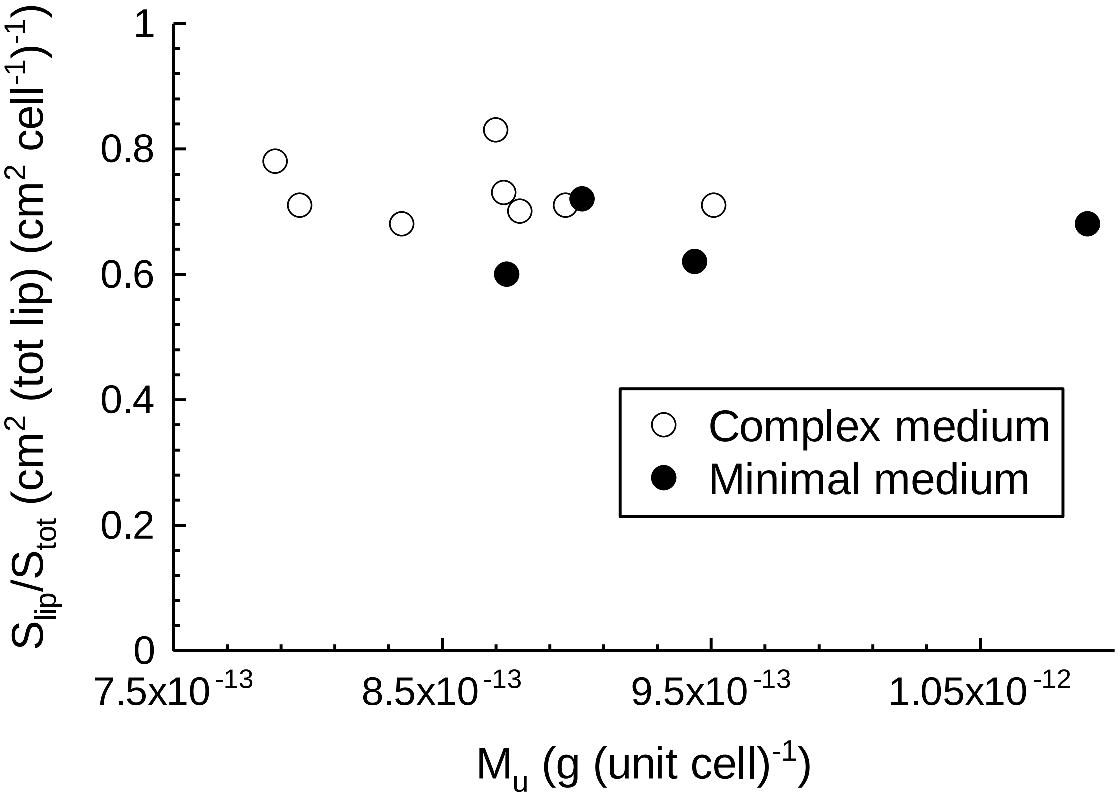

Supplementary Fig. 53. Membrane coverage in unit cells. The dependence between calculated values of unit cell mass (M_u, g (unit cell)$^{-1}$) and ratio of membrane surface covered by lipids to the cell surface (S_{lip}/S_{tot}, cm^2 (tot lip) ((cm^2 cell^{-1})$^{-1}$) from data of ref. [20] using SSUCM-M and SSUCM-R models (Supplementary Table 10).

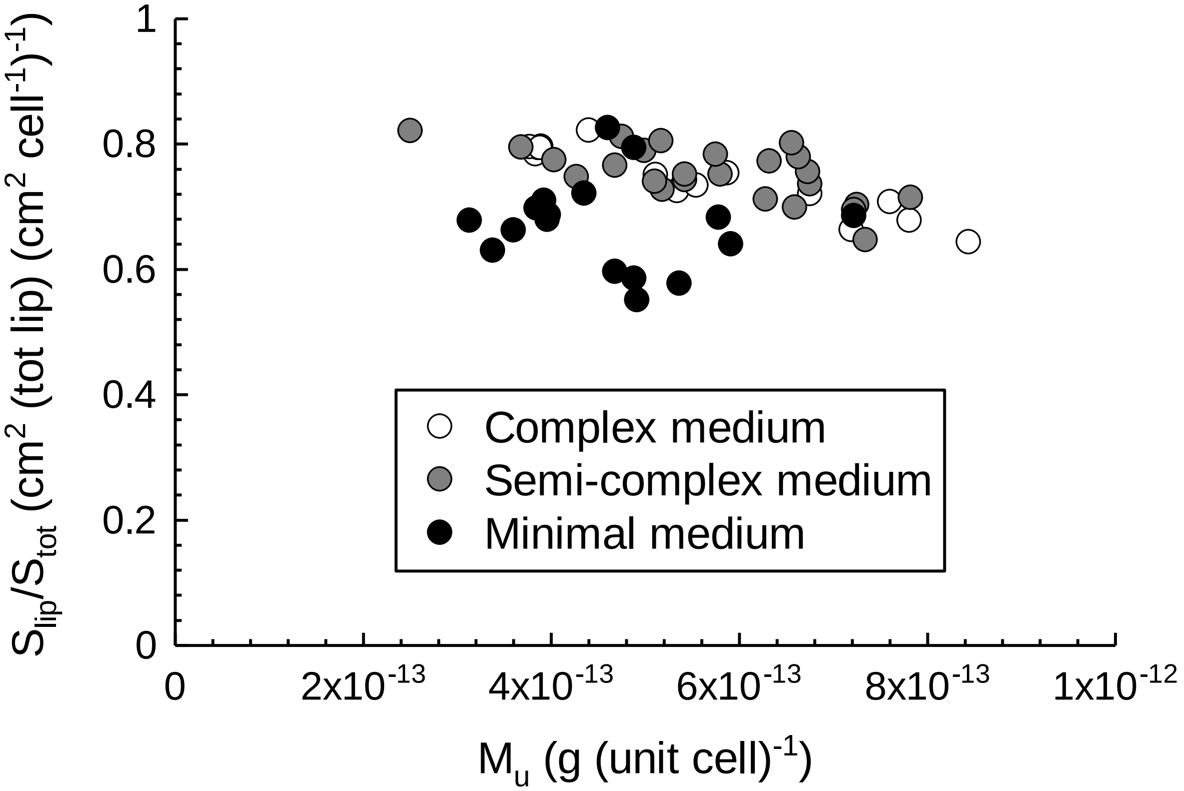

Supplementary Fig. 54. Membrane coverage in unit cells. The dependence between calculated values of unit cell mass (M_u, g (unit cell)$^{-1}$) and ratio of membrane surface covered by lipids to the cell surface (S_{lip}/S_{tot}, cm^2 (tot lip) ((cm^2 cell^{-1})$^{-1}$) from data of ref. [18] using SSUCM-M and SSUCM-R models (Supplementary Table 10).

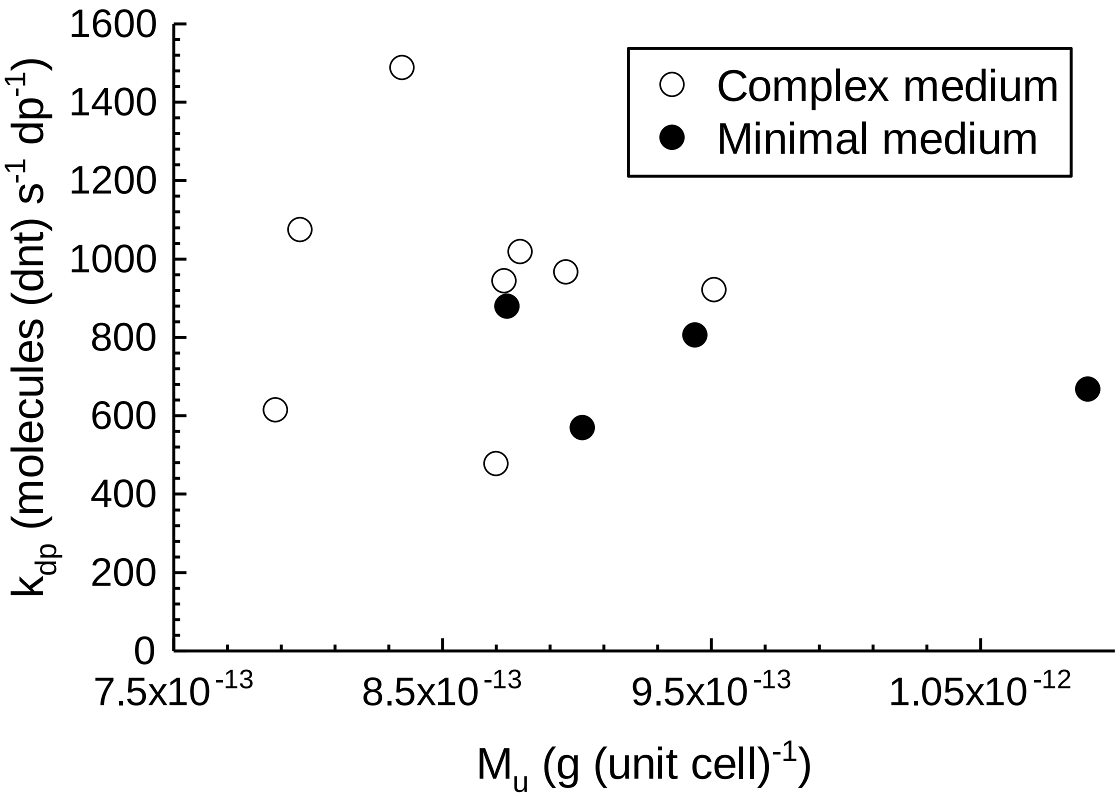

Supplementary Fig. 55. DNA polymerase velocity in unit cells. The dependence between calculated values of unit cell mass (M_u, g (unit cell)$^{-1}$) and apparent working rate of DNA polymerase (k_{dp}, molecules (dnt) s^{-1} dp^{-1}) from data of ref. [20] using SSUCM-M and SSUCM-R models (Supplementary Table 10).

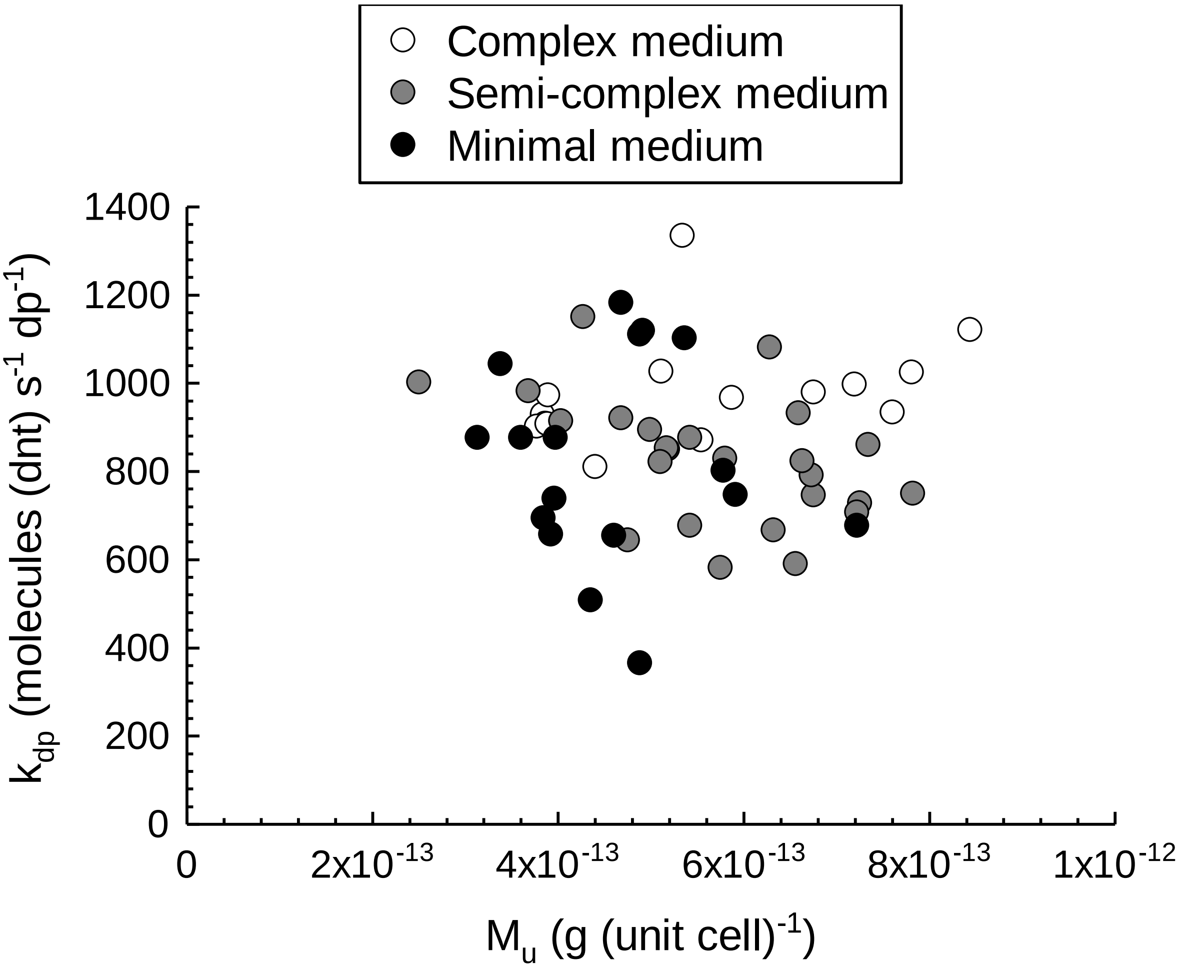

Supplementary Fig. 56. DNA polymerase velocity in unit cells. The dependence between calculated values of unit cell mass (M_u, g (unit cell)$^{-1}$) and apparent working rate of DNA polymerase (k_{dp}, molecules (dnt) s^{-1} dp^{-1}) from data of ref. [18] using SSUCM-M and SSUCM-R models (Supplementary Table 10).

Supplementary Discussion 5.9: Simplified derivation of t_{CDopt}

Figure 6 describes the hyperbolic dependencies between N_{rs} and Q_{cp}. It must be stressed that if C_{rs} is used instead of N_{rs}, then hyperbolic relations are transformed into linear relations (Supplementary Fig. 57).

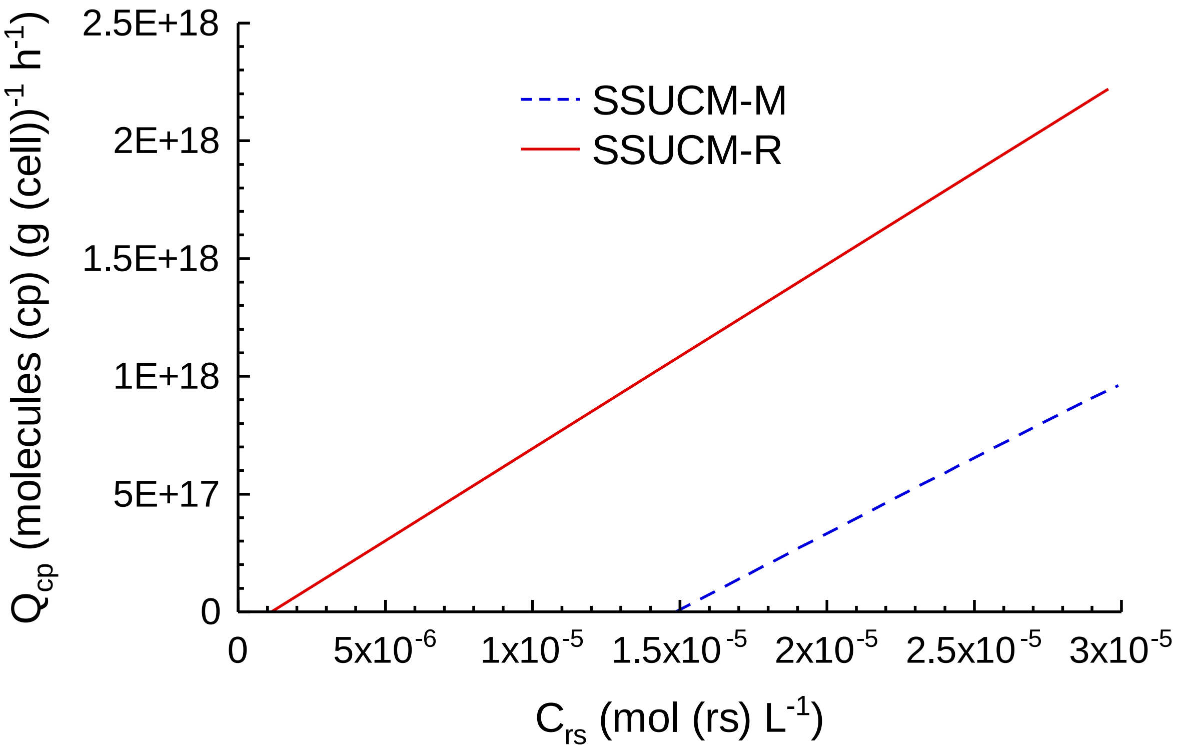

Supplementary Fig. 57: Productivity of unit cells (UCs). The relationship between the specific productivity of cushioning protein synthesis (Q_{cp}, molecules (cp) (g (cell))$^{-1}$ h^{-1}) and the molar concentration of ribosomes (C_{rs}, mol (rs) L^{-1}) of UCs growing on rich (solid line) and minimal medium (dashed line) if the cell cycle length of the unit cell t_{CD} is equal to *approximate* 3520 s = 1.0 h.

The SSUCM framework allows also calculate and determine the approximate location of the maximum of Q_{cp} as explained in the section "The productivity of cushioning protein synthesis in unit cells has optima". Here, the relation of the optimum and growth boundary is explained further by the following simplified derivation. Let us calculate t_{CDopt} in relation to t_{CDmin} (see Fig. 7c). Firstly, it is expected that only SRS is synthesized during t_{CDmin} which means that N_{cp} = 0 in case of characteristic growth boundary of smaller UCs. Secondly, it is assumed that N_{rs} is kept constant during changing from t_{CDmin} to t_{CDopt}. Obviously, $t_{CDopt} > t_{CDmin}$ because the content of self-reproducing cell components (ribosomes) decreases in the UC due to the increase of CP synthesis during t_{CD} increase. CP is synthesized ensuring maximal productivity at t_{CDopt}. Although the N_{rs} value remains constant at t_{CDopt}, there are certain differences between SRS (numbers and time for synthesis of components of SRS) at t_{CDopt} and t_{CDmin}. Therefore, additional time t_{se} is spent on rearrangements and synthesis of SRS at t_{CDopt}. To summarize, the synthesis of CP takes a relative period $t_{CDopt} - t_{CDmin} - t_{se}$ at t_{CDopt} whereas the synthesis of complete UC takes a relative period t_{CDopt}. The parameter T_{cp} is introduced instead of Q_{cp} (different physical meaning) and T_{cp} is expressed by the following Supplementary Eq. (10) which is analogical to Eq. (2):

$$T_{cp} = \frac{t_{CDopt} - t_{CD\min} - t_{se}}{t_{CDopt}} \cdot \frac{\ln 2}{t_{CDopt}} \tag{10}$$

The t_{CDopt} value at T_{cp} can be found by using the first derivative test and assuming the Gaussian function with global optimum (Supplementary Eq. (11)):

$$T_{cp}{}' = \frac{\ln 2}{t_{CDopt}{}^2} - \frac{2 \cdot \ln 2 \cdot \left(t_{CDopt} - t_{CD\min} - t_{se}\right)}{t_{CDopt}{}^3} \tag{11}$$

which leads to the optimality condition after solving Supplementary Eq. (11) at $T_{cp}{}' = 0$ Supplementary Eq. (12)):

$$t_{CDopt} = 2 \cdot \left(t_{CD\min} + t_{se}\right) \tag{12}$$

According to the derivation above, t_{CDopt} is approximately twice that needed for the reproduction of SRS. This relationship is universal although concrete values depend on the expression rate of CP. If the expression is higher, then T_{cp} is higher, growth can be faster and t_{CDopt} is smaller.

However, it should be kept in mind that Supplementary Eq. (12) is an extremely simplistic case and described theoretical dependencies are valid only if certain assumptions are made. The main assumption is that the CP (or more precisely cellular space) determines the growth boundaries. In the case of other growth limitation factors and altered base assumptions, the derivation becomes insufficient as T_{cp} might be located beyond growth boundaries and CP is also synthesized at t_{CDmin}.

In addition, if the values of k_{cell_comp} are increasing with t_{CD} decrease then it is possible that M_u increase rate does not exceed t_{CD} change. (If we assume that UC is modified so that the expression of CP remains the same then the location of Q_{cp_max} depends on the increase of M_u.) In that case, Q_{cp} would be increasing with decreasing t_{CD} but the optimum might be much more near to the t_{CDmin} value and the shape might be much more flat. If M_u growth is equal to the change of t_{CD} then Q_{cp} would be characterized by a horizontal line. If k_{cell_comp} decreases considerably with t_{CD}, it is also possible that t_{CDopt} would be near the maximal t_{CD}.

The physical content of the parameter t_{se} is not unequivocal and rather difficult to explain. For example, t_{CDmin} = 2520 s = 0.7 h and t_{CDopt} = 5250 s = 1.5 h at $N_{rs} = 10^4$ molecules (rs) cell^{-1} (Fig. 7c) or at $C_{rs} = 2 \cdot 10^{-5}$ mol (rs) L^{-1} and t_{se} is approximately 200 s = 0.06 h based on Supplementary Eq. (12). The detailed analysis of this example showed that the values of $N_{enz\_PW2\_r}$, N_{rs}, N_{rc}, N_{dna}, N_{rrna} and N_{trna} remained constant at t_{CDmin} and t_{CDopt}. However, the values of N_{cp} and N_{mrna_cp} were not the only ones that increased after the shift from t_{CDmin} to t_{CDopt}. The value of N_{lip} increased approximately 3 times because the cell was obviously bigger and less membrane proteins were needed at t_{CDopt} whereas the increase of N_{lpe} and $N_{enz\_PW5\_r}$ was smaller (1.4 times) as there was more time for synthesis of lipids. A longer synthesis period was also the reason why parameter values of remaining SRS components decreased: $N_{enz\_PW4\_r}$ and N_{rp} decreased approximately 2 times, energy production (N_{etc}) 10 % and $F_{enz\_PW1\_r}$ and F_{stp} 5 %. In conclusion, the final SRS was slightly different from the original SRS but the effects of those differences are not very clear. The calculations showed that approximately 45 % of t_{CD} or N_{rs} were involved in the synthesis of SRS proteins at t_{CDopt} which means that t_{d_srs} was approximately 2370 s = 0.7 h. It also means that calculated $t_{d\_srs} < t_{CDmin}$ (by 150 s = 0.04 h) and protein synthesis was not the reason for the appearance of t_{se}. Furthermore, M_{srs} was approximately $4.37 \cdot 10^{-13}$ g srs^{-1} at t_{CDopt} whereas M_u was $4.45 \cdot 10^{-13}$ g (unit cell)$^{-1}$ at t_{CDmin} which means that the overall difference of M_{srs} values was negligible. Formally, it seems that t_{se} is solely caused by lipid synthesis increase at t_{CDopt}. Although CP was represented as the only CPrm component in the current text based on the simplest qualitative division of cell components into SRS and CL categories, the more precise quantitative division of cell components is more complex (Supplementary Discussion 5.5) and

justifies the inclusion of part of SRS (like currently observed lipid synthesis increase) to cushioning mass in certain cases.

Supplementary Discussion 5.10: Descriptions of used models

Used SSUCMs (Fig. 8, Supplementary Fig. 58) are similar to SSPCMs described thoroughly in ref. [3]. The models involve various cellular interactions (cellular processes and reactions, stoichiometric dependencies etc.) between different model components. Cellular model components and interactions comprise the system of linear and nonlinear algebraic equations[3] (Eqs. (1)-(2), Supplementary Eqs. (13)-(32), (39)-(48)) based on a list of simplifications and assumptions (steady-state, exact cell doubling, linear growth law of the cell etc.) providing stoichiometric models (section "Description of models"). The equations define various balances (cell components, time, mass/volume, surface area) in the models and are more explicitly described in ref. [3] and the following subsections. Parameters of equations (and of model components) are defined and input parameter values necessary to carry out calculations are provided in Supplementary Discussion 5.11.2.2 of ref. [3] and Supplementary Discussions 5.10.1.2, 5.10.2.2, 5.10.3.2, 5.10.4.2. Input parameters correspond mostly to such cellular parameters (like dimensions and compositions of molecules) that are determined only by genotype whereas output parameters depend also on cell growth (like cell size, flux patterns, numbers of biopolymers) (Supplementary Tables 25-26 of ref. [3] and Supplementary Tables 12, 15-16). The majority of the values of input parameters are *specific*, *precise*, *average* or *approximate* corresponding to values of *E. coli* K12 MG1655 if possible. The remaining smaller part of parameter values is *generic* or variables. To simplify the solving of models, the number of different model components and the number of relations between them are equal which enables to find unique solutions without optimization (Supplementary Discussions 5.10.1.3, 5.10.2.3, 5.10.3.3, 5.10.4.3). Also, the selection of input and output parameters enables to simplify model solving by carrying it out in different steps and part of the equations (for example equations describing DNA replication, flux patterns, cellular compositions or geometry properties)) can be solved and corresponding parameters calculated separately after solving the main system of equations. Some values of calculated output parameters are provided in Supplementary Tables 13-14.

SUPPLEMENTARY DISCUSSION 5.10.1: DESCRIPTION OF SSUCM-SRS-M

The developed SSUCM-SRS-M has exactly the same cellular structures, cell components and interaction types as SSPCM-SRS-M (Supplementary Discussion 5.11.2 of ref. [3]). There are differences only in equations and parameters ($t_{d\_srs\text{-}m}$ and t_{d_srs} are replaced by t_{CD}, M_{tot} is replaced by M_u) due to the introduction of UC concept. Only the differences compared to SSPCM-SRS-M are subsequently presented.

Supplementary Discussion 5.10.1.1: Model components and interactions

The metabolic network and cell components of the SSUCM-SRS-M are visualized schematically in Fig. 3 of ref. [3].

Supplementary Discussion 5.10.1.1.1: Metabolic network

The metabolic network is the same as in SSPCM-SRS-M (Supplementary Discussion 5.11.2.1.1 of ref. [3]).

Supplementary Discussion 5.10.1.1.2: Protein synthesis

The protein fraction of the UC is comprised of the same proteins and the description of their synthesis is basically similar to SSPCM-SRS-M (Supplementary Discussion 5.11.2.1.2 of ref. [3]). $N_{rs\_cell\_comp}$ of proteins are determined by t_{CD} and the requirement of amino acids of each protein according to the following Supplementary Eqs. (13)-(19) of which right side expressions are identical to those of Supplementary Eqs. (80), (82)-(87) of ref. [3] and only left side expressions are different because $t_{d\_srs\text{-}m}$ and t_{d_srs} are replaced by t_{CD}:

$$t_{CD} = \frac{N_{rs} \cdot n_{rpc}}{N_{rs\_rpc} \cdot k_{rs}} \tag{13}$$

$$t_{CD} = \frac{N_{rp} \cdot n_{rp}}{N_{rs\_rp} \cdot k_{rs}} \tag{14}$$

$$t_{CD} = \frac{n_{enz} \cdot \sum_{i=1}^{5} N_{enz\_PWi\_r} \cdot l_{PWi}}{N_{rs\_enz} \cdot k_{rs}} \tag{15}$$

$$t_{CD} = \frac{N_{lpe} \cdot n_{lpe}}{N_{rs\_lpe} \cdot k_{rs}} \tag{16}$$

$$t_{CD} = \frac{N_{etc} \cdot n_{etc}}{N_{rs\_etc} \cdot k_{rs}} \tag{17}$$

$$t_{CD} = \frac{N_{stp} \cdot n_{stp}}{N_{rs\_stp} \cdot k_{rs}} \tag{18}$$

$$t_{CD} = \frac{N_{rc} \cdot n_{rc}}{N_{rs\_rc} \cdot k_{rs}} \tag{19}$$

N_{rs} is expressed as the sum of all $N_{rs\_cell\_comp}$ (Supplementary Eq. (88) of ref. [3]).

Supplementary Discussion 5.10.1.1.3: RNA synthesis

The RNA fraction of the UC is comprised of the same RNAs and the description of their synthesis is basically similar to SSPCM-SRS-M (Supplementary Discussion 5.11.2.1.3 of ref. [3]). Transcription is described by Supplementary Eq. (91) of ref. [3] and by the following Supplementary Eq. (20) of which the right side expression is identical to that of Supplementary Eq. (90) of ref. [3] and only left side expressions are different because $t_{d\_srs\text{-}m}$ is replaced by t_{CD}:

$$t_{CD} = \frac{N_{rrna} \cdot n_{rrna} + N_{trna} \cdot n_{trna} + \sum_{1}^{7} N_{mrna\_cell\_comp} \cdot n_{mrna\_cell\_comp}}{N_{rp} \cdot k_{rp}} \tag{20}$$

The stoichiometry of the ribosome is described by Supplementary Eq. (92) of ref. [3] and tRNA balances by Supplementary Eq. (93) of ref. [3]. $N_{mrna\_cell\_comp}$ values are determined by translational activity and also by P_{cell_comp} (Supplementary Eqs. (94)-(100) of ref. [3]).

Supplementary Discussion 5.10.1.1.4: Lipid synthesis

The value of N_{lip} is doubled during the cell cycle by LPE according to the following Supplementary Eq. (21) of which the right-hand side is identical to Supplementary Eq. (101) of ref. [3] and only left side expressions are different because $t_{d\_srs\text{-}m}$ is replaced by t_{CD}:

$$t_{CD} = \frac{N_{lip}}{N_{lpe} \cdot k_{lpe}} \quad (21)$$

Supplementary Discussion 5.10.1.1.5: Energy balance

The description of energy synthesis and consumption balance of the UC is the same as in SSPCM-SRS-M (Supplementary Discussion 5.11.2.1.5 of ref. [3]).

Supplementary Discussion 5.10.1.1.6: Cell geometry

The geometry of the UC is the same as in SSPCM-SRS-M (Supplementary Discussion 5.11.2.1.6 of ref. [3]).

Supplementary Discussion 5.10.1.1.7: Mass balance

M_u can be divided roughly into two parts by cell structures – M_{cyt} and M_{mem} – and it is the sum of masses of all cell components including water (mass of water is described by *approximate DWC*) according to the following Supplementary Eq. (22) of which right-hand side is identical to Supplementary Eq. (105) of ref. [3] and only left side expressions are different because M_{tot} is replaced by M_u:

$$M_u = M_{cyt} + \frac{N_{lip} \cdot m_{lip} + N_{stp} \cdot m_{stp} + N_{etc} \cdot m_{etc}}{DWC} \quad (22)$$

The cytoplasmic space of the UC is filled by the same cell component species as for SSPCM-SRS-M and therefore M_{cyt} is described by Supplementary Eqs. (106)-(107) of ref. [3].

Supplementary Discussion 5.10.1.1.8: DNA synthesis

The description of DNA replication is basically similar to that of SSPCM-SRS-M (Supplementary Discussion 5.11.2.1.8 of ref. [3]) and genome synthesis is described by the following Supplementary Eq. (23) of which the right-hand side is identical to Supplementary Eq. (108) of ref. [3] and only left side expressions are different because $t_{d\_srs\text{-}m}$ is replaced by t_{CD}:

$$t_{CD} = \frac{N_{dna} \cdot n_{dna}}{2 \cdot N_{rce} \cdot k_{dp}} \quad (23)$$

Supplementary Discussion 5.10.1.2: Model parameters

Corresponding input parameters, their values and output parameters of SSPCM-SRS-M (Supplementary Discussions 5.11.2.2, 5.11.2.2.1-5.11.2.2.2 of ref. [3]) are also used in the current model except M_{tot} (replaced by M_u), $t_{d\_srs\text{-}m}$ and t_{d_srs} (replaced by t_{CD}). SSUCM-SRS-M includes the following input and output parameters missing in SSPCM-SRS-M (Supplementary Table 12).

Supplementary Table 12. Input and output parameters of SSUCM-SRS-M that are missing in SSPCM-SRS-M.

Cell parameter	Symbol	Unit
**Input**		
Cell cycle length of unit cell [12.1]	t_{CD}	s and h
Cell division time [12.2]	t_D	s and h
**Output**		

Unit cell mass [12.3]	M_u	g (unit cell)$^{-1}$
**Molar concentrations [12.4]**		
Molar concentration of ribosomes	C_{rs}	mol (rs) L^{-1}
Molar concentration of ribosomes for the synthesis of RPCs	C_{rs_rpc}	mol (rs rpc) L^{-1}
Molar concentration of ribosomes for the synthesis of enzymes	C_{rs_enz}	mol (rs enz) L^{-1}
Molar concentration of ribosomes for the synthesis of RP complexes	C_{rs_rp}	mol (rs rp) L^{-1}
Molar concentration of ribosomes for the synthesis of LPEs	C_{rs_lpe}	mol (rs lpe) L^{-1}
Molar concentration of ribosomes for the synthesis of ETC complexes	C_{rs_etc}	mol (rs etc) L^{-1}
Molar concentration of ribosomes for the synthesis of transport proteins	C_{rs_stp}	mol (rs stp) L^{-1}
Molar concentration of ribosomes for the synthesis of RCs	C_{rs_rc}	mol (rs rc) L^{-1}
Molar concentration of RP complexes	C_{rp}	mol (rp) L^{-1}
Molar concentration of LPE	C_{lpe}	mol (lpe) L^{-1}
Molar concentration of ETC complexes	C_{etc}	mol (etc) L^{-1}
Molar concentration of transport protein complexes	C_{stp}	mol (stp) L^{-1}
Molar concentration of effective RCs	C_{rce}	mol (rce) L^{-1}
Molar concentration of enzyme catalysing a single reaction r of central metabolic pathway PW_1	$C_{enz\_PW1\_r}$	mol (enz PW_1) L^{-1} reaction^{-1}
Molar concentration of enzyme catalysing a single reaction r of amino acid biosynthesis pathway PW_2	$C_{enz\_PW2\_r}$	mol (enz PW_2) L^{-1} reaction^{-1}
Molar concentration of enzyme catalysing a single reaction r of deoxyribonucleotide biosynthesis pathway PW_3	$C_{enz\_PW3\_r}$	mol (enz PW_3) L^{-1} reaction^{-1}
Molar concentration of enzyme catalysing a single reaction r of ribonucleotide biosynthesis pathway PW_4	$C_{enz\_PW4\_r}$	mol (enz PW_4) L^{-1} reaction^{-1}
Molar concentration of enzyme catalysing a single reaction r of lipid biosynthesis pathway PW_5	$C_{enz\_PW5\_r}$	mol (enz PW_5) L^{-1} reaction^{-1}
Molar concentration of assembled rRNA complexes	C_{rrna}	mol (rrna) L^{-1}
Molar concentration of tRNA	C_{trna}	mol (trna) L^{-1}
Molar concentration of mRNA of RPC	C_{mrna_rpc}	mol (mrna rpc) L^{-1}
Molar concentration of mRNA of RP complex	C_{mrna_rp}	mol (mrna rp) L^{-1}
Molar concentration of mRNA of enzyme	C_{mrna_enz}	mol (mrna enz) L^{-1}
Molar concentration of mRNA of LPE	C_{mrna_lpe}	mol (mrna lpe) L^{-1}
Molar concentration of mRNA of ETC complex	C_{mrna_etc}	mol (mrna etc) L^{-1}
Molar concentration of mRNA of transport protein	C_{mrna_stp}	mol (mrna stp) L^{-1}

Molar concentration of mRNA of RC	C_{mrna_rc}	mol (mrna rc) L^{-1}
Molar concentration of cell membrane lipid	C_{lip}	mol (lip) L^{-1}

[12.1] The *approximate* standard value (3520 s = 1.0 h) of t_{CD} is calculated from Eq. (1) based on standard values of t_D (Supplementary Table 12) and t_C (Supplementary Table 24 of ref. [3]). The parameter t_{CD} was also used as an independent variable with the range of $t_{CD} = t_{CDmin} - 10^4$ s = 2.8 h. The selected range of t_{CD} values in the paper is larger than experimentally observed values of *E. coli*[16,17]. The lower ($t_{CD} < 3000$ s = 0.8 h) t_{CD} values were selected in the analysis in order to calculate and visualize theoretical growth boundaries. Further explanations have been provided in Supplementary Discussion 5.10.3.2.1.
[12.2] *Approximate* standard value (1200 s = 0.3 h) of t_D corresponds to higher growth rates of *E. coli* cells[5]. The parameter t_D was also used as an independent variable with the range of 150 – 8000 s = 0.04 – 2.2 h. The selected range of t_D values in the paper is larger than experimentally observed values of *E. coli*[16-17,21]. The lower ($t_D < 1200$ s = 0.3 h) t_D values were selected in the analysis in order to calculate and visualize theoretical growth boundaries. Further explanations have been provided in Supplementary Discussion 5.10.3.2.1.
[12.3] Output parameter.
[12.4] Molar concentration parameters are calculated according to the following equation where 1000 is the conversion factor from cm^3 to Liter:

$$C_{cell\_comp} = \frac{N_{cell\_comp}}{N_A} \cdot \frac{\rho_{tot}}{M_u} \cdot 1000 \qquad (24)$$

Supplementary Discussion 5.10.1.3: Calculation scheme

The calculation scheme is identical to SSPCM-SRS-M (Supplementary Discussion 5.11.2.3 of ref. [3]). Some calculation results of the model are presented in Supplementary Table 13.

Supplementary Table 13. Calculated output parameters of SSUCM-SRS-M.

Parameter	Symbol	Unit	Values	
Cell cycle length of unit cell	t_{CD}	s	2474	3520
		h	0.7	1
Cell division time	t_D	s	155	1200
		h	0.04	0.3
Number of ribosomes in the cell	N_{rs}	molecules (rs) cell^{-1}	$3.7 \cdot 10^4$	337
Molar concentration of ribosomes	C_{rs}	mol (rs) L^{-1}	$4.0 \cdot 10^{-5}$	$1.5 \cdot 10^{-5}$
Number of ribosomes for the synthesis of ribosomal protein complexes (RPC)	N_{rs_rpc}	molecules (rs rpc) cell^{-1}	5414	35
Molar concentration of ribosomes for the synthesis of RPCs	C_{rs_rpc}	mol (rs rpc) L^{-1}	$5.8 \cdot 10^{-6}$	$1.5 \cdot 10^{-6}$
Number of ribosomes for the synthesis of enzymes	N_{rs_enz}	molecules (rs enz) cell^{-1}	$2.0 \cdot 10^4$	175
Molar concentration of ribosomes for the synthesis of enzymes	C_{rs_enz}	mol (rs enz) L^{-1}	$2.1 \cdot 10^{-5}$	$7.7 \cdot 10^{-6}$
Number of ribosomes for the synthesis of RNA polymerase (RP) complexes	N_{rs_rp}	molecules (rs rp) cell^{-1}	214	0.97
Molar concentration of ribosomes for the synthesis of RP complexes	C_{rs_rp}	mol (rs rp) L^{-1}	$2.3 \cdot 10^{-7}$	$4.3 \cdot 10^{-8}$
Number of ribosomes for the synthesis of lipid synthesis enzymes (LPE)	N_{rs_lpe}	molecules (rs lpe) cell^{-1}	$4.7 \cdot 10^{-5}$	0.018
Molar concentration of ribosomes for the synthesis of LPEs	C_{rs_lpe}	mol (rs lpe) L^{-1}	$5.0 \cdot 10^{-14}$	$7.9 \cdot 10^{-10}$
Number of ribosomes for the synthesis of electron transport chain (ETC) complexes	N_{rs_etc}	molecules (rs etc) cell^{-1}	$1.1 \cdot 10^4$	122
Molar concentration of ribosomes for the synthesis of ETC complexes	C_{rs_etc}	mol (rs etc) L^{-1}	$1.2 \cdot 10^{-5}$	$5.4 \cdot 10^{-6}$
Number of ribosomes for the synthesis of transport proteins	N_{rs_stp}	molecules (rs stp) cell^{-1}	331	3
Molar concentration of ribosomes for the synthesis of transport proteins	C_{rs_stp}	mol (rs stp) L^{-1}	$3.6 \cdot 10^{-7}$	$1.3 \cdot 10^{-7}$
Number of ribosomes for the synthesis of replisome complexes (RC)	N_{rs_rc}	molecules (rs rc) cell^{-1}	2	1
Molar concentration of ribosomes for the synthesis of RCs	C_{rs_rc}	mol (rs rc) L^{-1}	$2.1 \cdot 10^{-9}$	$4.4 \cdot 10^{-8}$
Number of RP complexes in the cell	N_{rp}	molecules (rp) cell^{-1}	1899	12
Molar concentration of RP complexes	C_{rp}	mol (rp) L^{-1}	$2.0 \cdot 10^{-6}$	$5.3 \cdot 10^{-7}$
Number of LPE molecules in the cell	N_{lpe}	molecules (lpe) cell^{-1}	0.008	4

Molar concentration of LPE	C_{lpe}	mol (lpe) L^{-1}	$8.6 \cdot 10^{-12}$	$1.8 \cdot 10^{-7}$
Number of ETC complexes in the cell	N_{etc}	molecules (etc) $cell^{-1}$	$5.5 \cdot 10^{4}$	858
Molar concentration of ETC complexes	C_{etc}	mol (etc) L^{-1}	$5.9 \cdot 10^{-5}$	$3.8 \cdot 10^{-5}$
Number of transport protein molecules in the cell	N_{stp}	molecules (stp) $cell^{-1}$	8196	103
Molar concentration of transport protein complexes	C_{stp}	mol (stp) L^{-1}	$8.8 \cdot 10^{-6}$	$4.5 \cdot 10^{-6}$
Number of effective RC in the cell	N_{rce}	molecules (rce) $cell^{-1}$	2	1
Molar concentration of effective RCs	C_{rce}	mol (rce) L^{-1}	$2.1 \cdot 10^{-9}$	$4.4 \cdot 10^{-8}$
Number of molecules of the enzyme catalysing a single reaction r of central metabolic pathway PW_1	$N_{enz\_PW1\_r}$	molecules (enz PW_1) $cell^{-1}$ $reaction^{-1}$	8196	103
Molar concentration of enzyme catalysing a single reaction r of central metabolic pathway PW_1	$C_{enz\_PW1\_r}$	mol (enz PW_1) L^{-1} $reaction^{-1}$	$8.8 \cdot 10^{-6}$	$4.5 \cdot 10^{-6}$
Number of molecules of the enzyme catalysing a single reaction r of amino acid biosynthesis pathway PW_2	$N_{enz\_PW2\_r}$	molecules (enz PW_2) $cell^{-1}$ $reaction^{-1}$	7399	67
Molar concentration of enzyme catalysing a single reaction r of amino acid biosynthesis pathway PW_2	$C_{enz\_PW2\_r}$	mol (enz PW_2) L^{-1} $reaction^{-1}$	$7.9 \cdot 10^{-6}$	$3.0 \cdot 10^{-6}$
Number of molecules of the enzyme catalysing a single reaction r of deoxyribonucleotide biosynthesis pathway PW_3	$N_{enz\_PW3\_r}$	molecules (enz PW_3) $cell^{-1}$ $reaction^{-1}$	38	26
Molar concentration of enzyme catalysing a single reaction r of deoxyribonucleotide biosynthesis pathway PW_3	$C_{enz\_PW3\_r}$	mol (enz PW_3) L^{-1} $reaction^{-1}$	$4.1 \cdot 10^{-8}$	$1.2 \cdot 10^{-6}$
Number of molecules of the enzyme catalysing a single reaction r of ribonucleotide biosynthesis pathway PW_4	$N_{enz\_PW4\_r}$	molecules (enz PW_4) $cell^{-1}$ $reaction^{-1}$	760	5
Molar concentration of enzyme catalysing a single reaction r of ribonucleotide biosynthesis pathway PW_4	$C_{enz\_PW4\_r}$	mol (enz PW_4) L^{-1} $reaction^{-1}$	$8.1 \cdot 10^{-7}$	$2.2 \cdot 10^{-7}$
Number of molecules of the enzyme catalysing a single reaction r of lipid biosynthesis pathway PW_5	$N_{enz\_PW5\_r}$	molecules (enz PW_5) $cell^{-1}$ $reaction^{-1}$	0.008	4
Molar concentration of enzyme catalysing a single reaction r of lipid biosynthesis pathway PW_5	$C_{enz\_PW5\_r}$	mol (enz PW_5) L^{-1} $reaction^{-1}$	$8.6 \cdot 10^{-12}$	$1.8 \cdot 10^{-7}$

Number of assembled rRNA complexes in the cell	N_{rrna}	molecules (rrna) cell^{-1}	$3.7 \cdot 10^{4}$	337
Molar concentration of assembled rRNA complexes	C_{rrna}	mol (rrna) L^{-1}	$4.0 \cdot 10^{-5}$	$1.5 \cdot 10^{-5}$
Number of tRNA molecules in the cell	N_{trna}	molecules (trna) cell^{-1}	$1.9 \cdot 10^{5}$	1687
Molar concentration of tRNA	C_{trna}	mol (trna) L^{-1}	$2.0 \cdot 10^{-4}$	$7.4 \cdot 10^{-5}$
Number of molecules of mRNA of RPC in the cell	N_{mrna_rpc}	molecules (mrna rpc) cell^{-1}	54	0.35
Molar concentration of mRNA of RPC	C_{mrna_rpc}	mol (mrna rpc) L^{-1}	$5.8 \cdot 10^{-8}$	$1.5 \cdot 10^{-8}$
Number of molecules of mRNA of RP complex in the cell	N_{mrna_rp}	molecules (mrna rp) cell^{-1}	2	0.0097
Molar concentration of mRNA of RP complex	C_{mrna_rp}	mol (mrna rp) L^{-1}	$2.1 \cdot 10^{-9}$	$4.3 \cdot 10^{-10}$
Number of molecules of mRNA of enzyme in the cell	N_{mrna_enz}	molecules (mrna enz) cell^{-1}	199	2
Molar concentration of mRNA of enzyme	C_{mrna_enz}	mol (mrna enz) L^{-1}	$2.1 \cdot 10^{-7}$	$8.8 \cdot 10^{-8}$
Number of molecules of mRNA of LPE in the cell	N_{mrna_lpe}	molecules (mrna lpe) cell^{-1}	$2 \cdot 10^{-6}$	$8.9 \cdot 10^{-4}$
Molar concentration of mRNA of LPE	C_{mrna_lpe}	mol (mrna lpe) L^{-1}	$2.1 \cdot 10^{-15}$	$3.9 \cdot 10^{-11}$
Number of molecules of mRNA of ETC complex in the cell	N_{mrna_etc}	molecules (mrna etc) cell^{-1}	112	1
Molar concentration of mRNA of ETC complex	C_{mrna_etc}	mol (mrna etc) L^{-1}	$1.2 \cdot 10^{-7}$	$4.4 \cdot 10^{-8}$
Number of molecules of mRNA of transport protein in the cell	N_{mrna_stp}	molecules (mrna stp) cell^{-1}	3	0.029
Molar concentration of mRNA of transport protein	C_{mrna_stp}	mol (mrna stp) L^{-1}	$3.2 \cdot 10^{-9}$	$1.3 \cdot 10^{-9}$
Number of molecules of mRNA of RC in the cell	N_{mrna_rc}	molecules (mrna rc) cell^{-1}	0.02	0.014
Molar concentration of mRNA of RC	C_{mrna_rc}	mol (mrna rc) L^{-1}	$2.1 \cdot 10^{-11}$	$6.2 \cdot 10^{-10}$
Number of cell membrane lipid molecules in the cell	N_{lip}	molecules (lip) cell^{-1}	1913	$1.5 \cdot 10^{6}$
Molar concentration of cell membrane lipid	C_{lip}	mol (lip) L^{-1}	$2.1 \cdot 10^{-6}$	0.065
Mass of cytoplasm of the cell	M_{cyt}	g cyt^{-1}	$1.18 \cdot 10^{-12}$	$2.87 \cdot 10^{-14}$
Unit cell mass	M_u	g (unit cell)$^{-1}$	$1.55 \cdot 10^{-12}$	$3.77 \cdot 10^{-14}$

SUPPLEMENTARY DISCUSSION 5.10.2: DESCRIPTION OF SSUCM-SRS-R

The developed SSUCM-SRS-R has exactly the same cellular structures, cell components and interaction types as SSPCM-SRS-R (Supplementary Discussion 5.11.11 of ref. [3]). There are differences only in equations and parameters ($t_{d\_srs\text{-}r}$ and t_{d_srs} are replaced by t_{CD}, M_{tot} is replaced by M_u) due to the introduction of UC concept. Only the differences compared to SSPCM-SRS-R are subsequently presented.

Supplementary Discussion 5.10.2.1: Model components and interactions

The metabolic network and cell components of the SSUCM-SRS-R are visualized schematically in Supplementary Fig. 9 of ref. [3].

Supplementary Discussion 5.10.2.1.1: Metabolic network

The metabolic network is the same as in SSPCM-SRS-R (Supplementary Discussion 5.11.11.1.1 of ref. [3]).

Supplementary Discussion 5.10.2.1.2: Protein synthesis

The protein fraction of the UC is comprised of the same proteins as in SSPCM-SRS-R (Supplementary Discussion 5.11.11.1.2 of ref. [3]) and the synthesis of proteins is described by Supplementary Eqs. (13)-(14), (16)-(19) of SSUCM-SRS-M. N_{rs} is expressed as the sum of all $N_{rs\_cell\_comp}$ (Supplementary Eq. (181) of ref. [3]).

Supplementary Discussion 5.10.2.1.3: RNA synthesis

The RNA fraction of the UC is comprised of the same RNAs and the description of their synthesis is basically similar to SSPCM-SRS-R (Supplementary Discussion 5.11.11.1.3 of ref. [3]). Transcription is described by Supplementary Eq. (183) of ref. [3] and by the following Supplementary Eq. (25) of which the right side expression is identical to that of Supplementary Eq. (182) of ref. [3] and only left side expressions are different because $t_{d\_srs\text{-}r}$ is replaced by t_{CD}:

$$t_{CD} = \frac{N_{rrna} \cdot n_{rrna} + N_{trna} \cdot n_{trna} + \sum_{1}^{6} N_{mrna\_cell\_comp} \cdot n_{mrna\_cell\_comp}}{N_{rp} \cdot k_{rp}} \tag{25}$$

The stoichiometry of the ribosome is described by Supplementary Eq. (92) of ref. [3] and tRNA balances by Supplementary Eq. (93) of ref. [3]. $N_{mrna\_cell\_comp}$ values are determined by translational activity and also by P_{cell_comp} (Supplementary Eqs. (94)-(99) of ref. [3]).

Supplementary Discussion 5.10.2.1.4: Lipid synthesis

The description of lipid synthesis is the same as in SSUCM-SRS-M (Supplementary Discussion 5.10.1.1.4).

Supplementary Discussion 5.10.2.1.5: Energy balance

The description of energy synthesis and consumption balance of the UC is the same as in SSPCM-SRS-R (Supplementary Discussion 5.11.11.1.5 of ref. [3]).

Supplementary Discussion 5.10.2.1.6: Cell geometry

The geometry of the UC is the same as in SSPCM-SRS-M (Supplementary Discussion 5.11.2.1.6 of ref. [3]).

Supplementary Discussion 5.10.2.1.7: Mass balance

M_u can be divided roughly into two parts by cell structures – M_{cyt} and M_{mem} – and it is the sum of masses of all cell components including water (mass of water is described by *approximate DWC*) according to Supplementary Eq. (22). The mass balance of M_{cyt} is the same as in SSPCM-SRS-R (Supplementary Discussion 5.11.11.1.7 of ref. [3]).

Supplementary Discussion 5.10.2.1.8: DNA

The DNA replication is described in Supplementary Discussion 5.10.1.1.8.

Supplementary Discussion 5.10.2.2: Model parameters

Corresponding input parameters, their values and output parameters of SSPCM-SRS-R (Supplementary Discussions 5.11.11.2 of ref. [3]) are also used in the current model except M_{tot} (replaced by M_u), $t_{d\_srs\text{-}m}$ and t_{d_srs} (replaced by t_{CD}). SSUCM-SRS-R includes also unique input and output parameters of SSUCM-SRS-M (Supplementary Table 12), further explanations have been provided in Supplementary Discussion 5.10.4.2.

Supplementary Discussion 5.10.2.3: Calculation scheme

The calculation scheme is identical to SSPCM-SRS-R (Supplementary Discussion 5.11.11.3 of ref. [3]). Some calculation results of the model are presented in Supplementary Table 14.

Supplementary Table 14. Calculated output parameters of SSUCM-SRS-R.

Parameter	Symbol	Unit	Values	
Cell cycle length of unit cell	t_{CD}	s	936	3520
		h	0.3	1
Cell division time	t_D	s	0	1200
		h	0	0.3
Apparent working rate of DNA polymerase	k_{dp}	molecules (dnt) s^{-1} dp^{-1}	2477	1000
Number of ribosomes in the cell	N_{rs}	molecules (rs) $cell^{-1}$	$2.5 \cdot 10^4$	13
Molar concentration of ribosomes	C_{rs}	mol (rs) L^{-1}	$7.5 \cdot 10^{-5}$	$1.1 \cdot 10^{-6}$
Number of ribosomes for the synthesis of ribosomal protein complexes (RPC)	N_{rs_rpc}	molecules (rs rpc) $cell^{-1}$	9544	1
Molar concentration of ribosomes for the synthesis of RPCs	C_{rs_rpc}	mol (rs rpc) L^{-1}	$2.9 \cdot 10^{-5}$	$8.7 \cdot 10^{-8}$
Number of ribosomes for the synthesis of RNA polymerase (RP) complexes	N_{rs_rp}	molecules (rs rp) $cell^{-1}$	1021	0.039
Molar concentration of ribosomes for the synthesis of RP complexes	C_{rs_rp}	mol (rs rp) L^{-1}	$3.1 \cdot 10^{-6}$	$3.4 \cdot 10^{-9}$
Number of ribosomes for the synthesis of lipid synthesis enzymes (LPE)	N_{rs_lpe}	molecules (rs lpe) $cell^{-1}$	$4 \cdot 10^{-5}$	0.015
Molar concentration of ribosomes for the synthesis of LPEs	C_{rs_lpe}	mol (rs lpe) L^{-1}	$1.2 \cdot 10^{-13}$	$1.3 \cdot 10^{-9}$
Number of ribosomes for the synthesis of electron transport chain (ETC) complexes	N_{rs_etc}	molecules (rs etc) $cell^{-1}$	$1.3 \cdot 10^4$	9
Molar concentration of ribosomes for the synthesis of ETC complexes	C_{rs_etc}	mol (rs etc) L^{-1}	$4.1 \cdot 10^{-5}$	$7.8 \cdot 10^{-7}$
Number of ribosomes for the synthesis of transport proteins	N_{rs_stp}	molecules (rs stp) $cell^{-1}$	684	0.93
Molar concentration of ribosomes for the synthesis of transport proteins	C_{rs_stp}	mol (rs stp) L^{-1}	$2.1 \cdot 10^{-6}$	$8.1 \cdot 10^{-8}$
Number of ribosomes for the synthesis of replisome complexes (RC)	N_{rs_rc}	molecules (rs rc) $cell^{-1}$	5	1
Molar concentration of ribosomes for the synthesis of RCs	C_{rs_rc}	mol (rs rc) L^{-1}	$1.5 \cdot 10^{-8}$	$8.7 \cdot 10^{-8}$
Number of RP complexes in the cell	N_{rp}	molecules (rp) $cell^{-1}$	3432	0.49
Molar concentration of RP complexes	C_{rp}	mol (rp) L^{-1}	$1.0 \cdot 10^{-5}$	$4.3 \cdot 10^{-8}$
Number of LPE molecules in the cell	N_{lpe}	molecules (lpe) $cell^{-1}$	0.003	3
Molar concentration of LPE	C_{lpe}	mol (lpe) L^{-1}	$9.1 \cdot 10^{-12}$	$2.6 \cdot 10^{-7}$

Number of ETC complexes in the cell	N_{etc}	molecules (etc) cell^{-1}	$2.5 \cdot 10^{4}$	65
Molar concentration of ETC complexes	C_{etc}	mol (etc) L^{-1}	$7.6 \cdot 10^{-5}$	$5.7 \cdot 10^{-6}$
Number of transport protein molecules in the cell	N_{stp}	molecules (stp) cell^{-1}	6408	33
Molar concentration of transport protein complexes	C_{stp}	mol (stp) L^{-1}	$1.9 \cdot 10^{-5}$	$2.9 \cdot 10^{-6}$
Number of effective RC in the cell	N_{rce}	molecules (rce) cell^{-1}	2	1
Molar concentration of effective RCs	C_{rce}	mol (rce) L^{-1}	$6.1 \cdot 10^{-9}$	$8.7 \cdot 10^{-8}$
Number of assembled rRNA complexes in the cell	N_{rrna}	molecules (rrna) cell^{-1}	$2.5 \cdot 10^{4}$	13
Molar concentration of assembled rRNA complexes	C_{rrna}	mol (rrna) L^{-1}	$7.5 \cdot 10^{-5}$	$1.1 \cdot 10^{-6}$
Number of tRNA molecules in the cell	N_{trna}	molecules (trna) cell^{-1}	$1.2 \cdot 10^{5}$	65
Molar concentration of tRNA	C_{trna}	mol (trna) L^{-1}	$3.7 \cdot 10^{-4}$	$5.7 \cdot 10^{-6}$
Number of molecules of mRNA of RPC in the cell	N_{mrna_rs}	molecules (mrna rpc) cell^{-1}	95	0.013
Molar concentration of mRNA of RPC	C_{mrna_rs}	mol (mrna rpc) L^{-1}	$2.9 \cdot 10^{-7}$	$1.1 \cdot 10^{-9}$
Number of molecules of mRNA of RP complex in the cell	N_{mrna_rp}	molecules (mrna rp) cell^{-1}	10	$3.9 \cdot 10^{-4}$
Molar concentration of mRNA of RP complex	C_{mrna_rp}	mol (mrna rp) L^{-1}	$3.0 \cdot 10^{-8}$	$3.4 \cdot 10^{-11}$
Number of molecules of mRNA of LPE in the cell	N_{mrna_lpe}	molecules (mrna lpe) cell^{-1}	$2 \cdot 10^{-6}$	$7.3 \cdot 10^{-4}$
Molar concentration of mRNA of LPE	C_{mrna_lpe}	mol (mrna lpe) L^{-1}	$6.1 \cdot 10^{-15}$	$6.3 \cdot 10^{-11}$
Number of molecules of mRNA of ETC complex in the cell	N_{mrna_etc}	molecules (mrna etc) cell^{-1}	134	0.093
Molar concentration of mRNA of ETC complex	C_{mrna_etc}	mol (mrna etc) L^{-1}	$4.1 \cdot 10^{-7}$	$8.1 \cdot 10^{-9}$
Number of molecules of mRNA of transport protein in the cell	N_{mrna_stp}	molecules (mrna stp) cell^{-1}	7	0.0093
Molar concentration of mRNA of transport protein	C_{mrna_stp}	mol (mrna stp) L^{-1}	$2.1 \cdot 10^{-8}$	$8.1 \cdot 10^{-10}$
Number of molecules of mRNA of RC in the cell	N_{mrna_rc}	molecules (mrna rc) cell^{-1}	0.05	0.014
Molar concentration of mRNA of RC	C_{mrna_rc}	mol (mrna rc) L^{-1}	$1.5 \cdot 10^{-10}$	$1.2 \cdot 10^{-9}$
Number of cell membrane lipid molecules in the cell	N_{lip}	molecules (lip) cell^{-1}	242	$1.2 \cdot 10^{-6}$

Molar concentration of cell membrane lipid	C_{lip}	mol (lip) L^{-1}	$7.3\cdot10^{-7}$	$1.0\cdot10^{-13}$
Mass of cytoplasm of the cell	M_{cyt}	g cyt^{-1}	$3.75\cdot10^{-13}$	$1.61\cdot10^{-14}$
Unit cell mass	M_u	g (unit cell)$^{-1}$	$5.49\cdot10^{-13}$	$1.91\cdot10^{-14}$

SUPPLEMENTARY DISCUSSION 5.10.3: DESCRIPTION OF SSUCM-M

The description of the model (SSUCM-M) is based on the full descriptions of previous models (SSPCM-SRS-M and SSUCM-SRS-M (Supplementary Discussion 5.11.2 of ref. [3] and Supplementary Discussion 5.10.1)), only the differences compared with previous models are subsequently presented.

Supplementary Discussion 5.10.3.1: Model components and interactions

The developed SSUCM-M has essentially the same cell components and interactions as in SSPCM-SRS-M and SSUCM-SRS-M (Supplementary Tables 1-3 of ref. [3]). The main difference is the synthesis of CP and corresponding mRNA in the cytoplasmic space of SSUCM-M (Fig. 8). The concept of CP is analogical to ref. [42,44-45] where such cell component has been named dummy, useless and unspecified proteins, respectively. It is expected that their involvement is necessary and covers distinct aspects of physiology (regulation, structure, signalling, households, unused metabolic proteins) that are not described explicitly in models.

Supplementary Discussion 5.10.3.1.1: Metabolic network

The metabolic network is the same as in SSPCM-SRS-M and is described in Supplementary Discussion 5.11.2.1.1 of ref. [3].

Supplementary Discussion 5.10.3.1.2: Protein synthesis

The protein fraction of the UC is comprised of CP (does not have a specific function for self-reproduction and is not part of SRS, cushioning function) and of all different proteins (Fig. 8) from SSPCM-SRS-M (Supplementary Discussion 5.11.2.1.2 of ref. [3]). CP was also assumed to be single molecule (monomeric polypeptide chain) without different subunits.

The synthesis of proteins from SSPCM-SRS-M is described by Supplementary Eqs. (13)-(19). The synthesis of CP is described similarly to other proteins. The amount of CP is doubled during the cell cycle by ribosomes carrying out translation. Different actual steps of translation and post-translational processes (protein maturation, folding, cofactor binding, complex formation etc.) are not described. Instead, there is a heavily simplified translation process which uses amino acids as substrates and produces proteins. N_{rs_cp} is determined by t_{CD} and by the total number of amino acids needed for the synthesis of CP (Supplementary Eq. (26)):

$$t_{CD} = \frac{N_{cp} \cdot n_{cp}}{N_{rs\_cp} \cdot k_{rs}} \tag{26}$$

N_{rs} is expressed as the sum of all $N_{rs\_cell\_comp}$ (Supplementary Eq. (27)):

$$N_{rs} = N_{rs\_rc} + N_{rs\_rp} + N_{rs\_rpc} + N_{rs\_etc} + N_{rs\_stp} + N_{rs\_lpe} + N_{rs\_enz} + N_{rs\_cp} \tag{27}$$

Supplementary Discussion 5.10.3.1.3: RNA synthesis

RNA fraction of the UC is comprised of mRNA coding CP and of all different RNAs (Fig. 8) from SSPCM-SRS-M (Supplementary Discussion 5.11.2.1.3 of ref. [3]). The mRNA of CP is also assumed to contain only coding regions to simplify the model.

Numbers of all RNA molecules are doubled during the cell cycle by RP complexes carrying out transcription. Different actual steps of transcription and post-transcriptional processes (RNA processing, modifications, charging, degradation etc.) are not described. Instead, there

is a heavily simplified transcription process which uses ribonucleotides as substrates and produces RNA molecules (Supplementary Eqs. (28)-(29)):

$$t_{CD} = \frac{N_{rrna} \cdot n_{rrna} + N_{trna} \cdot n_{trna} + \sum_{1}^{8} N_{mrna\_cell\_comp} \cdot n_{mrna\_cell\_comp}}{N_{rp} \cdot k_{rp}} \tag{28}$$

$$\sum_{1}^{8} N_{mrna\_cell\_comp} \cdot n_{mrna\_cell\_comp} = N_{mrna\_rc} \cdot n_{mrna\_rc} + N_{mrna\_rp} \cdot n_{mrna\_rp} + N_{mrna\_rpc} \cdot n_{mrna\_rpc} +$$
$$+ N_{mrna\_etc} \cdot n_{mrna\_etc} + N_{mrna\_stp} \cdot n_{mrna\_stp} + N_{mrna\_lpe} \cdot n_{mrna\_lpe} + N_{mrna\_enz} \cdot n_{mrna\_enz} + N_{mrna\_cp} \cdot n_{mrna\_cp} \tag{29}$$

The stoichiometry of the ribosome is described by Supplementary Eq. (92) of ref. [3] and tRNA balances by Supplementary Eq. (93) of ref. [3]. $N_{mrna\_cell\_comp}$ values are determined by translational activity and also by P_{cell_comp} (Supplementary Eqs. (94)-(100) of ref. [3], Supplementary Eq. (30)).

$$N_{mrna\_cp} = N_{rs\_cp} \cdot P_{cp} \tag{30}$$

Supplementary Discussion 5.10.3.1.4: Lipid synthesis

The description of lipid synthesis is the same as in SSUCM-SRS-M (Supplementary Discussion 5.10.1.1.4).

Supplementary Discussion 5.10.3.1.5: Energy balance

The description of energy synthesis and consumption balance of the UC is the same as in SSPCM-SRS-M (Supplementary Discussion 5.11.2.1.5 of ref. [3]).

Supplementary Discussion 5.10.3.1.6: Cell geometry

The geometry of the UC is the same as in SSPCM-SRS-M (Supplementary Discussion 5.11.2.1.6 of ref. [3]).

Supplementary Discussion 5.10.3.1.7: Mass balance

M_u can be divided roughly into two parts by cell structures – M_{cyt} and M_{mem} – and it is the sum of masses of all cell components including water (mass of water is described by *approximate DWC*) according to Supplementary Eq. (22). The M_{cyt} equals with the sum of masses of all cell components (including water) localized in cytoplasmic space (Supplementary Eqs. (31)-(32)):

$$M_{cyt} = \frac{N_{dna} \cdot m_{dna} + N_{rc} \cdot m_{rc} + N_{rp} \cdot m_{rp} + N_{rs} \cdot m_{rpc} + N_{lpe} \cdot m_{lpe} + N_{trna} \cdot m_{trna} +}{DWC}$$

$$+ \frac{N_{rrna} \cdot m_{rrna} + m_{enz} \cdot \sum_{i=1}^{5} N_{enz\_PW_i\_r} \cdot l_{PW_i} + N_{cp} \cdot m_{cp} +}{DWC} \quad (31)$$

$$+ \frac{\sum_{1}^{8} N_{mrna\_cell\_comp} \cdot m_{mrna\_cell\_comp}}{DWC}$$

$$\sum_{1}^{8} N_{mrna\_cell\_comp} \cdot m_{mrna\_cell\_comp} = N_{mrna\_rc} \cdot m_{mrna\_rc} + N_{mrna\_rp} \cdot m_{mrna\_rp} +$$
$$+ N_{mrna\_rpc} \cdot m_{mrna\_rpc} + N_{mrna\_etc} \cdot m_{mrna\_etc} + N_{mrna\_stp} \cdot m_{mrna\_stp} + \quad (32)$$
$$+ N_{mrna\_lpe} \cdot m_{mrna\_lpe} + N_{mrna\_enz} \cdot m_{mrna\_enz} + N_{mrna\_cp} \cdot m_{mrna\_cp}$$

Supplementary Discussion 5.10.3.1.8: DNA synthesis

The DNA replication is described in Supplementary Discussion 5.10.1.1.8.

Supplementary Discussion 5.10.3.2: Model parameters

Corresponding input parameters, their values and output parameters of SSUCM-SRS-M (Supplementary Discussion 5.10.1.2) are also used in the current model with the following additional parameters described in sub-chapters.

Supplementary Discussion 5.10.3.2.1: Input parameters

Following input (Supplementary Table 15) parameters related to CP were used in SSUCM-M beside input parameters of SSUCM-SRS-M.

Supplementary Table 15. Input parameters of SSUCM-M that are missing in SSUCM-SRS-M.

Cell parameter	Symbol	Value	Term	Unit
Number of amino acid molecules in the cushioning protein (CP) molecule	n_{cp}	300	*generic*	molecules (aa) cp^{-1}
Number of ribonucleotide molecules in the molecule of mRNA of CP [15.1]	n_{mrna_cp}	900	*generic*	molecules (nt) $(mrna\ cp)^{-1}$
Mass of a molecule of mRNA of CP [15.2]	m_{mrna_cp}	$4.8 \cdot 10^{-19}$	*generic*	g $(mrna\ cp)^{-1}$
Mass of CP molecule [15.2]	m_{cp}	$5.9 \cdot 10^{-20}$	*generic*	g cp^{-1}
Polysome density of mRNA of CP [15.3]	P_{cp}	0.05	*generic*	molecules (nt (covered by rs)) (molecules (nt (covered by tot rs)))$^{-1}$
Minimal polysome density of mRNA of CP [15.4]	P_{cp_min}	0.044		molecules (nt (covered by rs)) (molecules (nt (mrna cp)$^{-1}$))$^{-1}$
Number of CP molecules in the cell [15.5]	N_{cp}			molecules (cp) $cell^{-1}$

[15.1] Value of *generic* n_{mrna_cp} is calculated from Supplementary Eq. (109) of ref. [3].
[15.2] Values are calculated from Supplementary Eq. (111) of ref. [3].
[15.3] Reciprocal value of *generic* P_{cp} (20 molecules (rs cp) $cell^{-1}$ (molecules (mrna cp) $cell^{-1}$)$^{-1}$) is the number of ribosome molecules per molecule of mRNA of CP. Additional explanations of polysome density parameter have been presented in Supplementary Table 22 of ref. [3].
[15.4] Reciprocal value of P_{cp_min} (23 molecules (rs cp) $cell^{-1}$ (molecules (mrna cp) $cell^{-1}$)$^{-1}$) is the maximum number of ribosome molecules per molecule of mRNA of CP. Additional explanations of minimal polysome density parameter have been presented in Supplementary Table 22 of ref. [3].
[15.5] N_{cp} was defined as an independent variable. The varied range was approximately 0 – $1.7 \cdot 10^{7}$ molecules (cp) $cell^{-1}$ (corresponding C_{cp} range was approximately 0 – 0.028 mol (cp) L^{-1}) and it corresponded to the M_u range of 10^{-14} – 10^{-12} g (unit cell)$^{-1}$. The corresponding range of M_u values in the paper is larger than probable M_u values of *E. coli* (10^{-13} – 10^{-12} g (unit cell)$^{-1}$). The lower ($M_u < 10^{-13}$ g (unit cell)$^{-1}$) M_u values were selected in the analysis in order to calculate and visualize theoretical growth boundaries for small cells determined by the available cellular space and membrane surface area, respectively. $N_{cp} = 0$ corresponded to the t_{CDmin} growth boundary. The variation of N_{cp} describes the possibility to increase UC size by increasing the synthesis and accumulation of CP.
The alternative theoretical possibility (besides the variation of N_{cp}) to increase the size of UC mass and load-bearing capacities without changing the expression of CP is to change t_{CD}. It means that the values of t_C and/or t_D must be changed. It must be stressed that the variability of t_C and t_D

is not an abstract choice but those variations have been observed between cell generations even at the same t_{CD} in several theoretical and experimental studies carried out with synchronized cultures[46]. Fluctuations of t_D values were allegedly the principal component causing variations in cell cycle events taking into account the asymmetric distribution function of t_D according to ref. [47]. Also, it has been shown that t_C and t_D values depend on medium composition but are relatively independent of temperature[16]. However, it has been noticed that temperature increase is followed by DNA content increase whereas cell mass distribution does not change. According to the literature, DNA content increase is not related to t_C value changes[48].

The value of t_D can be changed, for example, by modifying the rates of periodic cell division processes. According to the literature, t_D can be divided into 3 phases[17]:

1. A period of about 480 s = 0.1 h preparing for the formation of the constriction.
2. A period of variable length during which constriction begins with a constant probability per unit of time.
3. A termination period of about 540 s = 0.2 h, during which the constriction is completed and the two daughter cells are separated.

t_D has the least effect on other cell parameters as it influences only the value of t_{CD}.

The value of t_C can be changed via k_{dp} (changes in molecular properties of DNA polymerase III) and/or n_{dna} (changes in genome length) according to the Supplementary Eq. (114) of ref. [3]. Note that each parameter affects the model differently. k_{dp} influences also N_{rce} beside t_C according to Supplementary Eq. (23) which can be described by following simple derivation. t_{CD} is expressed by the following equation by combining Supplementary Eq. (114) of ref. [3] and Supplementary Eq. (23) after necessary substitutions of t_C and t_{CD}:

$$t_{CD} = \frac{n_{dna}}{4 \cdot k_{dp}} + t_D = \frac{N_{dna} \cdot n_{dna}}{2 \cdot k_{dp} \cdot N_{rce}} \tag{33}$$

Note that N_{dna} = 1 molecules (genome) cell^{-1} (corresponding range of $C_{dna} = 1.7 \cdot 10^{-9} – 1.7 \cdot 10^{-7}$ mol (genome) L^{-1}) always in UC according to the definition and therefore all other parameters of Supplementary Eq. (33) are independent of N_{dna}. N_{rce} is expressed by the following equation after necessary derivations in Supplementary Eq. (33) and by removing N_{dna}:

$$N_{rce} = \left(\frac{2 \cdot t_D \cdot k_{dp}}{n_{dna}} + \frac{1}{2} \right)^{-1} \tag{34}$$

It can be seen from Supplementary Eqs. (33)-(34) that N_{rce} and k_{dp} are inversely proportional. The value of N_{rce} decreases non-linearly in case of k_{dp} increase and vice versa if t_D is constant. At the same time, the value of F_{rce} increases non-linearly with k_{dp} increase due to the effect of N_{rce} based on the following Supplementary Eq. (35) derived from Supplementary Eq. (33) after necessary substitutions:

$$F_{rce} = \frac{n_{dna}}{\frac{n_{dna}}{4 \cdot k_{dp}} + t_D} \quad (35)$$

In addition, k_{dp} influences also deoxyribonucleotide synthesis balance (Supplementary Eq. (74) of ref. [3]) and energy balance via DNA replication energy costs (Supplementary Eq. (102) of ref. [3]). $N_{enz\_PW3\_r}$ is expressed by following Supplementary Eq. (36) derived from Supplementary Eqs. (74), (114) of ref. [3] and Supplementary Eq. (23):

$$N_{enz\_PW3\_r} = 2 \cdot \frac{k_{dp}}{k_{enz}} \left(\frac{2 \cdot t_D \cdot k_{dp}}{n_{dna}} + \frac{1}{2} \right)^{-1} \quad (36)$$

Note that in the theoretical case of $t_C{'} + t_D{'} = t_C{''} + t_D{''}$, only N_{rce} values are different between these two conditions but other N_{cell_comp} and F_{cell_comp} values are equal. For example, if $2 \cdot k_{dp}{'} = k_{dp}{''}$ then $t_C{'} = 2 \cdot t_C{''}$ and $N_{rce}{'} = 2 \cdot N_{rce}{''}$. At the same time, the difference of $t_D{'}$ and $t_D{''}$ is not proportional but expressed by following Supplementary Eq. (37):

$$t_D{''} - t_D{'} = \frac{n_{dna}}{8 \cdot k_{dp}{'}} \quad (37)$$

n_{dna} is involved besides Supplementary Eq. (114) of ref. [3] and Supplementary Eq. (23) also in Supplementary Eqs. (110)-(111) of ref. [3] and Supplementary Eqs. (31), (43) which means that n_{dna} also affects the value of M_u and the latter influences practically all output parameters of the cell. Note that in the theoretical case of $t_C{'} + t_D{'} = t_C{''} + t_D{''}$, all N_{cell_comp} and F_{cell_comp} values are different between these two conditions. For example, if $2 \cdot n_{dna}{'} = n_{dna}{''}$ then $t_C{'} = 2 \cdot t_C{''}$, $N_{rce}{'} = 2 \cdot N_{rce}{''}$ and $F_{rce}{'} = 2 \cdot F_{rce}{''}$. At the same time, the difference of $t_D{'}$ and $t_D{''}$ is again not proportional but expressed by following Supplementary Eq. (38):

$$t_D{''} - t_D{'} = \frac{n_{dna}{''}}{8 \cdot k_{dp}} \quad (38)$$

In conclusion, t_{CD} can be varied using different input parameters which affect other cell parameters to different extents:

1. t_D is the most neutral and does not affect other cell parameters in UC.
2. k_{dp} influences various cellular parameters including N_{rce}, F_{rce}, $N_{enz\_PW3\_r}$, N_{etc} etc.
3. n_{dna} influences practically all cellular parameters.

Therefore, previously introduced SSUCM-SRS-M input parameters t_{CD} and t_D are not used only as constants with defined values in SSUCM-M but these parameters are also used as independent variables (Supplementary Table 12). Current t_{CD} variations in SSUCM-M calculations were carried out by varying t_D values (Figs. 3-5, 7). That choice was preferred because it enabled to separately determine the effects of t_{CD} changes not shadowed by the effects of other cellular parameters. Also, t_D was the parameter of the cellular process (cell cycle) whereas k_{dp} and n_{dna} were parameters of cell components (RC, genome) and therefore the latter variants expected changes in molecular properties. The varied range for t_D

was approximately 150 – 8000 s = 0.04 – 2.2 h for SSUCM-M which corresponded to the range of t_{CD} = t_{CDmin} – 10^4 s = 2.8 h. Note that the selected ranges of t_{CD} and t_D values in the paper are larger than experimentally observed values of *E. coli*[16-17,21]. The lower and higher values were selected in the analysis in order to calculate and visualize theoretical growth boundaries.

Supplementary Discussion 5.10.3.2.2: Output parameters

Also, the following additional output parameters (Supplementary Table 16) related to CP, cellular fluxes and compositions were used in SSUCM-M.

Supplementary Table 16. Output parameters of SSUCM-M that are missing in SSUCM-SRS-M.

**Cell parameter**	**Symbol**	**Unit**
Total mass of cushioning protein (CP) molecules in the cell [16.1]	M_{cp}	g (tot cp) cell^{-1}
Specific productivity of CP synthesis [16.2]	Q_{cp}	molecules (cp) (g (cell))$^{-1}$ h^{-1}
Number of molecules of mRNA of CP in the cell	N_{mrna_cp}	molecules (mrna cp) cell^{-1}
Number of ribosomes for the synthesis of CP	N_{rs_cp}	molecules (rs cp) cell^{-1}
DNA replication flux [16.3]	F_{rce}	molecules (dnt) s^{-1} cell^{-1}
Flux of reaction r of central metabolic pathway PW_1 [16.4]	$F_{enz\_PW1\_r}$	molecules (metabolite) s^{-1} cell^{-1} reaction^{-1}
Flux of reaction r of deoxyribonucleotide synthesis pathway PW_3 [16.4]	$F_{enz\_PW3\_r}$	molecules (metabolite) s^{-1} cell^{-1} reaction^{-1}
Flux of reaction r of ribonucleotide synthesis pathway PW_4 [16.4]	$F_{enz\_PW4\_r}$	molecules (metabolite) s^{-1} cell^{-1} reaction^{-1}
Flux of reaction r of lipid synthesis pathway PW_5 [16.4]	$F_{enz\_PW5\_r}$	molecules (metabolite) s^{-1} cell^{-1} reaction^{-1}
ATP synthesis flux of electron transport chain (ETC) complex [16.4]	F_{etc}	molecules (atp) s^{-1} cell^{-1}
Lipid synthesis flux [16.4]	F_{lpe}	molecules (lip) s^{-1} cell^{-1}
Substrate transport flux [16.4]	F_{stp}	molecules (substrate) s^{-1} cell^{-1}
Transcription flux [16.4]	F_{rp}	molecules (nt) s^{-1} cell^{-1}
Total dry weight content of replisome complex in the protein fraction [16.5]	$RC\%_{fc}$	% (g (tot rc) (g (tot prot))$^{-1}$)
Total dry weight content of enzymes of central and biosynthesis pathways in the protein fraction [16.5]	$Enz\%_{fc}$	% (g (tot enz) (g (tot prot))$^{-1}$)
Total dry weight content of enzymes of central and biosynthesis pathways in the cell [16.6]	$Enz\%_{mc}$	% (g (tot enz) (g (dw cell))$^{-1}$)
Total dry weight content of ETC complex in the protein fraction [16.5]	$ETC\%_{fc}$	% (g (tot etc) (g (tot prot))$^{-1}$)
Total dry weight content of lipid synthesis enzyme in the protein fraction [16.5]	$Lpe\%_{fc}$	% (g (tot lpe) (g (tot prot))$^{-1}$)
Total dry weight content of mRNAs in the RNA fraction [16.5]	$mRNA\%_{fc}$	% (g (tot mrna) (g (tot rna))$^{-1}$)

Total dry weight content of protein fraction in the cell [16.7]	$PROT\%_{mmc}$	% (g (tot prot) (g (dw cell))$^{-1}$)
Total dry weight content of transporter protein in the protein fraction [16.5]	$STP\%_{fc}$	% (g (tot stp) (g (tot prot))$^{-1}$)
Total dry weight content of CP in the cell [16.6]	$CP\%_{mc}$	% (g (tot cp) (g (dw cell))$^{-1}$)
Total dry weight content of CP in the protein fraction [16.5]	$CP\%_{fc}$	% (g (tot cp) (g (tot prot))$^{-1}$)
Total dry weight content of RNA polymerase complex in the protein fraction [16.5]	$RP\%_{fc}$	% (g (tot rp) (g (tot prot))$^{-1}$)
Total dry weight content of RNA fraction in the cell [16.7]	$RNA\%_{mmc}$	% (g (tot rna) (g (dw cell))$^{-1}$)
Total dry weight content of assembled rRNA complex in the RNA fraction [16.5]	$rRNA\%_{fc}$	% (g (tot rrna) (g (tot rna))$^{-1}$)
Total dry weight content of ribosomal protein complex in the protein fraction [16.5]	$RPC\%_{fc}$	% (g (tot rpc) (g (tot prot))$^{-1}$)
Total dry weight content of tRNA in the RNA fraction [16.5]	$tRNA\%_{fc}$	% (g (tot trna) (g (tot rna))$^{-1}$)
Surface area of the internal layer of cell membrane [16.8]	S_{mem_i}	cm^2 $cell^{-1}$
Surface area of cell membrane covered by membrane proteins [16.9]	S_{prot}	cm^2 (tot mprot)$^{-1}$

16.1 The value of M_{cp} is calculated from the following Supplementary Eq. (39):

$$M_{cp} = N_{cp} \cdot m_{cp} \tag{39}$$

16.2 The value of Q_{cp} is calculated from the Eq. (2).
16.3 The value of F_{rce} is calculated from the following Supplementary Eq. (40):

$$F_{rce} = 2 \cdot N_{rce} \cdot k_{dp} \tag{40}$$

16.4 F_{cell_comp} is calculated from Supplementary Eq. (117) of ref. [3].
16.5 The value of $cell\_comp\%_{fc}$ is calculated from the following Supplementary Eq. (41):

$$cell\_comp\%_{fc} = \frac{N_{cell\_comp} \cdot m_{cell\_comp}}{\sum_{cell\_comp \in mmc\_fraction} \left(N_{cell\_comp} \cdot m_{cell\_comp}\right)} \cdot 100 \tag{41}$$

16.6 The value of $cell\_comp\%_{mc}$ is calculated from the following Supplementary Eq. (42):

$$cell\_comp\%_{mc} = \frac{N_{cell\_comp} \cdot m_{cell\_comp}}{M_u \cdot DWC} \cdot 100 \tag{42}$$

16.7 The value of $cell\_comp\%_{mmc}$ is calculated from the following Supplementary Eq. (43):

$$cell\_comp\%_{mmc} = \frac{\sum_{cell\_comp \in mmc\_fraction} \left(N_{cell\_comp} \cdot m_{cell\_comp}\right)}{M_u \cdot DWC} \cdot 100 \tag{43}$$

16.8 The value of S_{mem_i} is calculated from the following Supplementary Eq. (44):

$$S_{mem\_i} = 2 \cdot \pi \cdot \left(HR + 2\right) \cdot \left(\frac{V_{cyt}}{\left(HR + \frac{4}{3}\right) \cdot \pi} \right)^{\frac{2}{3}} \tag{44}$$

16.9 The value of S_{prot} is calculated from the following Supplementary Eq. (45):

$$S_{prot} = S_{etc} + S_{stp} \tag{45}$$

The values of S_{etc} and S_{stp} are calculated from Supplementary Eq. (125) of ref. [3].

Supplementary Discussion 5.10.3.3: Calculation scheme

The calculation scheme is similar to SSPCM-SRS-M (Supplementary Discussion 5.11.2.3 of ref. [3]). The first step (calculation of input parameter values (masses of molecules, lengths of mRNA molecules etc), solving of equations and calculation of output parameters related to DNA replication and associated polymerization) also involves the calculation of following SSUCM-M specific input parameters: *generic* n_{mrna_cp}, *generic* m_{mrna_cp} and *generic* m_{cp}. The second step (solving the main system of equations) involves almost all equations (except Supplementary Eqs. (74), (87), (94), (108) of ref. [3]) and corresponding output parameters (except $N_{enz\_PW3\_r}$, N_{rs_rc}, N_{mrna_rc}, N_{rce}) mentioned in Supplementary Discussions 5.10.3.1.1-5.10.3.1.8 including also N_{mrna_cp} and N_{rs_cp}. The third step (solving equations and calculation of output parameters related to flux patterns, cellular compositions and geometric properties) also involves all equations and output parameters (except N_{mrna_cp} and N_{rs_cp}) mentioned in Supplementary Table 16.

It must be stressed that the solutions of SSUCM-M at N_{cp} = 0 (growth boundary of smaller UCs) are partially identical to solutions of SSUCM-SRS-M at the same t_{CD} (t_D) value. Although there are differences between both models in terms of cell components and interactions (CP and related cell components in SSUCM-M), the values of all physiological cell response parameters available only in SSUCM-M are 0 at N_{cp} = 0 and the values of remaining output parameters are exactly equal between models.

SUPPLEMENTARY DISCUSSION 5.10.4: DESCRIPTION OF SSUCM-R

The description of the model (SSUCM-R) is based on the full descriptions of previous models (SSPCM-SRS-M, SSPCM-SRS-R, SSUCM-SRS-M and SSUCM-M (Supplementary Discussions 5.11.2 and 5.11.11 of ref. [3], Supplementary Discussions 5.10.1-5.10.3)), only the differences compared with previous models are subsequently presented.

Supplementary Discussion 5.10.4.1: Model components and interactions

The developed SSUCM-R has essentially the same cell components and interactions as in SSPCM-SRS-R and SSUCM-SRS-R. The main difference is the synthesis of CP and corresponding mRNA in the cytoplasmic space of SSUCM-R (Supplementary Fig. 58).

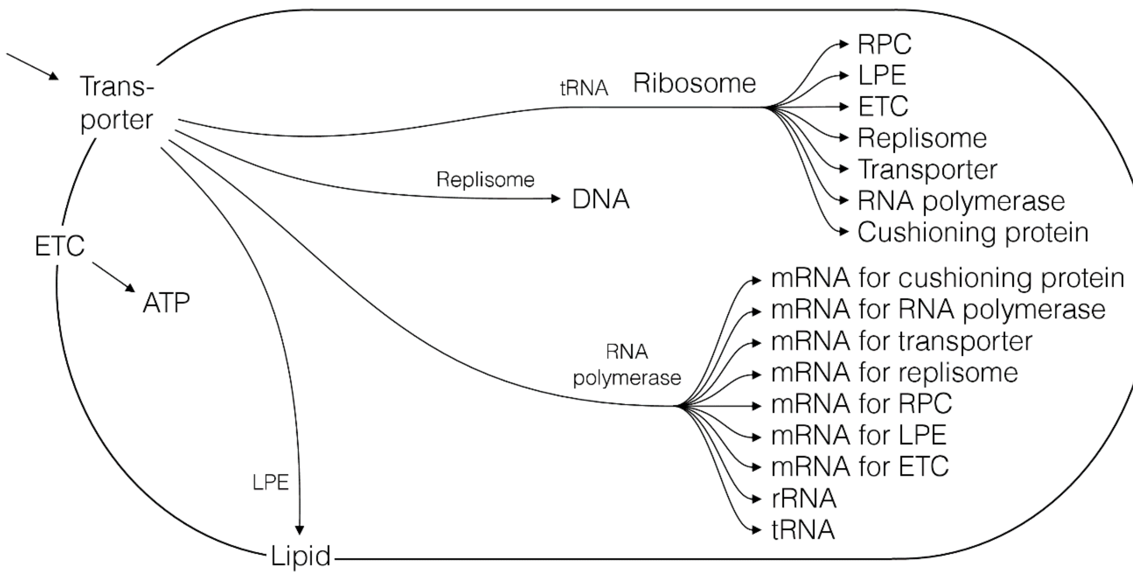

Supplementary Fig. 58. Simplified bacterial unit cell growing on rich medium, corresponds to SSUCM-R. The developed model includes all the main cell components (DNA, RNA, proteins, membrane lipids) and their monomers (nucleotides, amino acids). Most of the cell components are localized in the cytoplasmic space except membrane lipids, transporter proteins and electron transport chain (ETC) complexes that belong to the bilayer cell membrane. The genome of the cell is a bihelix circular chromosome. RNA fraction consists of different types (assembled rRNA complex, universal tRNA (not shown) and mRNAs for all proteins). Different proteins are taken into account based on their functions (polymerases, membrane proteins). The growth medium contains all the monomers for polymerisation processes (nucleotides, amino acids, lipid precursors) that are transported through the membrane by multifunctional membrane transporter. Energy is synthesized by ETC on the membrane. Monomers and energy are further utilized by polymerization processes (replication catalysed by replisome complex, transcription catalysed by RNA polymerase complex, translation catalysed by ribosome (consists of rRNA and ribosomal protein complex (RPC)), membrane lipid synthesis catalysed by lipid synthesis enzyme (LPE)). SSUCM-R includes also cushioning protein (CP) that is not directly necessary for the self-reproduction of the UC but fulfils a cushioning role. CP is accumulated in the cytoplasmic space and it represents unused SRS and cell load components.

Supplementary Discussion 5.10.4.1.1: Metabolic network

The metabolic network is the same as in SSPCM-SRS-R and is described in Supplementary Discussion 5.11.11.1.1 of ref. [3].

Supplementary Discussion 5.10.4.1.2: Protein synthesis

The protein fraction of the UC is comprised of CP (does not have a specific function for self-reproduction and is not part of SRS, cushioning function) and of all different proteins (Supplementary Fig. 58) from SSPCM-SRS-R (Supplementary Discussion 5.11.11.1.2 of ref. [3]). CP was also assumed to be single molecule (monomeric polypeptide chain) without different subunits. The synthesis of proteins is described by Supplementary Eqs. (13)-(14), (16)-(19), (25). N_{rs} is expressed as the sum of all $N_{rs\_cell\_comp}$ (Supplementary Eq. (46)):

$$N_{rs} = N_{rs\_rc} + N_{rs\_rp} + N_{rs\_rpc} + N_{rs\_etc} + N_{rs\_stp} + N_{rs\_lpe} + N_{rs\_cp} \tag{46}$$

Supplementary Discussion 5.10.4.1.3: RNA synthesis

RNA fraction of the UC is comprised of mRNA coding CP and of all different RNAs (Supplementary Fig. 58) from SSPCM-SRS-R (Supplementary Discussion 5.11.11.1.3 of ref. [3]). The mRNA of CP is also assumed to contain only coding regions to simplify the model.
Numbers of all RNA molecules are doubled during the cell cycle by RP complexes carrying out transcription. Different actual steps of transcription and post-transcriptional processes (RNA processing, modifications, charging, degradation etc.) are not described. Instead, there is a heavily simplified transcription process which uses ribonucleotides as substrates and produces RNA molecules (Supplementary Eqs. (47)-(48)):

$$t_{CD} = \frac{N_{rrna} \cdot n_{rrna} + N_{trna} \cdot n_{trna} + \sum_{1}^{7} N_{mrna\_cell\_comp} \cdot n_{mrna\_cell\_comp}}{N_{rp} \cdot k_{rp}} \quad (47)$$

$$\sum_{1}^{7} N_{mrna\_cell\_comp} \cdot m_{mrna\_cell\_comp} = N_{mrna\_rc} \cdot m_{mr\_rc} + N_{mrna\_rp} \cdot m_{mrna\_rp} + N_{mrna\_rpc} \cdot m_{mrna\_rpc} + $$
$$+ N_{mrna\_etc} \cdot m_{mrna\_etc} + N_{mrna\_stp} \cdot m_{mrna\_stp} + N_{mrna\_lpe} \cdot m_{mrna\_lpe} + N_{mrna\_cp} \cdot m_{mrna\_cp} \quad (48)$$

The stoichiometry of the ribosome is described by Supplementary Eq. (92) of ref. [3] and tRNA balances by Supplementary Eq. (93) of ref. [3]. $N_{mrna\_cell\_comp}$ values are determined by translational activity and also by P_{cell_comp} (Supplementary Eqs. (94)-(99) of ref. [3], Supplementary Eq. (30)).

Supplementary Discussion 5.10.4.1.4: Lipid synthesis

The description of lipid synthesis is the same as in SSUCM-SRS-M (Supplementary Discussion 5.10.1.1.4).

Supplementary Discussion 5.10.4.1.5: Energy balance

The description of energy synthesis and consumption balance of the UC is the same as in SSPCM-SRS-R (Supplementary Discussion 5.11.11.1.5 of ref. [3]).

Supplementary Discussion 5.10.4.1.6: Cell geometry

The geometry of the UC is the same as in SSPCM-SRS-M (Supplementary Discussion 5.11.2.1.6 of ref. [3]).

Supplementary Discussion 5.10.4.1.7: Mass balance

M_u can be divided roughly into two parts by cell structures – M_{cyt} and M_{mem} – and it is the sum of masses of all cell components including water (mass of water is described by *approximate DWC*) according to Supplementary Eq. (22). The M_{cyt} equals with the sum of masses of all cell components (including water) localized in cytoplasmic space (Supplementary Eqs. (49)-(50)):

$$M_{cyt} = \frac{N_{dna} \cdot m_{dna} + N_{rc} \cdot m_{rc} + N_{rp} \cdot m_{rp} + N_{rs} \cdot m_{rpc} + N_{lpe} \cdot m_{lpe} + N_{trna} \cdot m_{trna} + N_{rrna} \cdot m_{rrna}}{DWC} + \\ + \frac{N_{cp} \cdot m_{cp} + \sum_{1}^{7} N_{mrna\_cell\_comp} \cdot m_{mrna\_cell\_comp}}{DWC} \quad (49)$$

$$\sum_{1}^{7} N_{mrna\_cell\_comp} \cdot m_{mrna\_cell\_comp} = N_{mrna\_rc} \cdot m_{mrna\_rc} + N_{mrna\_rp} \cdot m_{mrna\_rp} + N_{mrna\_rpc} \cdot m_{mrna\_rpc} + \\ + N_{mrna\_etc} \cdot m_{mrna\_etc} + N_{mrna\_stp} \cdot m_{mrna\_stp} + N_{mrna\_lpe} \cdot m_{mrna\_lpe} + N_{mrna\_cp} \cdot m_{mrna\_cp} \quad (50)$$

Supplementary Discussion 5.10.4.1.8: DNA synthesis

The DNA replication is described in Supplementary Discussion 5.10.1.1.8.

Supplementary Discussion 5.10.4.2: Model parameters

Corresponding input parameters, their values and output parameters of SSUCM-SRS-R (Supplementary Discussion 5.10.2.2) are also used in the current model. Also, additional input (Supplementary Table 15) and output parameters (Supplementary Table 16) related to CP, cellular fluxes and compositions were used in SSUCM-R. Similarly to SSUCM-M, input parameters t_{CD} and t_D are not used only as constants with defined values in SSUCM-R but these parameters are also used as independent variables (Supplementary Tables 12, 15). The varied range of t_D was approximately 0 – 8000 s = 0 – 2.2 h for SSUCM-R which corresponded to the range of t_{CD} = 2320 – 10^4 s = 0.6 – 2.8 h if *approximate* k_{dp} = 1000 molecules (dnt) s^{-1} dp^{-1}.
However, whereas t_D variations enabled to cover the whole growth range (including t_{CDmin}) for SSUCM-M, it was not possible for SSUCM-R due to considerably lower t_{CDmin} values. It means that growth boundaries were not achieved at t_D = 0. Therefore, t_C (Supplementary Discussion 5.10.3.2.1) values had to be additionally varied (increased) at faster growth if $t_{CD\min}$ < 2320 s = 0.6 h. Therefore, k_{dp} was defined as an independent variable in SSUCM-R for a limited growth range. The varied range was approximately 1000 – 2500 molecules (dnt) s^{-1} dp^{-1} that corresponded to the range of $t_{CD} = t_{CDmin}$ – 2320 s = 0.6 h if t_D = 0.
It must be stressed that the selected ranges of t_C and t_D values in the paper are larger than experimentally observed values of *E. coli*[16-17]. The lower (t_C < 1800 s = 0.5 h, t_D < 1200 s = 0.3 h) values were selected in the analysis in order to calculate and visualize theoretical growth boundaries.

Supplementary Discussion 5.10.4.3: Calculation scheme

The calculation scheme is similar to SSPCM-SRS-R (Supplementary Discussion 5.11.11.3 of ref. [3]). The first step (calculation of input parameter values (masses of molecules, lengths of mRNA molecules etc), solving of equations and calculation of output parameters related to DNA replication and associated polymerization) also involves the calculation of following SSUCM-M specific input parameters: *generic* n_{mrna_cp}, *generic* m_{mrna_cp} and *generic* m_{cp}. The second step (solving the main system of equations) involves almost all equations (except Supplementary Eqs. (87), (94), (108) of ref. [3]) and corresponding output parameters (except N_{rs_rc}, N_{mrna_rc}, N_{rce}) mentioned in Supplementary Discussions 5.10.4.1.1-5.10.4.1.8 including also N_{mrna_cp} and N_{rs_cp}. The third step (solving of equations and calculation of output parameters related to flux patterns, cellular compositions and geometric properties) also involves all equations and output parameters (except N_{mrna_cp}, N_{rs_cp}, $F_{enz\_PWi\_r}$ and $Enz\%_{fc}$) mentioned in Supplementary Table 16. As already mentioned in the previous chapter, the

values of t_{CD} of SSUCM-R were varied conditionally based on two steps: varying t_D during slower growth t_{CD} > 2320 s = 0.6 h) and varying k_{dp} at faster growth (t_{CD} < 2320 s = 0.6 h) if $t_D = 0$ and $N_{rc} = N_{rce}$. Note that a similar t_{CD} variation scheme was also used for SSPCM-SRS-R and SSUCM-SRS-R.
It must be stressed that the solutions of SSUCM-R at $N_{cp} = 0$ (growth boundary of smaller UCs) are partially identical to solutions of SSUCM-SRS-R at the same t_{CD} value. Although there are differences between both models in terms of cell components and interactions (CP and related cell components in SSUCM-R), the values of all physiological cell response parameters available only in SSUCM-R are 0 at $N_{cp} = 0$ and the values of remaining output parameters are exactly equal between models.

Supplementary References

1. Abner, K., Aaviksaar, T., Adamberg, K. & Vilu, R. Single-cell model of prokaryotic cell cycle. *J. Theor. Biol.* **341**, 78-87 (2014).
2. Nishimura, Y. & Bailey, J. E. On the dynamics of Cooper-Helmstetter-Donachie procaryote populations. *Math. Biosci.* **51**, 305-328 (1980).
3. Abner, K. et al. Self-reproduction and doubling time limits of different cellular subsystems. *NPJ Syst. Biol. Appl.* **9**, 44 (2023).
4. Bleecken, S. Duplication of the bacterial cell and its initiation. *J. Theor. Biol.* **25**, 137-158 (1969).
5. Cooper, S. & Helmstetter, C. Chromosome replication and the division cycle of *Escherichia coli* B/r. *J. Mol. Biol.* **31**, 519-540 (1968).
6. Sahoo, R. Degradation characteristics of steel making materials during handling. *Powder Technol.* **176**, 77-87 (2007).
7. Kart, C. S., Metress, E. K. & Metress, S. P. Part II: Biomedical aspects of aging. Chapter 6: The aging skeletal system. in *Human Aging and Chronic Diseases* 87-92 (Jones and Bartlett Publishers, Sudbury, 1992).
8. Hayssen, V. & Orr, T. J. Part I: The reproducing female. Chapter 4: Anatomy. in *Reproduction in Mammals: The Female Perspective* 37-67 (Johns Hopkins University Press, Baltimore, 2017).
9. Schrum, J. P, Zhu, T. F. & Szostak J. W. The origins of cellular life. *Cold Spring Harb. Perspect. Biol.* **2**, 002212 (2010).
10. Kuzenkov, O. & Morozov, A. Towards the construction of a mathematically rigorous framework for the modelling of evolutionary fitness. *Bull. Math. Biol.* **81**, 4675-4700 (2019).
11. Sakuma, Y. & Imai, M. From vesicles to protocells: the roles of amphiphilic molecules. *Life* **5**, 651-675 (2015).
12. Ueno, T. Functionalization of viral protein assemblies by self-assembly reactions. *J. Mater. Chem.* **18**, 3741-3745 (2008).
13. Morçöl, G. Chapter 4: Self-Organization. in *A Complexity Theory for Public Policy* 93-121 (Routledge, New York, 2012).
14. Volkmer, B. & Heinemann, M. Condition-dependent cell volume and concentration of *Escherichia coli* to facilitate data conversion for systems biology modeling. *PLoS One* **6**, e23126 (2011).
15. Koch, A. L. & Blumberg, G. Distribution of bacteria in the velocity gradient centrifuge. *Biophys. J.* **16**, 389-405 (1976).

16. Stokke, C., Flåtten, I. & Skarstad K. An easy-to-use simulation program demonstrates variations in bacterial cell cycle parameters depending on medium and temperature. *PLoS One* **7**, e30981 (2012).
17. Bremer, H. & Dennis, P. P. Modulation of chemical composition and other parameters of the cell at different exponential growth rates. *EcoSal. Plus* **3**, 10.1128/ecosal.5.2.3 (2008).
18. Si, F. et al. Invariance of initiation mass and predictability of cell size in *Escherichia coli*. *Curr. Biol.* **27**, 1278-1287 (2017).
19. Bipatnath, M., Dennis, P. P. & Bremer, H. Initiation and velocity of chromosome replication in *Escherichia coli* B/r and K-12. *J. Bacteriol.* **180**, 265-273 (1998).
20. Zhu, M., Dai, X., Guo, W., Ge, Z., Yang, M., Wang, H. & Wang, Y. P. Manipulating the bacterial cell cycle and cell size by titrating the expression of ribonucleotide reductase. *mBio* **8**, e01741-17 (2017).
21. Dai, X. & Zhu, M. High osmolarity modulates bacterial cell size through reducing initiation volume in *Escherichia coli*. *mSphere* **3**, e00430-18 (2018).
22. Donachie, W. D. Relationship between cell size and time of initiation of DNA replication. *Nature* **219**, 1077-1079 (1968).
23. Churchward, G., Estiva, E. & Bremer, H. Growth rate-dependent control of chromosome replication initiation in *Escherichia coli*. *J. Bacteriol.* **145**, 1232-1238 (1981).
24. Wold, S., Skarstad, K., Steen, H. B., Stokke, T. & Boye E. The initiation mass for DNA replication in *Escherichia coli* K-12 is dependent on growth rate. *EMBO J.* **13**, 2097-2102 (1994).
25. DeLong, J. P., Okie, J. G., Moses, M. E., Sibly, R.M. & Brown, J. H. Shifts in metabolic scaling, production, and efficiency across major evolutionary transitions of life. *Proc. Natl. Acad. Sci. U. S. A.* **107**, 12941-12945 (2010).
26. Koch, A. L. What size should a bacterium be? A question of scale. *Annu. Rev. Microbiol.* **50**, 317-348 (1996).
27. Svenstrup, H. F., Fedder, J., Abraham-Peskir, J., Birkelund, S. & Christiansen, G. *Mycoplasma genitalium* attaches to human spermatozoa. *Hum. Reprod.* **18**, 2103-2109 (2003).
28. Karr, J. R. et al. A whole-cell computational model predicts phenotype from genotype. *Cell* **150**, 389-401 (2012).
29. Luef, B. et al. Diverse uncultivated ultra-small bacterial cells in groundwater. *Nat. Commun.* **6**, 6372 (2015).
30. Comolli, L. R., Baker, B. J., Downing, K. H., Siegerist, C. E. & Banfield, J. F. Three-dimensional analysis of the structure and ecology of a novel, ultra-small archaeon. *ISME J.* **3**, 159-167 (2009).
31. Ghuneim, L. J., Jones, D. L., Golyshin, P. N. & Golyshina, O. V. Nano-sized and filterable bacteria and archaea: biodiversity and function. *Front. Microbiol.* **9**, 1971 (2018).
32. Kempes, C. P., Wang, L., Amend, J. P., Doyle, J. & Hoehler, T. Evolutionary tradeoffs in cellular composition across diverse bacteria. *ISME J.* **10**, 2145-2157 (2016).
33. Dusenbery, D. B. Minimum size limit for useful locomotion by free-swimming microbes. *Proc. Natl. Acad. Sci. U. S. A.* **94**, 10949-10954 (1997).
34. Belliveau, N. M. et al. Fundamental limits on the rate of bacterial growth and their influence on proteomic composition. *Cell Syst.* **12**, 924-944 (2021).
35. Berkhout, J. et al. How biochemical constraints of cellular growth shape evolutionary adaptations in metabolism. *Genetics* **194**, 505-512 (2013).

36. Metzl-Raz, E. et al. Principles of cellular resource allocation revealed by condition-dependent proteome profiling. *Elife* **6**, e28034 (2017).
37. Levine, E. & Hwa, T. Stochastic fluctuations in metabolic pathways. *Proc. Natl. Acad. Sci. U. S. A.* **104**, 9224-9229 (2007).
38. Vazquez, A. Optimal macromolecular density in the cell. *Proc. Natl. Acad. Sci. U. S. A.* **109**, E533 (2012).
39. Schuster, S., Pfeiffer, T. & Fell, D. A. Is maximization of molar yield in metabolic networks favoured by evolution? *J. Theor. Biol.* **252**, 497-504 (2008).
40. Schultz, A. & Qutub, A. A. Predicting internal cell fluxes at sub-optimal growth. *BMC Syst. Biol.* **9**, 18 (2015).
41. Klamt, S., Müller, S., Regensburger, G. & Zanghellini, J. A mathematical framework for yield (vs. rate) optimization in constraint-based modeling and applications in metabolic engineering. *Metab. Eng.* **47**, 153-169 (2018).
42. Elsemman, I. E. et al. Whole-cell modeling in yeast predicts compartment-specific proteome constraints that drive metabolic strategies. *Nat. Commun.* **13**, 801 (2022).
43. Gerdes, S. Y. et al. Experimental determination and system level analysis of essential genes in *Escherichia coli* MG1655. *J. Bacteriol.* **185**, 5673-5684 (2003).
44. Molenaar, D., van Berlo, R., de Ridder, D. & Teusink B. Shifts in growth strategies reflect tradeoffs in cellular economics. *Mol. Syst. Biol.* **5**, 323 (2009).
45. Bertaux, F., von Kügelgen, J., Marguerat, S. & Shahrezaei, V. A bacterial size law revealed by a coarse-grained model of cell physiology. *PLoS Comput. Biol.* **16**, e1008245 (2020).
46. Bremer, H. & Chuang, L. The cell cycle in *Escherichia coli* B/r. *J. Theor. Biol.* **88**, 47-81 (1981).
47. Bremer, H. & Chuang, L. Cell division after inhibition of chromosome replication in *Escherichia coli*. *J. Theor. Biol.* **93**, 909-926 (1981).
48. Bernander, R. & Nordström, K. Chromosome replication does not trigger cell division in *E. coli*. *Cell* **60**, 365-374 (1990).
