# Supplementary material for: The design of unit cells by combining the self-reproduction systems and metabolic cushioning loads
Source: Commun Biol. 2025 Feb 15;8:241. doi: 10.1038/s42003-025-07655-2 (PMC11830011; doi:10.1038/s42003-025-07655-2)
Supplement: Supplementary file 2 — Description of Additional Supplementary File [file 42003_2025_7655_MOESM2_ESM.pdf]

Description Of Additional Supplementary File

File name: Supplementary Data

Description: Numerical source data of Figures 1-7 in txt-format.
